# Alternative splicing fine-tunes modular protein interfaces to control protein localization

**DOI:** 10.1038/s41594-026-01875-8

**Published:** 2026-10-14

**Authors:** Peter Kjer-Hansen, Karina Pazaky, Daisy Kavanagh, Helen E. King, Savannah O’Connell, Gabriela Santos-Rodriguez, Abigail K. Grootveld, Helaine Graziele Santos Vieira, Scott Berry, Javier Fernandez-Chamorro, Robert J. Weatheritt

**Affiliations:** 1 School of Biotechnology and Biomolecular Sciences, University of New South Wales, Sydney, New South Wales Australia; 2 St. Vincent’s Healthcare Clinical Campus, School of Clinical Medicine, Faculty of Medicine and Health, UNSW Sydney, Darlinghurst, New South Wales Australia; 3 EMBL Australia, Garvan Institute of Medical Research, Darlinghurst, New South Wales Australia; 4 Precision Immunology Program, Garvan Institute of Medical Research, Sydney, New South Wales Australia; 5 UNSW RNA Institute, University of New South Wales, Sydney, New South Wales Australia; 6 EMBL Australia Node in Single Molecule Science, University of New South Wales, Sydney, New South Wales Australia; 7 Department of Molecular Medicine, School of Biomedical Sciences, University of New South Wales, Sydney, New South Wales Australia

**Keywords:** RNA, Proteomics, Systems biology, Proteins, RNA splicing

## Abstract

Alternative splicing provides a pervasive means to expand proteome complexity, yet how it reorganizes protein interactions and cellular localization remains unclear. Here, we show that tissue-regulated longer exons preferably reshape local interaction patterns, whereas microexons fine-tune key interfaces linking distinct cellular processes. Integration of subcellular localization data further indicates that such rewiring can redistribute proteins within cells. By using a high-content imaging approach to systematically evaluate the influence of individual exons on protein localization, we show that 38% of the tested isoform pairs alter localization, with microexons, albeit typically shorter than five amino acids, accounting for a substantial proportion of these effects. Bioinformatic and structural analyses demonstrate that microexons can extend secondary structural regions and reposition charged residues, suggesting a potential to modulate local electrostatic environments. Consistent with this, biochemical analysis of a 4-aa microexon in sorting nexin 2, identified in our screen, confirms that residue insertion rather than side-chain chemistry drives key differences in protein localization through repositioning of a flanking, charged residue. Together, these findings suggest that tissue-regulated alternative splicing fine-tunes interface architecture, thus regulating protein assembly, localization and proteome organization.

## Abstract (web summary)

Kjer-Hansen et al. show that alternative splicing reshapes how proteins interact and where they localize in cells, with tiny microexons making a disproportionate contribution to protein organization and cellular function.

## Main

Alternative splicing (AS) occurs in nearly all multiexonic human genes and can alter protein-coding sequences^1–3^. Although the contribution of AS to proteome diversity has been debated^4,5^, recent efforts have shown that most well-expressed alternative transcripts give rise to protein isoforms detectable by mass spectrometry (MS)^6,7^, indicating the existence of vast AS-enabled protein diversity. Case studies demonstrate AS-derived proteins influence diverse cellular processes with wide-ranging biological consequences^8–10^ but the functions of most isoforms remain unclear, making their characterization important for both basic biology and therapeutic development^11^.

High-throughput examination of AS-derived protein isoforms has mainly focused on protein–protein interactions (PPIs)^12–14^, especially binary PPIs^12,14^, which have shown that AS frequently rewires protein interactomes, with most isoforms having distinct interactomes^14^. These analyses corroborate earlier observations that tissue-regulated AS is enriched in intrinsically disordered regions^15^ containing short linear interaction motifs^12,16,17^ that mediate PPIs^18^. This suggests that AS frequently enables a protein to function in new contexts through altered interactions while maintaining its core functions^10^.

In multimodular proteins, discrete binding motifs and globular domains combine in specific configurations that dictate molecular specificity, partner recruitment and subcellular targeting^19–22^. Through the selective inclusion or exclusion of interaction modules, AS can potentially remodel these configurations and modulate the assembly and dynamics of protein complexes. However, how interface-level remodeling shapes proteome organization and whether it contributes to the spatial and functional organization of proteins within the cell has yet to be established.

To address this, we combined interface-resolved network modeling, high-content imaging of protein isoforms, machine-learning-based analysis, structural bioinformatics and biochemical approaches to examine how AS shapes protein connectivity, localization and molecular interfaces. We reveal that tissue-specific splicing events preferentially fine-tune the modular architecture of multidomain proteins, enabling local rewiring of interaction networks. These features are exon type dependent, with longer exons predominately affecting local interaction patterns, whereas microexons fine-tune interfaces linking distinct cellular processes. Notably, both exon types exert cellular effects, with many of the tested isoform pairs (16/42) displaying altered subcellular localization. Structural and biochemical analyses further showed that microexons within ordered regions, especially α-helices, influence protein architecture in two principal ways: by disrupting helical continuity to generate or extend loops or by lengthening existing helices. Moreover, microexons often occur near charged residues and can remodel interaction interfaces by displacing these residues, as exemplified by two SNX2 isoforms. Together, these analyses establish how AS fine-tunes protein assembly and deployment across structural, spatial and networks levels.

## Results

### Tissue-specific splicing events refine interfaces within pathways, whereas more tissue-wide events regulate long-range interactions

**Fig. 1:**
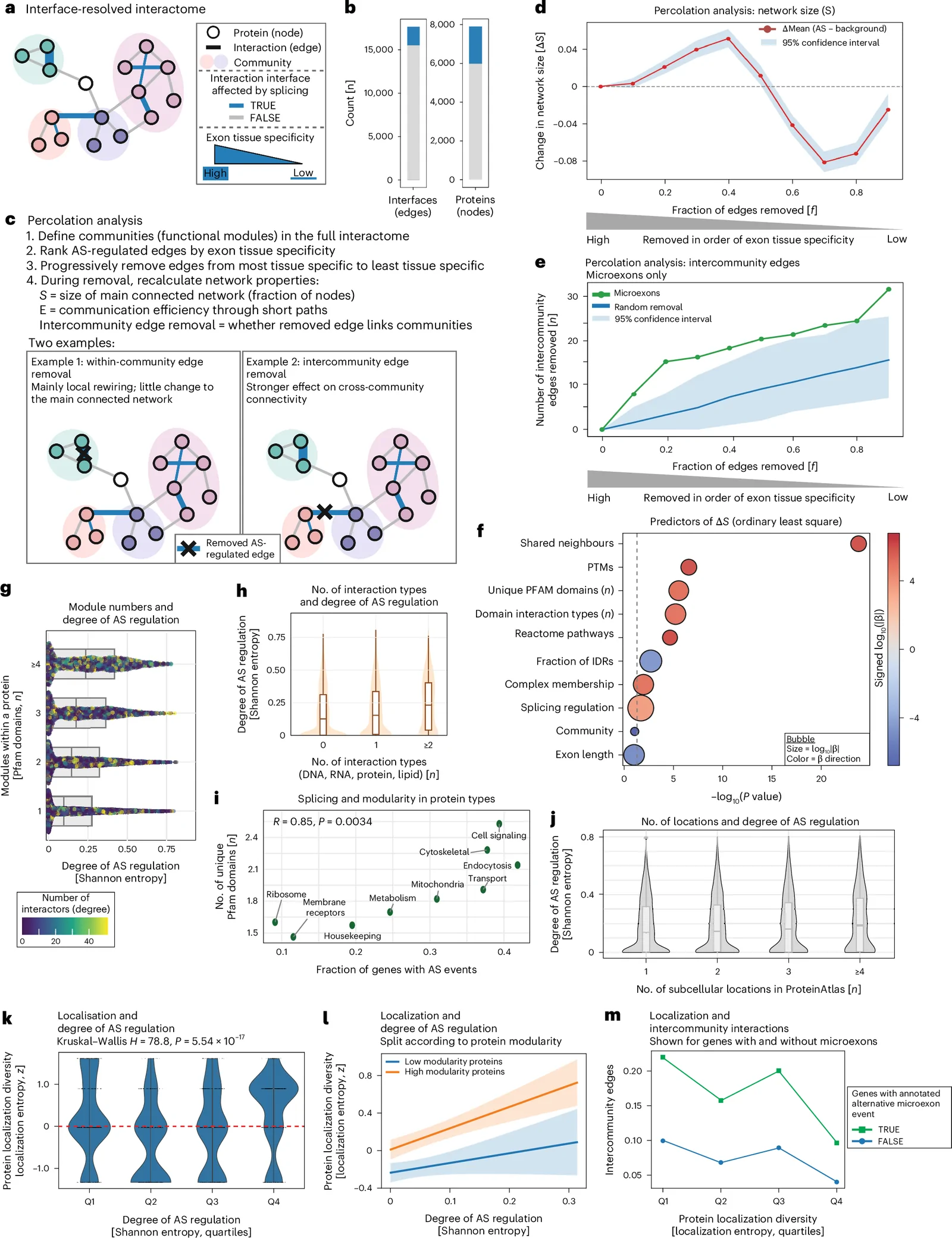
AS can rewire protein networks and correlates with protein multimodularity and diversification of protein subcellular localization. **a**, Schematic of the interface-resolved protein interactome. Nodes represent proteins, edges represent annotated PPIs, shaded circles indicate network communities and blue edges indicate AS-affected interaction interfaces. The blue gradient denotes exon tissue specificity. Created using BioRender.com; Kjer-Hansen, P. https://www.BioRender.com/i6qcxf2 (2026). **b**, Bar plots showing the number of AS-regulated (blue) and non-AS-regulated edges (interactions) and nodes (proteins) within the network. **c**, Overview of percolation analysis used to examine how AS-regulated interfaces affect network organization. Communities represent densely connected groups of proteins and often represent functional modules, such as proteins in the same pathway. Created using BioRender.com; Kjer-Hansen, P. https://www.BioRender.com/i6qcxf2 (2026). **d**, Percolation analysis of network size (relative to complete network) (*S*) during progressive removal of AS-regulated edges. The red line indicates the mean difference in network size (Δ*S* = *S*_AS_ − *S*_null_) as a function of the fraction of AS-regulated edges (**f**) progressively removed from most tissue-specific to least tissue-specific events. Shading denotes the 95% confidence interval; the gray dashed line denotes Δ*S* = 0 (no difference). Null background edges correspond to non-AS interactions randomly selected to match the degree distribution of AS-regulated edges. Positive Δ*S* values indicate the removal of AS-regulated edges causes less network fragmentation than expected under the null. **e**, Percolation analysis of intercommunity connectivity during progressive removal of microexon-regulated interfaces. The green line shows the number of intercommunity edges removed as a function of the fraction of microexon-regulated edges (**f**) progressively removed from most tissue-specific to least tissue-specific events. The blue line corresponds to the mean of non-AS interactions randomly selected to match the degree distribution of AS-regulated edges. Shading denotes the 95% confidence interval. Values above the null indicate that microexon-regulated interfaces are enriched among edges linking distinct communities. **f**, Bubble plot showing OLS regression model coefficients for top ten structural, biophysical and network features (*y* axis) predictors of Δ*S*. Features are ordered by statistical significance (*x* axis). The bubble size reflects effect size (log_10_|β|). The color indicates the β coefficient direction (red = positive, blue = negative). **g**, Violin and box plots showing the distribution of AS regulation (Shannon entropy) for proteins with different numbers of Pfam domains. Each dot represents a protein, colored by its total number of interaction partners (degree). Violin plots show the data distribution. Box plots show the median (center line) and interquartile range (IQR) (box ends) and whiskers show observations within 1.5× the IQR. Group 1, *n* = 589; group 2, *n* = 462; group 3, *n* = 304; group ≥4, *n* = 321). **h**, Violin and box plots showing the distribution of AS regulation (Shannon entropy) for proteins with different numbers of interaction substrate types (ability to interact with protein, DNA, RNA and lipids based on domain annotation; Methods). A description of box plots is provided in **g** (0, *n* = 1,122; 1, *n* = 396; >2, *n* = 158). **i**, Scatter plot showing the mean number of unique domains versus the fraction of genes per functional group with detected AS events. *R* and *P* were calculated using a two-sided Pearson correlation test. **j**, Violin and box plots showing the distribution of AS regulation (Shannon entropy) as a function of the number of annotated subcellular locations per protein (Human Protein Atlas) (group 1, *n* = 535; group 2, *n* = 501; group 3, *n* = 242; group >4, *n* = 167). A description of box plots is provided in **g**. **k**, Violin and box plots showing the distribution of localization diversity (localization entropy) of proteins when divided into quartiles on the basis of their degree of AS regulation (Shannon entropy) (*n* = 1,445). A description of box plots is provided in **g**. **l**, Plot showing the relationship between AS regulation (*x* axis) and localization diversity (*y* axis) for proteins with low versus high modularity. Regression lines are plotted for proteins ±1 s.d. of the mean *z*-transformed number of Pfam domains (PFAMz), representing proteins with fewer and more modular domains. Shading denotes the 95% confidence intervals. **m**, Marginal effects plot showing the interaction between subcellular localization diversity and microexon-containing proteins on intercommunity connectivity. The *y* axis indicates model-predicted probability of an interface connecting distinct functional communities, plotted across increasing levels of subcellular localization diversity (localization entropy) (*x* axis). Lines represent microexon-containing proteins (green) and non-microexon-containing proteins (blue). Source data

To investigate how AS reshapes protein interactions at the level of mapped interaction interfaces, we constructed an interface-resolved human interactome by integrating interface annotations from UniProt^23^, DOMINO^24^, IntAct^25^, 3DID^26,27^ and ELM^28^. In this network, each node represents a protein and each edge represents an experimentally supported PPI where the interface region was mapped onto UniProt canonical sequences (Fig. 1a). This network comprised 17,660 experimentally defined interfaces (Supplementary Table 1), providing a detailed framework for assessing how exon choice influences protein connectivity. Integrating differential splicing analysis^29,30^ across 75 tissues and cell types, (change in percent spliced in (PSI) > 20) we identified frame-preserving tissue-regulated AS events whose encoded protein regions overlapped at least one mapped interface residue or interval. This ensured that only exons known to alter specific contact surfaces, rather than entire proteins, were considered.

**Extended Data Fig. 1:**
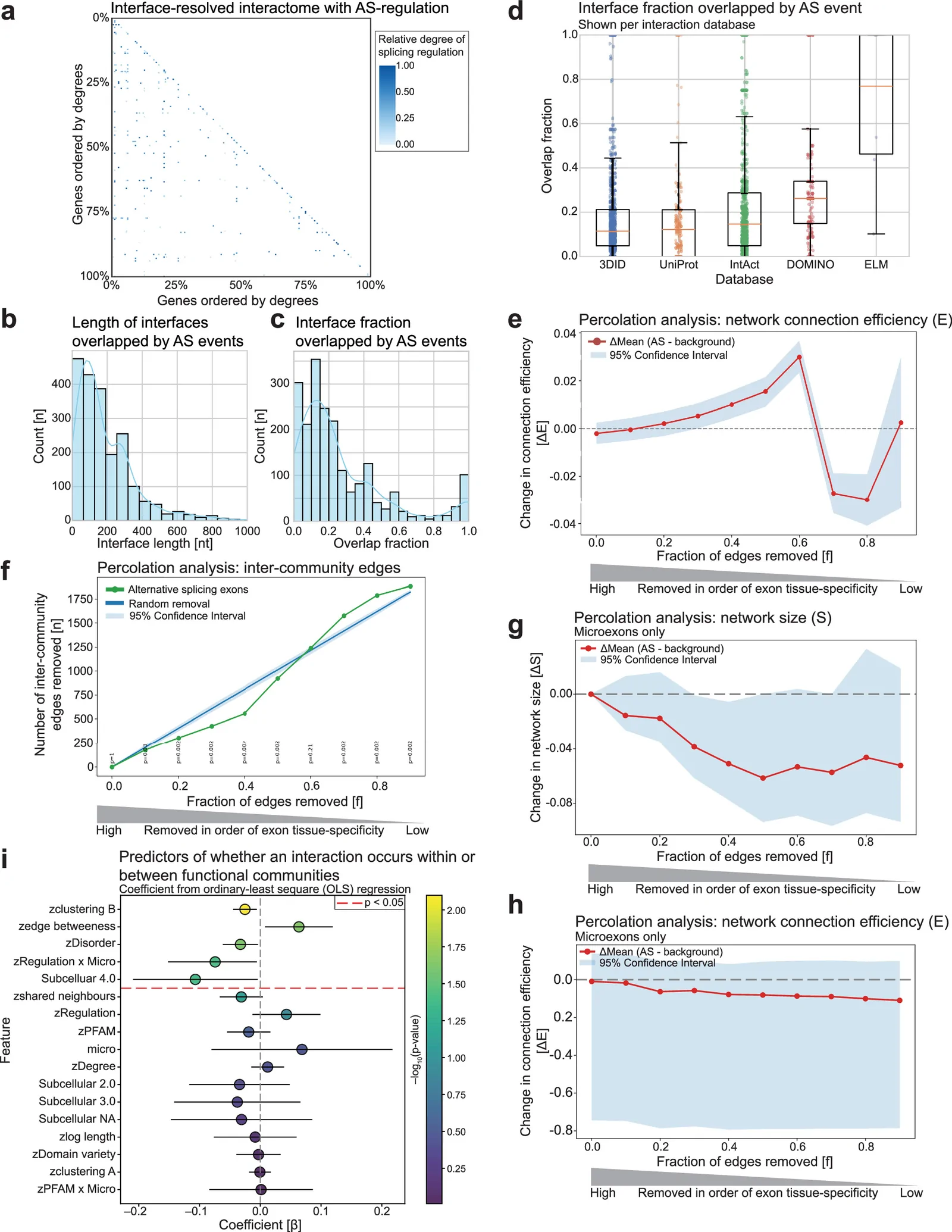
Alternative splicing-regulated interactions in protein network analysis. **a**, Triangular adjacency heatmap of the interface-resolved interactome ordered by total node degree (top 200 proteins). Cells represent PPIs; colour encodes interaction diversity (network entropy; percentile scaled), with non-AS edges in white. **b**, Length distribution of annotated interfaces overlapping alternative exons. Curves indicate kernel density estimates. **c**, Distribution of the fraction of each interface overlapped by the alternative exon. (0, no overlap; 1, complete overlap). Curves show kernel density estimates. **d**, Overlap fractions between alternative exons and annotated interaction interfaces from 3DID, UniProt, IntAct, DOMINO and ELM. Points represent individual exon–interface pairs. Boxplots show median, IQR and 1.5×IQR whiskers. **e**, Percolation analysis of network communication efficiency (ΔE = E_AS_–E_null_) during progressive removal of AS-regulated edges, ordered from the most to least tissue-specific events. Shading indicates the 95% confidence interval, and the dashed line indicates ΔE = 0. Null edges were degree-matched non-AS interactions. Positive values indicate higher communication efficiency than expected by chance. **f**, Percolation analysis of inter-community connectivity during progressive removal of AS-regulated edges. Green indicates observed inter-community edge removal and blue the degree-matched null mean (1,000 permutations; 95% confidence interval). Values above the null indicate preferential removal of connections between communities. **g**, Percolation analysis of network size (ΔS = S_AS_–S_null_) during progressive removal of microexon-regulated edges. Shading indicates the 95% confidence interval, and the dashed line indicates ΔS = 0 (no difference). Positive values indicate less network fragmentation than expected under the degree-matched null. **h**, Percolation analysis of network communication efficiency (ΔE = E_AS_–E_null_) during progressive removal of microexon-regulated edges. Plot elements are as in e. **I)** Multivariable linear regression identifying features associated with interactions between versus within functional communities. Shown are standardised regression coefficients with 95% confidence intervals, ordered and coloured by statistical significance (–log_10_ p-value). Positive coefficients indicate features are associated with inter-community interactions, whereas negative coefficients correspond to features enriched within community modules. Source data

By this definition, approximately 6.5% of all interfaces were subject to splicing regulation (Fig. 1b, Extended Data Fig. 1a–d and Methods) defining a proteome-wide network of interface-level variation. Consistent with previous observations^12,16,17^, AS-regulated interfaces were enriched at high-degree modular hubs where interaction diversity is greatest (Extended Data Fig. 1a), indicating that splicing acts locally within highly connected neighborhoods rather than globally rewiring the network.

We next investigated whether AS reshapes interaction topology at the systems level by examining how AS-regulated interfaces contribute to the organization of the interactome network using percolation analysis and intercommunity edge analysis (Fig. 1c). Here, AS-regulated edges were progressively removed from the interface-resolved interactome in order of decreasing exon tissue specificity, beginning with the most tissue-specific exons and ending with more tissue-wide events, defined here as exons detected across many tissues but still showing substantial tissue-to-tissue inclusion differences. At each removal step, we quantified two network properties: *S*, the fraction of nodes remaining in the main connected part of the interactome, which captures whether the network stays largely intact or breaks into smaller disconnected pieces, and *E*, the approximate global efficiency, which captures how efficiently information can still travel across the remaining network. These observed trajectories were then compared to degree-matched random edge-removal null models to determine whether AS-regulated interfaces have nonrandom effects on network structure (Δ*S* and Δ*E*).

Removal of the most tissue-specific AS resulted in a significant, positive Δ*S*, indicating less fragmentation than expected by chance (Fig. 1d; *P* < 0.001, empirical two-sided test). Continued edge removal, however, resulted in a sigmoidal shaped Δ*S* trajectory, suggesting that the effect is not uniform across the ranked AS events, with removal of the most tissue-specific edges having relatively little effect on the main connected part of the interactome, whereas later removal produces a sharper transition once a more critical set of integrative edges is affected. Together with the absence of a significant effect on *E* (Extended Data Fig. 1e; *P* > 0.05), this pattern is most consistent with highly tissue-specific exons preferentially rewiring interactions locally rather than impacting network-wide communication. We next examined whether AS-regulated edges preferentially connect distinct functional communities, wherein groups of proteins are more densely connected to one another than to the rest of the interactome. Consistent with network *S*, tissue-specific AS-regulated edges were depleted among edges linking distinct communities, whereas tissue-wide AS events significantly altered the fraction of edges linking communities (Extended Data Fig. 1f; *P* < 0.002, empirical two-sided test). This pattern suggests that tissue-wide events may contribute more strongly to maintaining connections between functional modules. Taken together, these findings support a model where tissue-specific exons mainly fine-tune local network structure, whereas more tissue-wide exons have greater influence on intercommunity integration.

Among tissue-specific events, microexons (<30 nt) represent a distinct class of AS events in terms of effect on protein structure. Unlike longer exons, microexons frequently alter the structured domains of proteins^29^, suggesting that they may exert different effects on interaction topology. Therefore, we tested whether microexons displayed a distinct pattern of network regulation. Whereas tissue-specific exons were associated primarily with local rewiring, microexons were significantly enriched among interfaces linking distinct functional communities (Fig. 1e and Extended Data Fig. 1g,h; *P* < 0.004, empirical two-sided test). This suggests that microexons preferentially regulate connections between pathways or complexes rather than acting only within local modules. Ordinary least squares (OLS) modeling (Methods) further showed that intercommunity connectivity depended on both tissue regulation and local topology, with a significant regulation × microexon interaction term (Extended Data Fig. 1i; *P* < 0.03, OLS *t*-test). Together, these observations are consistent with microexons representing a specialized subset of tissue-specific exons with a disproportionate potential to occupy network positions that modulate crosstalk between different complexes or subcellular locations.

### Tissue-specific splicing preferentially targets multidomain proteins with broad interaction and localization repertoires

We next asked whether the network effects associated with tissue-specific splicing were linked to specific protein properties. Regression and machine learning analyses identified multidomain architecture^31^, interaction substrate diversity, post-translational modification^32^ density and shared neighbor connectivity as the strongest predictors of Δ*S* (Fig. 1f; *P* < 10^−5^, heteroscedasticity-robust OLS (HC3) Wald test), suggesting that tissue-specific AS may preferentially affect modular, multifunctional proteins. To determine whether these trends extended beyond the interface-overlapping subset, we then analyzed all frame-preserving, tissue-specific AS events irrespective of direct interface overlap. Consistent with the interface-level results, tissue-specific events were enriched in multidomain proteins (Fig. 1g; *P* < 3.47 × 10^−59^, Kruskal–Wallis test) and in proteins with greater interaction substrate diversity (Fig. 1h; *P* < 1.62 × 10^−16^, Kruskal–Wallis test). Similarly, when proteins were grouped into broad categories on the basis of cellular function, AS propensity increased with the median number of unique domains per protein (Fig. 1i; *R* = 0.85, *P* = 0.0034, Spearman’s rank correlation). Together, these analyses indicate that tissue-specific AS preferentially affects modular, multifunctional proteins that are likely to tolerate local changes in binding surfaces or domain arrangement without disrupting core activity.

**Extended Data Fig. 2:**
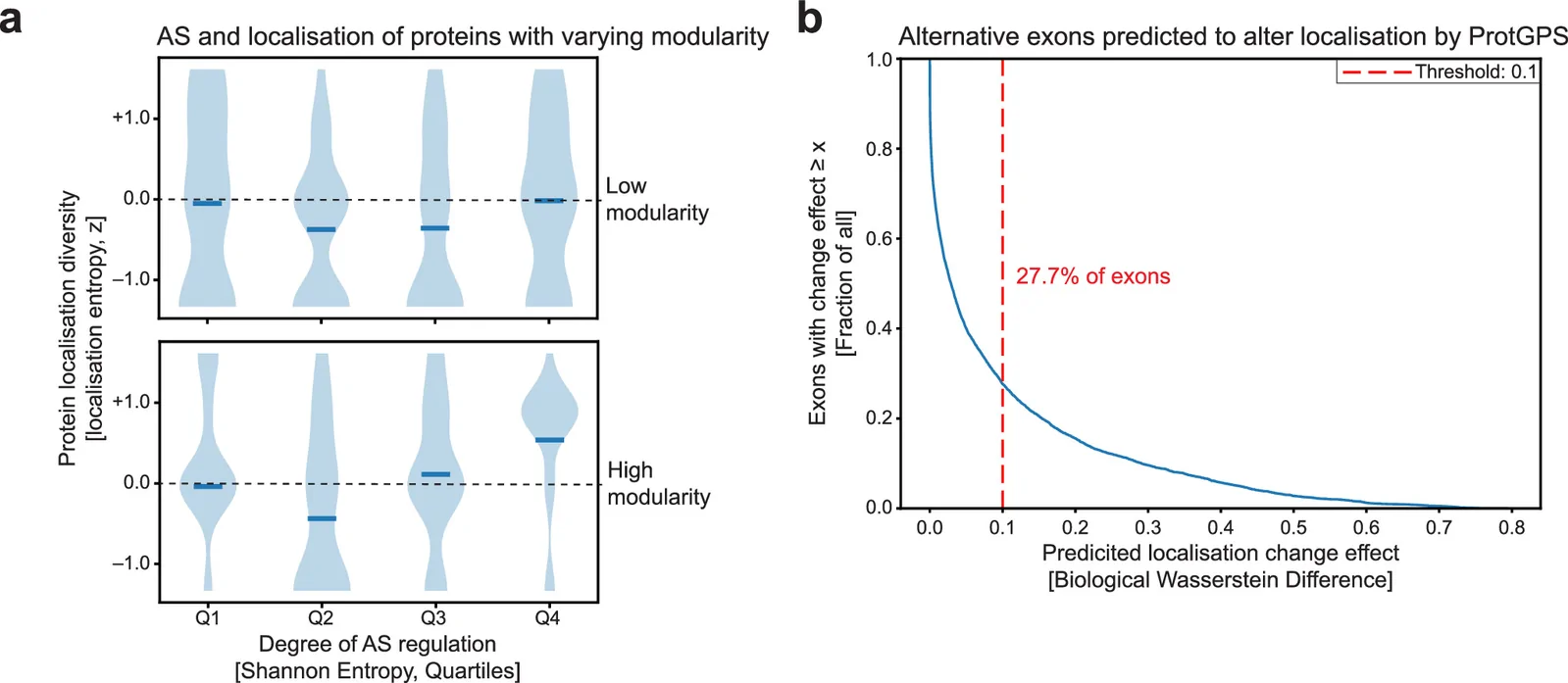
Tissue-regulated exons may alter localisation diversity. **a**, Violin plots showing the distribution of proteins’ localisation diversity (localisation entropy) when proteins are sorted into quartiles based on degree of splicing regulation (Shannon Entropy). Localisation diversity (y-axis, z-score) quantifies the number of distinct subcellular compartments in which each protein is detected (Human Protein Atlas). The analysis was performed separately for low (left) and high (right) modularity proteins regarding their number of Pfam domains. These groups were defined as proteins with one standard deviation below ( − 1 SD) and above ( + 1 SD) the mean z-transformed number of Pfam domains (PFAMz), respectively. **b**, ProtGPS-predicted localisation differences to 12 subcellular compartments for pairs of protein isoforms that differ in the inclusion or exclusion of a tissue-regulated exon. The predicted values are shown as a cumulative distribution plot of the biological Wasserstein distance (WD) values for tested isoform pair (n = 7,979) and shows the fraction of isoform pairs with localisation differences ≥ to a given magnitude. Isoform pairs with WD ≥ 0.1 (red, dashed line) are considered to show localisation differences, and so the splicing event is considered localisation-affecting. The used cutoff was the default ProtGPS cutoff. Source data

A potential consequence of altered interaction behavior is altered subcellular localization^33^. Therefore, we next asked whether proteins regulated by tissue-specific AS also show greater localization diversity. Integrating subcellular localization annotations from the Human Protein Atlas^34^, we found that proteins containing frame-preserving tissue-specific AS events were enriched among proteins localized to multiple cellular compartments (Fig. 1j; *P* < 0.0029, Kruskal–Wallis test). Likewise, proteins with tissue-specific interface-regulating events showed higher localization diversity (Fig. 1k; all tissue-specific exons *P* < 5.543 × 10^−17^, Kruskal–Wallis test across regulation bins). This association was strongest among highly modular proteins (Fig. 1l; β = 1.83, *P* = 9.5 × 10^−13^, OLS *t*-test) and remained significant when proteins were analyzed separately according to modularity (Extended Data Fig. 2a; high-modularity β = 0.58, *P* = 2.7 × 10^−15^, OLS *t*-test). Microexon-containing proteins also showed elevated localization diversity, although this association was attenuated at intercommunity interfaces (Fig. 1m; β = 0.49, *P* < 0.001, OLS *t*-test). Overall, these data support a link between tissue-specific AS and broader subcellular localization diversity of proteins.

To move from this gene-level association to the level of individual splicing events, we next asked how often tissue-regulated exons might influence localization by predicting localization shifts between matched isoforms with and without the regulated exon using ProtGPS^35^. By this approach, 27.7% of tested exons (*n* = 7,979) were associated with a predicted redistribution across subcellular compartments (Extended Data Fig. 2b). This suggested that exon-associated localization changes may be sufficiently common to justify direct experimental testing, motivating the imaging-based screen described below.

### High-content imaging reveals prevalent localization differences between isoforms

Our bioinformatics analyses linked tissue-specific AS events to both protein interactome rewiring and localization diversity, especially for multimodular proteins. To determine whether these network-level effects translate into measurable changes in subcellular distribution, we next examined how AS influences protein localization at the single-cell level using high-content imaging. We focused on tissue-regulated AS events (both microexons and longer cassette exons) in multidomain proteins involved in endocytosis and cytoskeletal organization, which display a high degree of tissue-specific AS regulation and high prevalence for multidomain structures (Fig. 1i).

**Fig. 2:**
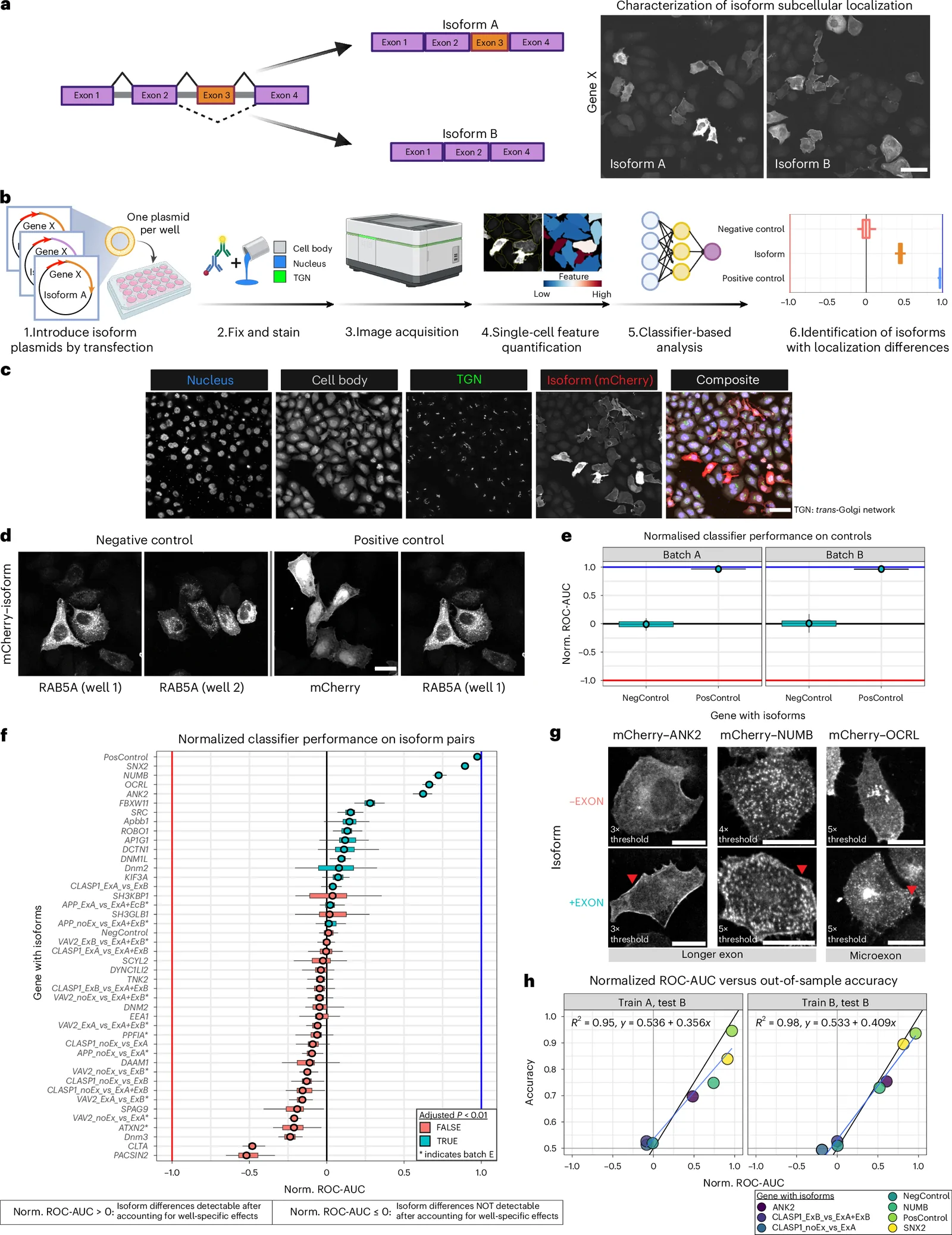
High-content imaging-based examination of a targeted protein isoform library reveals protein isoforms frequently differ in localization. **a**, Left, schematic of how exon skipping diversifies mRNAs and their coding potential. Right, example images of HeLa cells expressing fluorescent protein isoforms. Scale bar, 50 µm. **b**, Workflow of high-content imaging and classifier-based examination of protein isoforms (Methods). TGN, *trans*-Golgi network. **c**, Example of the four channels acquired for an FOV. The focus slice is shown after illumination correction. Scale bar, 50 µm. **d**, Representative images of cells expressing the mCherry constructs used as positive and negative controls. The negative control consists of two separate wells transfected with the same mCherry construct (mCherry–RAB5A). The positive control consists of two separate wells transfected with mCherry and mCherry–RAB5A. Scale bar, 20 µm. **e**, Box plot showing the ability of classifiers trained on mCherry features measured in cells expressing control plasmids to correctly assign the plasmid identifier (corresponding to well identifier), quantified as the normalized ROC-AUC. Cells came from three experimental repeats that were pooled for the classifier-based analysis with *n* cells ≥ 500 for each comparison after pooling. The experiment and analysis were performed twice (batch A, batch B), which were evaluated separately. The box plots illustrate the normalized ROC-AUCs from each of 50 iterations of cell sampling and classifier evaluation and the dot indicates the median normalized ROC-AUC (*n* = 50). Colored lines indicate perfect (blue), neutral (black) or worst (red) possible classifier performance, respectively. Box plots show the median (center line) and IQR (box ends) and whiskers show observations within 1.5× the IQR. **f**, Top, box plot showing the performance of classifiers trained on mCherry features of cells transfected with protein isoforms (or controls) making up isoform pairs, quantified as the normalized ROC-AUC. All isoform pairs from batches A–E are shown. Cells came from three experimental repeats from a given batch that were pooled for the classifier-based analysis. For isoform pairs present in multiple batches (batch A and B), cells expressing these were pooled before classifier-based analysis; hence, cells came from six experimental repeats. The shown controls also derive from pooling batch A and B (*n* cells ≥ 170 for each pair after pooling, with median *n* cells = 967; exact cell numbers listed in Supplementary Table 3). The box plots illustrate the normalized ROC-AUCs from each of 50 iterations of cell sampling and classifier evaluation and the dot indicates median (*n* = 50). Colors reflect whether the lower bound of a 99% bootstrap confidence interval included 0, after multiple-testing correction (FDR), with *P* values < 0.01 considered significant. *Batch E pairs. Gene names in all caps indicate human isoforms, whereas the other gene names indicate mouse isoforms. Bottom, interpretation of normalized ROC-AUC score. A description of box plots is provided in **e**. **g**, Representative images of HeLa cells expressing isoforms of mCherry–ANK2, mCherry–NUMB and mCherry–OCRL. The expression level (relative to the transfection threshold) is indicated in white text. Representative cells within each isoform pair were chosen to have comparable expression levels. Red arrowheads indicate the features accentuated in the +EXON isoform (ANK2, increased membrane association; NUMB, increased membrane association; OCRL, more granular appearance). The exon type (longer exon or microexon) is indicated by the gray bar. Scale bar, 15 µm. **h**, *XY* plot showing the correlation between the normalized ROC-AUC and the out-of-sample accuracy for the controls and five isoform pairs occurring in both batch A and B (*n* = 7). Colors indicate the pair identity. The normalized ROC-AUC for batch A is compared to the out-of-sample accuracy when trained on batch A and tested on B (vice versa for ROC-AUC for batch B). A linear regression was fitted to the data. After pooling cells across experimental repeats, ≥232 cells were present in both the training and the test set for a given comparison. Source data

We first generated a protein isoform library for imaging analysis by subcloning open reading frames (ORFs) encoding distinct isoforms into plasmids fused with mCherry. For each gene, we cloned at least two isoforms that differed by the inclusion or exclusion of an alternative exon. The final library comprised 68 isoforms across 32 genes, yielding 44 possible within-gene isoform comparisons (Supplementary Table 2), of which only ~16% had previously been examined for localization differences^36–42^. Our aim was to determine whether isoforms from the same gene (referred to as isoform pairs) showed distinguishable subcellular localization behaviors when examined under comparable conditions (Fig. 2b) on the basis of quantitative features of the mCherry fluorescence signal, using identical and clearly distinct constructs as negative and positive controls.

**Extended Data Fig. 3:**
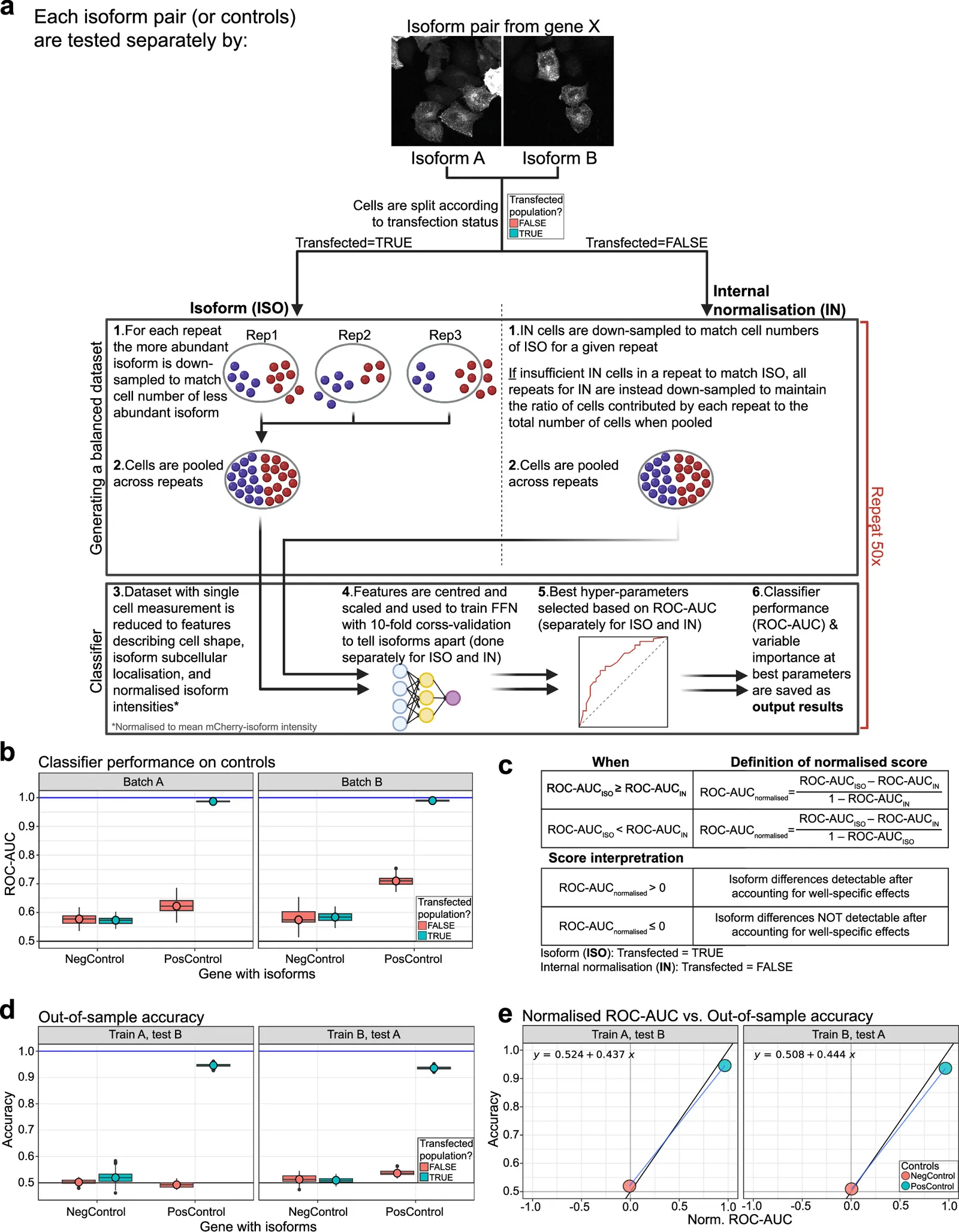
Classifier-based analysis of single cell measurements was used to detect isoform differences, which was validated on well-defined controls. **a**, Overview of classifier-based analysis. For each pairwise comparison, transfected (ISO) and non-transfected internal normalisation (IN) cells were analysed separately. Within each experimental repeat, the more abundant isoform was down-sampled to match the less abundant isoform before pooling across replicates, ensuring equal representation of each isoform while minimising replicate-specific effects. IN cells were sampled to match the ISO dataset. A feedforward neural network was trained using image-derived features describing cell morphology, mCherry localisation and normalised intensities. Classifier performance and feature importance were estimated using repeated down-sampling and 10-fold cross-validation (50 iterations). See Methods for details. **b**, Classifier performance for mCherry image features from control plasmids, quantified by ROC–AUC. Classifiers were trained separately on ISO and IN cells to assess contributions of well-specific effects. Data are pooled from three experimental repeats per batch (A and B; analysed separately), with ≥500 cells per comparison after pooling. Boxplots show the distribution across 50 down-sampling/classification iterations (median, IQR and 1.5×IQR whiskers). Blue and black lines indicate perfect and worst possible performance, respectively. **c**, Definition and interpretation of the normalised ROC-AUC score, where the internal normalisation (IN) classifier (using non-transfected cells, Transfected = FALSE) is used to normalise the isoform classifier (consisting of transfected cells, Transfected = TRUE). **d**, Cross-batch validation of classifiers trained on control plasmids. Classifiers trained on batch A were tested on batch B and vice versa. Test batches were down-sampled to equal numbers of cells per plasmid, giving an expected accuracy range of 0.5–1.0. Boxplots summarise 20 training/testing iterations (median, IQR and 1.5×IQR whiskers). ≥500 cells per condition were included in both training and test sets. See **b** for boxplot description. **e**, XY plot showing the correlation between the normalised ROC-AUC (norm. ROC-AUC) and the out-of-sample accuracy for the two controls. The normalised ROC-AUC for batch A is compared to the out-of-sample accuracy when trained on batch A and tested on B (vice versa for ROC-AUC for batch B). A linear regression was fitted to the data. Source data

Isoform-encoding plasmids were transiently transfected into HeLa cells (Fig. 2b), which were then fixed, stained and imaged by automated confocal microscopy across four channels (DAPI, NHS ester, anti-GOLGA1 and mCherry) (Fig. 2c). Single cells were segmented and ~200 features describing the mCherry signal were extracted to identify transfected cells and quantify fusion localization (Supplementary Fig. 1a). The isoform expression levels were generally comparable within isoform pairs (Supplementary Fig. 1b) and any residual imbalance was further reduced by excluding the brightest cells from the more highly expressed isoform (Supplementary Fig. 1c). We then used a classifier-based approach to examine each isoforms pair for differentially localizing isoforms on the basis of the mCherry signal features (Fig. 2b). Here, cells expressing either isoform within an isoform pair were pooled, downsampled to equal numbers of cells per isoform and used to train a classifier with tenfold cross-validation to assign isoform identity (Extended Data Fig. 3a and Methods). Classifier performance was measured as the mean area under the receiver operating characteristic curve (ROC-AUC; 0.5 = random, 1.0 = perfect separation), which we used to quantify the degree of difference between isoforms. Internal normalization classifiers were trained on nontransfected cells from the same wells as the corresponding isoform pair to account for well-specific batch effects. The classifiers also assigned feature importance scores, which identified the most discriminating signal characteristics between the isoforms.

To validate the approach, we assessed its performance on negative controls (duplicate mCherry–RAB5A wells) and positive controls (mCherry–RAB5A versus mCherry) (Fig. 2d). As expected, classifiers trained on transfected cells matched the performance of their internal normalization classifiers for the negative controls but showed near-perfect performance for positive controls (ROC-AUC ≈ 0.98 in two independent experimental batches; Extended Data Fig. 3b). To correct for well-specific effects, we used the internal normalization classifiers to define a normalized ROC-AUC metric, symmetrical around zero, where values > 0 indicated that isoform differences were detectable after accounting for well-specific effects (Extended Data Fig. 3c). Positive and negative controls scored near 1 and 0, respectively, across batches (Fig. 2e). We confirmed the reproducibility of the approach using out-of-sample testing (train on one batch, test on the other); out-of-sample classifiers achieved ~0.92–0.94 accuracy for positives and ~0.5 for negatives (Extended Data Fig. 3d) and scores correlated strongly (*r* ≈ 1.0) with within-batch normalized ROC-AUC (Extended Data Fig. 3e). Together, these results validated the classifier-based framework and its normalized ROC-AUC metric as a sensitive and reliable measure of construct-dependent localization differences.

**Extended Data Fig. 4:**
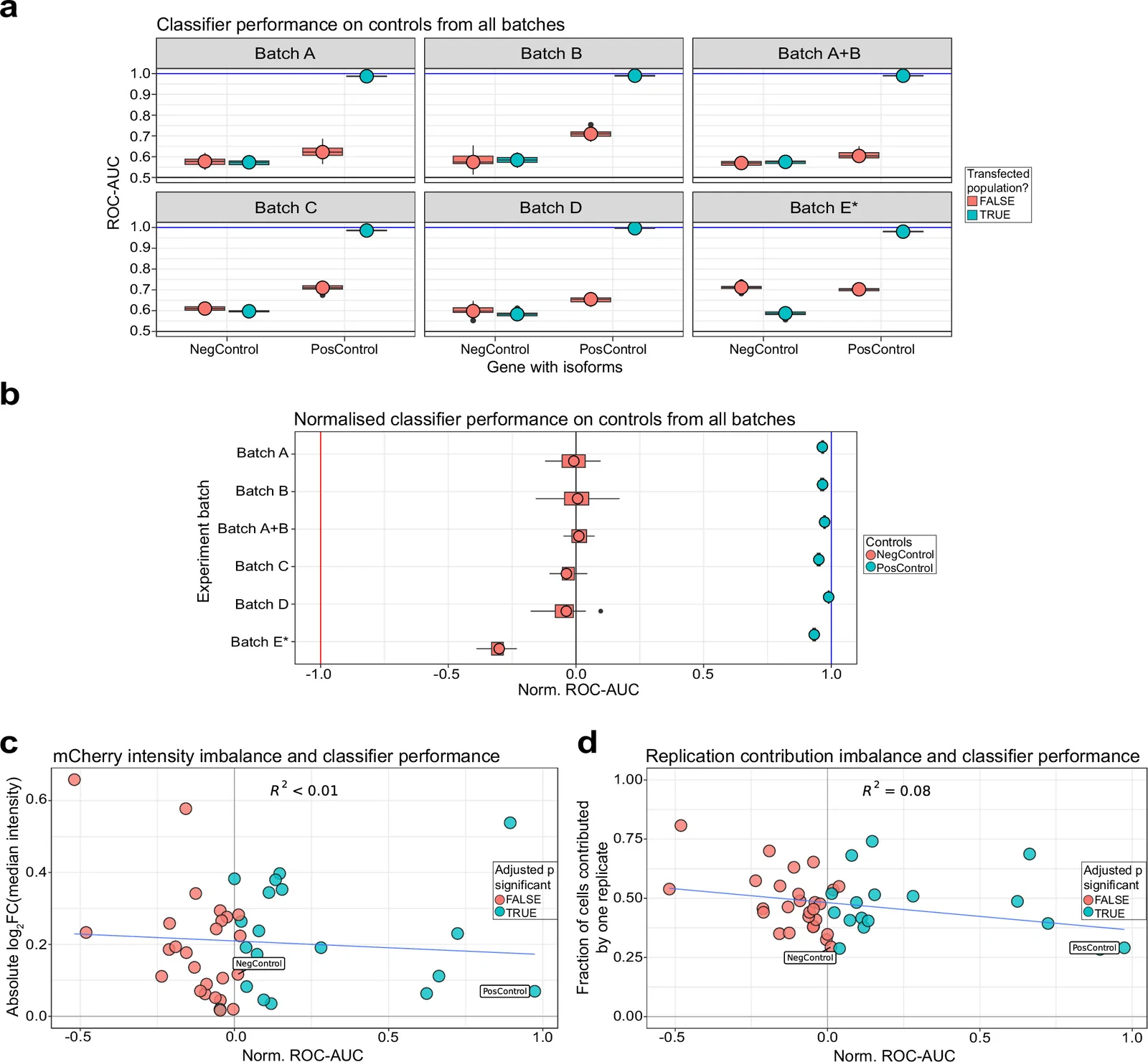
Performance on controls is largely consistent across batches, and classifier performance across library is not driven by expression level imbalance between isoforms within pairs nor by imbalanced contributions of cells from single replicates. **a**, Boxplots showing the ability of classifiers trained on mCherry features measured in cells expressing control plasmids to correctly assign the plasmid ID (which corresponds to well ID), quantified as ROC-AUC. The evaluation was performed separately on all experimental batches as well as the pooled batch A *+* B. Each batch consisted of three experimental repeats. n cells ≥ 500 for each comparison after pooling. The boxplots illustrate the ROC-AUCs from each of 50 iterations of cell sampling and classifier evaluation, with dot indicating median ROC-AUC (n *=* 50), box edges marking the interquartile range (IQR), and whiskers show observations within 1.5× IQR. Classifier-based analysis was performed separately on transfected (isoform) and non-transfected (internal normalisation) cells from the same wells, indicated by colour. Coloured lines indicate perfect (blue) and worst (black) possible performance, respectively. * indicates Batch E. **b**, Same as **a**, but the boxplots display classifier performance quantified as normalised ROC-AUC (norm. ROC-AUC). Coloured lines indicate perfect (blue), and neutral (black) performance, respectively. **c**, XY plot of normalised classifier performance (norm. ROC-AUC) against expression imbalance between isoforms within the isoform pair (calculated as absolute log_2_(median intensity_+EXON/median_intensity_-EXON) for all cells used for classifier assessment). Each dot represents an isoform pair (n = 42). Dot colour indicates whether isoforms were found to be differentially localising or not. Positive and negative controls are labelled. A linear regression line (x ~ y) is fitted to the data to describe the correlation. **d**, XY plot of normalised classifier performance (norm. ROC-AUC) against how large a fraction of cells originated from one replicate. Each dot represents an isoform pair (n = 42). Dot colour indicates whether isoforms were found to be differentially localising or not. Positive and negative controls are labelled. A linear regression line (x ~ y) is fitted to the data to describe the correlation. Source data

**Extended Data Fig. 5:**
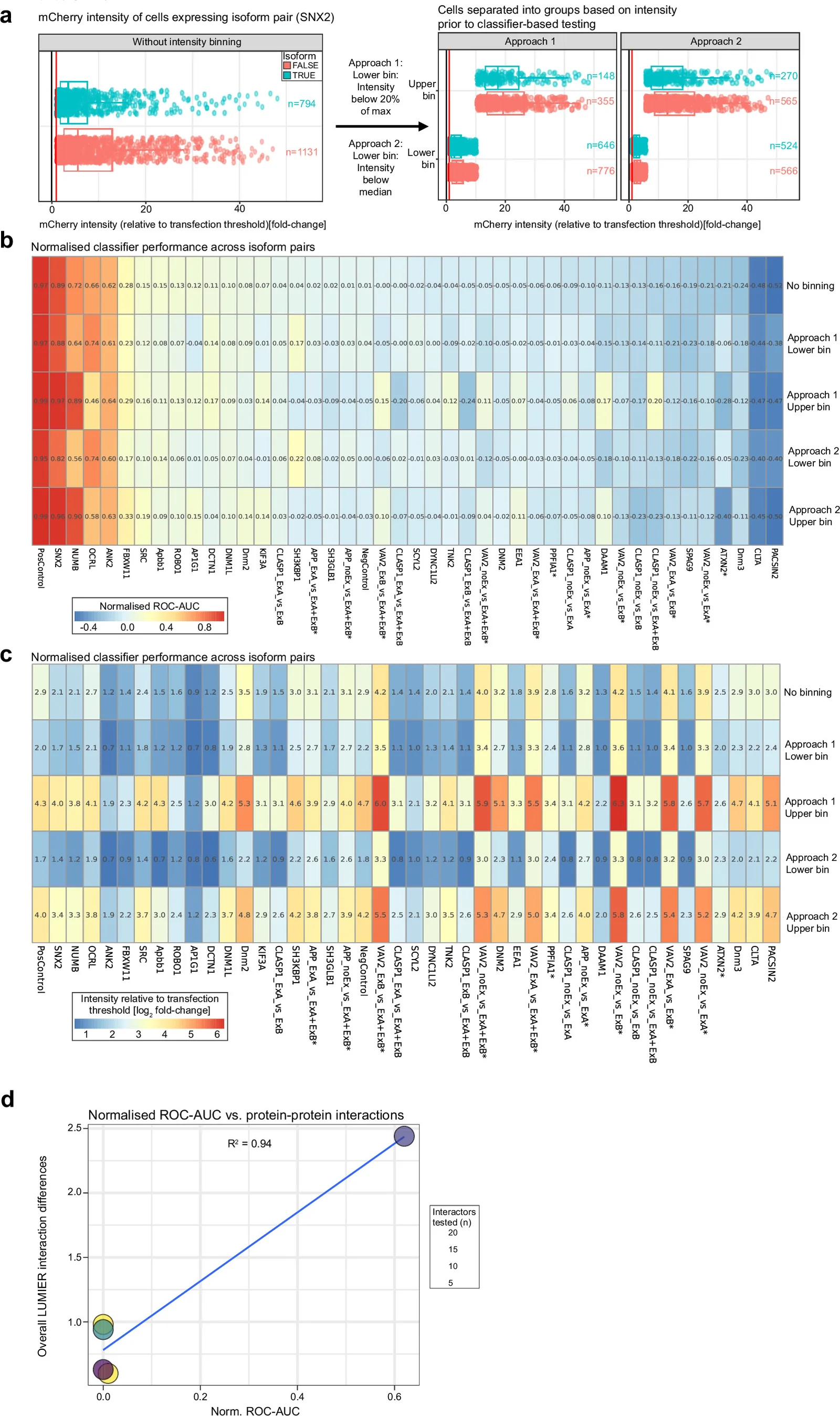
Separating cells in isoform pairs into high and low expression groups before classifier-based analysis yields comparable results to no separation. **a**, Boxplots of cell mCherry intensity (relative to the transfeciton threshold) of cells expressing mCherry-SNX2 isoforms with individual cells plotted as dots, before (**left**) and after (**right**) separating cells into low and high expression bins. Two binning approaches were used in parallel (Methods). Note, the binning thresholds were calculated for both isoforms, and the lower threshold was then applied across both isoforms. Colours indicate isoform identity. Red line indicates transfection threshold. Boxplots show median (centreline), and interquartile range (IQR) (box ends), and whiskers show observations within 1.5× IQR. **b**, Heatmap showing normalised classifier performance (norm. ROC-AUC) for all isoform pairs when using the full isoform dataset (No binning) or lower or higher intensity cells, separated via approach 1 or 2 outlined, as outlined in **a**. **c**, Heatmap showing median mCherry intensity relative to transfection threshold (log_2_ fold-change), for cells expressing isoforms from a given gene, either using all cells (No binning) or after cells were separated into lower and higher intensity bins. **d**, XY plot examining the correlation between the normalised ROC-AUC (norm. ROC-AUC) and overall LUMIER interaction difference (a PPI assay) (Methods) for isoform pairs where ≥ 3 interactors have been found to interact with at least one of the isoforms in a pair, n = 5 isoform pairs. A linear regression was fitted to the data, and the correlation was tested using a correlation test (cor(method = ‘Pearson’)). Source data

We next applied the high-content imaging and classifier-based framework to our isoform library. Isoforms were processed in five transfection, staining and imaging batches (A–E; three replicates per batch), with overlapping isoforms between A and B being pooled to increase cell numbers without negative affecting performance loss, as assessed by pooling the controls (Supplementary Fig. 2). Each batch included positive and negative controls to assess consistency, which had expected normalized ROC-AUCs near 1 and 0, respectively, except batch E, where a brighter NHS ester staining led to an anomalously low score for the negative control (~–0.3; Extended Data Fig. 4a,b). Despite this, we considered all batches suitable for analysis as E’s positive control performed as expected. In total, we successfully analyzed ~94% (64/68) of isoforms across 30 genes, yielding 42 pairwise comparisons (Fig. 2f and Supplementary Table 3). Normalized ROC-AUC values ranged from –0.5 to ~1.0, with most being between –0.2 and 0.2, indicating both distinct and similar localizations among isoform pairs. Significant differences (bootstrap 99% confidence interval > 0) were detected for the positive but not the negative control and in 38% (16/42) of pairs (Fig. 2f and Supplementary Fig. 3), including 71% (5/7) of pairs with previously described localization differences (Supplementary Table 3), with these five pairs displaying localization differences consistent with the previous reports^36–41^. We observed similar rates of localization differences for microexons (~38%, 10/26) and longer exons (~38%, 6/16), with both classes represented among top performing classifiers, such as ANK2, NUMB and OCRL (Fig. 2g). Importantly, the normalized performance was independent of overall expression level differences between isoforms within pairs (Extended Data Fig. 4c; *P* = 0.76, correlation test) and the fraction of total cells contributed by a single replicate (Extended Data Fig. 4d; *P* = 0.10, correlation test). Moreover, classifier performances were highly consistent when repeating the analysis after separating cells within isoform pairs into groups with low and high expression (Extended Data Fig. 5a,b), suggesting that the isoform differences are present across all measured expression levels, with the low-intensity groups being only slightly above the mCherry detection limit (transfection threshold) for many isoform pairs (Extended Data Fig. 5c). These analyses all support that the detected differences have biological rather than technical origins.

To assess reproducibility, we performed out-of-sample testing on isoform pairs recurring in batches A and B. Out-of-sample accuracy was significantly correlated with classifier normalized ROC-AUC for both batches (Fig. 2h; *P* = 0.01 and 0.003, slopes ≈ 0.36–0.41), indicating reproducible discrimination of isoform pairs across independently acquired experiments. Moreover, this pattern is consistent with classifier performance reflecting construct-associated differences rather than batch-specific or well-specific effects. Together, our imaging-based examination of isoforms, therefore, support evidence from our in silico analysis that AS frequently alters subcellular localization of multidomain proteins and does so through both microexons and longer exons in the examined isoforms, when expressed at comparable levels.

### Comparison of localization metric to independent interaction data suggests association between localization and PPIs

To assess whether the detected localization differences were supported by independent data, we examined whether differentially localizing isoform pairs also showed interaction differences in an independent PPI dataset. We reasoned that, if isoforms differ in localization, they may also differ in PPIs, because an altered subcellular context supposedly affects the pool of accessible interaction partners. While isoform-level data remain sparse, including isoform-related interaction data, we could examine the association for five isoform pairs by integrating data from LUMIER-based PPI screens^12^ (Supplementary Tables 4 and 5). We observed that isoform pairs with greater localization divergence also tended to exhibit greater interaction divergence (Extended Data Fig. 5d; *R*^2^ = 0.94, *P* = 0.0058). This cross-assay concordance is consistent with the idea that isoform-dependent localization differences are accompanied by changes in interaction behavior. Therefore, although limited in scope, this analysis supports a broader association between localization divergence and interaction divergence observed in the imaging screen and supports that the measured localization differences do occur for the select pairs present in both analyses.

### Microexon inclusion displaces charged residues within secondary-structure elements

Our imaging-based screen revealed that microexons, despite encoding only a few residues, alter protein localization to a similar extent to longer cassette exons. This is notable because AS typically modulates localization and interaction networks through well-characterized mechanisms such as domain inclusion^43–45^ or the gain or loss of short linear motifs^17,46^, which adds or removes interaction interfaces. By contrast, microexons are short insertions that often occur in structured regions and adjacent to existing interfaces^29^, raising the question of how such small insertions, which are unlikely to destroy or generate entire interaction surfaces, can exert consistent functional effects. One possibility is that microexons act through subtle structural mechanisms by adjusting the local geometry or the positioning of key residues within preexisting folds and interfaces. To explore this, we examined whether microexons display properties that allow them to preserve or modulate protein secondary structure, which may be one way they can alter the architecture and interfaces of structured regions.

**Fig. 3:**
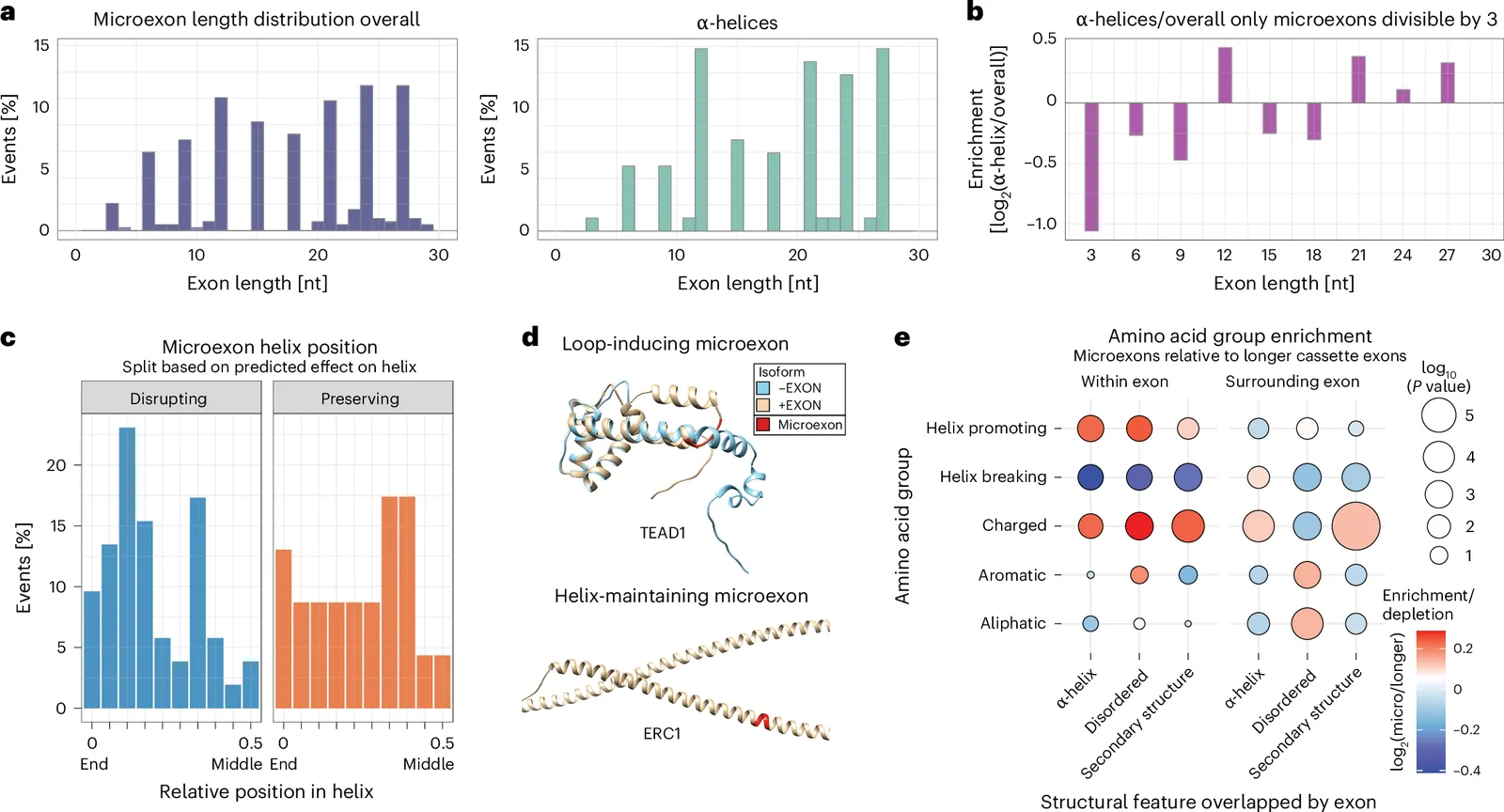
Microexons potentially affect α-helical structures in two common ways. **a**, Relative frequency histogram of microexon nucleotide lengths for tissue-specific microexons (<30 nt) (Methods). Left, overall. Right, microexons predicted to overlap α-helices (*n* microexons = 353, *n* microexons in α-helices = 82). **b**, Bar plot showing the enrichment and depletion of alternatively spliced microexons with specific lengths in α-helices, calculated as log_2_((Freq. α-helix_nt)/(Freq. overall_nt)). Only microexon lengths divisible by 3 are included. **c**, Relative frequency histogram of tissue-specific microexon positions within predicted α-helices, quantified as the relative distance of the point where the microexon is inserted to the nearest α-helix end (Methods). Microexons are grouped according to whether their length is predicted to preserve (12 nt, 21 nt; preserving) or disrupt (other lengths; disrupting) the helical structure. **d**, AlphaFold structures of TEAD1 and ERC1 isoforms, showcasing microexons predicted to induce or extend loops in α-helical structures and microexons predicted to preserve the helix. Microexons are colored red. For TEAD1, isoforms with and without the microexon are shown in separate colors (blue and gold) and overlaid. **e**, Bubble plot showing amino acid enrichment within exons (exon) and flanking regions (surround), for tissue-specific microexons overlapping predicted α-helices, disordered regions and secondary-structure elements (α-helices, β-strands and loops), compared to longer cassette exons (≥30nt) with a similar inclusion pattern. Only exons divisible by three were included. Amino acids were grouped according to properties in a non-mutually exclusive manner (helix forming, L, I, F, E, Y, W and M; helix breaking, P and G; charged, E, D, K and R; aromatic, F, Y W and H; aliphatic, L, I and V). Colors indicate the degree of enrichment (calculated as log_2_((Freq. microexon)/(Freq. longer exon)). Size indicates statistical significance calculated using the Fisher exact test. Flanking residues were the first 30 residues upstream and 30 residues downstream of the microexon (*n* microexons in α-helices = 77). Source data

As the top microexon hits in our imaging screen (SNX2 and OCRL) were located within or adjacent to α-helices, we first examined whether microexons in general show a bias toward preserving helical structural architecture. We analyzed the nucleotide length distribution of microexons in coding regions, comparing microexons in general to those predicted to occur within α-helices (Fig. 3a). We observed that microexons of 12 nt and 21 nt, encoding four and seven residues, respectively, corresponding to one and two helical turns, were enriched in predicted helices (Fig. 3b). This suggests that short microexons often preserve helical structure. However, the enrichment of slightly longer 24-nt and 27-nt microexons, which would be expected to affect helical structure differently, suggests that this bias is largely restricted to shorter insertions. Therefore, we next examined the position of microexons within predicted helices (Fig. 3c). Interestingly, helix-preserving microexons (12 nt and 21 nt) were rather evenly distributed along helices, whereas those with potentially disruptive lengths were enriched near helix ends, where they may extend or induce loops. This pattern was exemplified by models from the AlphaFold Protein Structure Database^47^ of ERC1 and TEAD1, where microexon inclusion is predicted to either maintain helical geometry or subtly change it by forming or extending loops, respectively (Fig. 3d). To explore the biochemical consequences of these occurrences, we compared the amino acid compositions of microexons and their flanking residues to those of longer (>30 nt) tissue-specific cassette exons with similar regulation patterns (Fig. 3e). Microexons were enriched in helix-promoting residues and charged residues, regardless of whether they occurred in predicted α-helices or disordered regions, although this trend weakened when β-strands and loops were considered. Moreover, residues flanking microexons within structured regions contained more charged residues than those surrounding longer exons, a difference not observed for disordered regions.

Together, these observations suggest that microexons may affect basic α-helical architecture in at least two ways: by preserving and extending helices or by disrupting the helix to some extent, including forming or extending loops. When they occur in α-helices and other secondary structures (β-strands and loops), they may displace the flanking residues, notably charged residues, which potentially serves as a way where microexons can fine-tune the properties of preexisting interfaces and possibly modulate intermolecular interactions.

### Insertion of the SNX2 microexon, not its composition, is a key determinant of isoform-related localization and interaction changes

**Fig. 4:**
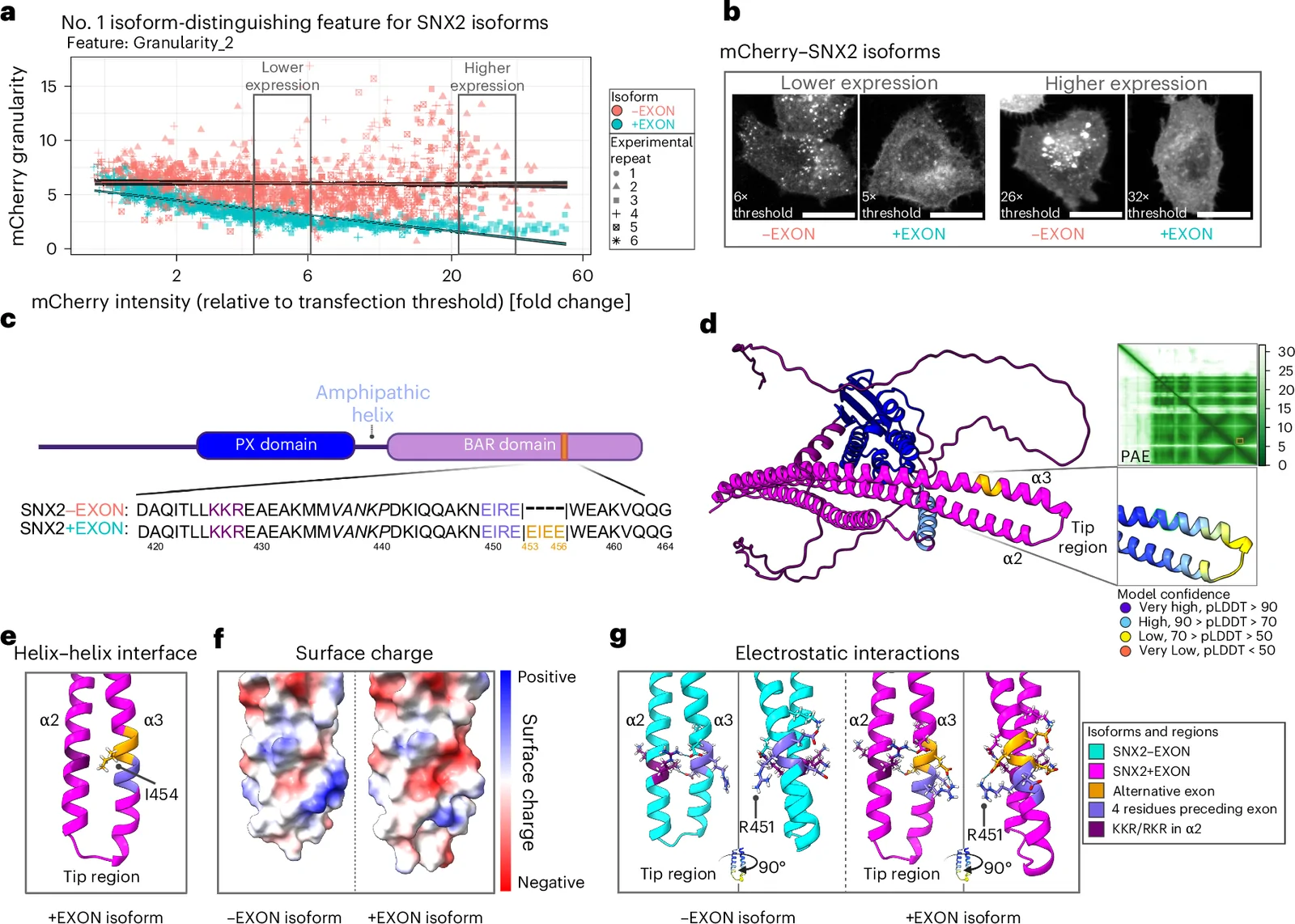
SNX2’s microexon alter localization granularity while maintaining SNX2’s BAR domain and extending an α-helix in the BAR domain. **a**, *XY* plot of top isoform-distinguishing feature for the SNX2 isoforms (mCherry granularity) against cell mean mCherry intensity (relative to transfection threshold) (fold change). Each data point corresponds to a cell, with color and shape indicating SNX2 isoform identity and experimental repeat identifier, respectively. Cells come from *n* = 6 experimental repeats, with pooled *n* cells > 1,300. Separate linear regression lines were fitted to cells expressing SNX2+EXON and SNX2−EXON. Two rectangles (lower expression, higher expression) indicate where the representative cells (Fig. 4b) fall within the overall population. **b**, Representative images of HeLa cells expressing mCherry–SNX2 isoforms. Cells with two different expression levels (lower, higher) are shown with the expression level (relative to the transfection threshold) indicated in white text. Representative cells were chosen to have comparable expression levels. Images derive from one of six independent experiments with similar outcome. Scale bar, 15 µm. **c**, Primary structure of human SNX2 with structured domains indicated as ovals and disordered regions as lines. The microexon (orange) is inserted into SNX2’s BAR domain (purple) and the exon’s residues (orange), as well as the residues surrounding the exon, are indicated (residues 419–464). The four residues preceding the exon are lavender and the KKR motif (426–428) is dark purple. Residue numbers before the exon corresponds to UniProt O60749-1. Residues after the exon have a position number that is +4 that of UniProt O60749-1. **d**, AlphaFold2 structure of human SNX2+EX. The BAR domain is colored magenta, the amphipathic helix is light blue and the PX domain is dark blue. The microexon is colored orange and inserts as an additional helical turn in the α3 helix of the BAR domain. The α2 helix and tip region connecting α2 and α3 are indicated. The predicted aligned error (PAE) for the model (region with exon marked by orange square) and the predicted local distance difference test (pLDDT) for the BAR region with the microexon are shown (exon marked with green outline). **e**, AlphaFold2 model showing the position of I454’s side chain, encoded by SNX2’s microexon, for the human SNX2+EX isoform in the BAR region containing residues 419–464. The BAR domain is viewed from the side that faces lipid membranes. The microexon is orange, the four residues preceding the exon are lavender and the KKR motif (426–428) is dark purple. **f**, Surface charge of AlphaFold2 models of human SNX2 isoforms in the BAR region containing residues 419–464. The BAR domain is viewed from the side that faces lipid membranes. **g**, Predicted electrostatic interactions between residues in the AlphaFold2 models of human SNX2 isoforms in the BAR region containing residues 419–464. Residues engaging in electrostatic interactions (salt bridges) are shown, along with residues that form electrostatic interactions in one isoform but not the other (R451, E452). The microexon, the four preceding residues and the KKR motif are indicated. Left, the BAR domain from the side that faces lipid membranes. Source data

**Extended Data Fig. 6:**
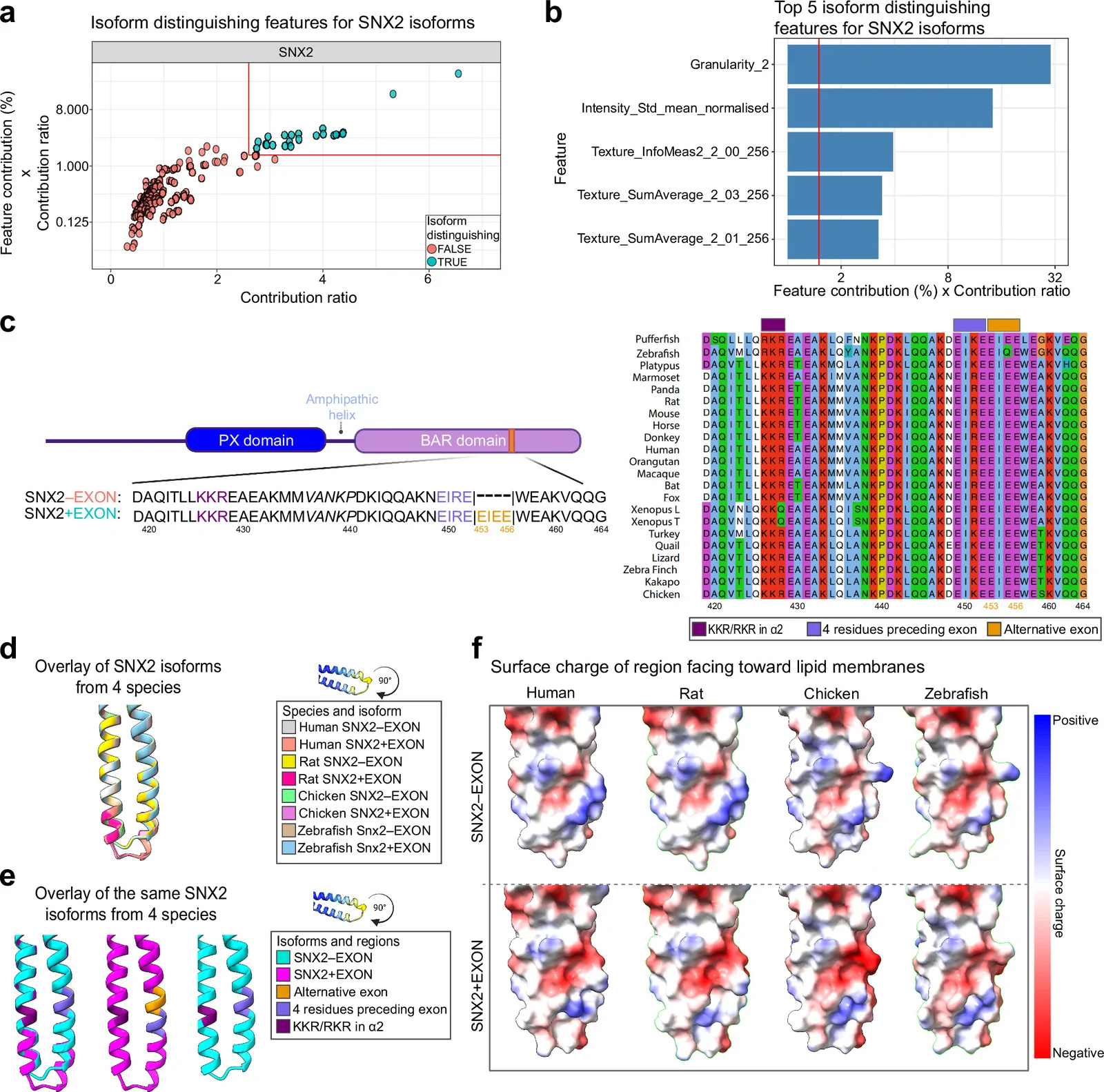
Multiple features capture the localisation differences related SNX2’s microexon, and SNX2’s microexon is highly conserved and is predicted to affect structure in a similar manner across vertebrates. **a**, XY plot of Contribution ratio and Contribution ratio multiplied with Feature contribution (%) for the ISO classifier for each feature for the SNX2 isoform pair, used to identify Isoform Distinguishing features (Methods). Of ~200 features, ~20 was identified as Isoform distinguishing. **b**, Barplot of the top 5 Isoform distinguishing features according to their Feature contribution (%) multiplied with Contribution ratio. The threshold value defining Isoform Distinguishing features is indicated as a red line. **c**, Multiple sequence alignment of SNX2 region corresponding to human SNX2 residues 419-464 for 22 vertebrate species. The location of the microexon (orange), the 4 residues preceding the microexon (lavender), and the KKR motif (dark purple) are indicated by coloured rectangles. The individual positions are coloured according to the residue properties that is conserved across the aligned sequences (default clustal colouring from JalView). **d**, Overlay of AlphaFold2 models of the SNX2 BAR region containing residues 419-464 (human SNX2 residue numbering) of both SNX2 isoforms from four species (human, rat, chicken, zebrafish). The BAR domain is viewed from the side that faces lipid membranes. Each species-isoform combination has a distinct colour. **e**, Overlays of AlphaFold2 models of the SNX2 BAR region containing residues 419-464 (human SNX2 residue numbering) from four species (human, rat, chicken, zebrafish), separated according to isoform identity ( ± EXON). Colours reflect isoform (-EX cyan, +EX magenta), microexon (orange), the four residues preceding the microexon (lavender), and the KKR motif (dark purple), rather than species-isoform combination. **f**, Surface charge of AlphaFold2 models of SNX2 isoforms from the four species (human, rat, chicken, zebrafish), in the BAR region containing residues 419-464 (human SNX2 residue numbering). The BAR domain is viewed from the side that faces lipid membranes. Source data

To test the predictions from this bioinformatic analysis for α-helical microexons, we next examined the two isoforms of sorting nexin 2 (SNX2), which were the top hits from our subcellular localization screen and differed in an α-helical microexon. SNX2 is a multidomain protein that regulates retrograde transport and recycling of internalized membrane proteins^48^ and the isoforms were previously uncharacterized. Our imaging data showed that microexon inclusion markedly altered SNX2 localization with approximately 20 features being isoform distinguishing (Extended Data Fig. 6a, Supplementary Table 6 and Methods), the most important being granularity (Extended Data Fig. 6b). The granularity feature captured the more punctuated, vesicular localization of SNX2−EX, which formed aggregates at higher expression, previously described as swollen endosomes for GFP-SNX2−EX^49^, whereas SNX2+EX displayed a more diffuse cytoplasmic distribution at all expression levels (Fig. 4a,b). The pronounced localization differences between the SNX2 isoforms highlighted them as a model to examine how an α-helical microexon can tune domain geometry and residue positioning with localization consequences.

SNX2’s microexon encodes four residues (EIEE) that insert into SNX2’s BAR domain (Fig. 4c)—a coiled-coil structure that mediates lipid binding, dimerization and higher-order oligomerization^50^. To understand how this altered SNX2 function, we first modeled both isoforms using AlphaFold2 (ref. ^51^). This suggested that the microexon inserts into the BAR domain as a single additional helical turn in the α3 helix (Fig. 4d) and presumably maintains the domain fold with the inserted I454 forming helix–helix interactions (Fig. 4e), which are critical for coiled-coil stability^52^. The insertion made the surface charge more negative (Fig. 4f) and was predicted to alter electrostatic interactions for a few, immediately flanking residues (R451, E452) (Fig. 4g), through a combination of the exon’s three glutamates and repositioning existing residues.

These observations were supported by multiple-sequence alignment of the microexon-containing region of SNX2’s BAR across vertebrates, which showed a high degree of conservation of both the microexon’s residues and the flanking residues (Extended Data Fig. 6c) and by AlphaFold2 models of both isoforms in select species (rat, chicken, zebrafish), which closely resembled the human isoforms (Extended Data Fig. 6d–f). Collectively, this indicated that the microexon alters SNX2’s BAR domain interface by changing surface charge and electrostatic interactions and subtly repositioning flanking charged residues, rather than changing the overall fold; these effects may cause the localization differences.

**Fig. 5:**
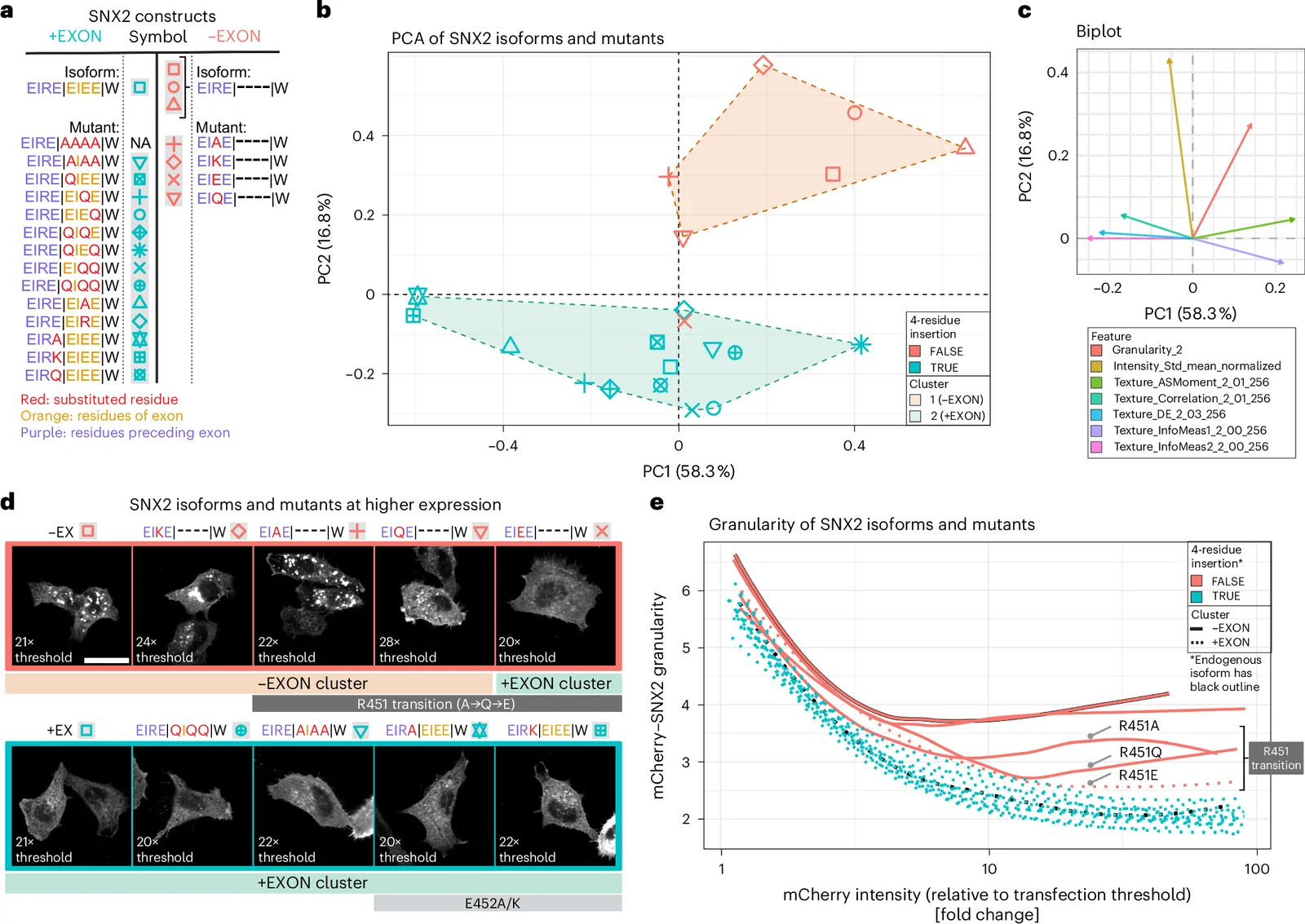
SNX2’s isoform-related subcellular localization is altered by insertion of the microexon, not by the composition of its residues. **a**, List of SNX2 mutant constructs examined by high-content imaging. Residue colors correspond to the colors in Fig. 4c, where purple indicates the four residues preceding the microexon and orange indicates the microexon. Substituted residues are indicated in red. Each construct has a symbol that indicates their position in the PCA, with symbol color representing the presence or absence of the four-residue insertion (microexon). Note, three technical replicates for SNX2−EX were included (indicated by different shapes). **b**, PCA of transfected cells positively expressing SNX2 isoforms and SNX2 mutants using the SNX2 isoform-distinguishing features as input. The plot is an *XY* plot of the centroid scores for PC1 and PC2 for each construct (that is, the mean PC1 and mean PC2 calculated for all cells expressing the given construct). Each construct is represented by a symbol (as Fig. 5a), colored according to the presence or absence of four-residue insertion (microexon) (true = cyan, false = red). The constructs are clustered into two groups on the basis of their centroid scores of PC1 to PC5. The two clusters are outlined by dashed polygons and have separate colors. The cells used for the centroid scores were pooled across three experimental replicates (*n* = 3) with ≥168 cells expressing each construct (cell numbers for each construct shown in Extended Data Fig. 7b). **c**, PCA biplot showing the loading vectors of the isoform-distinguishing features on PC1 and PC2 for the transfected cells (relates to **b**). Arrow color reflects the specific feature. Arrow direction and length indicate the relative contribution of each feature to the two PCs. Features with longer arrows indicate larger contributions. Features pointing in the same or opposite direction are positively and negatively correlated, respectively. ASmoment, angular second moment; DE, difference entropy. **d**, Representative images of HeLa cells expressing mCherry–SNX2 isoforms and mCherry–SNX2 mutants within the –EXON and +EXON clusters identified in Fig. 4b. The expression level (relative to the transfection threshold) for the brightest cell in each image is indicated in white text. Representative cells were chosen to have comparable expression levels. The PCA symbol and cluster identity for each construct are indicated. Molecular features that stand out from the clustering and granularity behavior (R451 and E452 status) are also indicated. Scale bar, 25 µm. **e**, Line plot of mCherry Granularity (*y* axis) against cell mean mCherry intensity (relative to the transfection threshold; fold change) for transfected cells expressing the mCherry–SNX2 isoforms and all SNX2 mutants. Each line represents a specific construct, with line color reflecting whether the four residues are inserted (microexon status) (true = cyan, false = red) and line type indicating cluster identity. The lines for the endogenous isoforms are outlined by shade and the SNX2−EX mutants with R451 substitutions that cause a gradual change in granularity are indicated. The cells used to form the lines (method = ‘loess’) are pooled across three experimental repeats (*n* = 3), with ≥168 cells expressing each construct (cell numbers for each construct shown in Extended Data Fig. 7b). Source data

**Extended Data Fig. 7:**
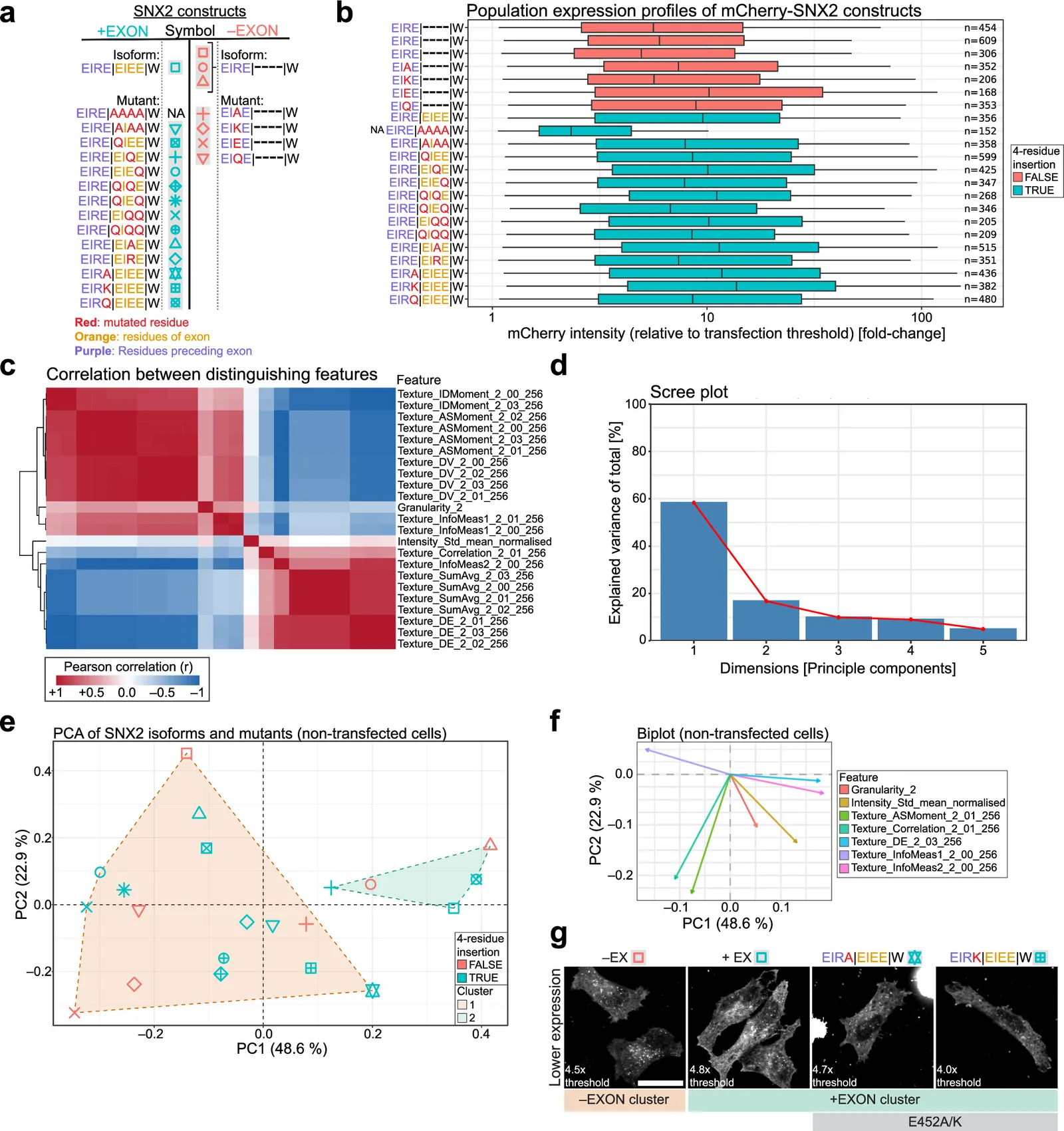
All but one mCherry-SNX2 construct express at comparable levels, and PCA clustering is driven by construct-dependent effects. **a**, List of SNX2 isoform and mutant constructs examined via high-content imaging. Same as Fig. 5a. **b**, Boxplots showing cell mCherry mean intensity (relative to transfection threshold) [fold-change] for cells expressing the various mCherry-SNX2 isoforms and mutants. Cells were pooled from three experimental repeats (N = 3). The number of cells after pooling is indicated for each construct (n). See 2e for boxplot description. **c**, Pairwise Pearson correlation heatmap between all SNX2 isoform distinguishing features calculated using all cells expressing the SNX2 isoforms and mutants. The features were hierarchically clustered based on their correlation (1-r) with the relationship between them indicated by the dendrogram on the left, revealing several groups of co-varying features. **d**, Scree plot summarising the proportion of variance explained by the first five principal component from the PCA of transfected cells expressing SNX2 isoforms and mutants, relates to Fig. 5b. **e**, Principal component analysis (PCA) of non-transfected cells in wells transfected with SNX2 isoforms or mutants using the isoform distinguishing features. Points represent construct centroid scores for PC1-PC2, coloured according to the exon status ( + EXON = cyan, -EXON = red) and grouped according to clustering based on PC1-5. The two clusters are outlined by dashed polygons and have separate colours. **f**, PCA biplot showing the contribution of Isoform distinguishing features to PC1 and PC2 for the non-transfected cells (relates to **e**). Arrow colour reflects the specific feature. Arrow direction and length indicate the relative contribution of each feature to the two PCs. Features with longer arrows indicate larger contributions. Features pointing in the same or opposite direction are positively and negatively correlated, respectively. **g**, Representative images of HeLa cells expressing mCherry-SNX2 isoforms and the mCherry-SNX2 E452A/K mutants. Cells expressing the isoforms at lower and higher expression levels are shown. The expression level (relative to the transfection threshold) for the brightest cell in each image is indicated in white text. Representative cells were chosen to have comparable expression levels. The PCA symbol and cluster identity for each construct is indicated. Scale bar: 25 µm. IDmoment: Inverse Difference Moment, ASmoment: Angular Second Moment, DV: Difference Variance, DE: Difference Entropy. Source data

To test these structural predictions, we generated 18 mCherry–SNX2 mutants (Fig. 5a), targeting the microexon’s residues and the flanking charged residues with predicted electrostatic interaction changes (R451, E452), to explore the role of charge and electrostatic interactions in localization. We retained I454 in all but one mutant because of its putative structural role. We transfected the mCherry–SNX2 constructs into HeLa cells and imaged them by automated confocal microscopy. All mutants, except the I454 mutant, expressed at comparable levels to the mCherry–SNX2 isoforms (Extended Data Fig. 7a,b) and were retained for subsequent localization analysis. Principal component analysis (PCA) of SNX2’s isoform-distinguishing features after removal of highly correlated features (Extended Data Fig. 7c and Methods) grouped constructs into two clusters using PC1 to PC5 scores, which captured 98.9% of variance (Extended Data Fig. 7d). The clusters contained the endogenous SNX2−EX (−EX cluster) and SNX2+EX (+EX cluster) isoforms, respectively, and were predominantly separated along PC2 (Fig. 5b,c). This pattern was absent in the nontransfected controls (Extended Data Fig. 7e,f), indicating that the clustering reflected construct-specific effects rather than batch effects. On the basis of this clustering, we considered that PC2 predominantly captured the differences between the endogenous isoforms. Accordingly, the two clusters separated almost perfectly along the granularity feature vector (Fig. 5c), the most important feature for SNX2 isoform classification (Extended Data Fig. 6b). Inspection of cells expressing constructs in either cluster further confirmed that this clustering captured the isoform-specific granularity phenotype, with aggregates forming in the −EX but not +EX cluster (Fig. 5d,e).

Interestingly, clustering reflected the presence or absence of the four-residue microexon, with all +EX mutants clustering with the +EX isoform regardless of amino acid substitutions within the exon (Fig. 5b). This indicated that residue insertion, rather than residue identity, was the main determinant of the major isoform-specific localization differences, particularly for granularity. Indeed, even the +EX E452A/K mutants, which were the most distinct from the SNX2+EX isoform, showed similar granularity behavior to +EX (Fig. 5d,e and Extended Data Fig. 7g). Among the SNX2−EX mutants, we observed that progressively changing R451 from positive or small to neutral to negative residues (R451K/A→R451Q→R451E) gradually shifted the SNX2−EX constructs from the –EX to the +EX cluster (Fig. 5b) and produced a corresponding shift in granularity behavior (Fig. 5d,e), implicating positive charge at residue 451 as an important determinant of the isoform differences.

Together, these results suggest that the SNX2 microexon functions as an α-helical insertion that extends local structure and alters isoform-related localization differences by repositioning preexisting, flanking charged residues. For the mutants tested here, this effect is influenced more strongly by insertion rather than its precise amino acid composition, although the microexon’s residues may fine-tune certain aspects of localization.

### Microexon-dependent residue displacement fine-tunes SNX2 localization and interactions

**Fig. 6:**
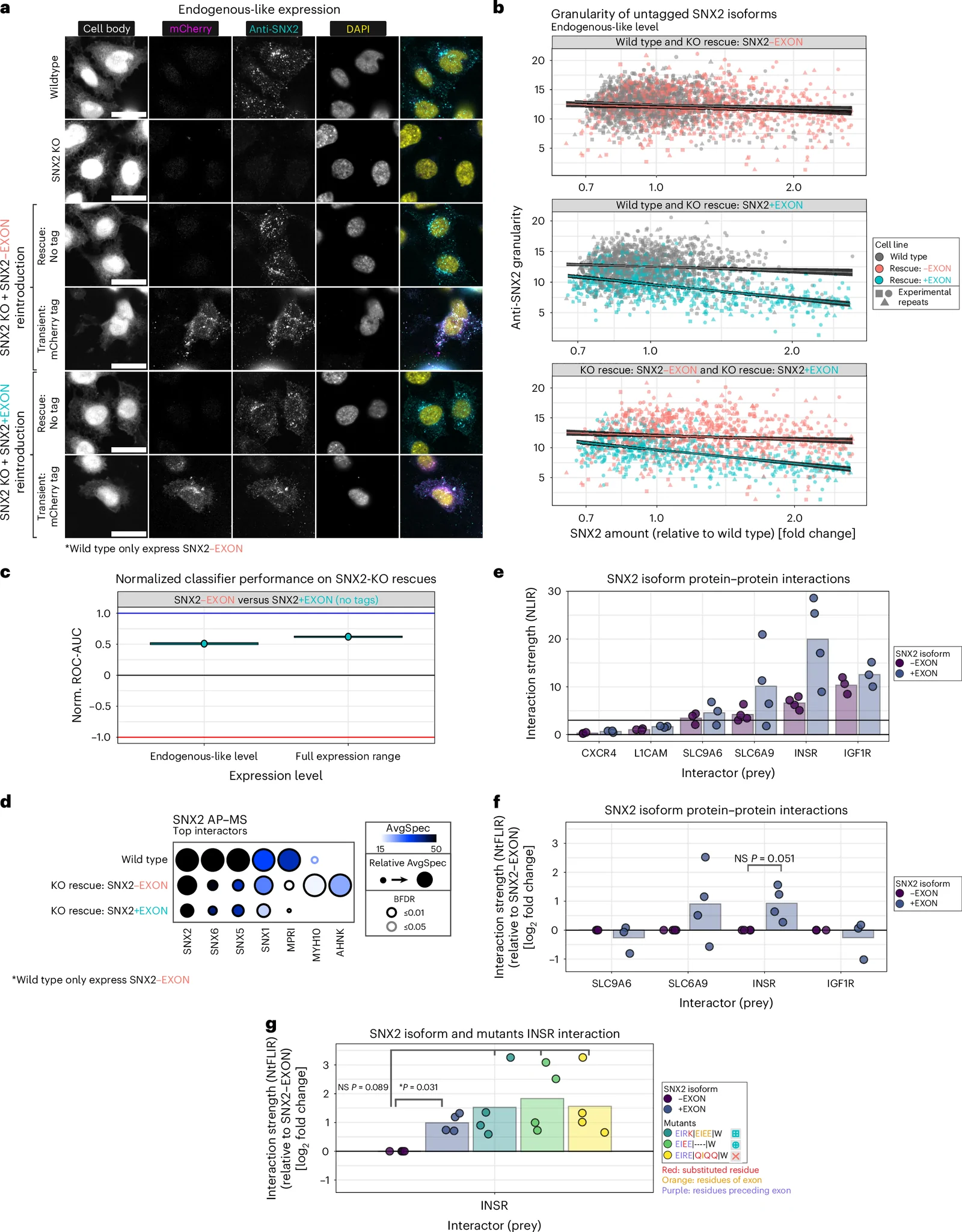
SNX2 isoforms localize differentially at endogenous levels and display different interactions with select protein partners, which relates to residue insertion rather than amino acid composition. **a**, Representative images of HeLa cell lines expressing SNX2 at endogenous-like levels. The cells are either HeLa wild type, HeLa SNX2-KO, HeLa SNX2-KO stably expressing untagged SNX2+EX or SNX2−EX or HeLa SNX2-KO transiently expressing mCherry–SNX2+EX or SNX2−EX. The cell body, mCherry, anti-SNX2 and DAPI channels are shown independently and merged. Representative cells were chosen to have comparable expression levels according to the anti-SNX2 immunostaining signal. Scale bar, 25 µm. **b**, *XY* plots of SNX2 amount (SNX2 intensity relative to the mean SNX2 intensity in HeLa wild type, fold change; *x* axis) against SNX2 granularity 2 (*y* axis) in the HeLa wild-type and SNX2 rescue lines, at endogenous levels. Each point represents a single cell, color represents cell line (*n* wild type = 1,351, *n* rescue SNX2−EX = 736, *n* rescue SNX2+EX = 558) and shape represents experimental repeat (*n* = 3). A linear regression line (*x* ≈ *y*) is fitted to the data, with the 95% confidence interval indicated as a black shade. Cell lines are shown pairwise to reduce overplotting. **c**, Box plot showing the normalized ROC-AUC values from classifier-based discrimination of HeLa SNX2 rescue cells expressing untagged SNX2−EX and SNX2+EX according to the SNX2 signal. The analysis was performed using cells expressing the SNX2 isoforms at either endogenous-like levels (~0.7–1.5× the mean SNX2 intensity in HeLa wild type; *n* cells = 418 per isoform) or the full measured expression range (~0.7–100× the mean SNX2 intensity in HeLa wild type, *n* cells = 763 per isoform). The cell sampling and classifier evaluation was performed 50 times and the box plot shows the distribution of the normalized ROC-AUCs from these iterations. The dot indicates the median (*n* = 50). Box plots show the median (center line), IQR (box ends) and observations within 1.5× the IQR (whiskers). **d**, Bubble plot of proteins detected upon AP–MS analysis of HeLa wild type and HeLa SNX2 isoform rescue lines. Only proteins with average spectral counts ≥ 15 are included (*n* = 2 independent experiments). Colors indicate average spectral counts, sizes indicate relative spectral counts and the line indicates the BFDR. **e**, Bar plot showing LUMIER interaction scores for SNX2 isoforms with six transmembrane proteins that are suggested to interact with and/or be regulated by SNX-BAR complexes. Interaction strength is quantified as NLIR and an NLIR > 3 (black line) is considered a positive interaction^70^. Column height indicates the mean NLIR across experimental repeats (*n* = 3 or 4 independent experiments) and dots reflect the NLIR from each experiment. Colors reflect SNX2 isoform identity. **f**, Bar plot showing LUMIER interaction strength of SNX2+EX with the four transmembrane proteins that were identified as positive interactors (Fig. 6f), relative to the SNX2−EX isoform’s interaction with them (log_2_ fold change). These are the same data used for **e** but the interaction score is normalized to the level of both prey and bait protein (NtFLIR). The column height indicates the mean log_2_ fold change across experiments (*n* = 3 independent experiments for SLC9A6 and IGF1R, *n* = 4 independent experiments for SLC6A9 and INSR). Dots reflect the log_2_ fold change for individual experiments. Colors reflect SNX2 isoforms. Statistical analysis was conducted using a one-sample *t*-test against µ = 0 (two-sided). NS, not significant (*P* > 0.05). **g**, Bar plot showing LUMIER interaction strength of SNX2+EX and three SNX2 mutants with INSR, relative to the SNX2−EX isoform’s interaction with INSR (log_2_ fold change). Individual dots reflect the log_2_ fold change for individual experiments and columns indicate the mean across experiments (*n* = 4 independent experiments). Interaction differences were tested using one-sample *t*-tests against µ = 0 (two-sided). *P* values were corrected for multiple testing using the FDR. *P < 0.05. Source data

**Extended Data Fig. 8:**
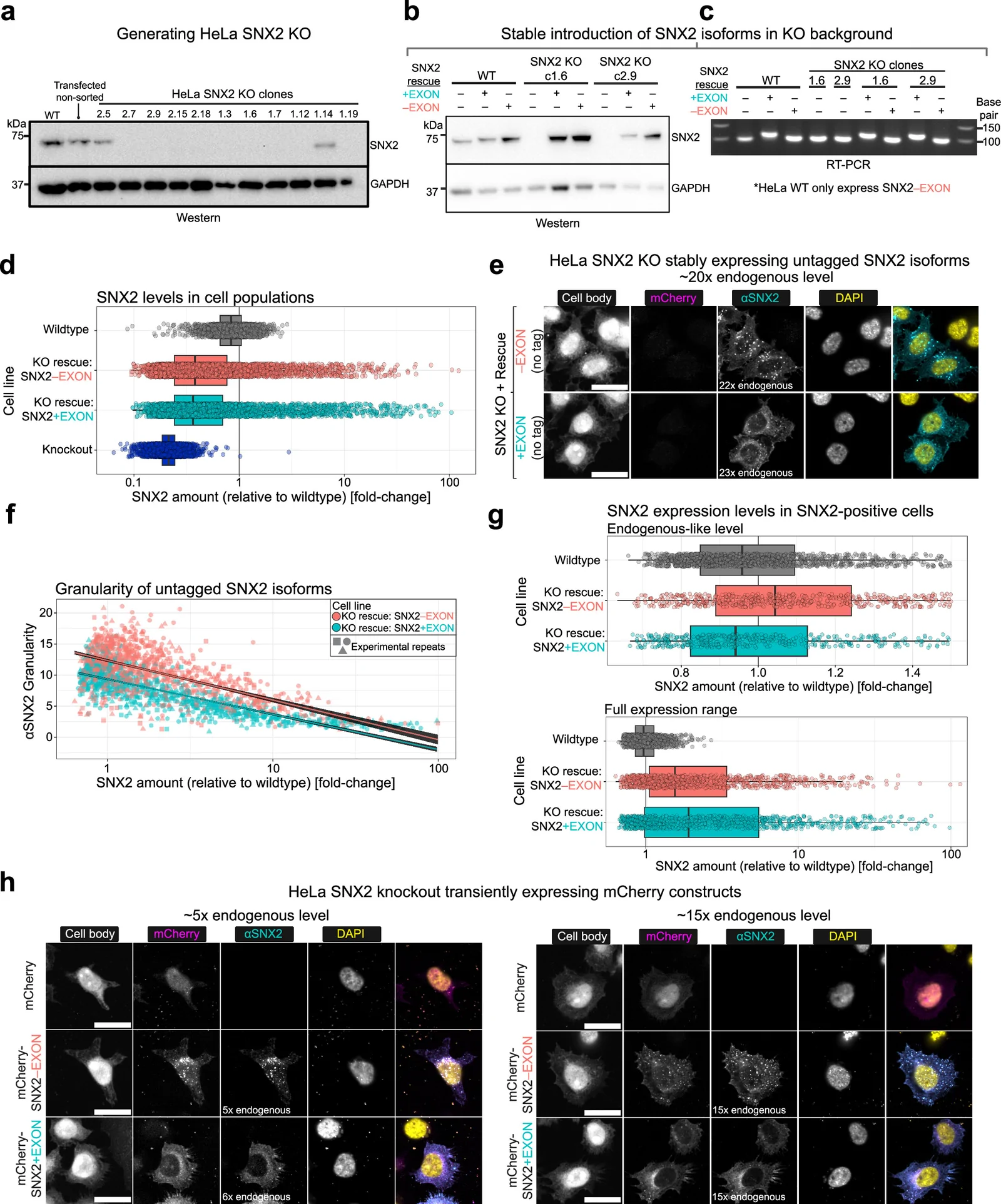
Generation of HeLa cell lines expressing select SNX2 isoforms and evaluation of the mCherry tag. **a**, Western blot of SNX2 and GAPDH in lysates from HeLa cell clones isolated after SNX2 knockout in parental HeLa population. Experiment was performed once. **b**, Western blot against SNX2 and GAPDH in lysates from HeLa wildtype cells or HeLa SNX2 knockout clones, where SNX2-EX or SNX2 + EX had subsequently been re-introduced via lentiviral transduction (SNX2 rescue). Experiment was performed once. **c**, Gel image of PCR products from end-point RT-PCR amplification using primers flanking the SNX2 microexon with total RNA from HeLa wildtype or HeLa SNX2 lines as input. Expected PCR product sizes are 108nt ( + EX) and 96nt (-EX) based on presence/absence of 12nt microexon. HeLa wildtype only express SNX2-EX. Residual amplification in knockout cells likely reflects low levels of SNX2 transcripts, as the knockout targets a region upstream of the amplified fragment. Experiment was performed once. **d**, Boxplot of SNX2 expression levels (SNX2 intensity, relative to the mean SNX2 intensity in HeLa wildtype population [fold-change], measured via αSNX2 antibody) of all cells within the indicated HeLa lines. Each dot represents a single cell. Colour of dots and boxplots represents the cell line. n cells wildtype = 2570, Rescue SNX2-EX = 4848, Rescue SNX2 + EX = 4488, Knockout = 5579. Cells were pooled from N = 3 experimental repeats. See 2e for boxplot description. **e**, Representative images of HeLa SNX2 rescue lines stably over-expressing untagged SNX2 isoforms at 20-25x endogenous levels. The cell body, mCherry, αSNX2, and DAPI channels are shown independently and merged. Representative cells were chosen to have comparable expression levels to αSNX2. Relative SNX2 expression (compared with the wildtype mean) is indicated below each image. Scale bar: 25 µm. **f**, Relative SNX2 amount (SNX2 intensity relative to the mean SNX2 intensity in HeLa wildtype [fold-change)) against SNX2 granularity 2 across rescue lines spanning ~0.7-100x endogenous expression. Point represents individual cells, coloured by cell line (n Rescue SNX2-EX = 1053, Rescue SNX2 + EX = 960), and shaped by experimental repeat (N = 3). Line shows linear regression with the 95% confidence interval. **g**, Boxplots showing the SNX2 amount within HeLa wildtype cells or HeLa SNX2 rescue cells expressing untagged SNX2-EX or SNX2 + EX, relative to the population mean of HeLa wildtype [fold-change]. The HeLa SNX2 rescue populations were used for classifier-based discrimination of the untagged SNX2 constructs in Fig. 6c. at endogenous-like level range ( ~ 0.7 to 1.5x wildtype) and full (0.7-100x) expression range. Colours indicate cell line (grey, HeLa-wildtype, red, Rescue SNX2-EX, blue, Rescue SNX2 + EX). See 2e for boxplot description. **h**, Representative images of HeLa SNX2 KO cells transiently expressing mCherry constructs corresponding to ~5x (left) or ~15x (right) endogenous SNX2. Cell body, mCherry, αSNX2, and DAPI channels are shown independently and merged. Representative cells were chosen to have comparable expression levels based on the αSNX2 immunostaining signal. Relative expression level of αSNX2 (vs HeLa wildtype) is indicated in the bottom of each image. Scale bar: 25 µm. Source data

To determine whether the effects of the microexon persist for isoforms expressed at near endogenous levels and irrespective of tagging and whether the effects extend beyond localization, we next examined SNX2 isoform localization at endogenous-like levels and whether the isoforms differed in their interaction networks. We first knocked out SNX2 in HeLa cells (Extended Data Fig. 8a,b), which endogenously only express SNX2−EX (Extended Data Fig. 8c) and then stably reintroduced untagged SNX2−EX or SNX2+EX by lentiviral transduction (rescue), as confirmed by western blotting and endpoint reverse transcription (RT)–PCR (Extended Data Fig. 8b,c). The rescue lines were heterogenous and expressed the SNX2 isoforms across varying expression levels (Extended Data Fig. 8d). By immunostaining the SNX2-knockout (KO) rescue cells alongside wild-type HeLa cells with a pan-SNX2 antibody and imaging them using confocal microscopy, we could compare SNX2 isoform behavior at varying expression levels by examining cells expressing the isoforms within specific ranges. This confirmed that both isoforms were cytosolic (Supplementary Fig. 4) and SNX2−EX displayed a more granular, punctuated localization than SNX2+EX at endogenous-like expression levels (Fig. 6a,b), with the same qualitative difference becoming more exaggerated upon overexpression (Extended Data Fig. 8e,f). Concordantly, our classifier-based approach confidently assigned correct isoform identities to the rescue cells at both endogenous-like levels (normalized ROC-AUC ≈ 0.5) and across the full expression range of the rescue cell populations (normalized ROC-AUC ≈ 0.6) (Fig. 6c and Extended Data Fig. 8g), supporting the differential localization of untagged SNX2 isoforms.

**Extended Data Fig. 9:**
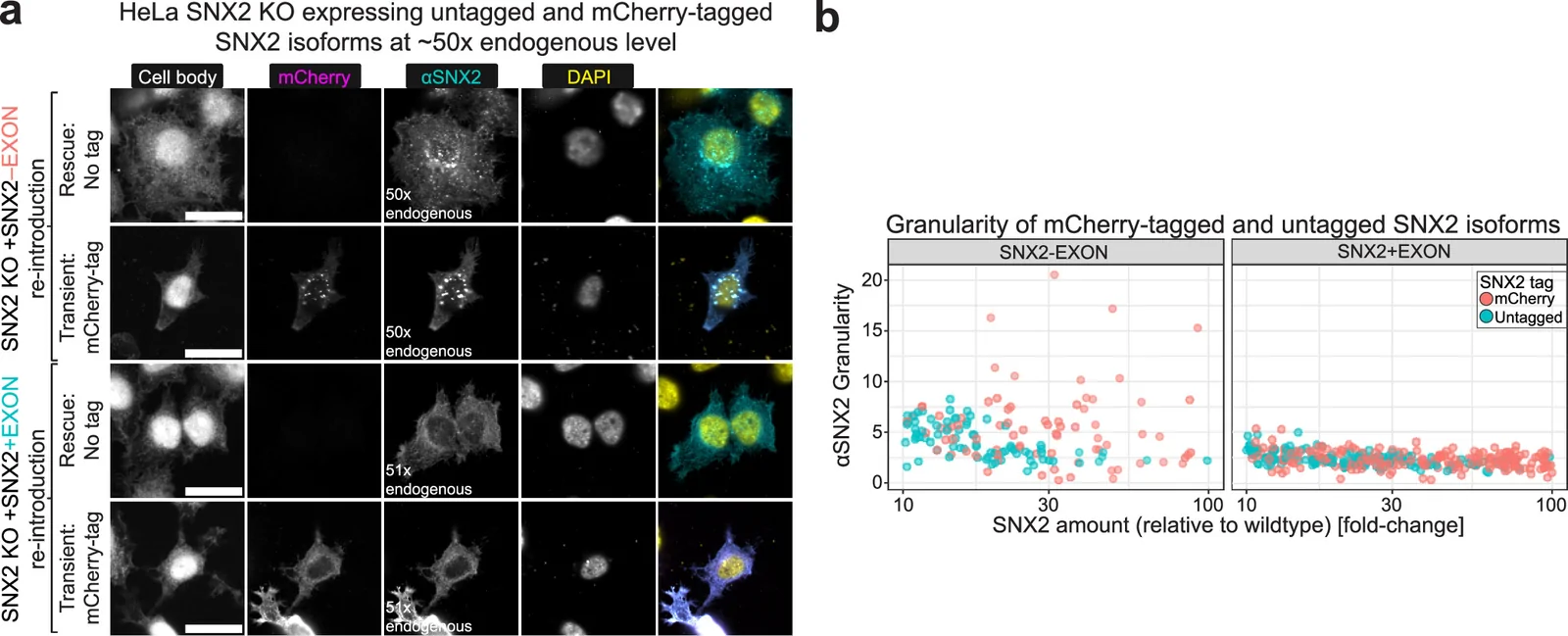
The SNX2 aggregates formed at higher expression levels are isoform-specific and dependent on the mCherry-tag. **a**, Representative images of HeLa cell lines over-expressing untagged and mCherry-tagged SNX2 isoforms at 50x endogenous levels. The cells are either HeLa SNX2 KO stably expressing untagged SNX2 + EX or SNX2-EX, or HeLa SNX2 KO transiently expressing mCherry-SNX2 + EX or SNX2-EX. The cell body, mCherry, αSNX2, and DAPI channels are shown independently and merged. Representative cells were chosen to have comparable expression levels based on the αSNX2 immunostaining signal. The expression level of αSNX2 relative to the HeLa wildtype mean SNX2 level is indicated in the bottom of each image. Scale bar: 25 µm. **b**, XY plot of SNX2 granularity versus SNX2 amount for cells over-expressing untagged and mCherry-tagged SNX2 isoforms. Each dot represents a cell with a SNX2 amount of 10-100x that of the population mean of HeLa wildtype. The plot is split according to exon status (SNX2-EX, SNX2 + EX), and dot colour indicates SNX2 tag status. n cells: untagged SNX2-EX n = 76, mCherry-SNX2-EX n = 76, untagged SNX2 + EX n = 133, mCherry-SNX2 + EX, n = 196. The cells were pooled from N = 3 experimental repeats. Source data

To evaluate the impact of the mCherry tag on SNX2’s behavior, we next transfected the SNX2-KO cells with the mCherry–SNX2 isoforms. At endogenous-like and lower overexpression levels, we observed similar localization behavior between untagged and tagged isoforms (Fig. 6a and Extended Data Fig. 8h), supporting the tag’s use in examining SNX2 localization at lower expression levels. By contrast, at higher expression, aggregate formation was only observed for mCherry-fused SNX2−EX (Extended Data Fig. 9a,b), indicating that the observed aggregates are tag dependent and likely arise from the tag accentuating isoform-specific behaviors, as we did not observe it for mCherry–SNX2+EX. Collectively, these results show that the SNX2 microexon promotes a more diffuse, less vesicular localization pattern that is evident at endogenous-like expression levels in untagged SNX2.

To then assess whether these structural and localization changes affected protein interactions, we compared PPIs between SNX2 isoforms and select SNX2 mutants using affinity purification (AP)–MS and LUMIER assays. We performed AP–MS of SNX2 in the HeLa SNX2 rescue lines stably expressing either isoform (untagged) (Supplementary Tables 7 and 8), which showed that both isoforms interacted with canonical SNX-BAR partners (SNX1, SNX5, SNX6)^53,54^ and cargo (MPRI)^55^ (Fig. 6d), confirming intact SNX-BAR complex formation. We then performed LUMIER to quantify interactions with SNX-BAR cargo proteins, examining interactions with six transmembrane cargos. We found both isoforms interacted with most cargos to similar extents (Fig. 6e,f); however, SNX2+EX had an approximately twofold stronger interaction with the insulin receptor (INSR) (Fig. 6f). Crucially, this enhanced binding persisted in two +EX mutants and the –EX R451E mutant (Fig. 6g), all of which clustered with SNX2+EX in the imaging experiment and did not form aggregates. Therefore, the four-residue insertion, rather than the precise amino acid identity of the microexon, was sufficient to alter interaction strength. Collectively, these findings demonstrate that inclusion of the conserved SNX2 four-residue microexon repositions charged residues within the BAR domain, which subtly alters electrostatics to fine-tune both subcellular localization and PPIs, exemplifying one mechanism by which microexons can alter structured regions.

## Discussion

In this study, we revealed that a common feature of AS is to fine-tune protein networks and subcellular localization by modulating protein residue composition, for instance, through microexon splicing, which changes the electrostatic properties at individual interaction interfaces. Within tissues, such fine-tuning enables proteins to preserve their core molecular functions while acquiring context-specific properties that modulate specialized interactions, whereby isoforms can contribute to cellular identity and physiology^10,46,56,57^. An example of this is a gastric epithelium-specific exon in MLCK1, which promotes tight junction localization, enabling this ubiquitously expressed protein kinase to regulate gastric barrier integrity through isoform-specific interactions^58^. This behavior is relevant for inflammatory bowel disease^58,59^ and blocking the isoform-specific interaction can alleviate disease symptoms^59^, illustrating the potential clinical benefits of understanding how AS fine-tunes protein behaviors, such as localization^11,60^.

Our network analysis revealed that AS preferentially targets multidomain, multifunctional proteins containing binding surfaces for multiple interaction partner types (proteins, RNA, DNA and lipids). We speculate that their multimodular nature makes them particularly amenable to AS, which can prune or enhance specific interfaces while preserving others^10,61,62^. By resolving AS effects at the interface rather than gene level, we captured how tissue-specific AS generally modifies network connectivity (local changes, with preservation of global structure) with distinct properties for different exon types. Longer exons predominantly act within functional modules, often functioning as digital switches that add or remove individual interactions, whereas microexons operate at intercommunity interfaces, fine-tuning rather than removing key connections. This dual mode of regulation provides a mechanistic basis for how AS can simultaneously refine local specificity and maintain global coordination across the proteome.

The observed impact of AS on network connectivity and its association with localization diversity prompted us to develop an imaging approach to screen whether isoform diversity manifests as changes in localization. We anticipate that such approaches will complement the more traditional interaction-based studies of protein isoform. Although relatively small, our screen identified that nearly half of the tested isoform pairs differed in subcellular distribution when examined at comparable expression levels, supporting our network-level predictions and suggesting that AS may frequently alter protein localization. This is consistent with a recent study of transcription factor isoforms, which found that ~50% of isoforms showed localization differences in the form of nuclear versus cytoplasmic localization and/or condensate formation^63^. Whereas that study examined complete transcript isoforms, our analysis focused on individual splicing events and identified a similar extent of subcellular regulation. This suggests that even single exons can alter protein localization, although combining multiple splicing events may produce stronger effects. Together, these findings argue that isoform-level information is important for understanding how protein localization is specified and for improving computational methods that predict protein localization from sequence^64,65^.

Our imaging data suggest that AS-driven localization changes are often graded rather than digital, fine-tuning the degree of subcellular localization rather than completely relocalizing a protein. This is illustrated in our screen by the ANK2 isoforms, which both localize to the plasma membrane but to different extents (Fig. 2g), and by the SNX2 isoforms, which localize to vesicular structures with varying enrichment. Our structural analyses indicated that such graded localization can arise through at least two mechanisms. First, AS can add or remove domains (or motifs)^10^. This occurs for ANK2, where alternative inclusion of a targeting module (specifically, a ZU5 domain) shifts the equilibrium of membrane association, yielding graded behavior across isoforms. Second, AS can remodel existing interfaces without altering domain structure, subtly tuning molecular interactions while preserving structural integrity. This is exemplified by SNX2, where microexon inclusion alters preexisting interaction surfaces. Specifically, SNX2’s microexon extends a BAR domain α-helix near the BARtip and repositions surrounding residues, including R451. This may alter contacts formed between SNX-BAR dimers during the assembly of higher-order protein coats such as ESCPE1 (ref. ^66^), consistent with the interaction differences observed in the LUMIER assay. Consistent with this model, SNX2 mutagenesis showed that isoform-specific differences in localization and partner specificity arise largely independently of the exon’s amino acid composition, implying a structural rather than compositional mechanism. A similar mechanism operates in SRC kinase, where a microexon within the SH3 domain switches the domain’s peptide-binding preferences from a PXXP motif to a RXPXXP motif^67^, thereby fine-tuning the composition and specificity of the signaling complex. These findings support a broader model in which AS, particularly through microexons, introduces small structural perturbations that retune molecular recognition and localization without compromising protein fold or stability.

This study had several limitations. First, the fraction of interfaces classified as AS-regulated is likely conservative, as our analysis was restricted to experimentally supported, annotated interaction interfaces, which are incomplete and biased toward structured, well-characterized interaction regions^68,69^. As tissue-regulated AS is enriched in intrinsically disordered regions and short linear motifs^17^, many AS-sensitive interfaces likely remain unannotated. In addition, our framework was limited to frame-preserving AS events that directly overlap annotated interfaces, providing a physically interpretable view of direct interface rewiring while excluding allosteric and other distal effects and frame-altering events. Therefore, our conclusions apply primarily to direct, interface-overlapping, frame-preserving AS events, which represent a tractable subset of the broader landscape of AS-mediated rewiring. Second, the imaging-based localization assays were performed primarily under transient overexpression of mCherry-tagged isoforms rather than conditions that fully recapitulate endogenous stoichiometries. Although this enabled controlled comparison of matched isoform pairs, overexpression may modify phenotypes that depend on interaction partner abundance, while tagging may also affect localization. We addressed this by controlling for expression range, comparing identically tagged isoforms and, for SNX2, observing a similar qualitative isoform-specific difference in an untagged rescue setting at endogenous-like expression, albeit with smaller magnitude. Thus, the assay likely captures intrinsic sequence-dependent localization differences, although their magnitude may vary with expression context. More broadly, the assay does not by itself resolve the mechanistic basis of localization differences. Beyond direct effects on targeting motifs or interaction interfaces, AS may alter localization indirectly through changes in post-translational regulation or protein abundance^10,33^. The assay is less suited to identifying abundance-driven localization effects because it is designed to compare isoforms expressed at similar levels^10,33^.

**Extended Data Fig. 10:**
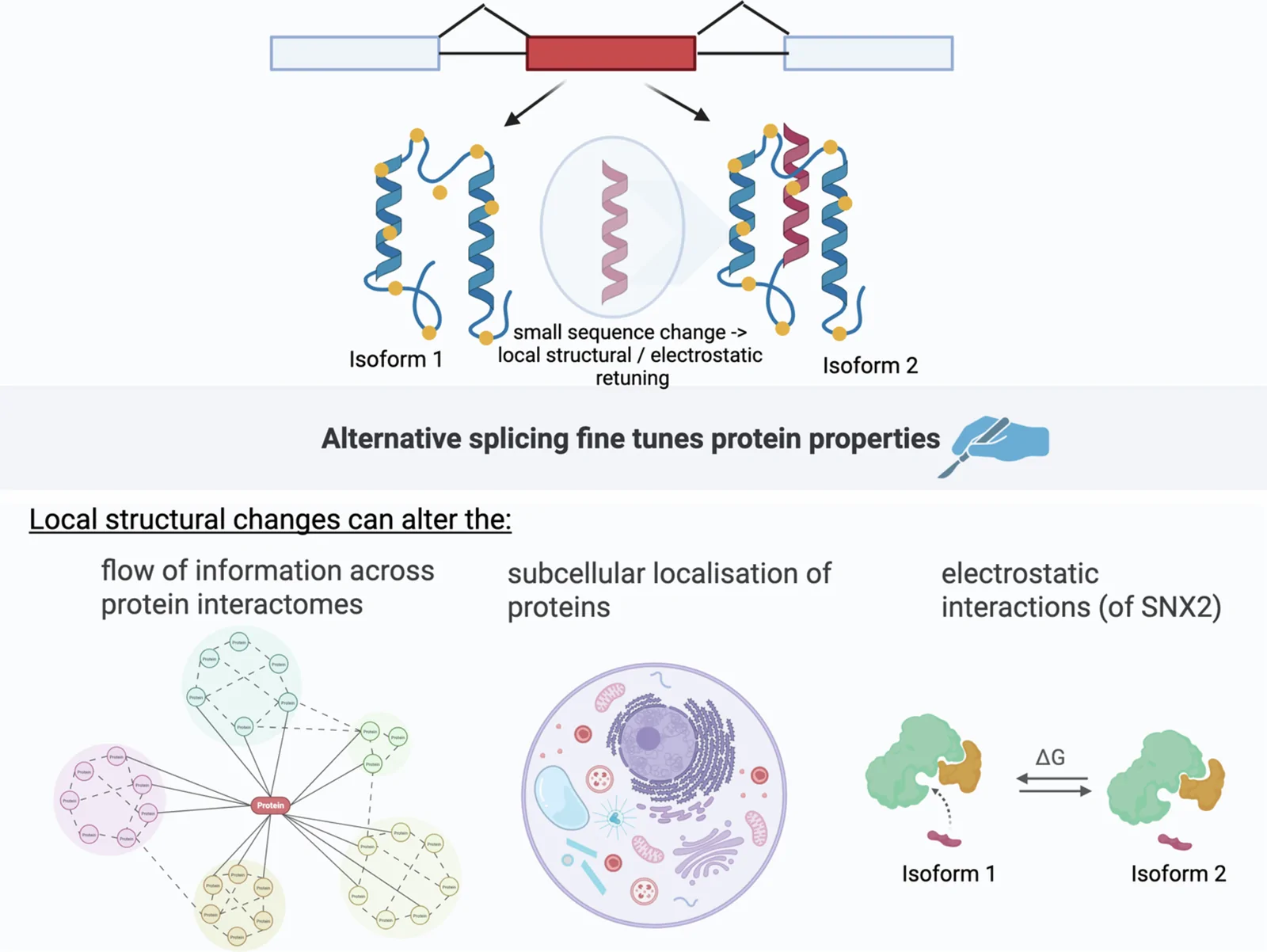
Visual abstract. Alternative splicing fine-tunes protein properties by inducing local structural and physiochemical changes that can alter protein interactions, subcellular localisation and binding energetics, as exemplied by SNX2 isoforms.

Together, our results support a model in which AS fine-tunes proteome organization by regulating protein modular architecture (Extended Data Fig. 10). By coupling interface-level adjustments to changes in localization and interaction specificity, AS provides a flexible yet robust means of evolving new functional capacities while preserving overall cellular architecture.

## Methods

The reagents (Supplementary Table 9), plasmids (Supplementary Table 10) and oligonucleotides (Supplementary Table 11) used are provided in the Supplementary Information. Antibody and dye dilutions are included in Supplementary Table 9.

### Molecular cloning

#### Gateway recombination

Most subcloning of protein isoform ORFs was carried out using the Gateway system (Thermo Scientific), using BP and LR clonase II enzyme mixes (donor: pDONR303, destination: mCherry–N and C–mCherry). Gateway cloning was similarly used to generate the SNX2 isoform lentiviral plasmids (destination: pLX303)^71^ and the bait and prey plasmids used for LUMIER (destinations: 3×FLAG–N or C–3×FLAG, *Renilla* luciferase–N) gifted by B. J. Blencowe’s group. Plasmids gifted by researchers through Addgene are listed in Supplementary Table 10.

The Gateway BP reactions were set up according to manufacturer’s protocol. If the insert of interest was not in a plasmid flanked by attB1/attB2 sites, these sites were introduced by PCR with primers tailed by the attB1/attB2 recombination sites. The PCR product was then used for BP reaction. If the insert of interest was already in a plasmid flanked by attB1/B2 sites, the plasmid was linearized by restriction enzyme digestion before the BP reaction. The BP reactions were generally incubated 2–3 h at 25 °C before proteinase K-based inactivation and 30% of the reaction was then used for bacterial transformation. The Gateway LR reactions were set up in a reaction volume half of that described by the manufacturer with every component halved, including DNA amounts, but were otherwise performed according to the manufacturer’s protocol. The LR reactions were generally incubated 1–2 h at 25 °C before proteinase K-based inactivation and 50% of the reaction was then used for bacterial transformation. If BP or LR reactions were not used immediately for bacterial transformation, the reaction was frozen at −20 °C and used within 1 week.

#### Site-directed mutagenesis

Protein isoform ORFs not obtained from the B. J. Blencowe lab were generated by site-directed mutagenesis. Similarly, site-directed mutagenesis was used to generate the SNX2 mutants and introduce stop codons in a few ORFs. All site-directed mutagenesis was performed using the Q5 site-directed mutagenesis kit (New England Biolabs) according to the manufacturer’s protocol. All plasmids (generated by Gateway recombination or site-directed mutagenesis) were verified by Sanger sequencing at the Garvan Molecular Genetics facility.

#### Bacterial transformation

Home-made DH5α *Escherichia* *coli* was used for most transformation reactions. Briefly, 50 µl of cells in 2-ml Eppendorf tubes were used per transformation. The cells were mixed with 1–10 ng of plasmid DNA, 30% of a BP reaction, 50% of an LR reaction or 50% of a site-directed mutagenesis reaction and then incubated on ice for 30 min. The cells were then heat shocked at 42 °C for 1 min and then incubated on ice for 2 min. Then, 900 µl of room temperature Luria–Bertani (LB) medium was added and the tubes were incubated in a shaking incubator (37 °C, 200–250 rpm) for 1 h. The bacterial suspension was then plated on LB agar plates with the appropriate antibiotic for the plasmid in question. Ampicillin was used at 100 µg ml^−1^, kanamycin was used at 50 µg ml^−1^ and spectinomycin was used at 75 µg ml^−1^. Transformations of constructs that transformed less efficiently (for instance, BP reactions of large inserts), were conducted using commercial DH5α *E*. *coli* (New England Biolabs, C2987H) according to the manufacturer’s protocol, using the same DNA input amounts as listed for home-made DH5α *E*. *coli*. Transformation of plasmids used for lentivirus production was conducted using commercial OneShot Stbl3 *E*. *coli* (Thermo Scientific, C737303), according to the manufacturer’s protocol. The Stbl3 cells were incubated at 30 °C rather than 37 °C.

Home-made DH5α *E*. *coli* was derived from commercial DH5α *E*. *coli* (New England Biolabs, C2987H) as previously described^72^.

#### Plasmid purification

Plasmids were purified from liquid cultures of bacterial transformants expressing the plasmid of interest, using various plasmid purification kits. The bacterial input amount and purification were according to the manufacturer’s protocol in all cases. Minipreps were performed using Promega’s Wizard Plus SV miniprep kit (A1330), midipreps were performed using Macherey-Nagel’s NucleoBond Xtra midi kit (740410) and maxipreps were performed using Promega’s PureYield kit (A2392). Liquid bacterial cultures were grown at 37 °C and 200–250 rpm for nonlentiviral plasmids and at 30 °C and 200–250 rpm for lentiviral plasmids.

#### Routine cell culture

HeLa cells (wild-type, stable and KO lines) and HEK293T wild-type cells were grown in noncoated TC-treated polystyrene plasticware (Corning) in DMEM (high glucose, sodium pyruvate; Thermo Scientific, 11995065) supplemented with 10% FBS, with or without Pen/Strep (penicillin: 100 U per ml, streptomycin: 100 µg ml^−1^), at 37 °C, 5% CO_2_ in a humidified cell incubator. Both cell lines were passed by washing the cells 2× in heated 1× PBS (137 mM NaCl, 10 mM Na_2_HPO_4_, 1.8 mM KH_2_PO_4_ and 2.7 mM KCl) and then treating the cells with trypsin-EDTA 0.25% for 4 min at 37 °C to detach them. The cells were then resuspended in supplemented DMEM and spun at 200*g* for 4 min at room temperature; the supernatant was discarded. The cell pellet was resuspended in fresh supplemented DMEM, counted using a Countess II automated cell counter, diluted further in supplemented DMEM to the desired cell density and used for downstream assays or continued culture.

HeLa and HEK293T wild-type cells were gifted by R. Lyons (Garvan Tissue Culture Facility manager).

### Cell line generation

#### CRISPR–Cas9-based gene KO

First, we cloned gRNAs targeting SNX2, either previously described^73^ or predicted using the CRISPRscan online tool^74^, into the pSpCas9(BB)-2A-GFP (PX458)^75^ (Addgene, 48138) plasmid under a U6 promoter. The gRNAs generally targeted exon junctions of exon 2 and were predicted to cause gene KO by disrupting exon 2 usage. The gRNA introduction into the plasmids was performed as described by Ran et al. for synthetic oligonucleotides^75^. Briefly, the gRNA oligo and the complementary sequence were ordered separately and then annealed in vitro. Both the gRNA and the complementary strand were ordered with overhanging 5′-ends that are complementary to the BbsI-generated sticky ends in the pSpCas9(BB)-2A-GFP (PX458) plasmid. The annealed gRNA and complementary strand could, therefore, be inserted into the plasmid by DNA ligation. All oligos were obtained from Integrated DNA Technologies.

To generate the SNX2-KO HeLa cells, 3 µg of gRNA-containing pSpCas9(BB)-2A-GFP (PX458) plasmid was transiently transfected into HeLa cells in p60 dishes using ViaFect transfection reagent (4:1 ratio of reagent to DNA) according to the manufacturer’s protocol. Notably, the two plasmids with different SNX2-targeting gRNAs were transfected into different p60 dishes to generate independent SNX2-KO HeLa cell lines. The cells were incubated for 48 h after transfection at normal culture condition. The cells were then detached and spun as for normal cell passage, then resuspended in 1× PBS with 2% FBS and Pen/Strep and passed through a 35-µm nylon mesh to remove cell clumps. The cell suspension was then sorted by fluorescence-activated cell sorting on a BD FACS Aria III (nozzle diameter: 100 µm) to isolate EGFP-positive cells, which were sorted into 96-well plates with one cell seeded per well. The wells contained 50 µl of fresh DMEM supplemented with Pen/Strep and 50 µl of conditioned DMEM. The single clones were then allowed to grow into individual clonal lines. Clonal lines were subsequently tested for successful gene KO by western blotting.

Conditioned DMEM was generated by aspirating the medium from HeLa cells after the cells had been incubated with the medium for 24–48 h. The isolated medium was then spun at 3,220*g* for 10 min at 4 °C to pellet potential cell debris and the supernatant was filtered through a 0.22-µm filter. The filtered supernatant was used as conditioned DMEM.

#### Stable cell lines

We generated cell lines that stably expressed the gene of interest by lentiviral transduction. We generated the lentiviruses by cotransfecting 60–80% confluent HEK293T in T-25 flasks with three plasmids: 4 µg of psPAX2 (packing vector; Addgene, plasmid 12260), 2 µg of pCAG-VSVG (envelope vector; Addgene, plasmid 35616) and 4 µg of pLX303 lentiviral plasmid containing the ORF for one of the two SNX2 isoforms. The transfection was performed using Lipofectamine 3000 reagent (Thermo Scientific) (1.5:1 ratio of reagent to DNA, 2:1 ratio of P3000 to DNA), according to the manufacturer’s protocol. Then, 24 h after the transfection, the medium was changed to supplemented DMEM. After another 24 h, the medium was isolated from the HEK293T and spun at 2,500*g* for 10 min at 4 °C to pellet cell debris. The supernatant was then filtered through at 0.45-µm filter and this filtered supernatant was used for viral transductions (virus-containing supplemented DMEM not used immediately was frozen at −70 °C for later use).

To transduce the target cells, the virus-containing supplemented DMEM was added to the medium of HeLa cells (either HeLa wild-type or SNX2-KO clones) in six-well plates. The cells were incubated for 48 h with the virus and the medium was then removed. The cells were washed ten times in 1× PBS; then, fresh supplemented DMEM was added and the cells were removed from the PC2 facility. The cells were subsequently passed (after 30 min of rest in a cell incubator) and seeded into new six-well plates in supplemented DMEM with 2 µg ml^−1^ blasticidin to select for transduced cells. The cells were treated for 4 days with blasticidin at this concentration (medium changed every second day) and passed as normal when they reached confluency. After 4 days, the blasticidin concentration was increased to 10 µg ml^−1^ to make sure surviving cells were transduced. This concentration was used for 6 days, after which the drug was removed and the cells were cultured as per normal to grow cells for freezing. Upon reviving frozen stocks of the stable cells, they were cultured at 3 µg ml^−1^ blasticidin when not used for experiments to keep selecting for cells expressing the transgene. The drug was removed when cells were used for AP–MS or immunofluorescence. Expression of the transgenic SNX2 (either +EXON or –EXON isoforms) was verified by western blotting and endpoint RT–PCR.

Generation of stable cells by viral transduction was performed in a Garvan PC2 laboratory designated for viral work (the point at which the cells were removed from the facility is specified), in accordance with Garvan’s PC2 guidelines.

### Cell preparation for imaging

#### Protein isoform library

HeLa wild-type cells were detached, counted and diluted in supplemented DMEM (as above) to make a cell suspension with a density of 90,000 cells per ml. Then, 500 µl of the cell suspension was added into each well in a glass-bottom 24-well plate (Cellvis) (45,000 cells per well). Immediately after adding the cell suspension to the wells, the cells were reverse-transfected by dripping in 50 µl of transfection mix consisting of 400 ng of plasmid encoding mCherry-tagged construct mixed with ViaFect (4:1 ratio of reagent to DNA) in OPTI-MEM as per the manufacturer’s protocol. Each well was transfected with a different construct (except the negative controls). One well was left untransfected and, instead, 50 µl of OPTI-MEM was added. Three experimental replicates for a given protein isoform batch were set up sequentially on the same day with cell suspensions and transfection mixes made separately for each replicate.

After seeding and reverse transfection, the cells were incubated for 24 h in the cell incubator. After the incubation, all wells were washed twice in 500 µl of heated 1× PBS and then fixed with 200 µl of 4% paraformaldehyde for 10 min at room temperature. The wells were subsequently washed three times in 500 µl of 1× PBS and 1 ml of 1× PBS was then added to each well. The well plate was then wrapped in parafilm and kept at 4 °C until used for immunofluorescence staining (within 7 days of fixation).

#### SNX2 mutants

HeLa wild-type cells were detached, counted and diluted in supplemented DMEM (as above) to make a cell suspension with a density of 100,000 cells per mL. Then, 450 µl of the cell suspension was added into each well in a poly(D-lysine)-coated glass-bottom 24-well plate (Cellvis) (45,000 cells per well). Cells were incubated for 16 h in a cell incubator and were then forward-transfected with plasmids encoding mCherry–SNX2 constructs (both endogenous isoforms and mutants) or mCherry itself by dripping in 50 µl of transfection mix consisting of 375 ng of plasmid DNA mixed with jetPRIME (2:1 ratio of reagent to DNA) in jetPRIME buffer as per the manufacturer’s protocol. Each well was transfected with a different construct (except three wells that were all transfected with mCherry–SNX2−EX). One well was left untransfected and, instead, 50 µl of jetPRIME buffer was added. Three experimental replicates were set up sequentially on the same day with cell suspensions and transfection mixes being made separately for each replicate.

The cells were fixed 24 h after transfection by removing the medium and adding 200 µl of 4% paraformaldehyde for 10 min at room temperature. The fixed sample was washed and stored as for the protein isoform library samples.

#### Untagged SNX2 in HeLa SNX2 cell lines

HeLa wild-type and SNX2 lines (KO and rescue cells) were handled as described above to generate samples for imaging of either untagged, stably expressed SNX2 isoforms (nontransfected wells) or mCherry–SNX2 isoforms in the KO background.

#### Immunofluorescence staining of protein isoform pairs and SNX2 mutants

The well plates with fixed, transfected cells were stained for imaging within 7 days of fixation. All replicates for a given protein isoform batch or SNX2 mutants were stained at the same time. The staining was performed in seven steps. Between each step, the wells were washed 2–4 times with 500 µl of 1× PBS per wash. During incubations, the well plates were covered with aluminum foil. In the first step, cells were permeabilized in 200 µl of 1× PBS with 0.2% Triton X-100 for 12 min at room temperature. In the second step, the cells were blocked in 200 µl of 1× PBS with 3% BSA for 60 min at room temperature. In the third step, the cells were then stained in 200 µl of 1× PBS with 3% BSA and mouse anti-Golgin97 (0.4 µg ml^−1^) for 120 min at 37 °C. In the fourth step, the cells were stained in 200 µl of 1× PBS with 3% BSA and goat anti-mouse–Alexa488 (3 µg ml^−1^) for 60 min at room temperature. In the fifth step, the cells were stained in 200 µl of 1× PBS with DAPI (1 µg ml^−1^) for 15 min at room temperature. In the sixth step, the cells were stained in 200 µl of 1× PBS with NHS ester–Alexa647 (0.125 µg ml^−1^) for 30 min at room temperature (the NHS ester was made up just before being added to the cells). In the final step, the cells were washed four times in 500 µl of 1× PBS and a final 1 ml of 1× PBS was added to each well to prevent the samples from drying out. The stained well plates were wrapped in parafilm and kept at 4 °C until used for imaging (typically less than 1 week).

#### Immunofluorescence staining of SNX2 in HeLa SNX2 lines

The samples were stained as described above except the primary antibody was a mouse anti-SNX2 antibody (1.25 µg ml^−1^).

### Imaging

#### Imaging of protein isoform library

Fixed and immunofluorescence-stained cells were imaged using a PerkinElmer Operetta CLS system using its spinning disk confocal setting (Supplementary Table 12). The cells were imaged with a ×40 (1.1 NA) water-immersion objective, resulting in fields of view (FOVs) of 0.32 mm × 0.32 mm (2,160 × 2,160 pixels), with a pixel size of 0.148 µm × 0.148 µm. We acquired ~50 FOVs per sample per experimental repeat, with 14 slices being acquired per FOV and 0.5 µm between each slice (*z* dimension). Three biological repeats were generated for each isoform batch.

Four channels (referred to as DAPI, Golgin97–Alexa488, mCherry and NHS ester–Alexa647) were acquired for each slice using the excitation, emission and exposure specified as follows (the microscope uses light-emitting diodes (LEDs) with bandpass filters (BPFs) rather than lasers for excitation): DAPI, excitation by 365-nm LED with 355–385-nm BPF, emission captured with 430–500-nm BPF and exposure at 130 mW for 100 ms; Golgin97–Alexa488, excitation by 475-nm LED with 460–490-nm BPF, emission captured with 500–550-nm BPF and exposure at 110 mW for 500 ms; mCherry, excitation by 550-nm LED with 530–560-nm BPF, emission captured with 570–650-nm BPF and exposure at 170 mW for 2,500 ms; NHS ester–Alexa647, excitation by 630-nm LED with 615–645-nm BPF, emission captured with 655–760-nm BPF and exposure at 120 mW for 700 ms.

#### Imaging SNX2 mutants

The SNX2 mutant samples were imaged similarly to the protein isoform library except only eight slices were acquired per FOV with a step size of 1.0 µm. A total of 41 FOVs were acquired per sample per repeat. Three biological repeats were generated.

#### Imaging of SNX2 in HeLa SNX2 lines

The HeLa SNX2 cell line samples were imaged similarly to the protein isoform library except only eight slices were acquired per FOV with a step size of 1.0 µm. A total of 46 FOVs were acquired per sample per repeat. Here, the green channel corresponded to SNX2. Three biological repeats were generated.

### Image processing, cell measurements, cell filtering and transfection status

#### Protein isoform screen and SNX2 mutants

We performed analysis of single slices from the acquired confocal stacks, using the slice corresponding to where the cell adheres to the glass surface.

The acquired confocal stacks were processed as outlined in Supplementary Fig. 2a and Supplementary Table 12. This was achieved by first identifying the focus slice in each confocal stack using ImageJ (version 1.54f)^76^ and the plugin Select focus slice (version 1.0; https://sites.google.com/site/qingzongtseng/find-focus, downloaded June 2022) with a user-defined variance threshold on the NHS–Alexa647 channel. The same threshold was applied to all images for a given repeat. The selected focus slice was then used for all channels for the given FOV. We illumination-corrected all channels in ImageJ using the BaSiC (version 1.0) plugin^77^, where all focus images from the experimental repeat for the given channel were used to calculate the correction profiles (both flat and dark field) using default settings. The correction profiles were then used to illumination correct the images. We segmented individual cells using CellPose (version 2.0)^78^, using the illumination-corrected NHS–Alexa647 and DAPI as input to define the cell body and nucleus, respectively. We used a custom segmentation model for segmentation generated by transfer learning using the default ‘cyto’ model with default settings as the starting model. Cell diameter was set to 150 pixels. Next, we trained a custom classifier to label cells as either interphase or mitotic using Ilastik (version 1.4)^79^ on the basis of cell morphology and NHS staining, to focus analysis on interphase cells. We then loaded the illumination-corrected channel images and cell segmentation maps into CellProfiler (version 4.1)^80^ and first subtracted the median background (defined as the part of the image not covered by cells) from all channel images. We then measured features describing cell morphology, the mCherry channel and the Golgin97 channel, as well as correlation between Golgin97 and mCherry. The dataset was then filtered to remove mitotic cells, cells on image borders, nonmononucleated cells, cells with touching nuclei (typically a result of missegmentation of multinucleated cell into two cells) and cells where >85% of their cell border contacts other cells, as this typically indicated tight packing of cells, which impaired segmentation accuracy. We then assigned transfection status to the cells on the basis of their cell body mean mCherry intensity and 75% quantile mCherry intensity, where both values had to surpass user-defined thresholds based on the nontransfected cell sample. Lastly, for transfected cells, we removed the brightest cells from the transfected populations on a pairwise basis that reflected which samples would be compared. We defined an outlier threshold for each sample as follows:

$$\begin{array}{l}{\rm{Outlier}}={\rm{mCherry}}{{\_}}{\rm{MeanIntensity}} \\ > {\rm{Population}}\,{\rm{mCherry}}{{\_}}(75 \% {\rm{QuantileIntensity}})\\+1.5\times {\rm{Population}\; {\rm{mCherry}}}{{\_}}{\rm{InterQuantileRange}}\end{array}$$

We then applied the lower threshold for a given isoform to both samples in that pair, resulting in a matched upper intensity range. Notably, this filtering step generally removed very few cells and, thus, only removed the brightest cells. This final step was not performed for the SNX2 mutant experiment as we did not perform pairwise comparisons. Instead, we removed the outliers for each population using the outlier threshold calculated (as above) for the same population.

The nontransfected and transfected cells made up the cells used for downstream analysis.

#### SNX2 cell lines

We analyzed the images as described above but also identified whether the cells expressed SNX2 (similar to the penultimate step used to determine mCherry transfection status). Here, the wells stained without a primary antibody was used to define the intensity thresholds (mean SNX2–Alexa488 intensity and 75% quantile SNX2–Alexa488 intensity) to determine cells expressing and not expressing SNX2. Notably, this was necessary, as not all cells in the rescue lines were expressing SNX2.

#### Representative images

Representative cell images were selected on the basis of our analyses of the overall population behavior of the given mCherry constructs (for isoform imaging and SNX2 mutants) or SNX2 isoforms (for SNX2 cell lines). The cell images were prepared in ImageJ (version 1.54f) using the illumination-corrected channel images. All cell images that were directly compared had the same contrast settings and were specifically chosen to show cells expressing the constructs at similar levels. Mean mCherry intensities for representative cells were determined by manually segmenting the cells, measuring their mean mCherry intensity and then subtracting the mean background mCherry intensity measured adjacent to each representative cell. The values were then normalized to the transfection threshold, giving the mCherry construct amount relative to the effective detection limit. These values directly correspond to the plotted intensity values in the population *XY* plots. The same approach was used to determine SNX2 expression levels in the SNX2 cell lines, although the expression value was normalized to the median intensity observed in the HeLa wild-type population rather than the transfection threshold, which gave us the SNX2 abundance relative to the endogenous level.

### Analysis of cell measurements

#### Classifier-based analysis of protein isoform pairs

We used the single-cell feature measurements to compare localization of mCherry constructs (such as two isoforms from the same gene) in a pairwise manner using a classifier-based approach as outlined in Extended Data Fig. 3a. We performed separate analyses for transfected and nontransfected cells from the same wells. These made up the ‘isoform’ and ‘internal control’ groups, respectively.

The analysis consisted of (1) generating an appropriate dataset through sampling and (2) analyzing the dataset through a classifier-based approach. For (1), we pooled transfected cells expressing the two isoforms to be compared across replicates and then downsampled the cells in the more abundant isoform group to match the number of cells of the less abundant isoform in a replicate-aware manner. Accordingly, replicates could contribute different numbers of cells compared to each other but the cells contributed by a replicate always constituted 50% isoform A and 50% isoform B. Cell numbers used for classifier assessment after downsampling and pooling was ≥170, although most pairs had substantially more cells (median cell number = 967). Cell numbers for each comparison are listed in Supplementary Table 3 The dataset from (1) was then used for classifier-based analysis (2), where the classifier was trained to assign the correct isoform identity (corresponding to well identity) on the basis of the measured features.

The classifier-based analysis was performed in R (version 4.3.3) using Caret (version 6.0-94)^81^ to control classifier training, testing and feature assessment, with the classifier itself being a feedforward neural network, from nnet (version 7.3-19)^82^. The initial input features for the classifier were all features describing the mCherry signal (although intensity features were normalized to mean mCherry intensity) and cell morphology. Zero-variance features were then removed and the remaining features (around 200 for all classifiers) were centered and then scaled, before being used for classifier assessment. For the assessment, the classifier was trained with tenfold cross-validation and we used the average ROC-AUC at the parameters resulting in the best performance across the test folds as the metric describing the degree of isoform differences. The sampling and classifier assessment was repeated 50 times.

To normalize the ROC-AUC metric for potential well-specific effects driving classification, we performed the same analysis on nontransfected cells from the same wells as the two mCherry constructs being compared and used these as internal controls. Notably, the internal controls were sampled to best mirror the isoforms in question and the nontransfected cells were, therefore, sampled to match the proportion of cells contributed by each replicate for the transfected cells, while also contributing the same number of cells from the two wells.

Normalized ROC-AUCs were calculated using the ROC-AUC from the isoform and internal control classifiers in one of two ways, depending on which ROC-AUC had the higher value.

If ${\rm{ROC}}\mbox{-}{\rm{AUC}}_{\rm{isoform}}\ge {\rm{ROC}}\mbox{-}{\rm{AUC}}_{\rm{IC}}$, then ${\mathrm{ROC}}\mathrm{-}{\mathrm{AUC}}_{\mathrm{normalized}}$ was defined as

$${\rm{ROC}}\mbox{-}{\rm{AUC}}_{\rm{normalized}}=\frac{{\rm{ROC}}\mbox{-}{\rm{AUC}}_{\rm{isoform}}-{\rm{ROC}}\mbox{-}{\rm{AUC}}_{\rm{IC}}}{1-{\rm{ROC}}\mbox{-}{\rm{AUC}}_{\rm{IC}}}$$

If ${\mathrm{ROC}}\mathrm{-}{\mathrm{AUC}}_{\mathrm{isoform}} < {\mathrm{ROC}}\mathrm{-}{\mathrm{AUC}}_{\mathrm{IC}}$, then ${\mathrm{ROC}}\mathrm{-}{\mathrm{AUC}}_{\mathrm{normalized}}$ was defined as

$${\mathrm{ROC}}\mathrm{-}{\mathrm{AUC}}_{\mathrm{normalized}}=\frac{\mathrm{ROCA}\mathrm{-}{\mathrm{UC}}_{\mathrm{isoform}}-{\mathrm{ROC}}\mathrm{-}{\mathrm{AUC}}_{\mathrm{IC}}}{1-{\mathrm{ROC}}\mathrm{-}{\mathrm{AUC}}_{\mathrm{isoform}}}$$

where ${\mathrm{ROC}}\mathrm{-}{\mathrm{AUC}}_{\mathrm{isoform}}$ and ${\mathrm{ROC}}\mathrm{-}{\mathrm{AUC}}_{\mathrm{IC}}$ denote the ROC-AUC from the isoform and internal control classifiers, respectively.

The ${\rm{ROC}}\mbox{-}{\rm{AUC}}_{\rm{normalized}}$ metric ranges from −1 to 1 and is symmetric around 0. ${\rm{ROC}}\mbox{-}{\rm{AUC}}_{\rm{normalized}} > 0$ indicates that mCherry construct differences were detectable after accounting for well-specific effects, while $\mathrm{ROC}\mathrm{-}{\mathrm{AUC}}_{\mathrm{normalized}}\le 0$ indicates no detectable differences between the compared constructs after accounting for well-specific effects. We statistically tested whether the normalized ROC-AUC for a given isoform comparison was significantly greater than zero using a one-sided bootstrap approach in R using boot (version 1.3-31)^83^. For each pairwise comparison, we performed 2,000 bootstrap iterations by resampling with replacement from the normalized ROC-AUC values, calculating bootstrap confidence intervals for the mean performance. If zero was excluded from the lower bound of the 99% one-sided confidence interval, the isoform classifier was determined to significantly outperform the internal control classifier, indicating isoform differences. We corrected for multiple testing using the Benjamini–Hochberg method to control the false discovery rate (FDR).

#### Classifier-based analysis after intensity binning

We examined whether our classifier-based analysis yielded consistent results across the mCherry expression range for isoform pairs by separating transfected cells for a given isoform pair into lower-intensity and higher-intensity groups and then performed the classifier-based analysis as described above separately on the groups. Cells were grouped using two separate approaches as outlined in Extended Data Fig. 5a. In approach 1, we identified the maximum single-cell mean mCherry intensity for both isoforms in a pair, selected the lower of the two and assigned cells expressing either isoform into bins as follows: lower bin = mean mCherry intensity < 20% of the maximum; higher bin = mean mCherry intensity > 20% of the maximum. In approach 2, we identified the median single-cell mean mCherry intensity for both isoforms in a pair, selected the lower of the two and assigned cells expressing either isoform into to bins as follows: lower bin = mean mCherry intensity < median; higher bin = mean mCherry intensity > median.

We compared the classifier results for all isoform pairs with and without binning by plotting the normalized ROC-AUC in R (version 4.3.3) using pheatmap (version 1.0.12)^84^. We compared the population mCherry level for all isoform pairs with and without binning by plotting the population median of mean mCherry intensities, relative to the transfection threshold.

#### Classifier-based analysis of untagged SNX2 isoforms in HeLa SNX2-KO rescues

We examined whether the untagged SNX2 isoforms were identified as differentially localizing by our classifier-based approach by performing the classifier-based analysis described above on the SNX2 signal feature measurements from HeLa SNX2 rescue cells expressing either untagged SNX2−EX or SNX2+EX. We performed the analysis using cells expressing the SNX2 isoforms at two different levels: an endogenous-like level, consisting of cells positively expressing SNX2 in the images with a mean SNX2 intensity ≤ 1.5× that of the wild-type HeLa population mean SNX2 intensity, and a full-expression range, consisting of all cells positively expressing SNX2 in the images. This resulted in *n* = 418 cells per isoform for the endogenous-like level and *n* = 763 cells per isoform for the full-expression range after downsampling. As an internal normalization control, we used the SNX2-negative cells present in the stable populations. These were defined as SNX-negative cells with mean SNX2 intensities ≤ 0.27× that of the wild-type HeLa population mean SNX2 intensity. This threshold was chosen such that ≥99% of HeLa SNX2-KO cells stained with the anti-SNX2 antibody fell into the negative population.

#### Out-of-sample testing

We assessed out-of-sample performance of classifiers trained on controls and select isoform pairs using cells from one batch (batch A or B) for the training set and cells from the other batch for testing. For the training set, we sampled and trained the classifier as described above. For the test set, we sampled the cells as described above to have the same number of cells for the two groups and then predicted isoform identity for the cells using Caret^81^ predict(). The performance was assessed as accuracy. Notably, we sampled the test set to have equal classes to both mirror the training set and make the accuracy comparable between conditions (as accuracy is sensitive to class imbalance). The accuracy can, therefore, range from 0.5 to 1.0. In total, 20 iterations of classifier training and testing were performed and the performance values were the mean accuracy from each iteration. For each iteration, a classifier was trained on the training batch and then used to predict isoform identities for the downsampled test batch, where the test batch downsampling and prediction was performed 50 times. The mean accuracy across the 50 times was used as the performance.

To examine the association between the accuracy of the out-of-sample tests and the normalized ROC-AUC approach, we performed linear regression, where the accuracy was proportional to the normalized ROC-AUC. We statistically tested whether the accuracy and normalized ROC-AUC were correlated in R using a correlation test, cor(method = ‘pearson’).

#### Replicate sampling imbalance

We quantified replicate sampling imbalance as the maximum fraction of cells used in given isoform pair comparison that came from a single experimental repeat over the total number of cells used for the comparison (cells pooled across all repeats).

#### mCherry intensity imbalance

We quantified mCherry intensity (expression) imbalance between isoforms in a pair by first calculating the population median of single-cell mean mCherry intensities for each isoform of the cells used for classifier analysis. We then determined the population difference as follows: intensity imbalance = |log_2_ ((median (+EXON))/(median (−EXON)))|. This was calculated for each classifier iteration as cell sampling was part of each classifier iteration and the final isoform imbalance score was defined as the median of the intensity imbalance scores for the given isoform pair.

#### Association between imbalance and performance

The correlation between normalized ROC-AUC and imbalance metrics (replicate sampling imbalance, mCherry intensity imbalance) was statistically examined in R using a correlation test, cor(method = ‘kendall’).

#### Identifying Isoform-distinguishing features

Using the default classifier importance scores generated by Caret for the nnet classifiers, we first calculated the feature contribution percentage (%), which specified how large a percentage of the sum of feature importance scores was contributed by a given feature:

$$\mathrm{Feature}\,\mathrm{contribution}\,( \% )=\frac{{\mathrm{Importance}\,\mathrm{score}}_{{\mathrm{feature}}_{{i}}}}{{\sum }_{i=1}^{{n}_{\mathrm{features}}}{\mathrm{Importance}\,\mathrm{score}}_{{\mathrm{feature}}_{i}}}\times 100$$

where *n* features (number of features used for the given classifier) ≈ 200 for all classifiers.

We did this for both the isoform classifier and the associated internal control, which allowed us to calculate contribution ratios, specifying the relative contribution of a feature to the isoform classifier compared to the internal control:

$${\rm{Contribution\; ratio}}=\frac{{\rm{Feature\; contribution}}_{\rm{isoform}}}{{\rm{Feature\; contribution}}_{\rm{IC}}}$$

We then defined isoform-distinguishing features (features that were notably more important for the isoform classifier compared to the associated internal control classifier) as features with a contribution ratio and a contribution ratio multiplied with the feature contribution percentage for the isoform classifier above two user-defined threshold values.

$$\begin{array}{l}{\mathrm{Noteworthy}}\,{\mathrm{feature}}={\mathrm{Contribution}}\,{\mathrm{ratio}}\\ > {\mathrm{threshold}}\,{\mathrm{ratio}}\,{\mathrm{and}}\,{\mathrm{contribution}}\,{\mathrm{ratio}}\\\times {\mathrm{feature}}\,{\mathrm{contribution}}\,( \% )\,{\mathrm{isoform}}\\ > {\mathrm{threshold}}\,({\mathrm{ratio}}\,\times \,{\mathrm{contribution}})\end{array}$$

The user-defined threshold values were defined such that the negative control had no distinguishing features. The contribution ratio threshold was 2.6 and the ratio × contribution threshold was 1.5.

#### PCA of SNX2 mutants

We analyzed the SNX2 mutant constructs by PCA in R.

Starting with the image-derived measurements of the SNX2 isoform-distinguishing features (~20) for all SNX2 construct-positive cells, we first reduced the number of input features by removing highly correlated features to improve interpretability and diminish the impact of a specific localization behavior caught by multiple features on the PCA. To do this, we calculated the pairwise Pearson correlation (*r*) between the features using the measurements from all SNX2 construct-positive cells (without grouping cells according to construct). The cells were pooled from three experimental repeats with *n* cells ≥ 168 expressing each construct (cell numbers for each construct listed in Extended Data Fig. 8b).

With the Pearson correlation values, we created a distance matrix (1 − *r*), which we visualized as a correlation heat map and used for hierarchical clustering of the features. This analysis revealed many features were highly correlated and we considered there to be seven main feature groups. Therefore, we reduced features with a Pearson correlation ≥ 0.7 to the single feature that showed the greatest variance across the cells, resulting in seven retained features. We centered and scaled the measurements of the retained features and then performed PCA using prcomp() for all SNX2 construct-positive cells. With the PCA output, we created centroid representations for each construct by extracting the PC scores for each cell, grouping the cells according to SNX2 construct and then calculating the mean PC score across the cells for each PC. We used the centroid PC scores from the first five PCs (capturing 98.9% of the variance) to cluster the constructs into two clusters by hierarchical clustering using Euclidean distance and Ward’s D2 linkage. We then plotted the centroid PC scores on our PCA plots, showing PC1/2, as well as the assigned cluster for each construct. From the PCA, we also generated biplots and scree plots, both of which were calculated using the full cell dataset (not centroids). The plots were created using the using the factoextra and tidyverse packages.

We performed the analysis above using transfected cells and then performed the PCA separately for nontransfected cells using the retained features identified using the transfected cells, as a way of assessing construct-specific effects versus batch effects.

### AP–MS

We examined the protein interactomes of the SNX2 isoforms using AP–MS on HeLa wild-type and HeLa SNX2 cell lines (KO and rescue lines). The experiment was repeated three times, although only two replicates were used for interaction analysis, as described below.

#### SNX2 immunoprecipitation

The immunoprecipitation protocol was based on a previous study^85^ with minor modifications. The HeLa cell lines were grown to 80–90% confluency in P150 dishes in supplemented DMEM with Pen/Strep. The cells were then washed twice in ice-cold 1× PBS, after which the cells were detached by cell scraping in 1 ml of 1× PBS. The detached cells were transferred to Eppendorf tubes and pelleted by centrifugation at 800*g* for 5 min at 4 °C. The supernatant was then removed and the cell mass was weighed and then snap-frozen in liquid nitrogen. The cell pellets were stored at −70 °C until the immunoprecipitation was continued (within 1 week of harvest). To immunoprecipitate SNX2 and interactors, the cell pellets were thawed on ice and resuspended in immunoprecipitation buffer (20 mM HEPES pH 7.2, 100 mM potassium acetate, 2.5 mM magnesium acetate, 0.1% Nonidet-P40 (NP40) (v/v), 1 mM DTT and 10% glycerol (v/v)) at a ratio of 4 µl per mg of pellet by gentle pipetting. The cell suspensions were then frozen on dry ice and thawed by holding the tubes one time to promote cell lysis (freeze–thaw). The lysates were then transferred to a tube spinner and spun at 12 rpm for 30 min at 4 °C. The cell lysates were then transferred to a 2-ml Dounce homogenizer for mechanical shearing (25 strokes). Homogenized lysates were then transferred to Eppendorf tubes and spun at 17,000*g* for 20 min at 4 °C to pellet nuclei. The supernatant was recovered and transferred to a new Eppendorf tube. Next, 15 µl of the isolated supernatant was transferred to a separate Eppendorf tube and served as a measure of the input fraction for western blotting (kept at −20 °C until use). The remaining supernatant was mixed with 5 µl of anti-SNX2 antibody (corresponding to 1.25 µg of antibody) and incubated on a tube spinner at 7 rpm overnight at 4 °C. The next day, the antibody–lysate mix was mixed with 50 µl of immunoprecipitation buffer-equilibrated protein G Dynabeads (Thermo Scientific) and the bead lysate suspension was then incubated on a tube spinner at 7 rpm for 2 h at 4 °C. After this incubation, the suspension was put on a magnetic rack to pull down the bead–antigen–antibody complexes. The supernatant was removed and transferred to a separate tube that was frozen at −20 °C (it served as the unbound fraction for western blotting). The beads were then resuspended in 1 ml of immunoprecipitation buffer and transferred to a new tube. The tubes were then placed on the magnetic rack again and the supernatant was removed. The beads where then resuspended in 1 ml of wash buffer (20 mM HEPES pH 7.2 and 10 mM MgCl_2_) by gentle pipetting and placed on the magnet a final time and the supernatant was removed. The sample was then eluted from the beads by adding 30 µl of 4× LDS sample buffer diluted to 1× in 1× PBS with 20 mM DTT. The sample was then incubated at 95 °C for 5 min, after which it was put back in magnetic rack to isolate the eluate from the beads. Next, 5 µl of the eluate was kept as the immunoprecipitated fraction for western blotting and the remaining 25 µl was used for MS analysis. Eluted samples were kept at −20 °C until use (within 1 week).

#### Downstream sample processing and MS acquisition

The IP eluates were first reduced with 5 mM DTT at 37 °C for 30 min, followed by alkylation with 10 mM iodoacetamide at room temperature in the dark for 30 min. Protein digestion was then performed using the SP3-based tryptic workflow^86^. Digested samples were desalted using two SDB-RPS disks (Empore, Sigma, 66886-U) packed into a 200-μl pipette tip as previously described^87^. Extracted peptides from each cleanup were reconstituted in 10 μl of 0.1% (v/v) formic acid, 0.05% (v/v) heptafluorobutyric acid and 2% acetonitrile in water.

Digest peptides were separated by nanoLC using an Ultimate nanoRSLC UPLC and autosampler system (Dionex). Samples (2.5 µl) were concentrated and desalted onto a micro C18 precolumn (300 µm × 5 mm; Dionex) with 98:2 water and acetonitrile + 0.1% TFA at 15 µl min^−1^. After a 4-min wash, the precolumn was switched (Valco 10 port UPLC valve, Valco) into line with a fritless nano column (75 µm × ~30 cm) containing C18AQ medium (1.9 µm, 120 Å, Dr Maisch). Peptides were eluted using a linear gradient of 98:2 water and acetonitrile + 0.1% formic acid to 64:36 water and acetonitrile + 0.1% formic acid at 200 nl min^−1^ over 90 min. High voltage (2,000V ) was applied to low-volume titanium union (Valco), with the tip positioned ~0.5 cm from the heated capillary (*T* = 275 °C) of a Orbitrap Fusion Lumos (Thermo Electron) MS instrument. Positive ions were generated by electrospray and the Fusion Lumos operated in data-dependent acquisition mode.

A survey scan of *m*/*z* 350–1,750 was acquired in the orbitrap (resolution = 120,000 at *m*/*z* 200, with an accumulation target value of 400,000 ions) and lockmass enabled (*m*/*z* 445.12003). Data-dependent tandem MS analysis was performed using a top-speed approach (cycle time of 2 s). MS2 spectra were fragmented by higher-energy collision-induced dissociation (normalized collision energy = 30) activation mode and the ion trap was selected as the mass analyzer. The intensity threshold for fragmentation was set to 25,000. A dynamic exclusion of 20 s was applied with a mass tolerance of 10 ppm.

The above steps were performed by the Bioanalytical MS Facility at the University of New South Wales.

#### MS data processing

The raw MS data were processed using Mascot version 2.8. Peak lists were generated using Mascot Daemon/Mascot Distiller (Matrix Science) using default parameters and submitted to the database search program Mascot (version 2.8; Matrix Science). Search parameters were as follows: precursor tolerance, 4 ppm; product ion tolerances, ±0.5 Da; oxidation of methionine and carboxyamidomethylation of cysteine as variable modifications; enzyme specificity, trypsin; two missed cleavages; UniProt database (February 2021).

The MS data were deposited to the PRIDE database (PXD074758).

#### Protein interactome analysis using SAINT

SAINTexpress (version 3.6.3)^88^ was used to identify high-confidence protein interactors in the SNX2-positive lines (HeLa wild type, HeLa rescue SNX2+EX, HeLa rescue SNX2−EX), while the HeLa SNX2-KO line was used to define the background. Raw spectral counts were extracted from the Mascot search results using the number of significant peptide matches per protein (prot_matches_sig, significance threshold *P* ≤ 0.05), with a minimum requirement of two unique peptide sequences (prot_sequences) for a protein to be considered a hit in a given sample (Supplementary Table 7). While three biological replicates were prepared for all cell lines, replicate 3 was excluded from the SAINT analysis^89^ for all SNX2-positive samples because of systematically lower total spectral counts compared to replicates 1 and 2. Note, all replicates were retained for HeLa SNX2-KO cells, which was used to model unspecific binding and used for control subtraction before abundance scoring in the SNX2-positive lines. Candidate interactors were filtered using a Bayesian FDR (BFDR) threshold of ≤0.01 as the primary cutoff, with a secondary threshold of ≤0.05. Protein abundance was reported as the average spectral count (AvgSpec) across replicates (Supplementary Table 8), capped at 50 for visualization purposes.

#### LUMIER

Luminescence-based mammalian interactome mapping (LUMIER)^90^ was performed to examine binary PPIs as described previously^70^, with slight modifications. Briefly, HEK293T wild-type cells were passaged as described above and seeded into wells in a 48-well plate (TC-treated, noncoated polystyrene) at 40,000 cells per well in 200 µl of supplemented DMEM. The cells were incubated for 24 h and then cotransfected with 200 ng of total plasmid DNA (100 ng of prey plasmid and 100 ng of bait plasmid) for the various interactions tested. The transfection was performed using Lipofectamine 3000 (1.5:1 ratio of reagent to DNA, 2:1 ratio of P3000 to DNA) with 20 µl of transfection mixture made up in OPTI-MEM being dripped into each well and mixed with the medium by gentle pipetting. Every transfection combination was performed in triplicate in each experiment and these wells were subsequently handled in parallel and used as technical replicates (observations from same experiment repeat). The cells were incubated for 48 h with the transfection medium and each well was then washed in once in 1× PBS and lysed in 150 µl of LUMIER lysis buffer (50 mM Tris-HCl pH 7.4, 150 mM NaCl, 1 mM tetrasodium EDTA, 0.5% Triton X-100 (v/v) and Halt protease and phosphatase inhibitor, added right before use). The wells were incubated with the lysis buffer for 40 min at 4 °C under constant, gentle agitation on an orbital shaker. Next, 90 µl of each lysate was transferred to a Lumitrac 96-well plate precoated with anti-FLAG antibody, as described below. This served as the IP plate. Another 20 µl of each lysate was transferred to an uncoated Lumitrac 96-well plate. This served as the TOTALs plate. Both plates were sealed with sealing tape and incubated for 60 min at 4 °C without agitation. The lysate was then removed from the IP plates by aspiration and the IP plate was washed six times in 250 µl of LUMIER wash buffer (50 mM Tris-HCl pH 7.4, 150 mM NaCl, 1 mM tetrasodium EDTA and 0.1% Triton X-100 (v/v)). The wash buffer was then removed and 100 µl of *Renilla*-GLO reagent (Promega) (made fresh according to manufacturer’s instructions) was added to each well. Right after, 20 µl of *Renilla*-GLO reagent was added to each well in the TOTALs plate. Both plates were incubated for 10 min at room temperature with the reagent and the luminescence was then read on a CLARIOstar plate reader (BMG). The IP plate was then used to measure the amount of prey protein by ELISA as follows. Each well was washed eight times in 250 µl of PBS-T (1× PBS and 0.05% Tween-20 (v/v)). The PBS-T was then removed and 100 µl of LUMIER anti-FLAG–HRP (horseradish peroxidase) antibody solution (1× PBS, 1% FBS (v/v) and 5% Tween-20 (v/v) with anti-FLAG–HRP (0.05 µg ml^−1^). Antibody added fresh) was added to each well. The plate was then sealed and incubated for 30 min at 4 °C without agitation. The wells were then washed another eight times in 250 µl of PBS-T. The PBS-T was removed and 100 µl of ELISA Pico substrate (made fresh) was added to each well. The plate was incubated for 7 min at room temperature and the absorbance was then read on a CLARIOstar plate reader (BMG). The experiment was repeated 3–4 times for all examined interactions.

#### LUMIER data analysis

The LUMIER interaction scores, NLIR and NtFLIR, were calculated using luminescence measurements of the TOTALs and IP plate and IP ELISA as described previously^70^. NLIR describes the luminescence signal in the IP plate divided by luminescence in the TOTAL plate. NLIR > 3 was considered a positive interaction^70^. NtFLIR was calculated as the NLIR score divided by the absorbance measured in the ELISA against FLAG (which quantifies the prey amount) and served as an interaction score (NLIR) normalized to the amount of prey protein present.

For each experimental repeat, we used the mean NLIR and NtFLIR across the technical replicates. We calculated the interaction differences between SNX2 isoforms with a given prey protein as: log_2_((NtFLIR (SNX2+EXON))/(NtFLIR (SNX2−EXON))), which we calculated for each experimental repeat. The SNX2−EX isoform was used as the reference for the SNX2+EX isoform, as well as for all SNX2 mutants, where interaction differences were calculated in the same way. We statistically tested whether the SNX2+EX isoform or SNX2 mutants interacted differently with prey proteins compared to SNX2−EX using two-sided one-sample *t*-tests on the interaction difference score, where the interaction differences for the different SNX2 constructs were compared to zero. We corrected for multiple testing using the Benjamini–Hochberg method to control the FDR.

#### Anti-FLAG plate coating

Lumitrac 96-well plates were coated with anti-FLAG antibody by incubating each well with 100 µl of 1× PBS with anti-FLAG antibody (10 µg ml^−1^) overnight at 4 °C. The next day, the antibody solution was removed and 250 µl of LUMIER blocking buffer (1× PBS, 3% BSA (w/v), 5% sucrose (w/v) and 0.5% Tween-20 (v/v)) was added to each well and incubated at 60 min at room temperature with gentle agitation on orbital shaker. The blocking buffer was then removed and the plate was sealed and kept at 4 °C until use.

#### Western blots

Western blots were performed using standard protocols. Briefly, all protein samples were run on Bolt Bis–Tris 8% gels (Invitrogen) using 10–30 µg input protein per sample (determined through a Bradford assay according to the manufacturer’s protocol) or, for immunoprecipitated samples, a specific percentage of the isolated fractions for (1/150 input and unbound and 1/3 immunoprecipitated sample). After gel electrophoresis, the proteins were transferred to methanol-activated PVDF membranes (Immobilon). The membranes were then blocked with TBS-T (1× TBS and 0.1% Tween-20 (v/v)) and 6% skim milk powder (w/v) for 1 h under constant, gentle agitation on orbital shaker. The membranes were then briefly washed in TBS-T and the membranes were then transferred to small plastic bags and covered with primary antibody solution, consisting of TBS-T, 1% BSA (w/v) and the primary antibody (concentrations below). Note, blots were typically cut into pieces to probe different parts of the membrane for specific proteins (based on size) after blocking but before primary antibody incubation. The membranes were incubated with the primary antibody solution at 4 °C overnight under constant agitation. The next day, the membranes were washed three times for 10 min at room temperature in TBS-T under constant gentle agitation. Then, the membranes were incubated with HRP-conjugated secondary antibody solution, consisting of TBS-T, 1% skim milk powder and secondary antibody–HRP raised against the host species of the primary antibody (details below), for 1 h at room temperature under constant agitation. This was also conducted in plastic bags. The membranes were then washed again three times for 10 min each at room temperature with TBS-T under constant gentle agitation. The membranes were then developed with Clarity Western ECL substrate (BioRad) according to the manufacturer’s instructions and imaged on a ChemiDoc.

To quantify the amount of SNX2 protein, the intensity of all bands of interest were measured in ImageJ (version 1.54), after which the background was subtracted. The amount was then normalized to the amount of GAPDH (reference gene) from the same sample, measured in the same way as SNX2.

#### SNX2 cell line verification blots

To verify KO or transgene expression, cells from HeLa wild-type, SNX2-KO and SNX2 rescue cell lines were washed in 1× PBS and then lysed in RIPA buffer (150 mM NaCl, 1% nonylphenol ethoxylated (v/v), 0.5% sodium deoxycholate (w/v), 0.1% SDS (w/v), 17.1 mM EDTA and 50 mM Tris-HCl pH 8) on ice for 5 min. The lysates where then transferred to Eppendorf tubes and spun at 17,000*g* for 10 min at 4 °C; the supernatant was isolated and used for western blots. Cell lysates were mixed with LDS sample buffer (Thermo Scientific) and DTT for final concentrations of 1× and 20 mM, respectively, and examined by western blotting as described above. The primary antibodies were mouse anti-SNX2 used at 0.25 µg ml^−1^ (1:1,000) and mouse anti-GAPDH used at 0.04 µg ml^−1^ (1:5,000). The secondary antibody was goat anti-mouse–HRP, used at 0.16 µg ml^−1^ (1:5,000). The experiment was performed once.

#### SNX2 immunoprecipitation verification blots

The three isolated fractions (input, unbound and immunoprecipitated) were diluted in 1× PBS; for the input fraction and the unbound fraction, this was 1/150, whereas, for the immunoprecipitated fraction, this was 1/3. The diluted samples were then prepared in 4× LDS sample buffer and 20 mM DTT and examined by western blotting as described above. The primary antibodies were mouse anti-SNX2 used at 0.25 µg ml^−1^ (1:1,000) and mouse anti-GAPDH used at 0.04 µg ml^−1^ (1:5,000). The secondary antibody was goat anti-mouse-HRP, used at 0.16 µg ml^−1^ (1:5,000). The experiment was performed once.

#### Endpoint RT–PCR

Endpoint RT–PCR was carried out to examine inclusion of SNX2’s microexon to verify correct isoform expression in HeLa SNX2 stable cell lines. We isolated RNA for the analysis from the HeLa SNX2 cell lines by washing adherent cells twice in 1× PBS and then lysing them in TRIzol reagent (Thermo Scientific). Total RNA was subsequently purified according to the manufacturer’s protocol for TRIzol-based RNA extraction. The precipitated RNA was redissolved in RNase-free H_2_O. The RT and subsequent PCR amplification was performed as a single step using Qiagen OneStep RT–PCR kit. The reactions were set up in a total volume of 10 µl, as described previously^91^. Briefly, each 10-µl reaction consisted of 3.7 µl of RNase-free H_2_O, 2 ml of 5× Qiagen OneStep RT–PCR buffer, 1.2 µl of 5 µM forward primer, 1.2 µl of 5 µm reverse primer, 0.4 µl of 10 mM dNTP mix, 0.5 µl of enzyme mix, 1 µl of RNA template (10–30 ng µl^−1^) in RNase-free H_2_O. The reactions were run in a Thermocycler with the following steps: (1) RT, 30 min at 50 °C; (2) initial PCR activation, 15 min at 95 °C; (3) three-step cycling (40 cycles), denaturation for 1 min at 94 °C, annealing for 1 min at 54 °C and extension for 1 min at 72 °C; (4) final extension, 10 min at 72 °C. The reactions were loaded on 2% agarose gels containing GelRed (Biotium), separated by gel electrophoresis and imaged on a ChemiDoc to examine RT–PCR product sizes. Size differences (96 nt, 108 nt) corresponding to the size of the microexon (12 nt) indicated which isoform was expressed. The primers used to examine SNX2’s microexon are listed in Supplementary Table 11. The experiment was performed once. Primers were obtained from Integrated DNA Technologies.

#### Correlation between normalized ROC-AUC and LUMIER interactions

We examined the correlation between normalized ROC-AUC (localization readout) and the overall PPI differences as quantified by LUMIER for isoform pairs where ≤3 interactions were tested across ≤2 experimental repeats and resulted in positive interactions with at least one isoform of the pair. The LUMIER data originated from publications by the same authors^12^ and can be found in Supplementary Table 5.

We calculated overall PPI differences between two isoforms from the LUMIER data by calculating the fold change in NLIR (NLIR_FC_) between isoforms for a given interactor for each repeat:

$${\mathrm{NLIR}}_{\mathrm{FC}}=\frac{{\mathrm{NLIR}}_{\mathrm{isoform}+{\mathrm{EXON}},{\mathrm{r}}}}{{\mathrm{NLIR}}_{\mathrm{isoform}-{\mathrm{EXON}},{\mathrm{r}}}}$$

Then, the NLIR_FC_ for all experimental repeats was averaged and the absolute log_2_ fold change was calculated for the averaged value:

$$|{\mathrm{log}}_{2}(\mathrm{mean}({\mathrm{NLIR}}_{\mathrm{FC},r})|$$

This score was calculated for each interactor that was examined for an isoform pair and we used the mean of these interaction difference scores as a measure of the overall PPI difference for an isoform pair. For the localization readout, we used the median normalized ROC-AUC. We statistically tested the correlation between the median normalized ROC-AUC and collective LUMIER interaction score in R using a correlation test, cor(method = ‘pearson<). A *P* value ≤ 0.05 was considered significant.

#### Analysis of microexons in α-helices

We extracted microexons (nucleotide length < 30 nt) from vastDB (version 11)^30^ and filtered them to only include alternatively spliced microexons showing tissue-specific regulation. We specifically used microexons that were differentially included in neural tissues compared to nonneural tissues (using vast-tools compare) and with a PSI > 15 in neurally annotated adult tissues in the vastDB resource. This captured most tissue-regulated microexons^29^.

The localization of microexons within secondary protein structures was assessed using DSSP (database of secondary structure) assignments on the basis of protein entities in the Protein Data Bank^92^. Here, we defined α-helices as annotated by DSSP as H (α-helix) or G (3_10_-helix), β-strands as B (isolated β-bridge) or E (extended β-strand) and loops as S (bend) or T (hydrogen-bonded turn). Microexons were considered within a secondary-structure element if any of its residues (or the immediately preceding or proceeding residue) was annotated as a secondary-structure element by DSSP. The relative position of the microexon within α-helices was defined on the basis of the minimum distance from microexon to the beginning or end of the helix as defined by DSSP.

We analyzed the residue composition of microexons and flanking residues compared to those of larger cassette exons (≥30 nt) that followed a similar inclusion pattern to that of the microexons, as described above. Only exon lengths divisible by 3 were included. Residues were grouped as follows: aliphatic residues, I, L and V; charged residues, E, K, D and R; helix breaking, P and G; helix forming, L, I, F, E, Y, W and M; aromatic, F, Y, W and H. Residue proportions within exons and in the flanking regions were defined by counts. The flanking regions were defined as residues 30 aa upstream and downstream of the inserted exon. Enrichment was calculated as log_2_((Freq. microexon)/(Freq. longer exon)) and statistically examined using the Fisher exact test. Residue analysis of microexons overall, as well as microexon subsets (microexons in helices, microexons in secondary structure), was compared to the longer neural cassette overall.

#### Protein structural modeling

Protein structure predictions were obtained from two sources. For UniProt-annotated protein sequences with predictions available through the AlphaFold Protein Structure Database^47^, we used the corresponding AlphaFold Protein Structure Database models. Sequences for human TEAD1 and ERC1 protein isoforms were extracted from the UniProt database^23^ (TEAD1, P28347-1; ERC1, Q8IUD2-1).

For isoforms or exon-containing protein sequences not represented in UniProt annotation and, therefore, not directly available through the AlphaFold Protein Structure Database, we generated custom structure predictions using the Google Colab implementation of AlphaFold2. For SNX2 microexons, we extracted sequences for SNX2 protein isoforms from the UniProt database^23^ for human (O60749-1), rat (B2RYP4), chicken (A0A8V0X9Z2) and zebrafish (Q32LT0). Amino acid sequences corresponding to the microexons of interest and specific amino acid substitutions were then added or removed manually. The proteins were then modeled using AlphaFold2 (version 2.1) with the default input settings through Google Colab^93^. The isoform models were downloaded and visualized in ChimeraX (version 1.9)^94^. ChimeraX was also used to align residues, examine surface charge and examine electrostatic interactions (only salt bridges) of the SNX2 region corresponding to 419–464 of human SNX2 (UniProt O60749-1) with default input settings.

#### Annotation of the post-translation modification status of the alternative regions within imaged isoforms

We annotated the post-translation modification status within and immediately around (within two residues on either side) the isoform-specific regions for the imaged isoforms by cross-referencing PhosphoSitePlus^32^ (accessed March 2026). We considered a PTM detected in the region if it was identified in a single study. The annotations are included in Supplementary Table 2.

#### Annotation of the structural region affected among the imaged isoforms

We assessed the type of structural region affected by splicing events in the imaged isoform library by examining the predicted structure of the region in the AlphaFold Protein Structure Database^47^ (accessed June 2025). We based our structural annotation on the structure of the immediately flanking region to where the additional residues would be inserted and, if the alternative region was present in the model, how it was predicted to affect the structure. The annotations are included in Supplementary Table 2.

#### Amino acid conservation analysis of the SNX2 microexon

All isoforms of SNX2 annotated on the UniProt server^23^ were identified and isoforms containing the microexon and of canonical size (~500 aa) were extracted. Isoforms were further manually filtered to ensure no evolutionary clade was overrepresented. Sequences were aligned using Clustal Omega (version 1.2.4)^95^ using default parameters. JalView was then used to visualize sequences^96^. Secondary structure was predicted using JPred secondary-structure prediction (Lupas_14) and JNetPRED, buried residues were predicted using Jnet burial prediction of solvent accessibility and conservation using JNETConf^96^.

### Construction of the interface-resolved PPI network

PPIs were mapped to physical contact interfaces using curated evidence from UniProt^23^, Interactome3D^26^, 3DID^27^, Domino^24^, IntAct^25^ and ELM^28^. Records from all sources were harmonized to a common coordinate system by mapping proteins to UniProt canonical accessions and residue numbering. Redundant records describing the same interaction interface were merged while preserving source provenance and annotation type. The resulting interface-resolved interactome represents each edge as a specific annotated contact between two proteins (node), rather than a binary protein association alone, with mapped interface residues or residue intervals recorded for both partners where available. Note, Interactome3D and 3DID were grouped and referred to as ‘3DID‘ as they were created by the same group.

### Definition of AS-driven interface alteration

AS events were projected from transcript coordinates to protein coordinates. An event was classified as interface altering when the protein region encoded by the regulated exon overlapped at least one annotated interface residue or mapped interface interval for a given interaction. Primary analyses used a permissive binary criterion of any overlap of at least one residue, following the results of prior AS interactome studies. In addition, overlap extent was quantified using fractional overlap metrics, including the proportion of interface residues overlapped by the exon and the proportion of exon-encoded residues overlapping the interface.

#### Identification of AS events

Vast-tools compare was used to analyze AS from 75 tissue and cell types (https://vastdb.crg.eu/wiki/Downloads)^30^ using default setting except --min_dPSI 20 with all combinations of splicing events assessed. Only differential events annotated as exon-skipping events (S, C1, C2 and C3) whose length was divisible by 3 (that is, frame-preserving) and identified as differential between multiple (≥2) tissues were considered in the interface-resolved interactome.

#### Integration of AS data

For each AS exon, Shannon entropy (*H*) and the Gini index (*G*) were calculated from the PSI distribution across tissues:

$${p}_{i}=\frac{{\rm{PSI}}_{i}}{{\sum }_{j=1}^{c}{\rm{PSI}}_{j}}$$

where ${\rm{PSI}}_{i}$ is the PSI of the exon in tissue *i* and *c* is the number of tissues.

Shannon entropy was then calculated as follows:

$$H=-\mathop{\sum }\limits_{i=1}^{c}{p}_{i}\log 2({p}_{i})$$

The Gini index was calculated as follows:

$$G=1-\mathop{\sum }\limits_{i=1}^{c}{p}_{i\,}^{2}$$

High-entropy and high-Gini exons correspond to tissue-specific splicing^97^, while low values reflect constitutive inclusion. For each in-frame exon, its corresponding amino acids in the canonical ensembl protein transcript^98^ were identified. For the interface interactome, only those splicing events overlapping an annotated protein interface(s) were retained. Interactions regulated by these interfaces were considered AS-regulated edges. AS events were annotated with additional information on the basis of whether they overlapped known Pfam domains^31^, disordered regions^99^, domain varieties^31^ (RNA, DNA, protein or lipid), phosphorylation sites^32^ and UniProt annotations^23^.

#### Percolation analysis

To evaluate how AS reshapes interaction topology at the systems level, we performed percolation analysis on the interface-resolved interactome. Each edge in this network corresponds to a PPI interface. AS-regulated edges were ranked by exon tissue specificity, such that the most strongly tissue-regulated exons were removed first and progressively less tissue-specific exons were removed later. For each removal fraction *f*, corresponding to removal of the top ranked AS-regulated edges, we recalculated network properties on the residual graph *H*_*f*_.

We quantified two complementary network properties. First, we calculated *S*(*f*), the fraction of nodes remaining in the main connected part of the interactome:

$$S(f)=\frac{{|L}({H}_{f})|}{{|V}({H}_{f})|}$$

$$S(f)=|L({H}_{f}){{|}}/|V({H}_{f}){{|}}$$

where $L({H}_{f})$ denotes the largest connected component of the residual graph ${H}_{f}$ and ${|V}({H}_{f})|$ is the total number of nodes in ${H}_{f}$. Thus, S(f) measures the extent to which the interactome remains largely intact, rather than breaking into smaller disconnected pieces, and is a standard measure of robustness under progressive perturbation^100^.

Second, we calculated *E*(*f*), the approximate global efficiency of the residual graph, following the approach introduced by Latora and Marchiori^101^, which measures how efficiently proteins can still reach one another through short paths after edge removal. Because exact all-pairs global efficiency is computationally expensive for repeated percolation analyses, we estimated this quantity by averaging inverse shortest-path distances over a sampled set of source nodes at each removal step. Let *Q*_*f*_ denote the set of source nodes used at step *f* (all nodes if the graph contained ≤200 nodes; otherwise, a random sample of 200 source nodes) and let *d*_(*Hf*}(*u*,*v*)_ denote the shortest-path distance between nodes *u* and *v* in *H*_*f*_. Approximate global efficiency was calculated as follows:

$$E(f)=\frac{1}{{R}_{f}}\sum _{u\in {Q}_{f}}\sum _{\begin{array}{c}\upsilon \in V({H}_{f})\\ \upsilon \ne u,{d}_{{H}_{f}(u,\upsilon ) < \infty }\end{array}}\frac{1}{{d}_{{H}_{f}(u,\upsilon )}}$$

where *R*_*f*_ is the number of reachable ordered source–target pairs included in the calculation and *Q*_*f*_ is the sampled set of source nodes. Lower values of *E*(*f*) indicate that short communication paths are being lost, even if many proteins remain in the same main connected part of the network.

To assess whether the observed percolation trajectory differed from random expectation, we generated 1,000 null trajectories. In the degree-matched null model used here, all network edges were first grouped into bins according to the minimum degree of their two endpoint nodes in the original graph. For each null replicate and each removal step *k*, we randomly removed edges from these degree bins in proportion to the number of observed AS-regulated edges removed at that step. This produced null removal sets matched to the endpoint degree profile of the observed removal set while preserving the total number of removed edges at each step.

For each metric ${\rm{M}}\mathrm{in}(S,E)$ the observed trajectory *M*_AS(*f*)_ was compared to the null distribution $f{{({M}_{(\mathrm{null},r)}(f))}_{(r=1)}}^{1,000}$ at the same removal fractions. We summarized the null distribution at each *f* by its mean and 95% interval (2.5th–97.5th percentiles). We also calculated paired deviations for each replicate as follows:

$${\Delta }_{r}(f)={M}_{\rm{AS}}(f)-{M}_{{\rm{null}},r}(f)$$

and plotted the mean and 95% interval of delta_*r*_(*f*) across null replicates. Thus, observed versus randomized trajectories were compared directly through the observed curve relative to the null mean and interval, as well as through these paired AS − null deviations.

To summarize differences across the full percolation trajectory, we calculated the AUC for of the 1,000 null trajectories using the trapezoidal rule. Null trajectories were generated from the same degree-matched random edge-removal procedure described above. For *S*(*f*) and *E*(*f*), empirical significance was assessed by comparing the observed AUC to the distribution of null AUC values using a one-sided lower-tail test:

$${P}_{\mathrm{AUC}}=\frac{1+{\sum }_{r=1}^{R}1({\mathrm{AUC}}_{\mathrm{null},r}\le {\mathrm{AUC}}_{\mathrm{AS}}}{R+1}$$

where *R* = 1,000, AUC_AS_ is the AUC of the observed AS-guided removal trajectory, AUC_null,*r*_ is the AUC of null replicate *r* and *I* is the indicator function. Thus, the empirical *P* value corresponds to the proportion of null replicates whose AUC is less than or equal to the observed AUC, with a +1 correction applied to both numerator and denominator. This formulation tests whether AS-guided edge removal produces a smaller AUC than expected under random removal, corresponding to a more disruptive percolation trajectory. Reported *P* values for the *S* and *E* percolation analyses, therefore, refer to this trajectory-level AUC comparison rather than to a separate pointwise *P* value at each removal step.

#### Intercommunity edge analysis

As part of the network analysis, we examined whether AS-regulated edges preferentially connect distinct functional communities. Communities were defined once on the original interactome using modularity-based community detection and each edge was annotated as intercommunity if its two endpoint proteins belonged to different communities. Using the same ranked edge-removal sequence as in the percolation analysis, we calculated at each removal fraction *f* the number of removed edges connecting distinct communities.

Observed intercommunity counts were compared to the corresponding null distribution from the same 1,000 degree-matched random removal replicates. At each removal fraction *f*, we calculated a two-sided empirical *P* value by comparing the observed intercommunity count to the null distribution at that same step. Specifically, if the observed value lay below the null mean, the lower-tail empirical probability was computed, whereas, if it lay above the null mean, the upper-tail empirical probability was computed; this tail probability was then doubled to obtain a two-sided empirical *P* value:

$${p}_{f}=2\,\min (\frac{1+\sum _{r=1}^{R}1({I}_{\mathrm{null},r}(f)\le {I}_{\mathrm{AS}}(f))}{R+1},\frac{1+{\sum }_{r=1}^{R}1({I}_{\mathrm{null},r}(f)\ge {I}_{\mathrm{AS}}(f))}{R+1}$$

with values capped at 1. Here, ${I}_{\rm{AS}}(f)$ denotes the observed number of intercommunity edges removed at fraction *f* and ${I}_{{\rm{null}},f}(f)$ is the corresponding value in null replicate *r*. This analysis tests whether AS-regulated interfaces are preferentially positioned at edges linking distinct modules rather than acting mainly within a single module.

#### OLS regression analysis

To evaluate the determinants of AS-driven network variation, we fitted OLS regression models using standardized predictors derived from the interface-resolved interactome. All continuous variables were *z*-scored before modeling to ensure comparability of effect sizes and to reduce scale-dependent differences among predictors. Model parameters were estimated using the statsmodels Python package (version 0.14) and HC3 standard errors were used throughout.

Depending on the analysis, the dependent variable was either a continuous network metric derived from the percolation framework or a continuous edge-level or topology-derived quantity. For analyses linked to percolation, outcomes included *S*, the fraction of nodes retained in the main connected part of the interactome, *E*, the approximate global efficiency of the residual network, and the intercommunity edge metric, which quantifies whether removed edges preferentially connect proteins assigned to different functional communities. Other models used localization entropy or related continuous structural or network features as outcomes, as described below. Structural and regulatory predictors included Pfam, modularity, domain variety, intrinsic disorder, tissue regulation score and network centrality measures such as betweenness, shared neighbors and clustering coefficient. Model fit was assessed using adjusted *R*^2^ and overall model significance was assessed by robust *F*-tests.

To test whether the effects of these predictors differed for microexon-containing proteins, we included explicit interaction terms (for example, Pfam × microexon or regulation × microexon). For each interaction, continuous variables were mean-centered before multiplication to reduce collinearity. The resulting interaction term tests whether the slope of a continuous predictor differs between microexon-containing and non-microexon-containing proteins. For binary variables such as microexon status (1 = microexon-containing, 0 = non-microexon-containing), a significant interaction indicates that the predictor’s effect on the response variable changes specifically in the presence of microexons. When significant interactions were detected, simple-slope analyses were used to estimate the direction and magnitude of the relationship within each group. Model coefficients and 95% confidence intervals were exported for visualization in coefficient plots.

#### Outlier enrichment and quantile regression analysis

To characterize the biological and structural features associated with the most impactful AS events, we performed an outlier enrichment analysis on the extremes of the network impact distribution. Proteins were partitioned into ten equal-sized quantile bins according to their Δ*S*, Δ*E* or combined impact scores. For each bin, we calculated a *z* score by comparing the observed feature mean to a null distribution generated from 200 permutations of the network impact labels. To specifically compare the distribution tails, significance for the top and bottom 10% of nodes was assessed using a two-sided Mann–Whitney *U*-test, with effect sizes quantified using Cliff’s delta. Lastly, we used quantile regression to model the relationship between protein features and network impact at specific quantiles (*τ* = 0.1, 0.5 and 0.9), allowing us to identify features that exert a disproportionate influence on high-impact outliers compared to the median.

#### Localization entropy analysis

To examine spatial consequences of these network effects, subcellular localization data were obtained from the Human Protein Atlas^34^, where each protein was annotated with 1–4+ cellular compartments. A localization entropy metric was computed for each protein as the Shannon diversity of its annotated compartments. Higher localization entropy indicates a broader or more dispersed subcellular distribution. OLS models were then used with localization entropy as the dependent variable and intercomm, microexon status and their interaction (intercomm × micro) as predictors. This allowed testing whether microexons occurring at intercommunity connectors modulate spatial dispersion. A positive coefficient indicates broader localization, whereas a significant negative term indicates event restricts localization to specific compartments.

#### ProtGPS prediction analysis

For protein localization analysis, alternative isoforms were compared using ProtGPS, a protein language model built on ESM2 that predicts the independent probability that a protein sequence localizes to each of 12 condensate-associated subcellular compartments^35^. The model can report probabilities for compartments including, for example, the nucleolus, nuclear speckles, chromosome and nuclear pore complex. For each exon, genomic coordinates were mapped to UniProt protein coordinates and the canonical UniProt sequence was compared to a matched sequence in which the protein segment encoded by the alternative exon was either included or excluded, yielding a +EXON and −EXON isoform pair. ProtGPS probabilities were calculated for both isoforms and a compartment was considered positively predicted when its ProtGPS probability exceeded 0.83, a conservative threshold chosen to match the lower bound of the model’s reported held-out performance. To quantify localization change between isoforms, we calculated the Wasserstein distance (WD) between their 12-dimensional ProtGPS probability distributions. As used in the ProtGPS study^35^, WD provides a measure of dissimilarity between two probability distributions, with larger values indicating a greater predicted redistribution across compartments. Exons with WD > 0.1 were classified as localization changing.

#### Protein annotation

Multimodule proteins were defined as proteins with multiple unique Pfam domains^31^. Phosphorylation sites were based on phosphosite annotation^32^. Proteins with interaction substrate diversity were based on the following Pfam annotations^31^ of domain types: protein (PF00017, PF00018, PF00397, PF00595, PF08416, PF01030, PF00498, PF00533, PF00244, PF00568, PF02213, PF00169, PF00373, PF01344, PF00400, PF00515, PF00514, PF02985, PF00023, PF00560, PF00651, PF00531, PF01335, PF00619, PF02758, PF00452, PF02809, PF00627, PF00789, PF00567, PF00385, PF00439, PF02820, PF01426, PF00622, PF00630), DNA (PF00096, PF00105, PF00046, PF00157, PF00172, PF00010, PF00170, PF00505, PF00250, PF00178, PF02319, PF08779, PF00249, PF00251, PF00907, PF00319, PF01388, PF00651, PF00554, PF04299, PF00126, PF00440, PF02037), RNA (PF00076, PF00013, PF00035, PF00270, PF00271, PF00806, PF02171, PF02170, PF01423, PF01479, PF01299, PF04146, PF00642, PF00098, PF00575, PF01336, PF02926, PF01472, PF01918, PF04352, PF01535), lipid binding (PF00169, PF00168, PF01363, PF00787, PF00130, PF01417, PF07653, PF03114, PF10455, PF00611, PF00373, PF02149, PF02893, PF01167, PF01852, PF01237, PF00650, PF08718, PF00191, PF01477, PF00400). Gene annotations for Fig. 1i were based on Gene Ontology term annotations downloaded from the Ensembl Biomart database^98^.

Data were analyzed and statistically tested as indicated in the relevant sections and figure legends. When multiple tests were performed, *P* values were corrected for FDR using the Benjamini–Hochberg method. The exact numbers of experimental repeats are stated in the figure legends and/or relevant sections. *P* values ≤ 0.05 were considered significant for LUMIER and correlation tests, while *P* values ≤ 0.01 were considered significant for the examination of normalized classifier performances. For all linear modeling, we used HC3 OLS to account for nonconstant variance across network and localization data. Statistical significance for individual model coefficients was determined using a Wald test to generate robust *t* statistics and *P* values. Continuous predictors (for example, regulation score, modularity and centrality) were mean-centered and standardized to unit variance before model fitting; thus, regression coefficients (β) represent the change in the dependent variable per 1-s.d. increase in the predictor. For interaction models, a significant interaction term indicates a difference in the slope of the relationship between groups (for example, microexon-containing versus non-microexon-containing).

In instances where example and representative images are shown, these reflect the outcome of at least three independent experiments with similar outcomes unless otherwise explicitly stated in the associated figure legend and main text.

### Reporting summary

Further information on research design is available in the Nature Portfolio Reporting Summary linked to this article.

## Online content

Any methods, additional references, Nature Portfolio reporting summaries, source data, extended data, supplementary information, acknowledgements, peer review information; details of author contributions and competing interests; and statements of data and code availability are available at https://doi.org/10.1038/s41594-026-01875-8.

## Supplementary information

Supplementary Information Supplementary Figs. 1–4.

Reporting Summary

Peer Review File

Supplementary Tables 1–11 Supplementary Table 1. Interface interact network: interface-resolved interaction network. Columns: ‘gene_a’ and ‘gene_b’ indicate the proteins that interact; ‘interface_start’ and ‘interface_end’ denote the first and last amino acid residues of ‘gene_a’ forming the interaction interface of the used UniProt reference; ‘interface_details’ provides more information on the nature of the region; ‘database’ specifies which interaction database the interaction was extracted from; ‘AS_controlled’ specifies whether the interface in ‘gene_a’ is overlapped by an alternatively spliced exon; ‘VASTDB_event_ID’ specifies the unique exon in vastDB; ‘exon_length_nt’ describes the length of the alternative exon in nucleotides; ‘exon_start’ and ‘exon_end’ describe the first and last amino acids encoded by the exon within the UniProt reference. An exon overlaps an interface if it encodes any residues that occur within the interface. In instances where the ‘exon_start’ and ‘exon_end’ columns start with ‘EEJ = ’, the residues encoded by the exon are not present in the UniProt reference and, instead, the columns indicate where the residues will be inserted. ‘AS_regulation_degree’ specifies the overall tissue specificity for the alternative exon, which is used for ordered removal of the AS-regulated interfaces. Supplementary Table 2. Isoform library: overview of protein isoform library, including exon identifiers, PSIs, predicted effect on protein structure, tag location, PTM status and previous examination. Supplementary Table 3. Imaged isoform pairs: the number of cells, ROC-AUCs and normalized ROC-AUCs for all isoform pairs that we succesfully examined, along with a comparison to previous literature on their localization. Supplementary Table 4. Classifier score versus LUMIER: the median ROC-AUC scores and overall PPI differences based on LUMIER data for all isoform pairs where at least three interactors were positively examined, used to calculate the correlation between localisation and PPI differences. Supplementary Table 5. LUMIER data: the raw LUMIER data (NLIR for each isoform for a given interactor for each experimental repeat) used to assess the overall PPI differences between isoform pairs. Supplementary Table 6. Distinguishing features: the different importance metrics (contribution prop, contribution ratio, etc.) for all features for each isoform pair. ‘P_adj_value’ and ‘adj_significant’ refer to the isoform pair (the protein isoforms differentially localizing), while ‘distinguishing_feature’ refers to whether the given feature was isoform distinguishing for the specific protein isoform pair. Supplementary Table 7. SNX2 AP–MS abundance: the Mascot output of protein quantitation within AP–MS samples, describing the number of significant peptide matches per protein (prot_matches_sig(SPCs)) within each sample. Each column is a sample and each row is a gene. Supplementary Table 8. SNX2 AP–MS interactors: the SAINTexpress output, describing the proteins that are enriched within the IP fraction from the various SNX2 cell lines after pulling down on SNX2. The bait column indicates the sample (wild type = HeLa wild type (expressing SNX2−EX), RescueSNX2plusEx = HeLa SNX2-KO rescued with SNX2+EX and RescueSNX2minusEx = HeLa SNX2-KO rescued with SNX2−EX). The prey column indicates the detected interactor. The spec column describes the spectral counts in each replicate, with | delimiting the replicate. CtrlCount refers to spectral counts in HeLa SNX2-KO cells, which is used to define background or unspecific binding. Supplementary Table 9. Reagents: the reagents used, including the manufacturers, catalog numbers and used concentrations of antibodies and dyes. Supplementary Table 10. Plasmids: the available plasmids used in this study. Supplementary Table 11. Oligonucleotides: the key oligonucleotides used in this study.

Supplementary Table 12 Light microscopy reporting table.

## Source data

Source Data Fig. 1 Statistical source data.

Source Data Fig. 2 Statistical source data.

Source Data Fig. 3 Statistical source data.

Source Data Fig. 4 Statistical source data.

Source Data Fig. 5 Statistical source data.

Source Data Fig. 6 Statistical source data.

Source Data Extended Data Fig. 1 Statistical source data.

Source Data Extended Data Fig. 2 Statistical source data.

Source Data Extended Data Fig. 3 Statistical source data.

Source Data Extended Data Fig. 4 Statistical source data.

Source Data Extended Data Fig. 5 Statistical source data.

Source Data Extended Data Fig. 6 Statistical source data.

Source Data Extended Data Fig. 7 Statistical source data.

Source Data Extended Data Fig. 8 Statistical source data.

Source Data Extended Data Fig. 9 Statistical source data.

Source Data Extended Data Fig. 9 Unprocessed western blots and RT–PCR gel.

## Data Availability

The cell measurements from the imaging-based isoform library examination (https://doi.org/10.6084/m9.figshare.29486819)^102^, the performance metrics of the classifiers generated from analyzing the isoform cell measurements (https://doi.org/10.6084/m9.figshare.29487851)^103^, the classifier-derived feature importances for the isoform classifiers (https://doi.org/10.6084/m9.figshare.29487845)^104^, the cell measurements from the imaging-based SNX2 mutant library examination (https://doi.org/10.6084/m9.figshare.29487671)^105^ and the cell measurements from the imaging-based HeLa SNX2 cell line examination using antibodies (https://doi.org/10.6084/m9.figshare.32178351)^106^ were deposited to figshare. The MS data were deposited to the PRIDE database with accession number PXD074758. Source data are provided with this paper.

## Code availability

In-house developed custom analysis scripts are available from GitHub (https://github.com/weatheritt2/Hansen_AS/) under MIT license.
