# Supplementary material for: Alternative splicing fine-tunes modular protein interfaces to control protein localization
Source: Nat Struct Mol Biol. 2026 Oct 14;33(11):1609–23. doi: 10.1038/s41594-026-01875-8 (PMC13759335; doi:10.1038/s41594-026-01875-8)
Supplement: Supplementary file 1 [file 41594_2026_1875_MOESM1_ESM.pdf]

nature structural & molecular biology

Supplementary information https://doi.org/10.1038/s41594-026-01875-8

Alternative splicing fine-tunes modular protein interfaces to control protein localization

In the format provided by the authors and unedited

Supplementary Figure 1

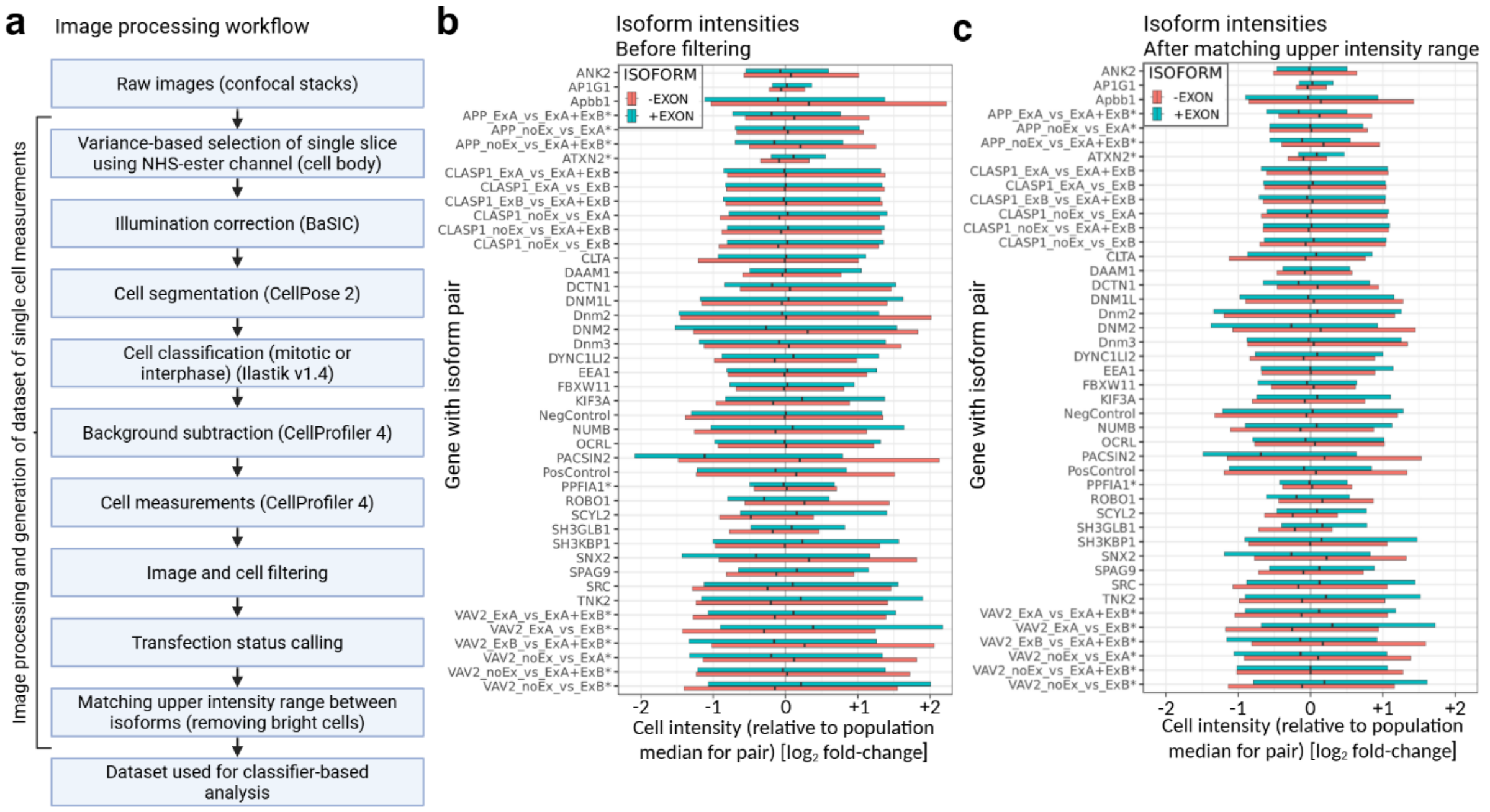

Supplementary Fig 1. Generation of a single cell isoform measurement dataset from the acquired images, and relative isoform expression levels within isoform pairs before/after matching upper expression range. a, Overview of image processing to generate the cell datasets used for the classifier-based analysis (**Extended Data Fig. 3a**) (Methods). The selected focus slice from each confocal stack is used for all four channels for all subsequent processing. **b,** Boxplots showing the cells' isoform expression (cell mean intensity) relative to the median population expression (mean cell intensity) of all cells expressing either isoform forming the given pair before applying any form of intensity filtering. The boxplot's centreline indicates median, and box edges mark the interquartile range (IQR). The x-axis shows the relative expression level on a log2-scaled axis, and the y-axis shows the isoform pairs. Colour indicates isoform. **c,** Boxplots showing the cells' isoform expression (cell mean intensity) relative to the median population expression (mean cell intensity) of all cells expressing either isoform forming the given pair, after the isoform pair had been filtered to have a matched upper intensity (Methods). The boxplot's centreline indicates median, and box edges mark the interquartile range (IQR). The x-axis shows the relative expression level on a log2-scaled axis, and the y-axis shows the isoform pairs. Colour indicates isoform.

Supplementary Figure 2

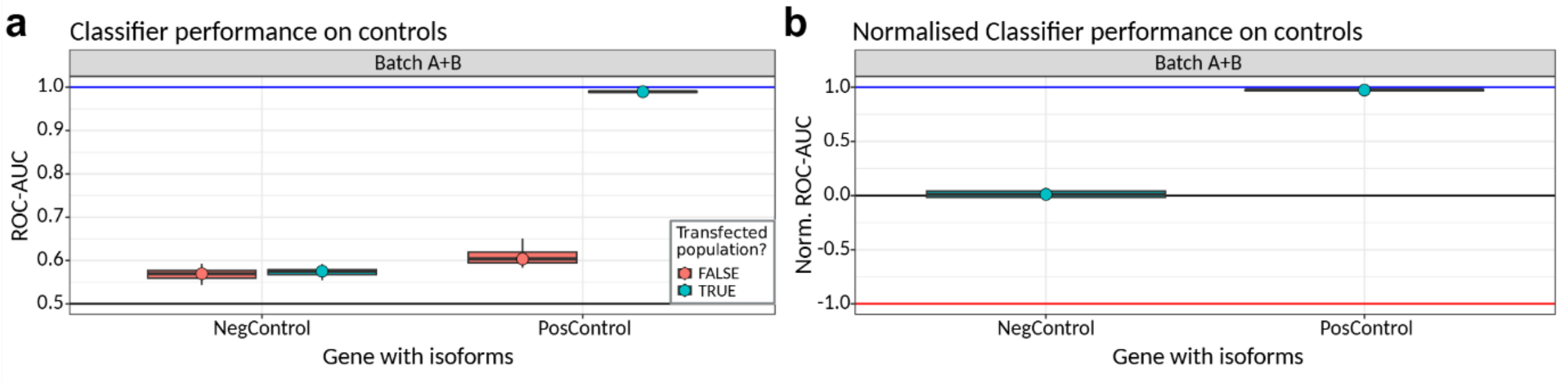

Supplementary Fig. 2. Pooling cells across experiment batches doesn't alter performance for the positive and negative controls. a, Boxplots showing the ability of classifiers trained on mCherry features measured in cells expressing control plasmids to correctly assign the plasmid ID (which corresponds to well ID), quantified as ROC-AUC. Cells were pooled from two separate experiments (batch A and B), each with three experimental repeats, before the classifier-based evaluation (n cells ≥ 1500 after pooling). The boxplots illustrate the ROC-AUCs from each of 50 iterations of cell sampling and classifier evaluation, and dots indicate median ROC-AUC, box edges mark the interquartile range (IQR), and whiskers show observations within 1.5× IQR. Classifier-based analysis was performed separately on transfected (isoform) and non-transfected (internal normalisation) cells from the same, indicated by colour. Coloured lines indicate perfect (blue) and worst (black) possible performance, respectively. **b,** Same as **a.**, but the boxplots display classifier performance quantified as normalised ROC-AUC (norm. ROC-AUC). Coloured lines indicate perfect (blue), neutral (black) or worst (red) possible performance, respectively.

Supplementary Figure 3

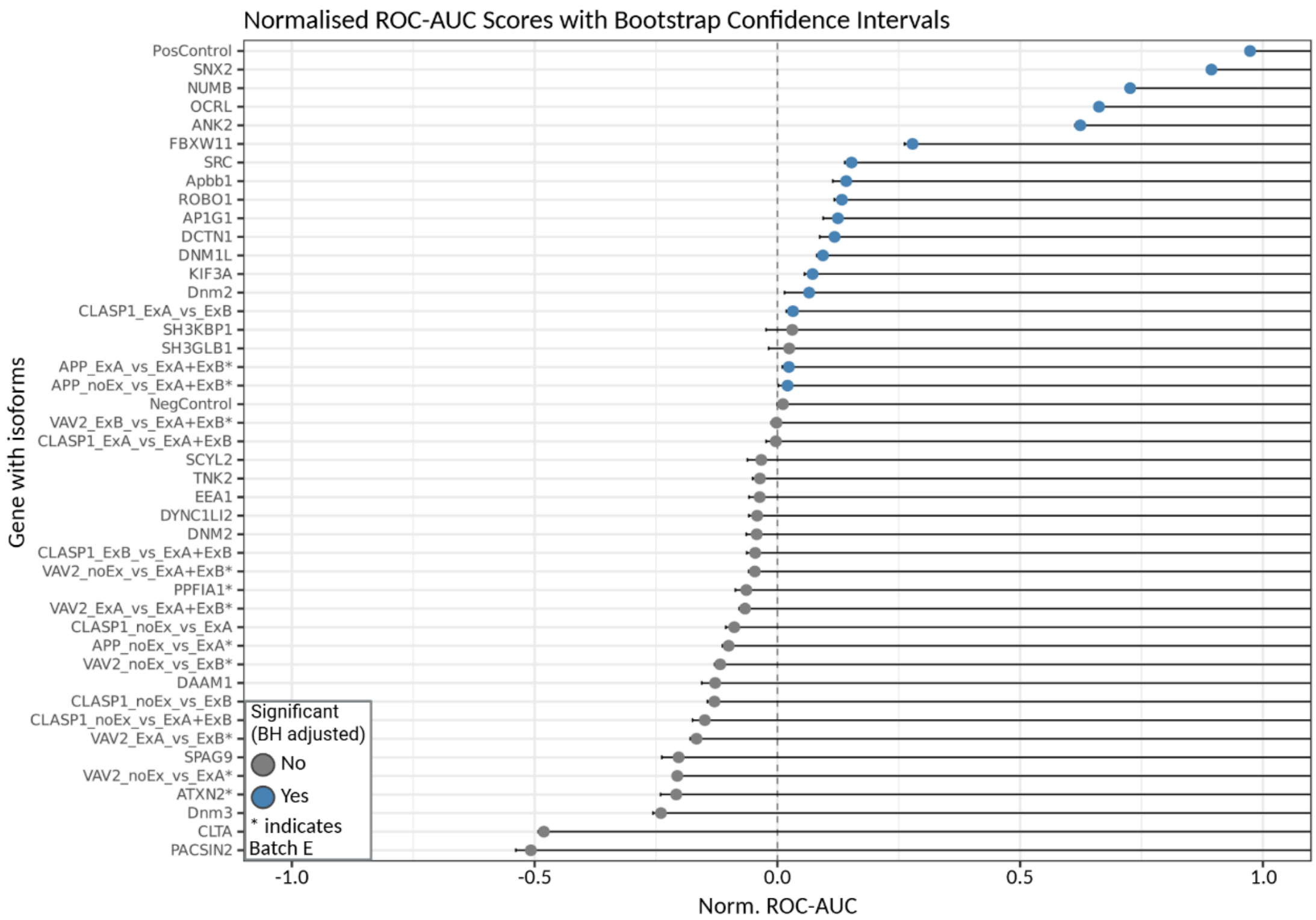

Supplementary Fig. 3. Bootstrap analysis of normalised ROC-AUCs from isoform pairs. XY plots showing bootstrap statistical testing of the normalised ROC-AUC (norm. ROC-AUC) scores for the isoform pairs from **Fig. 2f**. The normalised ROC-AUC scores for each pair were analysed via bootstrapping, and the displayed values are the bootstrap mean (dot) and 99% confidence intervals (CI) (line). Isoform pairs that didn't contain 0 in the lower bound of the CI after false discovery rate correction were identified as having isoform classifiers significantly outperforming the internal normalisation classifier, interpreted as isoform differences are detectable after considering well-specific effects, and are coloured blue. Isoform pairs with 0 in the CI were found as non-significant, coloured grey. n bootstrap iterations = 2000, one-sided test, $p < 0.01$, *.

Supplementary Figure 4

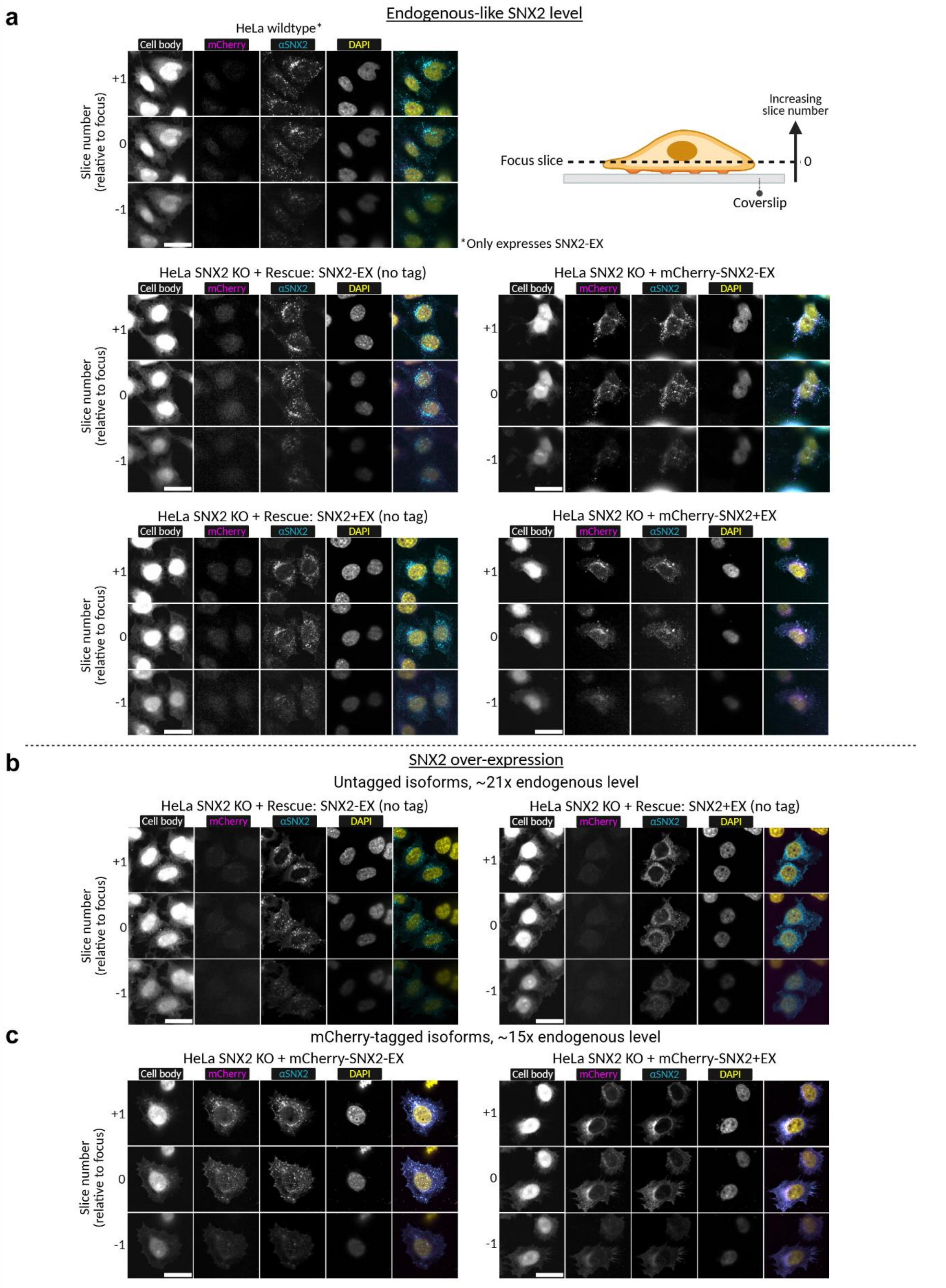

Supplementary Fig. 4. Slice-by-slice of representative images of HeLa cell lines expressing untagged and mCherry-SNX2 isoforms. a, Slice-by-slice view of the representative images shown in **Fig. 6a** with cells expressing the SNX2 constructs at endogenous-like levels. The cells are either HeLa wildtype, HeLa SNX2 KO, HeLa SNX2 KO stably expressing untagged SNX2+EX or SNX2-EX, or HeLa SNX2 KO transiently expressing mCherry-SNX2+EX or

SNX2-EX. The cell body, mCherry, αSNX2, and DAPI channels are shown independently and merged. Representative cells were chosen to have comparable expression levels based on the αSNX2 immunostaining signal. The same FOV is shown across three consecutive slices (-1, 0, +1) from the confocal stack, with 0 denoting the 'focus slice', which is the one shown in **Fig. 6a**. The association between the slice number and what region of the cell is imaged is indicated by the schematic top right. Scale bar: 25μm. **b,** Same as **a**, except the shown slices relate to the representative images shown in **Extended Data Fig. 9e**. The cells are HeLa SNX2 KO rescues over-expressing untagged SNX2 isoforms at ~21x endogenous SNX2 amounts. **c,** Same as **a**, except the shown slices relate to the representative images shown in **Extended Data Fig. 9h**. The cells are HeLa SNX2 KO transiently expressing mCherry-SNX2 isoforms at ~15x endogenous SNX2 amounts. The representative images reflect the behavior that was consistently observed across 3 independent experiments.
