# Supplementary material for: Alternative splicing fine-tunes modular protein interfaces to control protein localization
Source: Nat Struct Mol Biol. 2026 Oct 14;33(11):1609–23. doi: 10.1038/s41594-026-01875-8 (PMC13759335; doi:10.1038/s41594-026-01875-8)
Supplement: Supplementary file 2 [file 41594_2026_1875_MOESM21_ESM.pdf]

Extended Figure S8A (SNX2 KO, western)

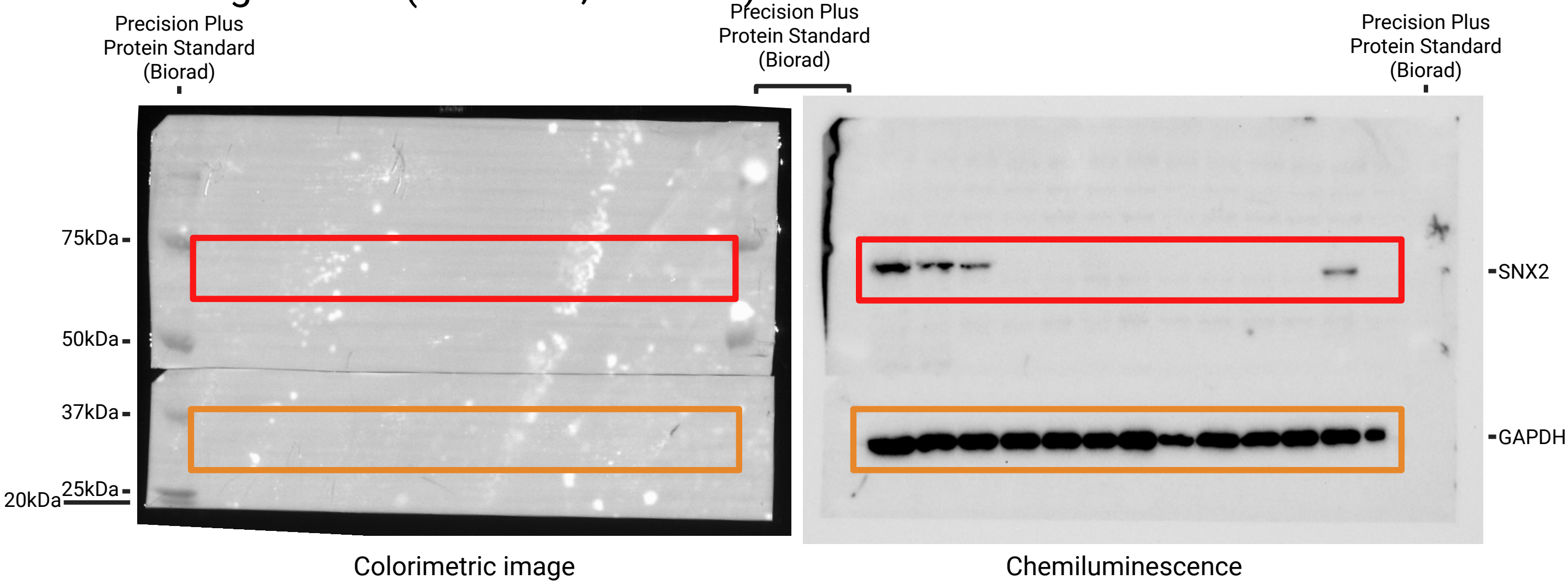

Extended Figure S8B (SNX2 rescue, western)

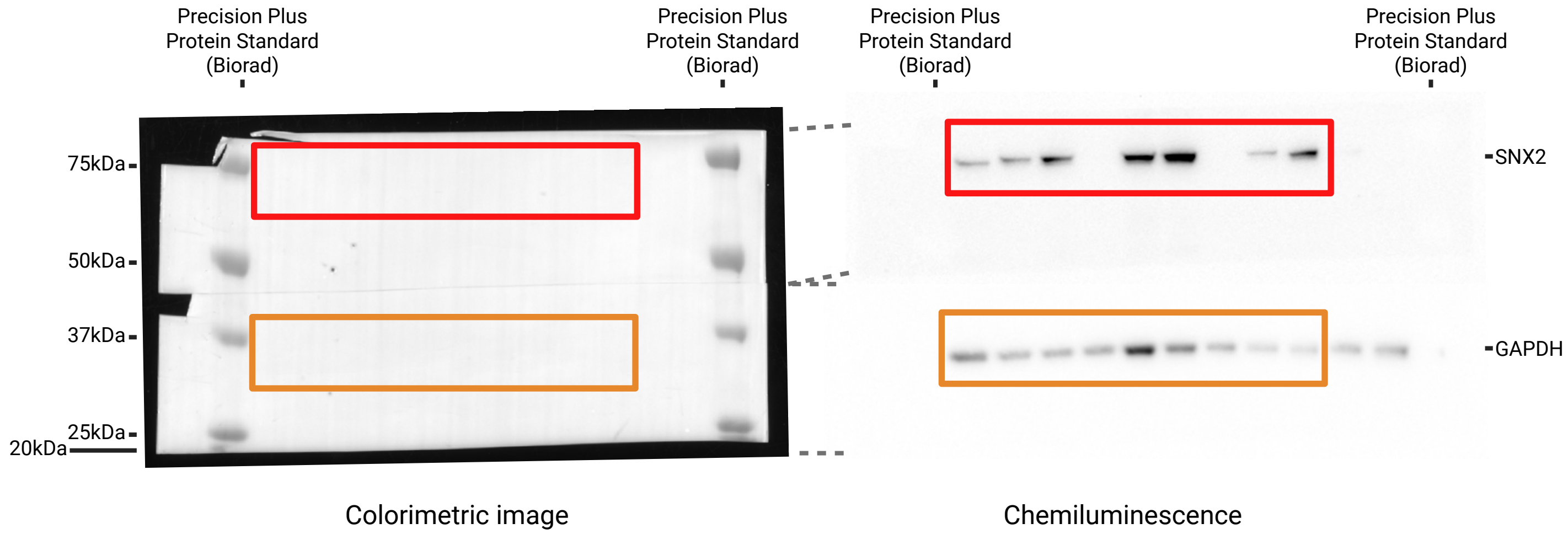

Exteded Figure S8C (RT-PCR)

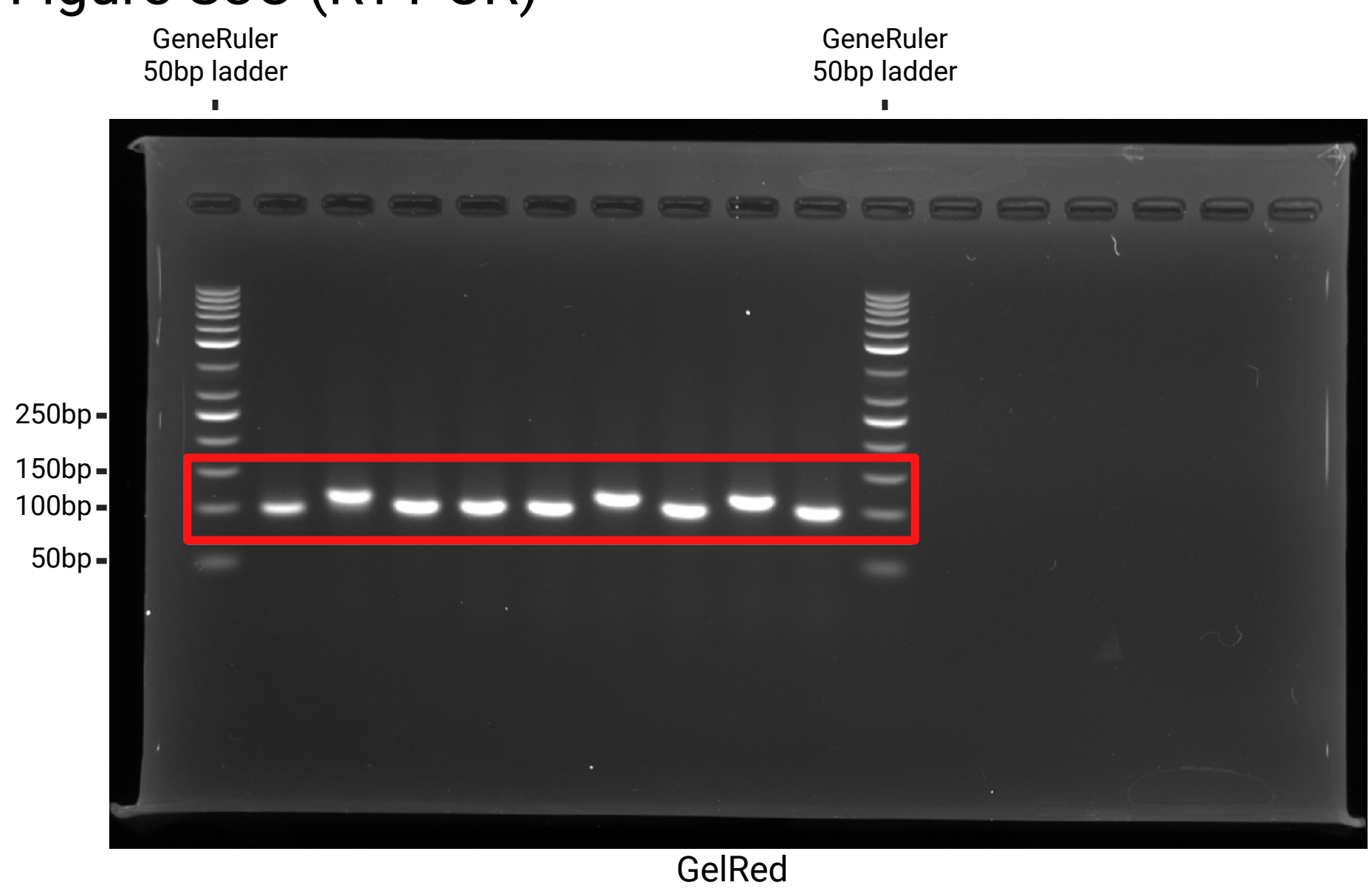

End-point RT-PCR with primers flanking SNX2 microexon

Primers:
Forward:
GCTGAAGCAAAAATGATGGTTGC
Reverse:
TTCCCCTTGTTGCACTTTCGC

Expected PCR products:
96bp (microexon excluded)
108bp (microexon included)
