# Supplementary material for: Alternative splicing fine-tunes modular protein interfaces to control protein localization
Source: Nat Struct Mol Biol. 2026 Oct 14;33(11):1609–23. doi: 10.1038/s41594-026-01875-8 (PMC13759335; doi:10.1038/s41594-026-01875-8)
Supplement: Supplementary file 3 [file 41594_2026_1875_MOESM2_ESM.pdf]

nature portfolio

Corresponding author(s): ROBERT WEATHERITT

Last updated by author(s): Jul 7, 2026

Reporting Summary

Nature Portfolio wishes to improve the reproducibility of the work that we publish. This form provides structure for consistency and transparency in reporting. For further information on Nature Portfolio policies, see our Editorial Policies and the Editorial Policy Checklist.

Statistics

For all statistical analyses, confirm that the following items are present in the figure legend, table legend, main text, or Methods section.

n/a	Confirmed	
☐	☒	The exact sample size (*n*) for each experimental group/condition, given as a discrete number and unit of measurement
☐	☒	A statement on whether measurements were taken from distinct samples or whether the same sample was measured repeatedly
☐	☒	The statistical test(s) used AND whether they are one- or two-sided<br>*Only common tests should be described solely by name; describe more complex techniques in the Methods section.*
☒	☐	A description of all covariates tested
☐	☒	A description of any assumptions or corrections, such as tests of normality and adjustment for multiple comparisons
☐	☒	A full description of the statistical parameters including central tendency (e.g. means) or other basic estimates (e.g. regression coefficient) AND variation (e.g. standard deviation) or associated estimates of uncertainty (e.g. confidence intervals)
☐	☒	For null hypothesis testing, the test statistic (e.g. *F*, *t*, *r*) with confidence intervals, effect sizes, degrees of freedom and *P* value noted<br>*Give P values as exact values whenever suitable.*
☒	☐	For Bayesian analysis, information on the choice of priors and Markov chain Monte Carlo settings
☒	☐	For hierarchical and complex designs, identification of the appropriate level for tests and full reporting of outcomes
☒	☐	Estimates of effect sizes (e.g. Cohen's *d*, Pearson's *r*), indicating how they were calculated

Our web collection on statistics for biologists contains articles on many of the points above.

Software and code

Policy information about availability of computer code

Data collection	Flow cytometry software: BD FACSDiva software v8.0<br>Fluorescence microscopy software (for PerkinElmer Operetta CLS): Harmony v4.9<br>CLARIOstar plate-reader software: CLARIOstar v5.01
Data analysis	NETWORK ANALYSIS.<br>Python 3.9 and R version 4.41 were used for data analysis. All analysis scripts are available with dependencies at https://github.com/weatheritt2/Hansen_AS/. For imaging analysis the following softwares and plugins were used: ImageJ (>v1.51), BaSIC v1.0 (imageJ plugin), find focused slice v1.0 (imageJ plugin), CellPose v2.0, Ilastik (>v1.4), CellProfiler (>v4.25).<br>For affinity purification mass-spectrometry (AP-MS), the following software was used: Mascot v2.8, SAINTexpress v3.6.3.

For manuscripts utilizing custom algorithms or software that are central to the research but not yet described in published literature, software must be made available to editors and reviewers. We strongly encourage code deposition in a community repository (e.g. GitHub). See the Nature Portfolio guidelines for submitting code & software for further information.

Data

Policy information about availability of data

All manuscripts must include a data availability statement. This statement should provide the following information, where applicable:

- Accession codes, unique identifiers, or web links for publicly available datasets
- A description of any restrictions on data availability
- For clinical datasets or third party data, please ensure that the statement adheres to our policy

A large amount of the supplementary data is available via the supplementary tables (1-12).

In addition, specific datasets are available as follows:
The cell measurements from the imaging-based isoform library examination have been deposited at Figshare: https://doi.org/10.6084/m9.figshare.29486819
The performance metrics of the classifiers generated from analysing the isoform cell measurements have been deposited at Figshare: https://doi.org/10.6084/m9.figshare.29487851
The classifier-derived feature importances for the isoform classifiers have been deposited at FigShare: https://doi.org/10.6084/m9.figshare.29487845
The cell measurements from the imaging-based SNX2 mutant library examination have been deposited at Figshare: https://doi.org/10.6084/m9.figshare.29487671
The cell measurements from the imaging-based HeLa SNX2 cell lines examination using antibodies has been deposited at Figshare: https://doi.org/10.6084/m9.figshare.32178351
The mass spectrometry data has been deposited in PRIDE database with accession number: PXD074758

Research involving human participants, their data, or biological material

Policy information about studies with human participants or human data. See also policy information about sex, gender (identity/presentation), and sexual orientation and race, ethnicity and racism.

Reporting on sex and gender	N/A
Reporting on race, ethnicity, or other socially relevant groupings	N/A
Population characteristics	N/A
Recruitment	N/A
Ethics oversight	N/A

Note that full information on the approval of the study protocol must also be provided in the manuscript.

Field-specific reporting

Please select the one below that is the best fit for your research. If you are not sure, read the appropriate sections before making your selection.

☒ Life sciences ☐ Behavioural & social sciences ☐ Ecological, evolutionary & environmental sciences

For a reference copy of the document with all sections, see nature.com/documents/nr-reporting-summary-flat.pdf

Life sciences study design

All studies must disclose on these points even when the disclosure is negative.

Sample size	No statistical methods were used to predetermine sample size, as effect sizes and variance were unknown prior to experimentation, which precluded formal power calculations. Sample sizes were generally N = 3-4 independent biological replicates which were chosen based on standard practice for similar molecular biology experiments and were sufficient to assess consistency of effects and perform statistical testing. For the imaging experiments, our experimental design was more elaborate and incorporated two levels of replication (as is typical for single-cell imaging studies): 1) biological replication with N = 3-6 independent experiments (separate cell cultures/transfections performed on different days or sequentially on the same day), and 2) technical replication with at least >500 single-cell measurements per condition across all replicates (typically around 500 cells per biological replicate). The biological replicates ensure reproducibility of findings across independent experiments, while the large number of single-cell observations provides sufficient statistical power to detect both large and subtle phenotypic differences.
Data exclusions	For the imaging data of the protein isoform library and SNX2 mutant constructs, we excluded certain data at the image and single cell level based on pre-established quality control criteria, which we applied before the down-stream quantitative analysis. These exclusions were based on technical considerations and were performed to reduce skewing subsequent analysis and were applied uniformly to all conditions. For entire images, we excluded images with 1) visible artifacts such as fibres, 2) out-of-focus cells, 3) completely confluent field-of-view. This removed <5% of the raw data. For the single cells, we excluded cells, that were 1) predicted to be mitotic or dying, 2) non-mononucleated, 3) whose nucleus touched another nucleus, or 4) had most of its cell boundary (>85%) touching other cells. These exclusions were made to

remove cells in a different cell state (mitosis/dying) and had characteristics that increased the risk of inaccurate cell segmentation. The single cell criteria removed approximately 20-30% of all cells.
For the AP-MS data, one replicate (replicate 3/experiment 3) was excluded from the SAINTexpress analysis as two prey samples (HeLa wildtype, HeLa RescueSNX2plusEx) had substantially lower peptide counts than in the remaining experiments/

For the other data types in the study, no data was excluded.

Replication

All key experiments were repeated 3-6 times as independent biological replicates. These repeats were either performed on separate days or in a sequential fashion on the same day. In either case, the repeat was performed with independently cultured and passaged cells and transfections. Specifically, cells in different repeats from the same experiment were passaged separately twice before the experiment to ensure some biological heterogeneity in the population. The results from the experiments (protein localisation, protein-protein interactions) were consistent across all replicates.

Randomization

Randomization is not relevant to this study. Experimental conditions (protein isoforms/variants) were defined by the specific genetic constructs being tested rather than assigned to samples.

Blinding

Investigators were blinded to the sample identifies (isoform pair identity, and SNX2 mutant construct identity) when performing the imaging experiments. In some cases, blinding wasn't possible. Generally, blinding was less relevant to this study as data acquisition was automated (images were acquired in the same, pre-defined pattern for all samples) to improve consistency and reduce bias. For data analysis, we similarly used a standardized approach that was applied indiscriminately to all samples for a given experiment

Reporting for specific materials, systems and methods

We require information from authors about some types of materials, experimental systems and methods used in many studies. Here, indicate whether each material, system or method listed is relevant to your study. If you are not sure if a list item applies to your research, read the appropriate section before selecting a response.

Materials & experimental systems

n/a	Involved in the study
☐	☒ Antibodies
☐	☒ Eukaryotic cell lines
☒	☐ Palaeontology and archaeology
☒	☐ Animals and other organisms
☒	☐ Clinical data
☒	☐ Dual use research of concern
☒	☐ Plants

Methods

n/a	Involved in the study
☒	☐ ChIP-seq
☐	☒ Flow cytometry
☒	☐ MRI-based neuroimaging

Antibodies

Antibodies used

Use concentrations are specified in parenthesis after the specific use.
• α-SNX2 (Host: Mouse. Clonality: Monoclonal. Clone: 13/SNX2. Manufacturer: BD BioSciences. Catalogue: 611308. LOT: 1060652) used for western blotting (0.25µg/mL), immunoprecipitation (1.25µg), and immunofluorescence (1.25µg/mL).
• α-GOLGA1 (Host: Mouse. Clonality: Monoclonal. Clone: CDF4. Manufacturer: ThermoFisher Scientific. Catalogue: 14-9767-82. LOT: 2373437) used for immunofluorescence (0.4µg/mL).
• α-GAPDH (Host: Mouse. Clonality: Monoclonal. Clone: 0411. Manufacturer: Santa Cruz. Catalogue: sc-47724. LOT: G2920) used for western blotting (0.04µg/mL).
• α-FLAG M2 (Host: Mouse. Clonality: Monoclonal. Clone: M2. Manufacturer: Merck. Catalogue: F1804) used for LUMIER (10µg/mL).
• Goat α-mouse Alexa-488 (Host: Goat. Clonality: Polyclonal. Manufacturer: Jackson ImmunoResearch. Catalogue: 115-546-062) used for immunofluorescence (3µg/mL).
• Goat α-mouse-HRP (Host: Goat. Clonality: Polyclonal. Manufacturer: ThermoFisher Scientific. Catalogue: 31430) used for western blotting (0.16µg/mL).

Validation

Relating to primary antibodies:
• SNX2: In the manuscript, the specificity of the SNX2 antibody is verified by the loss of a band upon western blotting of whole-cell lysates from HeLa SNX2 knockout cell lines and, similarly, by the loss of SNX2 signal upon fluorescence microscopy-based examination of the HeLa SNX2 knockout cells. The SNX2 signal was restored in both western blotting and immunofluorescence microscopy readouts upon exogenous introduction of SNX2 via transduction with a cassette encoding SNX2, confirming the antibody is specific to SNX2.
• GOLGA1: On the manufacturer's website, a western blot is provided that shows decreased golgin97 (protein encoded by GOLGA1) abundance upon treating cells with siRNA targeting GOLGA1 mRNA.
• GAPDH: On the manufacturer's website, a western blot is provided that shows decreased GAPDH protein abundance upon treating cells with siRNA targeting GAPDH mRNA (data shown for the siRNA product: sc-47724).
• FLAG: On the manufacturer's website, a fluorescence microscopy image is provided that shows antibody signal in only select cells whereas other cells show no signal. The cells were transfected with a plasmid encoding a FLAG-fusion and is the expected pattern if not all cells are transfected AND the FLAG epitope isn't endogenously expressed.

Eukaryotic cell lines

Policy information about cell lines and Sex and Gender in Research

Cell line source(s)	The cell lines were sourced from American Type Culture Collection (ATCC): HeLa (ATCC CCL-2), HEK293T (ATCC CRL-3216).
Authentication	None of the used cell lines were authenticated.
Mycoplasma contamination	Cell lines were not tested for mycoplasma contamination.
Commonly misidentified lines (See ICLAC register)	No commonly misidentified cell lines were used in this study.

Plants

Seed stocks	N/A
Novel plant genotypes	N/A
Authentication	N/A

Flow Cytometry

Plots

Confirm that:

☒ The axis labels state the marker and fluorochrome used (e.g. CD4-FITC).

☒ The axis scales are clearly visible. Include numbers along axes only for bottom left plot of group (a 'group' is an analysis of identical markers).

☒ All plots are contour plots with outliers or pseudocolor plots.

☒ A numerical value for number of cells or percentage (with statistics) is provided.

Methodology

Sample preparation	Flow cytometry was only used in the form of fluorescence-activated cell sorting (FACS) to generate clonal HeLa SNX2 knockout lines.<br>HeLa wildtype cells were transiently transfected with a plasmid encoding a GFP reporter alongside Cas9 and gRNAs targeting SNX2. 48 hours after transfection, the HeLa cells were washed in PBS, then dissociated using trypsin-EDTA for 5' at +37C. The cells were then resuspended in supplemented DMEM and pelleted by centrifugation (200g for 5' at room temperature). The supernatant was discarded, and the cell pellet was resuspended in PBS with 2% FBS and penicillin/streptomycin and then strained through a 35µm nylon mesh into flow tubes. The tubes with cells were kept on ice until sorted, which was performed at +4C.
Instrument	Cells were sorted on a BD FACS Aria III
Software	The instrument was controlled via the BD FACSDiva software v8.0
Cell population abundance	About 10% of cells were identified to be GFP+. Individual GFP+ cells were sorted directly into separate wells in 96-well plates
Gating strategy	1) Cells were initially identified by gating on FSC-A vs. SSC-A gate. From 1), we identified single cells 2) by gating on the diagonal of FSC-H vs. FSC-A. We then identified GFP+ single cells 3) by identifying single cells with GFP intensities (signal in the B-530/30 channel) at least >5 above a GFP- sample (mock transfected cells).

☐ Tick this box to confirm that a figure exemplifying the gating strategy is provided in the Supplementary Information.
