# Supplementary material for: Alternative splicing fine-tunes modular protein interfaces to control protein localization
Source: Nat Struct Mol Biol. 2026 Oct 14;33(11):1609–23. doi: 10.1038/s41594-026-01875-8 (PMC13759335; doi:10.1038/s41594-026-01875-8)
Supplement: Supplementary file 4 [file 41594_2026_1875_MOESM3_ESM.pdf]

nature portfolio

Peer Review File

Alternative splicing fine-tunes modular protein interfaces to control protein localisation

Corresponding Author: Dr Robert Weatheritt

Version 0:

Decision Letter:

17th Dec 2025

Dear Dr. Weatheritt,

Thank you again for submitting your manuscript "Alternative splicing fine-tunes modular protein interfaces to control protein localisation". We have decided to send your manuscript for peer review, but several points require your attention before we can proceed with peer review.

I am re-opening the manuscript submission system for you to resubmit when ready your manuscript with all associated files needed for peer review directly. As discussed in our correspondence, we expect to receive such information in the following weeks, at your convenience, and once we have done so, we will reach out to potential reviewers. Please follow the link at the bottom of this email to upload the documents listed below. If you have any issues, please reach out to us before completing the submission.

1. REPORTING SUMMARY:
We want to ensure that the methods and statistics reporting in our papers are of the highest quality. To that end, we ask authors to fill out a Reporting Summary that collects information on experimental design and reagents. If your paper includes ChIP-seq or flow cytometry data, we ask that you take special care to complete those sections of the Reporting Summary as this data will aid greatly in the review of your manuscript.

Reporting Summary:
https://www.nature.com/documents/nr-reporting-summary.pdf

2- PROTEOMICS AND SEQUENCING:
Please provide accession numbers and reviewer tokens to access sequencing or proteomics datasets if any unpublished datasets are part of your study. Please add this information to your manuscript file. Proteomics data should be deposited to any ProteomeXchange member repository (http://www.proteomexchange.org/) and DNA sequencing, RNA sequencing, genome assembly data should be deposited to any INSDC member repository (https://www.insdc.org/) or Genome Sequence Archive.

3- CODE, MD SIMULATIONS, AND MACHINE LEARNING REPORTING:
Please provide the code used to analyse the data if newly developed (unpublished) code was used in the work. You can upload the code to GitHub and provide the link directly in the manuscript. Alternatively, if you have not yet provided relevant code, you could provide a single compressed zip file containing the software with a readme.txt file or other user manual containing complete instructions for installing and running the software. If appropriate, please provide example data and expected output. Sufficient material should be provided for referees to directly test the performance of the software/algorithm.

Please fill out and upload the code and software submission checklist that will be made available to editors and reviewers. This form is a dynamic 'smart pdf' and must therefore be downloaded and completed in Adobe Reader, instead of opening it in a web browser.
https://www.nature.com/documents/nr-software-policy.pdf

If it applies, please complete the completed machine learning checklist with your submission and upload it for review:
https://www.nature.com/documents/machine-learning-checklist.pdf

Please use the link below to submit the files. **Please also remember to move forward all other files associated with this version of the paper.**

Link Redacted

Note: This URL links to your confidential home page and associated information about manuscripts you may have submitted, or that you are reviewing for us. If you wish to forward this email to co-authors, please delete the link to your homepage.

We look forward to seeing the revised manuscript and thank you for the opportunity to review your work.

Sincerely,

Dimitris Typas
Senior Editor
Nature Structural & Molecular Biology
ORCID: 0000-0002-8737-1319

Version 1:

Decision Letter:

30th Jan 2026

Dear Dr. Weatheritt,

Thank you again for submitting your manuscript "Alternative splicing fine-tunes modular protein interfaces to control protein localisation". We now have comments (below) from the 4 reviewers who evaluated your paper. In light of these reports, we remain interested in your study and would like to see your response to the comments of the referees in the form of a revised manuscript. Please note that Reviewer #4 co-reviewed the work with another referee as part of the Early Career Researcher inititive of Nature portfolio journals.

You will see that while all reviewers appreciate the potential of the study and its timeliness, they raise crucial concerns with respect to the rigour of aspects of the study which must be comprehensively and convincingly addressed in a revised version for the editorial team to re-engage with the reviewers. In particular, Reviewer #1 notes the lack of sufficient description of the computational part, that does not allow for its assessment and reproducibility. Further to fully resolving any issues with respect to how analyses were performed and abiding by relevant community standards, Reviewer #2 requests that mitigating effects of transient expression of fluorescent-tagged protein isoforms must be excluded to lend sufficient credence to the validity of the imaging screen, as well as depositing proteomics data via PRIDE. For this latter ommission, we editorially apologise as we should have mandated such deposition before sending the manuscript to the reviewers. Please note that we expect you to fully and persuasively respond to these and other technical points raised, and where relevant, such as in the final point of Reviewer #3 for example, provide additional analyses to expand the functional insight of the work.

Our standard revision period is 4-6 months. We appreciate that the requested revisions are extensive. We are committed to providing a fair and constructive peer-review process and encourage you to contact us if you would like to discuss the reviews or anticipate any delay with the resubmission.

We will be happy to consider your revision as long as nothing similar has been accepted for publication at NSMB or published elsewhere. Should your manuscript be substantially delayed without notifying us in advance and your article is eventually published, the received date would be that of the revised, not the original, version.

REPORTING AND DATA AVAILABILITY: Please provide updated files as follows with your revision. As you already know, we put great emphasis on ensuring that the methods and statistics reported in our papers are correct and accurate.

1. REPORTING SUMMARY: Please provide an updated file:

https://www.nature.com/documents/nr-reporting-summary.pdf

This form is a dynamic ‘smart pdf’ and must be downloaded and completed in Adobe Reader.

2. REPORTING OF STRUCTURAL DATA: If there are additional or modified structures in the revision, please submit the

corresponding PDB validation reports with your other files, and use the figshare integration system to provide access to maps, half-maps, and models.

Manuscripts reporting new structures should contain a table summarizing structural and refinement statistics. Please include these tables for new cryo-EM or -ET (https://www.nature.com/documents/nr-tables-cryo-em.doc and modifying this file for ET), NMR (https://www.nature.com/documents/nr-tables-nmr.doc) and X-ray crystallography (https://www.nature.com/documents/nr-tables-xray.doc). To facilitate assessment of the quality of the structural data, a stereo image of a portion of the electron density map (for crystallography papers) or of the superimposed lowest energy structures (>10; for NMR papers) should be provided with the submitted manuscript. If the reported structure represents a novel overall fold, a stereo image of the entire structure (as a backbone trace) should also be provided. For cryo-EM structures, a representative micrograph showing individual particles should be provided in the figures alongside a processing workflow (please see our Editorial for more information: https://www.nature.com/articles/s41594-025-01567-9).

DATA DEPOSITION: We require deposition of coordinates (and, in the case of crystal structures, structure factors) into the Protein Data Bank with the designation of immediate release upon publication (HPUB). Electron microscopy-derived density maps and coordinate data must be deposited in EMDB and released upon publication. Deposition and immediate release of NMR chemical shift assignments are requested. To avoid delays in publication, dataset accession numbers must be supplied with the revised manuscript and appropriate release dates must be indicated at the galley proof stage.

3. REPORTING OF LIGHT MICROSCOPY DATA: For any revision that includes light microscopy data, we ask our authors to please include a completed light microscopy reporting table [https://www.nature.com/documents/Light_microscopy_reporting_table.xlsx] to ensure the methods are described thoroughly. The table will be available to reviewers and ultimately published, should the manuscript be accepted at the journal. When submitting the revised version of your manuscript, please pay close attention to our href="https://www.nature.com/nature-portfolio/editorial-policies/image-integrity">Digital Image Integrity Guidelines.

4. CODE AND COMPUTATIONAL WORK: If newly developed, unpublished code was used in this work, it must be provided with the revision and access to it must be disclosed in the Code Availability Statement in the manuscript. Please also provide the completed software submission checklist. This form is a dynamic ‘smart pdf’ and must be downloaded and completed in Adobe Reader, instead of opening it in a web browser: https://www.nature.com/documents/nr-software-policy.pdf

If molecular dynamics (MD) simulations were performed, please refer to this Editorial and provide a completed MD simulations checklist with your revision: https://www.nature.com/articles/s42003-023-04653-0

Lastly, if it applies, please complete and upload the completed machine learning checklist with your submission for re-review: https://www.nature.com/documents/machine-learning-checklist.pdf

5. SOURCE GEL IMAGES: Unprocessed scans should be provided for all gels and western blots presented in figures and should be clearly labelled with the figure number in the file title. The source gel images should be uncropped, unmodified images, with molecular weight markers. Uncropped, unmodified gel images from existing experimental repeats, in addition to the data shown in the figures, can also be presented in source data. Please provide these files as PDF with your revision.

Please ensure that all control panels for gels and western blots are appropriately described as loading or IP controls. Lastly, please ensure that all images in the paper are checked for duplication of panels and for splicing of gel lanes.

6. SOURCE NUMERICAL DATA: we urge authors to provide, in tabular form in excel files, the data underlying the graphical representations used in figures. This is to further increase transparency in data reporting, as detailed in this editorial (http://www.nature.com/nsmb/journal/v22/n10/full/nsmb.3110.html). Please provide one excel file per figure max, with one panel per tab. When submitting files, the title field should indicate which figure the source data pertains to.

7. DATA AVAILABILITY: this journal strongly supports public availability of data. All data used in accepted papers should be available via a public data repository, or alternatively, as Supplementary Information. If data can only be shared on request, please explain why in your Data Availability Statement and in the correspondence with your editor.

For some data types, deposition in a public repository is mandatory as detailed below:

https://www.nature.com/nature-research/editorial-policies/reporting-standards#availability-of-data

Finally, please ensure that you retain unprocessed data and metadata files after publication, ideally archiving data in perpetuity, as these may be requested during the peer review and production process or after publication if any issues arise.

EXTENDED DATA FIGURES

When re-submitting your manuscript, please ensure that any supplementary figures and tables that are crucial to the manuscript's conclusions are converted into Extended Data figures and tables to increase visibility of these data. Extended Data figures and tables are online-only (present in the online PDF and full-text HTML versions of the paper), peer-reviewed display items that provide essential background to the article but are not included in the main article due to space constraints. A maximum of ten Extended Data display items (figures and tables) is permitted.

While we encourage the use of color in preparing figures, please note that this will incur a charge to partially defray the cost of printing. Information about color charges can be found at http://www.nature.com/nsmb/authors/submit/index.html#costs

Nature Structural & Molecular Biology is committed to improving transparency in authorship. As part of our efforts in this direction, we are now requesting that all authors identified as 'corresponding author' on published papers create and link their Open Researcher and Contributor Identifier (ORCID) with their account on the Manuscript Tracking System (MTS), prior to acceptance. This applies to primary research papers only. ORCID helps the scientific community achieve unambiguous attribution of all scholarly contributions. You can create and link your ORCID from the home page of the MTS by clicking on 'Modify my Springer Nature account'. For more information please visit please visit www.springernature.com/orcid.

Please use the link below to submit your revised manuscript and related files:

Link Redacted

Note: This URL links to your confidential home page and associated information about manuscripts you may have submitted, or that you are reviewing for us. If you wish to forward this email to co-authors, please delete the link to your homepage.

We look forward to seeing the revised manuscript and thank you for the opportunity to review your work.

Sincerely,

Dimitris Typas
Senior Editor
Nature Structural & Molecular Biology
ORCID: 0000-0002-8737-1319

Reviewers' Comments:

Reviewer #1 (Remarks to the Author):

The manuscript "Alternative splicing fine-tunes modular protein interfaces to control protein localisation" by Peter Kjer-Hansen et al. proposes a comprehensive study of the impact of alternative splicing on protein-protein interaction (PPI) network rewiring and protein cellular localisation. The study is at the cross-roads of high-throughput wet lab experiments, statistical analyses of networks, protein sequence analysis and molecular modeling. The topic is of utmost importance and the findings are novel. The results could be a great resource for researchers working on alternative splicing and proteins. The authors focus more specifically on the case study SNX2 with complementary in silico calculations and experimental validations.

My main concern is that the interface-resolved PPI network, which is central to the study, is not described in sufficient detail and is not usable by the community. Table S1 is insufficient and does not provide residue-level nor interface-level information about the network. The network was built by integrating diverse data sources. Such integration is in principle not trivial and the authors should describe the algorithms and choices they made. How did they map the different data sources? Are the interfaces resolved at the amino acid level? What are the criteria for determining that an AS event is altering an interaction? Does it suffice for the considered exon to be overlapping with the interface? What is the distribution of overlaps?

I also have concerns on specific points:

- The percolation analysis of the network is interesting but somewhat difficult to understand. The authors give take-home messages without actually explaining the plots and the measures used. How is the reader supposed to interpret the sigmoidal shape of the delta entropy curve? What is the formal definition of ΔE? Moreover, many observations seem weak (see panel 1G for instance, or some p-values for enrichment); I would recommend that the authors be more nuanced about the findings in those cases. "Empirical two-sided p-values were then calculated." is not sufficient: how were the p-values computed exactly? How are observed versus randomised values compared?

- What is the tissue regulation of the micro-exons? These exons were initially identified as up- or down-regulated in the nervous system at certain developmental stages. How does this relate to this analysis? Have the authors taken these aspects into consideration or only the exon length?

- There is a discrepancy between the Results and Methods sections about the usage of AlphaFold, is it version 2 or 3?

The 12 and 21 lengths are not the only ones enriched in panel 3A. The authors should clarify to what they compare these values in their interpretation. It seems the enrichment is relevant with respect to values < 12 and in between. Higher values have another interpretation.

- For the SNX2 case study, why did the authors focus on PC2? PC1 explains a much higher variance and the clustering between +EX and -EX along this direction is far from clear. Could they comment on this point? For instance, the K/A +EX mutants appear as outliers on the far left of the plot. Isn't it contradictory that the micro-exon composition does not play a role while the charges around the micro-exon are determinant for binding? In the 3D modeling, the authors consider only the monomer and focus on intra-molecular interactions between two helices. What is the rationale for doing so while the study emphasises inter-protein interfaces? What are the interfaces in this protein? What is the role of the micro-exons in these interfaces?

- Could the authors comment on the fact that the fraction of interfaces altered by AS is very small? Is it because some data are lacking or is the observed fraction a realistic estimate?

Reviewer #1 (Remarks on code availability):

I briefly reviewed the code. I could not find sufficiently detailed information on the construction of the PPI network nor on its content.

Reviewer #2 (Remarks to the Author):

AS leads to the creation of different protein isoforms, however in many/most cases the functional consequences of protein isoform variation derived from AS is unknown. This manuscript addresses the issue of functional divergence between AS-derived protein isoforms using a systems approach. They focus experimentally on identifying differences in subcellular localization of different splice isoforms, either containing, or lacking, a specific exon. The authors have used high content imaging-based screening of cells transiently transfected to express different splice isoforms and identify the cellular localization of different protein isoforms. They report altered subcellular localization between isoforms correlates with changes in protein interaction partners. In follow on analysis, SNX2 was used as an example to highlight what effect the presence or absence of a 4 residue microexon has on the alpha-helical structure, cellular localization and protein interactions. Surprisingly, they report that insertion of a 4 residue microexon in SNX2 was more important than its amino acid composition.

This study addresses an interesting problem and does so using an innovative strategy. The subject area of the study, is, in my opinion, suited to Nature Structural & Molecular Biology. However, I found this manuscript very difficult to follow and in its current format it is not likely to be accessible or easy to understand by the broad readership of NSMB. At a bare minimum, it would benefit from revision to improve the clarity of presentation and the inclusion of a visual abstract summarizing the main findings would be very helpful for the reader.

In addition, I had a number of technical questions about the experimental design used and assumptions being made, which have implications for the conclusions the authors want to draw. I am not clear about what quality control for the overall workflow was used to validate reproducibility of the results. A major part of the experimental evidence presented makes use of a high content, fluorescence imaging approach using transient transfection of constructs to express and compare different fluorescent-tagged protein isoforms. How are these data controlled for variation in protein expression and/or modification levels between the respective protein isoforms? For example, it is well known that localisation of FP-tagged proteins will be distorted relative to endogenous counterparts if their expression level (copy number) is not matched in stoichiometry by copy numbers of endogenous interaction partner proteins. How do the authors know that the effects they are measuring reflect genuinely meaningful structural consequences resulting from AS, as opposed to differences affecting protein half-lives, copy number and/or post translational modification, all of which can in turn alter subcellular localisation? While the authors state that an orthogonal validation of the localisation screen was performed, it is not clear that such validation was really performed in a comprehensive, unbiased way, as opposed to showing some cherry-picked examples showing positive results (NUMB and ANK2). How many examples are not consistent? For the AP-MS used to identify the protein interactomes of the SNX2 isoforms, no details seem to be provided concerning either mass spectrometry analysis or data searching/analysis, so I cannot evaluate the validity of the AP-MS results shown. The authors should upload the raw and processed MS data to an open, online database archive, such as PRIDE, to allow other researchers to download and verify the data.

Specific Points:

Figure S14 B clearly shows that the overall expression levels of the SNX2 -EX (granularity increased) is always higher than the expression of SNX2 +EX (lower levels of granularity) independently of the of the presence or absence of endogenous SNX2.

How did the authors achieve the same expression levels for both the SNX2 +EX and SMNX2 -EX in figure 4 A-B and S14 D and F?

SNX2 +EX and SNX2 -EX are compared to endogenous SNX2 levels in HeLa cells (Figure S14D and F). Does endogenous incorporate all SNX2 isoforms expressed in HeLa cells. Is there anything known about the expression individual SNX2 splice isoforms in HeLa cells?

Figure 5A: For a better understanding of where the SNX2 aggregates are located within the cell, it would be good to highlight the outline of the nucleus or show merged images of cell body/SNX2 staining cells.

Figure 5A and Figure S14D: It looks like the mCherry tag is affecting the localization of SNX2 -Ex.

Localization of SNX2+EX without mCherry tag in SNX2 KO cells (Figure 5A): SNX2 +EX shows a granular appearance mainly concentrated around the nucleus but also detected in the nucleus, albeit at lower levels when compared the SNX2 -

EX. Localization of SNX2+Ex with mCherry tag in SNX2 KO cells Figure S14D and F: mCherry SNX2+Ex is very much evenly distributed throughout the cytoplasm excluding the nucleus.
This raises the question whether it would have been possible to pick up any differences in the localization of SNX2-EX and SNX2+EX in a high content screen without tags and how much the mCherry tag contributes to the observed changes in the localization of different isoforms?

Reviewer #3 (Remarks to the Author):

This manuscript by Kjer-Hanset et al. explores the contribution that alternative splicing makes to protein interaction interfaces and protein localization. Overall the work is solid and the claims are in line with the evidence. A few points below would help strengthen the manuscript:

1. The analyses in Figures 1-2 are performed on highly-selective source data that will undoubtedly bias the resulting conclusions. For the most part these biases are acknowledged, but a more explicit discussion of these points would be in order. For example, by only studying exons that overlap mapped interaction sites, the results will be biased toward over-estimating interaction effects. On the other hand, they will miss potential roles for 'allosteric' splicing regulation where interaction effects occur due to distant amino acid changes. Also, by only including frame-preserving splicing changes, any frame-altering splicing events that might change interaction or localization properties will be missed.

2. Figure 1, in particular panels C and onward, would benefit from better presentation to allow the network-mathematical approaches to be more readily understood and interpreted by a structure and molecular biology reading audience.

3. It might be interesting to know: do interaction-affecting or localization-affecting AS events tend to occur e.g. near the beginning of a transcript, or in the middle, or at the 3' end?

Reviewer #4 (Remarks to the Author):

I co-reviewed this manuscript with one of the reviewers who provided the listed reports. This is part of the Nature Structural & Molecular Biology initiative to facilitate training in peer review and to provide appropriate recognition for Early Career Researchers who co-review manuscripts.

Version 2:

Decision Letter:

Our ref: NSMB-A52122B

10th Jun 2026

Dear Dr. Weatheritt,

Thank you for submitting your revised manuscript "Alternative splicing fine-tunes modular protein interfaces to control protein localisation" (NSMB-A52122B). It has now been seen by the original referees and their comments are below. The reviewers find that the paper has improved in revision, and therefore we'll be happy in principle to publish it in Nature Structural & Molecular Biology, pending minor revisions to satisfy the referees' final requests and to comply with our editorial and formatting guidelines.

We are now performing detailed checks on your paper and will send you a checklist detailing our editorial and formatting requirements in a couple of weeks. Please do not upload the final materials and make any revisions until you receive this additional information from us.

To facilitate our work at this stage, it is important that we have a copy of the main text as a word file. If you could please send along a word version of this file as soon as possible, we would greatly appreciate it; please make sure to copy the NSMB account (cc'ed above).

Thank you again for your interest in Nature Structural & Molecular Biology Please do not hesitate to contact me if you have any questions.

Sincerely,

Dimitris Typas
Senior Editor
Nature Structural & Molecular Biology
ORCID: 0000-0002-8737-1319

Reviewer #1 (Remarks to the Author):

The authors have satisfactorily addressed my concerns, thank you.

Reviewer #1 (Remarks on code availability):

I reviewed the files and documentation, they seem ok, but I did not run the code.

Reviewer #2 (Remarks to the Author):

The authors have responded to the various comments made by all three reviewers and substantially rewritten their manuscript. The revised manuscript has added considerable new data and detailed explanations, as well as clarifying a number of points that were not clear or well documented in the original version. As a result of these revisions, I judge that the study is much more accessible to a broad readership and the manuscript is now easier to read and understand. The key findings include novel data exploring how
experimentally defined protein contact sites may have their structure modulated via tissue-specific alternative splicing mechanisms, with potentially distinct roles for longer exons and microexons. I think these data will of general interest to other researchers in the field.

Reviewer #3 (Remarks to the Author):

The manuscript is now improved in clarity/interpretability and I believe is suitable for publication.

Reviewer #3 (Remarks on code availability):

Acceptable

Reviewer #4 (Remarks to the Author):

I co-reviewed this manuscript with one of the reviewers who provided the listed reports. This is part of the Nature Structural & Molecular Biology initiative to facilitate training in peer review and to provide appropriate recognition for Early Career Researchers who co-review manuscripts.

INTERNAL USE ONLY AIP modules required: ["Clinical","Life Science","Chemistry"]

Version 3:

Decision Letter:

3rd Aug 2026

Dear Dr. Weatheritt,

We are now happy to accept your revised paper "Alternative splicing fine-tunes modular protein interfaces to control protein localisation" for publication as an Article in Nature Structural & Molecular Biology.

Acceptance is conditional on the manuscript's not being published elsewhere and on there being no announcement of this work to the newspapers, magazines, radio or television until the publication date in Nature Structural & Molecular Biology.

Over the next few weeks, your paper will be copyedited to ensure that it conforms to Nature Structural & Molecular Biology style. Once your paper is typeset, you will receive an email with a link to choose the appropriate publishing options for your paper and our Author Services team will be in touch regarding any additional information that may be required.

After the grant of rights is completed, you will receive a link to your electronic proof via email with a request to make any corrections within 48 hours. If, when you receive your proof, you cannot meet this deadline, please inform us at rjsproduction@springernature.com immediately.

You will not receive your proofs until the publishing agreement has been received through our system.

Due to the importance of these deadlines, we ask that you please let us know now whether you will be difficult to contact over the next month. If this is the case, we ask you provide us with the contact information (email, phone and fax) of someone who will be able to check the proofs on your behalf, and who will be available to address any last-minute problems.

To assist our authors in disseminating their research to the broader community, our SharedIt initiative provides all co-authors with the ability to generate a unique shareable link that will allow anyone (with or without a subscription) to read the published article. Recipients of the link with a subscription will also be able to download and print the PDF.

As soon as your article is published, you can generate your shareable link by entering the DOI of your article here: http://authors.springernature.com/share. Corresponding authors will also receive an automated email with the shareable link

Note the policy of the journal on data deposition: http://www.nature.com/authors/policies/availability.html.

Your paper will be published online soon after we receive proof corrections and will appear in print in the next available issue. You can find out your date of online publication by contacting the production team shortly after sending your proof corrections.

You may wish to make your media relations office aware of your accepted publication, in case they consider it appropriate to organize some internal or external publicity. Once your paper has been scheduled you will receive an email confirming the publication details. This is normally 3-4 working days in advance of publication. If you need additional notice of the date and time of publication, please let the production team know when you receive the proof of your article to ensure there is sufficient time to coordinate. Further information on our embargo policies can be found here: https://www.nature.com/authors/policies/embargo.html

You can now use a single sign-on for all your accounts, view the status of all your manuscript submissions and reviews, access usage statistics for your published articles and download a record of your refereeing activity for the Nature journals.

If you have not already done so, we strongly recommend that you upload the step-by-step protocols used in this manuscript to the Protocol Exchange. Protocol Exchange is an open online resource that allows researchers to share their detailed experimental know-how. All uploaded protocols are made freely available, assigned DOIs for ease of citation and fully searchable through nature.com. Protocols can be linked to any publications in which they are used and will be linked to from your article. You can also establish a dedicated page to collect all your lab Protocols. By uploading your Protocols to Protocol Exchange, you are enabling researchers to more readily reproduce or adapt the methodology you use, as well as increasing the visibility of your protocols and papers. Upload your Protocols at www.nature.com/protocolexchange/. Further information can be found at www.nature.com/protocolexchange/about.

An online order form for reprints of your paper is available at https://www.nature.com/reprints/author-reprints.html. Please let your coauthors and your institutions' public affairs office know that they are also welcome to order reprints by this method.

Authors may need to take specific actions to achieve compliance with funder and institutional open access mandates. If your research is supported by a funder that requires immediate open access (e.g. according to Plan S principles or the NIH public access policy) then you should select the gold OA route, and we will direct you to the compliant route where possible. Because authors warrant under our subscription licensing terms that they haven't committed to licensing any version of their article under a licence inconsistent with the terms of our agreement – including the applicable embargo period – publication under the subscription model isn't suitable for authors whose funders require no embargo.

In approximately 10 business days you will receive an email with a link to choose the appropriate publishing options for your paper and our Author Services team will be in touch regarding any additional information that may be required.

You will not receive your proofs until the publishing agreement has been received through our system.

If you have any questions about our publishing options, costs, Open Access requirements, or our legal forms, please contact ASJournals@springernature.com

Sincerely,

Dimitris Typas
Senior Editor
Nature Structural & Molecular Biology
ORCID: 0000-0002-8737-1319

Click here if you would like to recommend Nature Structural & Molecular Biology to your librarian:

http://www.nature.com/subscriptions/recommend.html#forms

Open Access This Peer Review File is licensed under a Creative Commons Attribution 4.0 International License, which permits use, sharing, adaptation, distribution and reproduction in any medium or format, as long as you give appropriate credit to the original author(s) and the source, provide a link to the Creative Commons license, and indicate if changes were made.
In cases where reviewers are anonymous, credit should be given to 'Anonymous Referee' and the source.
The images or other third party material in this Peer Review File are included in the article's Creative Commons license, unless indicated otherwise in a credit line to the material. If material is not included in the article's Creative Commons license and your intended use is not permitted by statutory regulation or exceeds the permitted use, you will need to obtain permission directly from the copyright holder.
To view a copy of this license, visit https://creativecommons.org/licenses/by/4.0/

Explanation of the text style/colour:

- Reviewer comments.
- **Author responses.**
- Text that has been added to the manuscript.

Reviewer 1 Comment 1: My main concern is that the interface-resolved PPI network, which is central to the study, is not described in sufficient detail and is not usable by the community. Table S1 is insufficient and does not provide residue-level nor interface-level information about the network. The network was built by integrating diverse data sources. Such integration is in principle not trivial and the authors should describe the algorithms and choices they made.
How did they map the different data sources?
Are the interfaces resolved at the amino acid level?
What are the criteria for determining that an AS event is altering an interaction?
Does it suffice for the considered exon to be overlapping with the interface?
What is the distribution of overlaps?

We thank the reviewer for this comment and apologise that the original manuscript had insufficient details. We also fully agree that the data should be fully usable by the community. We have therefore substantially expanded the methodological detail on the interface-resolved interactome and improved usability of the data. We also apologise that the script used to generate the network was not included in the original GitHub repository. This has now been added, together with documentation of the required input format and processing steps (https://github.com/weatheritt2/Hansen_AS).

We have substantially expanded the Methods and Supplementary Materials to clarify how the interface-resolved network was constructed, how diverse data sources were harmonised, how alternative splicing events were mapped onto interfaces, and what criteria were used to classify an interaction as potentially altered by splicing.

Specifically, we now state that the network was assembled by integrating interface annotations from UniProt, Interactome3D, DOMINO, IntAct, 3DID, and ELM. Records from all sources were harmonised to a common coordinate framework by mapping proteins to UniProt canonical accessions and residue numbering. Where multiple resources described the same contact, redundant records were merged while preserving source provenance. The resulting network therefore represents protein–protein interactions with database-derived interface annotations, supported by structural and curated experimental evidence, mapped onto UniProt protein coordinates rather than as binary associations alone. An AS event was therefore classified as "interface-altering" when the protein region encoded by the regulated exon overlapped at least one annotated interface residue or interface interval for that

interaction. We used this permissive binary criterion to avoid excluding short interface elements, such as motif-based contacts, that could be affected by partial exon overlap. This choice was also motivated by previous work, including *Irimia et al. 2014*, showing that exon inclusion can affect protein binding even when the exon is adjacent to, rather than directly overlapping, the interaction interface.

The following section have now been added to the methods:

Construction of the interface-resolved PPI network.

Protein–protein interactions were mapped to physical contact interfaces using curated evidence from UniProt[23], Interactome3D[26], 3DID[27], Domino[24], IntAct[25], and ELM[28]. Records from all sources were harmonised to a common coordinate system by mapping proteins to UniProt canonical accessions and residue numbering. Redundant records describing the same interaction interface were merged while preserving source provenance and annotation type. The resulting interface-resolved interactome represents each edge as a specific annotated contact between two proteins (node), rather than a binary protein association alone, with mapped interface residues or residue intervals recorded for both partners where available. Note, Interactome3D and 3DID were grouped and referred to as `3DID` as they were created by the same group.

Definition of AS-driven interface alteration.

Alternative splicing events were projected from transcript coordinates to protein coordinates. An event was classified as interface-altering when the protein region encoded by the regulated exon overlapped at least one annotated interface residue or mapped interface interval for a given interaction. Primary analyses used a permissive binary criterion of any overlap of at least one residue, based on results prior AS interactome studies. In addition, overlap extent was quantified using fractional overlap metrics, including the proportion of interface residues overlapped by the exon and the proportion of exon-encoded residues overlapping the interface.

We agree, however, that the extent of overlap is important, and we therefore now provide the distribution of overlap sizes and overlap fractions across the dataset (Rebuttal Figure 1, included as Figure S1B-C in manuscript), together with a breakdown by source database (Rebuttal Figure 2, included as Figure S1D in manuscript).

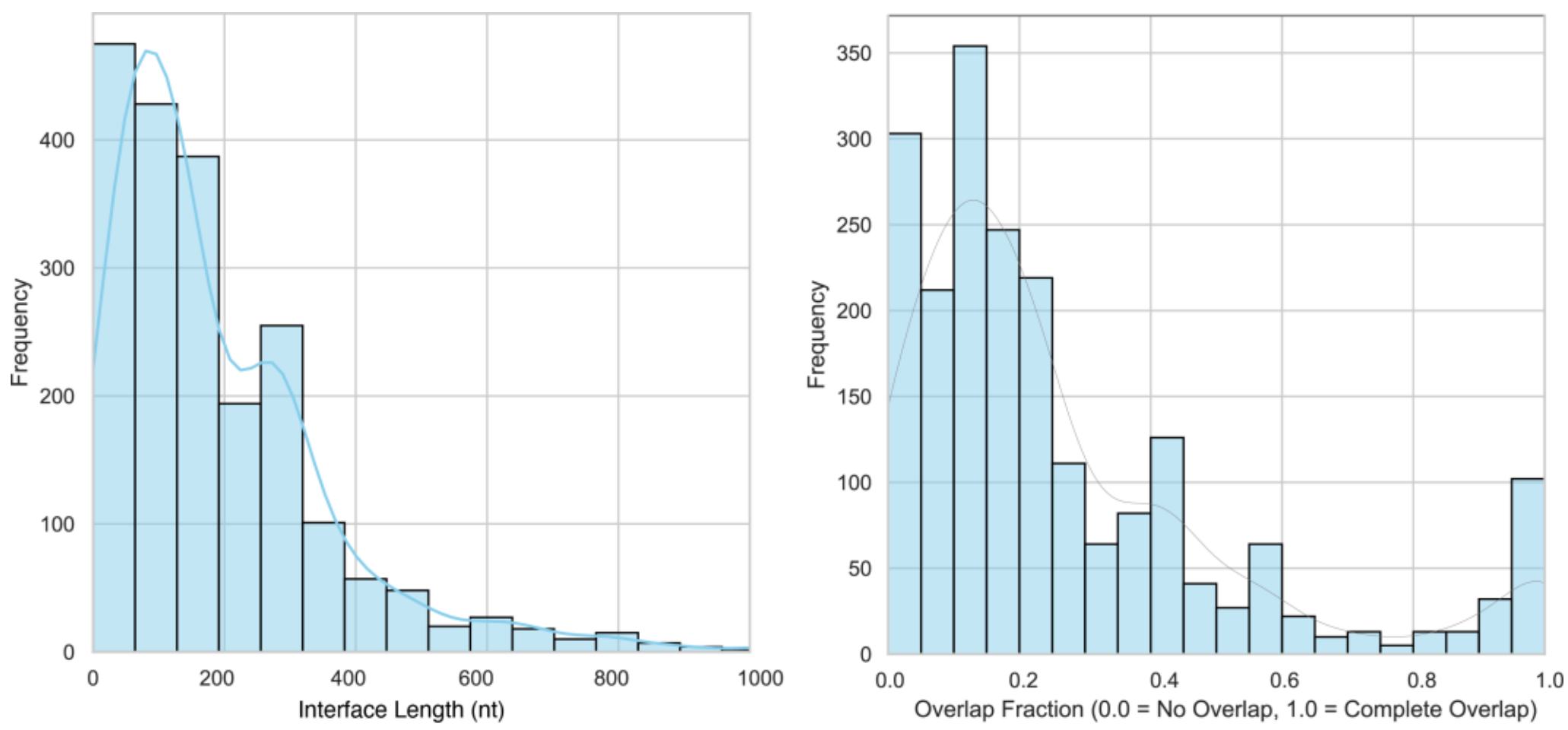

Rebuttal Figure. 1 | Distribution of interface lengths and exon–interface overlap fractions. Left, histogram showing the length distribution of annotated protein interaction interfaces overlapping alternative exons. Right, histogram showing the fraction of each interface overlapped by the alternative exon region, where 0 indicates no overlap and 1 indicates complete overlap. Curves indicate kernel density estimates

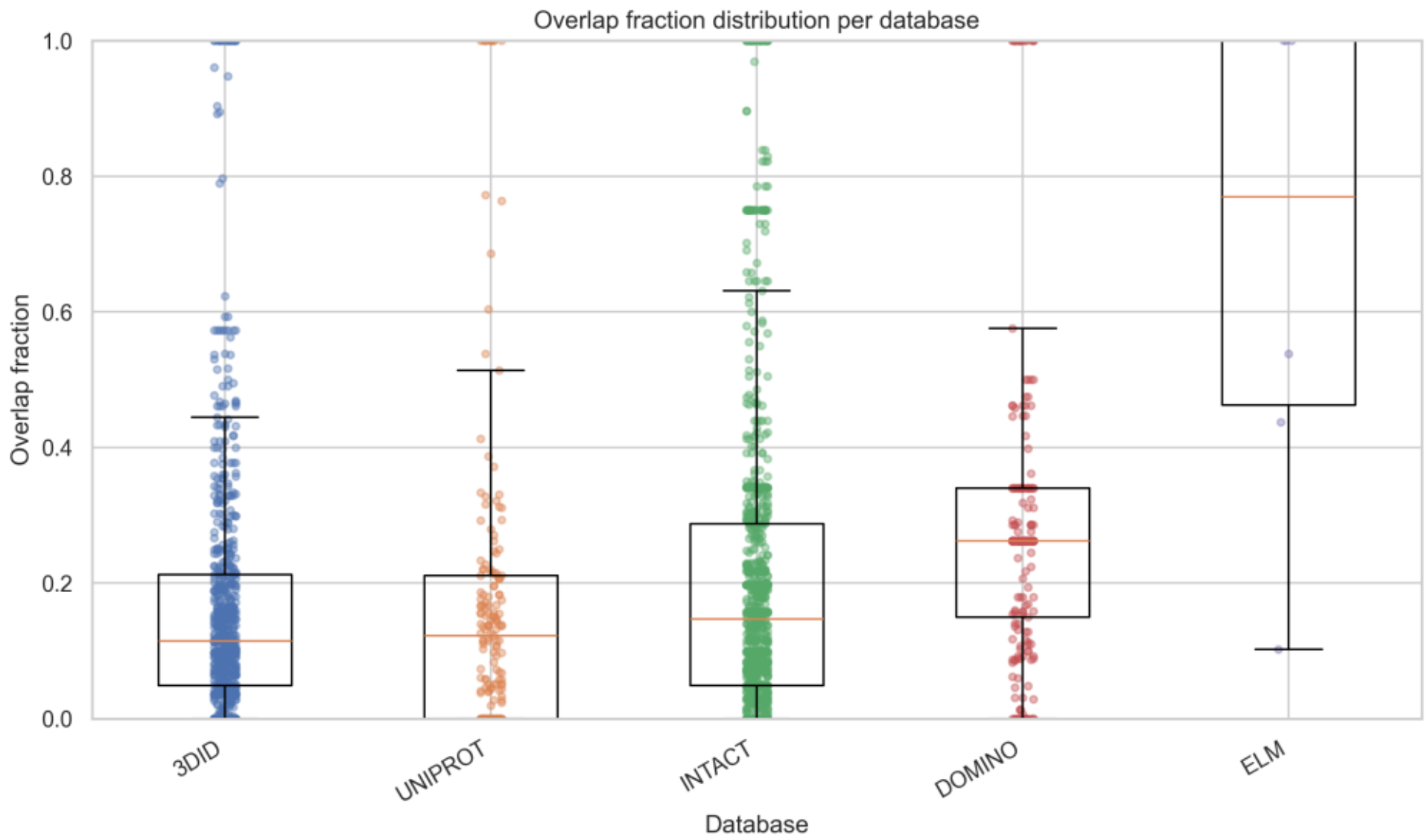

Rebuttal Figure. 2 | Overlap fraction between alternative exons and annotated interaction interfaces across databases. Boxplots showing the distribution of overlap fractions between alternative exon regions and annotated interaction interfaces from 3DID*, UniProt, IntAct, DOMINO and ELM. Points represent individual exon–interface overlaps. Overlap fraction was calculated as the proportion of the annotated interface region overlapped by the alternative exon, where 0 indicates no overlap and 1 indicates complete overlap. Centre lines indicate medians, boxes indicate the interquartile range, and whiskers extend to 1.5× the interquartile range. Note, 3DID* contains annotation from both 3DID and Interactome3D as the databases were created by the same lab.

These new analyses show that overlap fractions differ systematically by database. In particular, ELM and DOMINO tend to show higher overlap fractions than 3DID, UniProt and IntAct, consistent with their enrichment for short linear motifs and other short interaction elements. By contrast, interfaces annotated in structured-domain resources are often more extended, such that alternative exons more commonly overlap only part of the interaction surface. This likely reflects the fact that interfaces in structured domains are often distributed across folded regions, such that complete exon-mediated removal of the interface would frequently be expected to compromise domain stability.

To improve reusability of the interactome network itself, we have replaced the previous supplementary Table S1 with an expanded table that denotes the interactors: *gene_a, gene_b,* and describes the interaction interface in *gene*_a. We specifically include columns that specify the interface start and end coordinates (in the UniProt

amino acid sequence), annotation details for the interface (e.g. interpro domain name), and what interaction database the information comes from. We further indicate whether the interface is overlapped by an AS event. When this is the case, we provide the exon ID in vastDB, the exon length in nucleotides, the start and end amino acid position encoded by the exon (when projected onto the UniProt coordinates), and the tissue-specificity metric for the AS event used in percolation analysis. We believe these additions now make both the construction and the downstream use of the interface-resolved interactome substantially more transparent.

To describe these results, we have added the following text to the Results section of the manuscript:

To investigate how alternative splicing (AS) reshapes protein interactions at the level of mapped interaction interfaces, we constructed an interface-resolved human interactome by integrating curated interface annotations from UniProt[23], DOMINO[24], IntAct[25], 3DID[26,27] and ELM[28]. In this network, each node represents a protein, and each edge represents an experimentally supported protein-protein interaction where the interface region was mapped onto UniProt canonical sequences (**Figure 1A**). This network comprised 17,660 experimentally defined interfaces (**Table S1**), providing a detailed framework for assessing how exon choice influences protein connectivity. Integrating differential splicing analysis[29,30] across 75 tissues and cell types, (delta percent spliced in (dPSI) > 20) we identified frame-preserving tissue-regulated AS events whose encoded protein regions overlapped at least one mapped interface residue or interval.

__**Reviewer 1 Comment 2:**__ The percolation analysis of the network is interesting but somewhat difficult to understand. The authors give take-home messages without actually explaining the plots and the measures used.
How is the reader supposed to interpret the sigmoidal shape of the delta entropy curve?
What is the formal definition of ΔE?
Moreover, many observations seem weak (see panel 1G for instance, or some p-values for enrichment); I would recommend that the authors be more nuanced about the findings in those cases.
"Empirical two-sided p-values were then calculated." is not sufficient: how were the p-values computed exactly?
How are observed versus randomised values compared?

Many thanks for this comment we have extensively rewritten the Results and Methods sections on the percolation analysis and provided a visual schema (Rebuttal Figure 3, included as Figure 1C in manuscript) to both more clearly explain the approaches used, the plots and the methods to address this point (see updated manuscript for full changes).

The visual schema is:

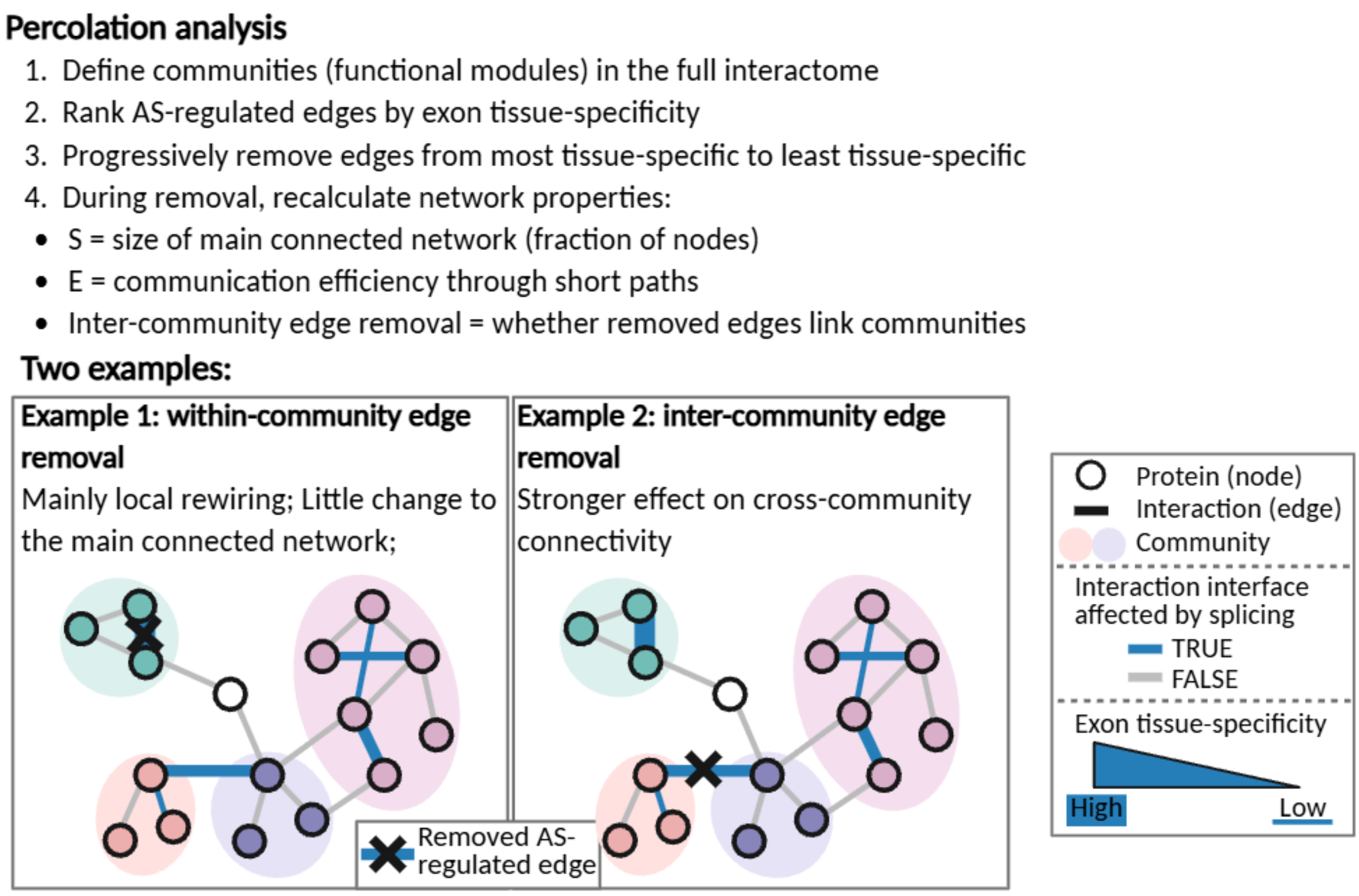

Rebuttal Figure. 3 | Overview of the percolation analysis used to examine the interaction network and the impact of alternative splicing events overlapping interaction interfaces on network wiring. Communities refer to densely connected proteins and therefore often represent functional modules, such as proteins in the same pathway. AS-regulated edges were ranked by tissue-specificity and progressively removed, and network properties were recalculated at each step. Two examples illustrate removal of an intra-community edge versus an inter-community edge.

To specifically answer the posed questions, we have now provided a clear explanation for how the reader is supposed to interpret the sigmoidal shape of the delta entropy curve:

The sigmoidal shape of the ΔS trajectory suggests that the effect of progressive edge removal is not uniform across the ranked AS events: early removal of the most tissue-specific edges has relatively little effect on the main connected part of the interactome, whereas later removal produces a sharper transition once a more critical set of integrative edges is affected.

The formal definition of ΔE is now written in the methods section as follows:

we calculated E(f), the approximate global efficiency of the residual graph, following the approach introduced by Latora and Marchiori[111], which measures how efficiently proteins can still reach one another through short paths after edge removal. Because exact all-pairs global efficiency is computationally expensive for repeated percolation analyses, we estimated this quantity by averaging inverse shortest-path distances over a sampled set of source nodes at each removal step. Let Q_f denote the set of source nodes used at step f (all nodes if the graph contained less than or equal to 200 nodes, otherwise a random sample of 200 source nodes), and let $d_{(H\_f\}(u,v)}$ denote the shortest-path distance between nodes u and v in H_f. Approximate global efficiency was calculated as:

$$E(f) = \frac{1}{R_f} \sum_{u \in Q_f} \sum_{\substack{v \in V(H_f) \\ v \neq u,\ d_{H_f}(u,v) < \infty}} \frac{1}{d_{H_f}(u,v)}$$

where R_f is the number of reachable ordered source–target pairs included in the calculation and Q_f is sampled set of source nodes. Lower values of E(f) indicate that short communication paths are being lost, even if many proteins remain in the same main connected part of the network.

We have also formally defined how the empirical p-value was calculated and described how the observed versus randomised values compared:

In the Percolation section of the Methods, we added:

To assess whether the observed percolation trajectory differed from random expectation, we generated 1,000 null trajectories. In the degree-matched null model used here, all network edges were first grouped into bins according to the minimum degree of their two endpoint nodes in the original graph. For each null replicate and each removal step k, we randomly removed edges from these degree bins in proportion to the number of observed AS-regulated edges removed at that step. This produced null removal sets matched to the endpoint-degree profile of the observed removal set, while preserving the total number of removed edges at each step.

For each metric $M\ in\ \{S, E\}$ the observed trajectory $M_{AS(f)}$ was compared with the null distribution $f\ \{M_{(null,r)(f)}\}_{r=1}^{1000}$ at the same removal fractions. We summarised the null distribution at each f by its mean and 95% interval (2.5th–97.5th percentiles). We also calculated paired deviations for each replicate as:

$$f\ Delta_{r(f)} = M_{(AS)(f)} - M_{(null,r)(f)}$$

and plotted the mean and 95% interval of $delta_r(f)$ across null replicates. Thus, observed versus randomised trajectories were compared both directly, through the observed curve relative to the null mean and interval, and through these paired AS-minus-null deviations.

And in the Statistics section of the Methods, we added:

To summarise differences across the full percolation trajectory, we calculated the area under the curve (AUC) for of the 1,000 null trajectories using the trapezoidal rule. Null trajectories were generated from the same degree-matched random edge-removal procedure described above. For S(f) and E(f), empirical significance was assessed by comparing the observed AUC with the distribution of null AUC values using a one-sided lower-tail test:

$$p_{AUC} = \frac{1 + sum_{r=1}^{R} I\left(AUC_{null,r} \leq AUC_{AS}\right)}{R + 1}$$

where R=1000, AUC_{AS} is the AUC of the observed AS-guided removal trajectory, $AUC_{null,r}$ is the AUC of null replicate r, and I is the indicator function. Thus, the empirical p-value corresponds to the proportion of null replicates whose AUC is less than or equal to the observed AUC, with a +1-correction applied to both numerator and denominator. This formulation tests whether AS-guided edge removal produces a smaller AUC than expected

under random removal, corresponding to a more disruptive percolation trajectory. Reported p-values for the S and E percolation analyses therefore refer to this trajectory-level AUC comparison rather than to a separate pointwise p-value at each removal step.

We have also rewritten the Results section to address the reviewer's concerns about giving take-home messages without explaining the plots and the measures used and not being nuanced enough about findings:

Because our goal was to understand how transcript diversity translates into protein interaction rewiring across cellular contexts, we next investigated whether alternative splicing reshapes interaction topology at the systems level. To do this we examined how AS-regulated interfaces contribute to network organisation using percolation analysis and inter-community edge analysis (**Figure 1C**). In this framework, AS-regulated edges were progressively removed from the interface-resolved interactome in order of decreasing exon tissue-specificity, beginning with the most strongly tissue-regulated exons and ending with more tissue-wide events, defined here as exons detected across many tissues but still showing substantial tissue-to-tissue differences in inclusion. At each removal step, we quantified two complementary properties of the interactome network: we quantified S, the fraction of nodes remaining in the main connected part of the interactome, which captures whether the network stays largely intact rather than breaking into smaller disconnected pieces, and E, the approximate global efficiency, which captures how efficiently information can still travel across the remaining network. These observed trajectories were then compared with degree-matched random edge-removal null models to determine whether AS-regulated interfaces have non-random effects on network structure.

Removal of the most strongly tissue-specific AS edges caused a significant deviation from the degree-matched null expectation in the fraction of nodes retained in the main connected part of the interactome network, but in a direction indicating less fragmentation than expected by chance (**Figure 1D**, ΔS: empirical two-sided $p < 0.001$). The sigmoidal shape of the ΔS trajectory suggests that the effect of progressive edge removal is not uniform across the ranked AS events: early removal of the most tissue-specific edges has relatively little effect on the main connected part of the interactome, whereas later removal produces a sharper transition once a more critical set of integrative edges is affected. Together with the absence of a significant effect on approximate global efficiency (**Figure S1E**, ΔE: $p > 0.05$), this pattern is most consistent with highly tissue-specific exons preferentially rewiring interactions locally rather than a broad disruption of network-wide communication. We next examined whether AS-regulated edges preferentially connect distinct functional communities, that is, groups of proteins that are more densely connected to one another than to the rest of the interactome. Consistent with network S, tissue-specific AS-regulated edges were depleted among edges linking distinct functional communities, whereas tissue-wide AS events significantly altered the fraction of edges linking distinct communities (**Figure S1F**, empirical two-sided $p < 0.002$). This pattern suggests tissue-wide events may contribute more strongly to maintaining connections between functional modules. Taken together, these findings are consistent with a model in which tissue-specific exons mainly fine-tune local network structure, whereas more tissue-wide exons have greater influence on inter-community integration.

Among tissue-specific events, microexons (< 30 nt) represent a distinct class of AS events in terms of effect on protein structure. Unlike longer exons, microexons frequently alter the structured domains of proteins[29], suggesting that they may exert different effects on

interaction topology. We therefore tested whether microexons displayed a distinct pattern of network regulation. In contrast to the broader class of tissue-specific exons, which were associated primarily with local rewiring, microexons were significantly enriched among interfaces linking distinct functional communities (**Figure 1E; Figure S1G-H**, $p < 0.004$, empirical two-sided). This suggests that microexons preferentially regulate connections between pathways or complexes rather than acting only within local modules. Ordinary least-squares (OLS) modelling (see Methods) further showed that inter-community connectivity depended on both tissue regulation and local topology, with a significant regulation × microexon interaction term (**Figure S1I**, $p < 0.03$, OLS t-test). Together, these observations are consistent with microexons representing a specialised subset of tissue-specific exons with a disproportionate potential to occupy network positions that modulate crosstalk between different complexes or subcellular locations.

Reviewer 1 Comment 3: What is the tissue regulation of the micro-exons? These exons were initially identified as up- or down-regulated in the nervous system at certain developmental stages. How does this relate to this analysis?
Have the authors taken these aspects into consideration or only the exon length?

In the present analysis, microexons are classified based on exon length (< 30 nt). We did not additionally stratify them by the specific tissue or developmental stage in which they are included or excluded. Our logic for analysing microexons as a separate class was due to their distinct structural properties rather than on a specific neural regulatory programme (see Irimia *et al.* 2014 [PMID:25525873]). In particular, despite encoding very short protein sequences, microexons can have disproportionate effects on structured protein features and interaction interfaces (see Irimia *et al.* 2014), making them conceptually distinct from longer alternative exons. Thus, in this study, we treated microexons as a structurally distinct AS class rather than a nervous system-specific regulatory program. We do agree, however, that microexons were originally characterised as developmentally regulated in neural tissue though subsequent analysis has shown some microexons are more broadly expression.

We have revised the manuscript to make clear that our analysis focused on the interaction wiring potential associated with microexon-sized sequence changes, rather than on modelling tissue-specific state of each microexon-mediated interaction.
Among tissue-specific events, microexons (< 30 nt) represent a distinct class of AS events in terms of effect on protein structure. Unlike longer exons, microexons frequently alter the structured domains of proteins[29], suggesting that they may exert different effects on interaction topology.

Reviewer 1 Comment 4: There is a discrepancy between the Results and Methods sections about the usage of AlphaFold, is it version 2 or 3?

The 12 and 21 lengths are not the only ones enriched in panel 3A. The authors should clarify to what they compare these values in their interpretation. It seems the enrichment is relevant with respect to values < 12 and in between. Higher values have another interpretation.

We thank the reviewer for highlighting this inconsistency and agree that our description of structure prediction was unclear. We have revised the Methods to distinguish explicitly between the two sources used in this study. When structure predictions were available through the AlphaFold Protein Structure Database for UniProt-annotated sequences, we used those database predictions. For isoforms that were not represented in UniProt annotation and therefore were not directly available through AlphaFold DB (for example, exon-containing custom sequences such as the SNX2 isoform), we generated predictions using the Google Colab implementation of AlphaFold2. We have amended the Methods accordingly to remove the ambiguity between AlphaFold DB predictions and custom AlphaFold2 modelling.

Second, we agree that our original wording about exon lengths was too narrow. The distributions in Figure 3A-B do not imply that 12-nt and 21-nt microexons are the only enriched lengths. Rather, these represent prominent local peaks relative to neighbouring exon lengths within the short microexon distribution. We have revised the Results to clarify that our interpretation refers to relative enrichment within the observed length distribution, not exclusive enrichment at only these two sizes. We also now note that additional enrichment is visible at longer microexon lengths, particularly in the 24–27 nt range, which may reflect a broader aspect of the distribution rather than the same interpretation applied to the shorter local peaks.

We have added following section to the methods:

Protein structural modelling

Protein structure predictions were obtained from two sources. For UniProt-annotated protein sequences with predictions available through the AlphaFold Protein Structure Database[47] (AlphaFold DB), we used the corresponding AlphaFold DB models. Sequences for human TEAD1 and ERC1 protein isoforms were extracted from the UniProt database[23] (TEAD1: P28347-1. ERC1: Q8IUD2-1).

For isoforms or exon-containing protein sequences not represented in UniProt annotation and therefore not directly available through AlphaFold DB, we generated custom structure predictions using the Google Colab implementation of AlphaFold2. This was the case for SNX2 microexon. In which case we extracted sequences for SNX2 protein isoforms were extracted from the UniProt database[23] for human (O60749-1), rat (B2RYP4), chicken (A0A8V0X9Z2), and zebrafish (Q32LT0). Amino acid sequences corresponding to the microexons-of-interest and specific amino acid substitutions were then added/removed manually. The proteins were then modelled using AlphaFold2 v2.1 with the default input settings via Google Colaboratory[96]. The isoform models were downloaded and visualised in ChimeraX[98] v1.9. ChimeraX was also used to align, examine surface charge, and examine

electrostatic interactions (only salt bridges) of the SNX2 region corresponding to 419 – 464 of human SNX2 (Uniprot ID: O60749-1) with default input settings.

The results section has been updated as follows:

Although enrichment was also observed at 24 and 27 nt, insertions of this length are unlikely to preserve an α-helix directly, suggesting that these longer classes may arise through a partly distinct mechanism.

As well as:

This pattern was exemplified by models from the AlphaFold Protein Structure Database[47] of ERC1 and TEAD1, where microexon inclusion is predicted to either maintain helical geometry or subtly change it by forming/extending loops, respectively (**Figure 3D**).

Reviewer 1 Comment 5A: For the SNX2 case study, why did the authors focus on PC2? PC1 explains a much higher variance and the clustering between +EX and -EX along this direction is far from clear.

Could they comment on this point? For instance, the K/A +EX mutants appear as outliers on the far left of the plot. Isn't it contradictory that the micro-exon composition does not play a role while the charges around the micro-exon are determinant for binding?

We thank the reviewer for this comment. We originally focused on PC2 because the endogenous SNX2−EX and SNX2+EX isoforms separate primarily along this axis, whereas PC1, although explaining more overall variance, does not clearly distinguish the two endogenous isoforms. Moreover, PC2 was strongly aligned with granularity, the feature that most clearly captured the visible difference between the endogenous isoforms and was among the most informative features for classification. We therefore interpreted PC2 as the component most relevant to the isoform-specific localisation differences, rather than the component explaining the greatest total variance.

In response to the reviewer's concern, we re-analysed the full mutant series more systematically. To reduce redundancy among correlated image features, we clustered the isoform-distinguishing features by pairwise correlation (Rebuttal Figure 4) and retained a reduced set of representative features for PCA and downstream analysis (Rebuttal Figure 5-7). This reanalysis confirms our original conclusion that the major separation between constructs occurs principally along PC2 and is associated with granularity and intensity standard deviation, whereas PC1 is driven mainly by texture-related features.

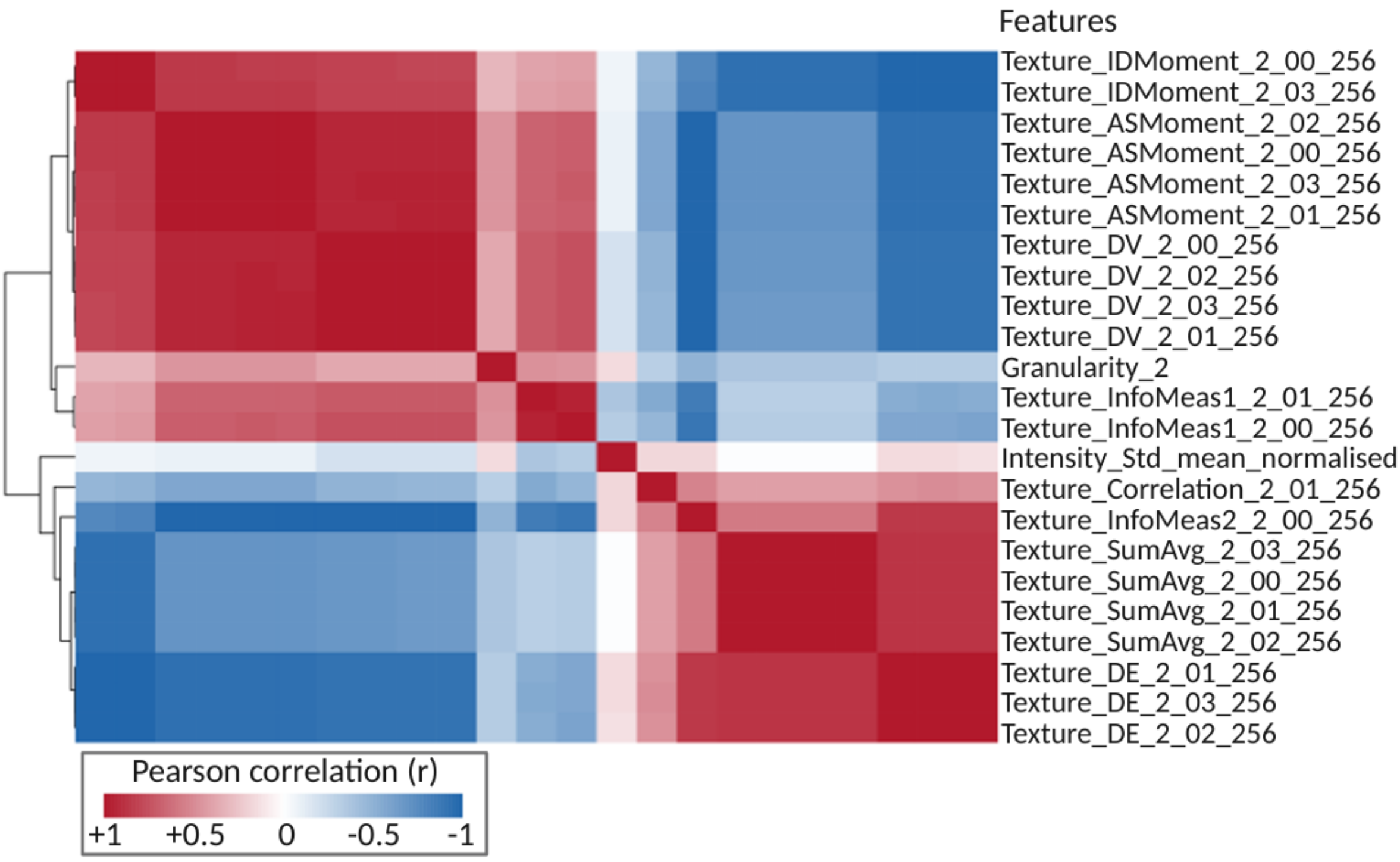

Rebuttal Figure 4 | Correlation heatmap of image-derived features. Hierarchical clustered heatmap showing pairwise correlations among the SNX2 isoform-distinguishing image features extracted from the mCherry channel, calculated on all cells positively expressing mCherry-SNX2 isoforms or mutants. Rows and columns represent the same set of features, and values are colour-coded according to the correlation coefficient, with red indicating positive correlation, blue indicating negative correlation, and white indicating weak or no correlation. Dendrograms indicate hierarchical clustering based on similarity in correlation profiles, revealing groups of co-varying features and inverse relationships between feature clusters.

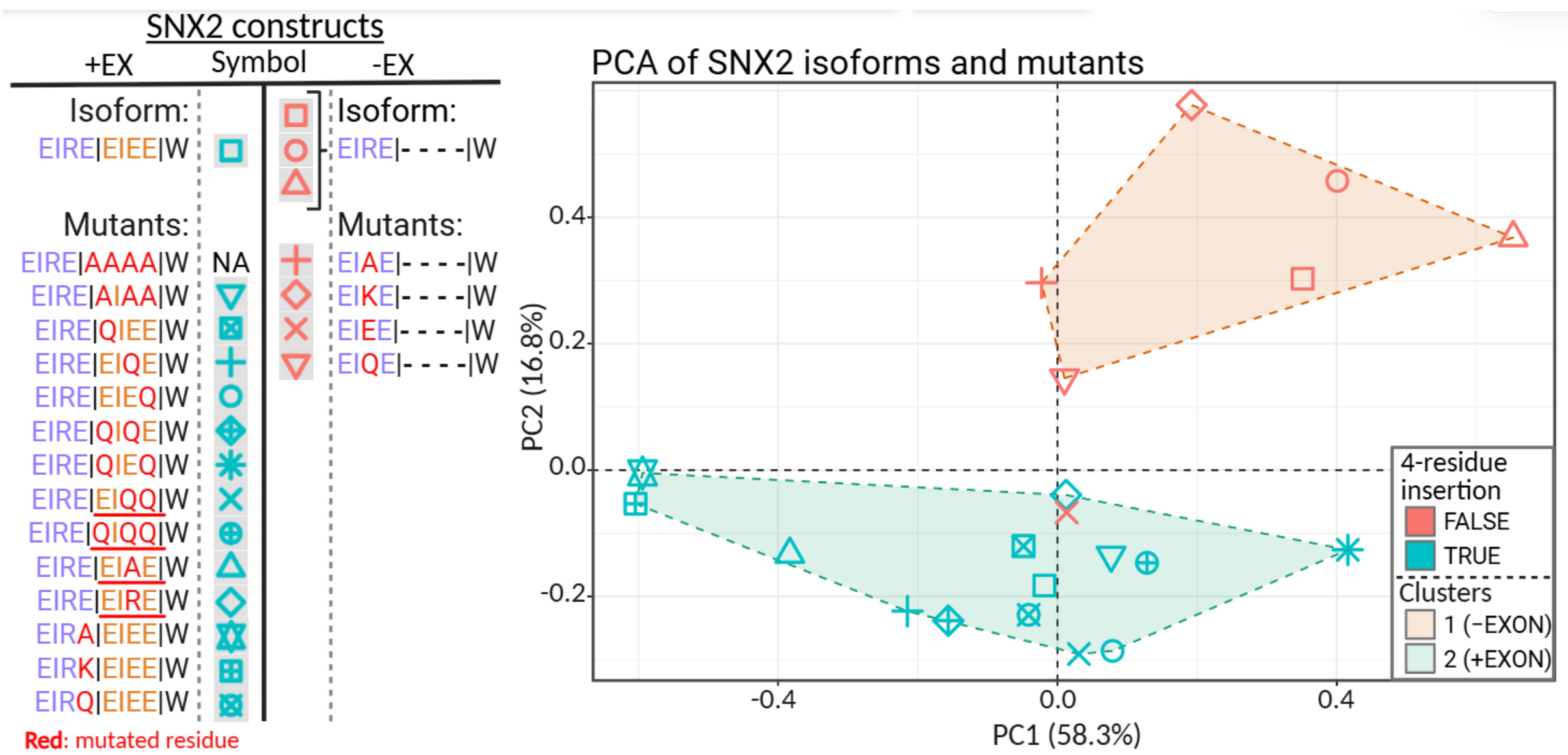

Rebuttal Figure 5 | PCA of mutations within and around the SNX2 microexon. Principal component analysis (PCA) of transfected cells positively expressing SNX2 isoforms and SNX2 mutants using the reduced SNX2 isoform distinguishing features as input. The plot shows the centroid scores for PC1 and PC2 for each construct (that is, the mean PC1 and mean PC2 calculated for all cells

expressing the given construct). Each construct is represented by a symbol (left), which are coloured according to the exon status (+EXON = cyan, -EXON = red). The constructs are clustered into two groups, based on their centroid scores of PC1-5. The two clusters are outlined by dashed polygons and have separate colours and show the constructs largely separating based on the microexon status.

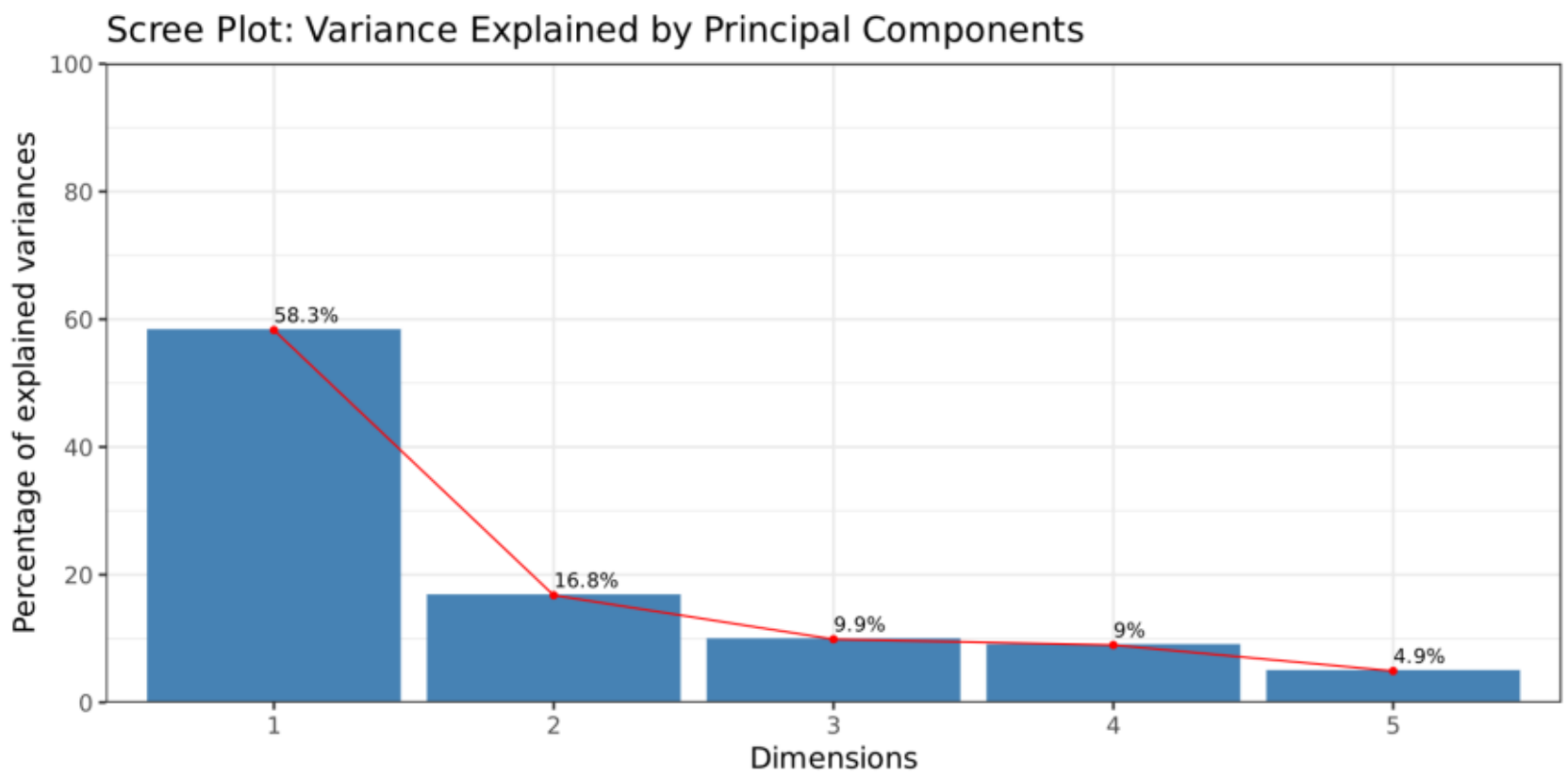

Rebuttal Figure 6 | Variance explained by principal components. Scree plot summarising the proportion of variance explained by each principal component from the PCA of centroid-level feature profiles. PC1 accounts for 58.3% of total variance and PC2 for 16.8%, together capturing 75.1% of the overall variation, with the remaining components each contributing less than 10%. This indicates that the major structure in the dataset is well represented in the first two dimensions.

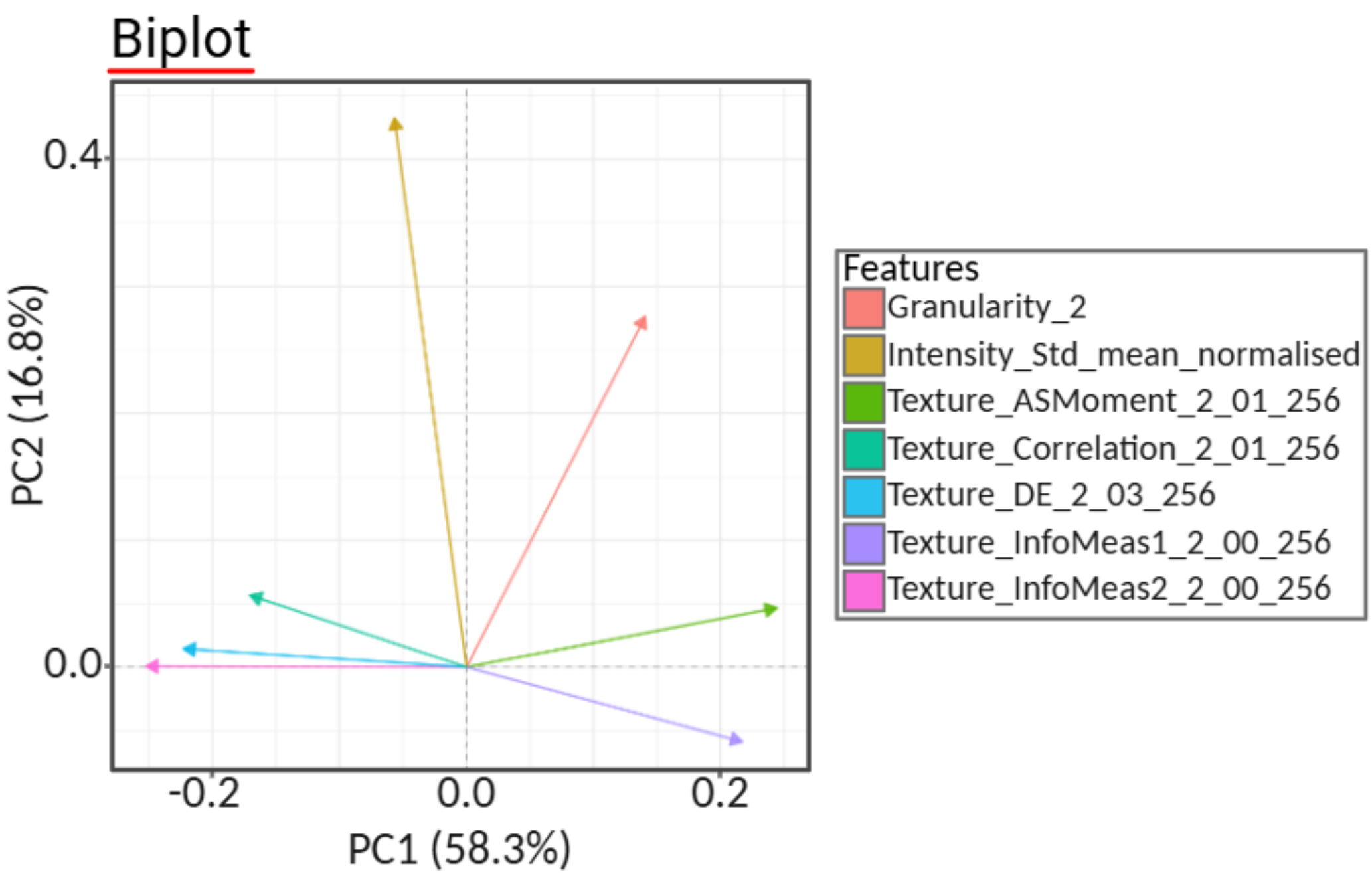

Rebuttal Figure 7 | PCA biplot showing feature loadings on the first two principal components. PCA biplot displaying the loading vectors of the selected image-derived features on PC1 and PC2. Arrows originate at the origin and indicate the direction and relative contribution of each feature to the two principal components. Features with longer arrows contribute more strongly to separation in the reduced feature space, whereas features pointing in similar directions are positively associated and those pointing in opposing directions are inversely related

Importantly, this analysis also clarifies the behaviour of the mutants. When clustering the constructs into two groups, all SNX2+EX constructs cluster together, with most +EX forming a group together except for specific charge-altering substitutions that shift their position along PC1 rather than toward the −EX cluster, which form the other cluster (Rebuttal Figure 5). Conversely, mutating the flanking residue R451 produces a graded shift of SNX2−EX constructs toward the SNX2+EX cluster, with R451E showing the strongest effect. Indeed, R451E is the only construct whose assigned cluster doesn't capture its exon status. Thus, the main localisation transition between the endogenous isoforms is better explained by the local charge context adjacent to the microexon, particularly R451, than by the internal microexon substitutions tested here.

We agree with the reviewer that the +EX E452A/K constructs are informative, and they are the constructs clustering the furthest from the main +EX group. Importantly, these mutants do not move toward the −EX state. Instead, they separate mainly along PC1, suggesting a distinct localisation phenotype rather than reversal of the endogenous isoform difference. To investigate this further we revisited the underlying microscopy images (Rebuttal Figure 8). Upon inspection, however, we found the texture-related differences between SNX2+EX and the SNX2+EX E452A/K mutants hard to single out, although it may relate to plasma membrane association. This contrasted the visually striking granularity differences between SNX2+EX and SNX2–EX constructs, where E452A/K behaves similarly to +EX.

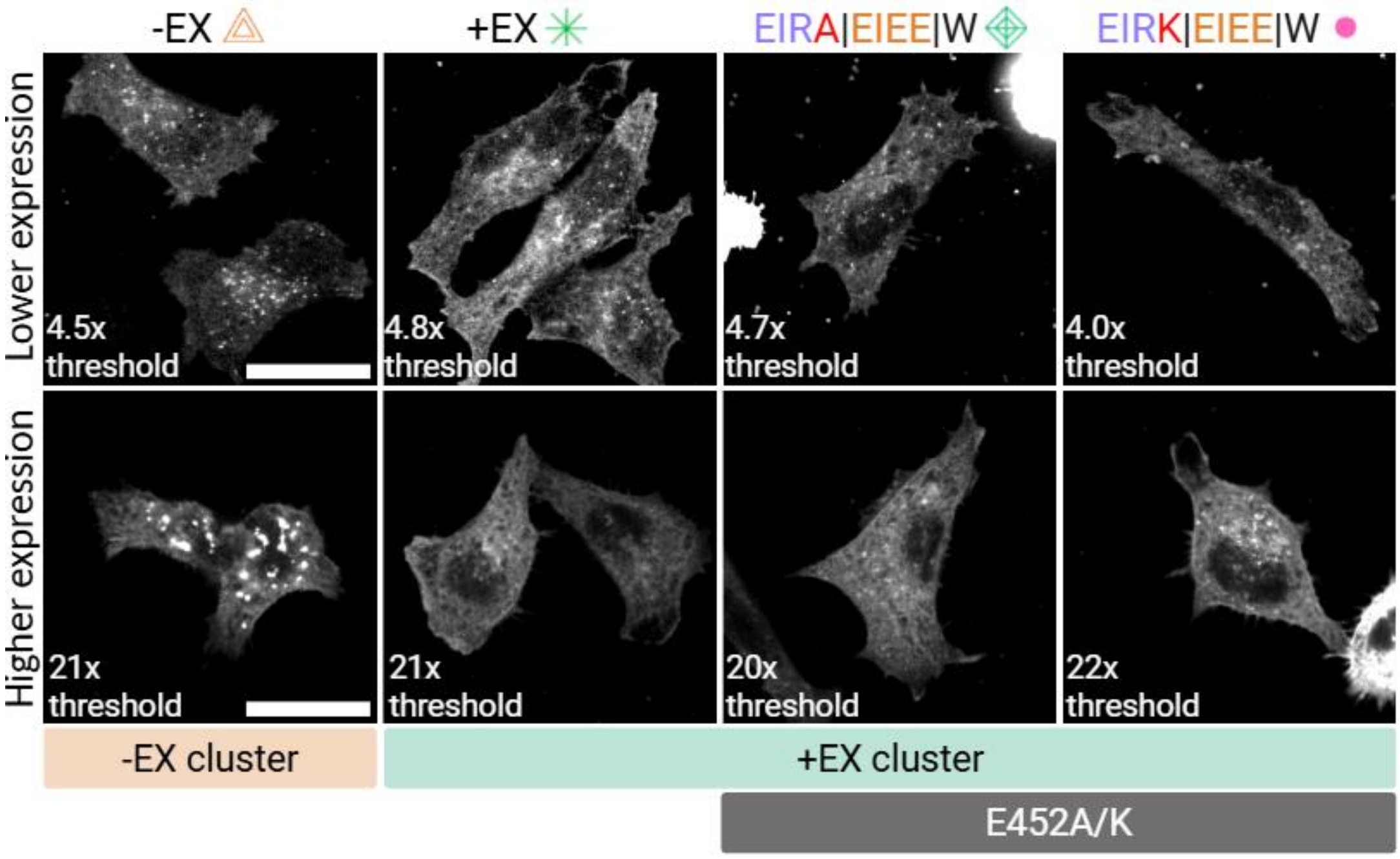

Rebuttal Figure 8 | Representative images of the SNX2 E452 isoform mutants. Representative fluorescence microscopy images of HeLa cells transiently expressing mCherry-SNX2 constructs with or without the alternatively spliced microexon and indicated mutations of the flanking E452 (highlighted in red). The exon-skipped isoform (−EX) and exon-included isoform (+EX) are shown alongside mutant constructs. Cells expressing the constructs at different amounts are shown (upper/lower rows), with

the cells' construct expression level relative to the threshold used to define the transfected population is indicated in bottom of each image. Bottom bars indicate the cluster assignment of each construct based on image-derived feature analysis, including a −EX cluster, a +EX cluster, and the E452A/K subcluster. Scale bar: 25μm.

Consistent with this interpretation, the LUMIER-based analysis of SNX2 mutant interaction with the insulin receptor (Figure 5G in manuscript) also supports the view that the SNX2+EX E452K substitution does not reverse the endogenous isoform difference. In particular, the LUMIER results better track the granularity-associated behaviour of the constructs than the texture-related shifts captured by PC1, with the SNX2-EX R451E mutant's interaction with INSR being more comparable to SNX2+EX's than SNX2-EX's. We therefore interpret the E451A/K mutants not as evidence against our conclusion, but rather as indicating that residues near the microexon can perturb different aspects of SNX2 behaviour - captured by texture-related features - to those affected by the microexon or R451.

Collectively, our revised analysis supports a more nuanced model than we originally stated. We agree with the reviewer that our original wording was too strong: the statement that the microexon residue composition "does not play a role" is not fully supported by the data. Instead, our analysis indicates that residues within the microexon can influence some localisation-derived features, but that these effects are more modest than those associated with the immediately flanking residue context, especially R451, and predominantly affect other parts of SNX2's localisation behaviour. We have therefore revised the manuscript text accordingly to clarify that, within the mutagenesis series tested here, the principal localisation difference is more strongly associated with the flanking residue context, while residues within the microexon may fine-tune aspects of localisation.

In the initial mutagenesis section, we have changed the section header:

Insertion of the SNX2 microexon, not its composition, is a key determinant of isoform-related localisation and interaction changes

In addition, we significantly rewrote Results text as follows:

Here, we performed principal component analysis (PCA) of SNX2's isoform-distinguishing features after removing highly correlated features (see Methods). Using PC1 to PC5 scores, which together captured 98.9% of the variance (**Figure S13D**), we then grouped the constructs into two clusters. These corresponded closely to the endogenous SNX2-EX (-EX cluster) and SNX2+EX (+EX cluster) and were predominantly separated along PC2 (**Figure 4H-I**). This pattern was absent in the non-transfected controls (**Figure S13E-F)**, indicating that the clustering reflected construct-specific effects rather than batch effects. Based on this clustering, we considered that PC2 predominantly captured the differences between the endogenous isoforms. Consistent with this, the two clusters separated almost perfectly along the granularity feature vector (**Figure 4J**), which was the most important feature for SNX2

isoform classification (**Figure S11B**). Inspection of cells expressing constructs in either cluster further confirmed that this clustering captured the isoform-specific granularity phenotype, with aggregates forming in the -EX but not +EX cluster (**Figure 4K-L**).

Interestingly, clustering reflected the presence or absence of the four-residue microexon with all +EX mutants clustering with the +EX isoform regardless of amino acid substitutions within the exon (**Figure 4I**). This indicated that residue insertion, rather than the specific side chain composition, was the dominant determinant of the major isoform-specific localisation differences, particularly granularity differences. Indeed, even the +EX E452A/K mutants, which were the most distinct from the SNX2+EX isoform, showed similar granularity behaviour to +EX (**Figure 4K-L, Figure S13G**). The differences between these mutants (and other SNX2+EX mutants) and SNX2+EX instead seemed to relate to localisation features captured primarily by PC1, which was driven primarily by texture features and reflected behaviours distinct from both the endogenous +EX isoform and the –EX cluster.

Among the SNX2-EX mutants, we observed that progressive substitutions of R451 from positive or small to neutral to negative residues (R451K/A → R451Q → R451E) gradually shifted the SNX2-EX constructs from the –EX to the +EX cluster (**Figure 4I**) and produced a corresponding shift in granularity behaviour (**Figure 4K-L**), implicating positive charge at residue 451 as an importance determinant of the isoform differences.

Together, these results suggest that the SNX2 microexon functions as an α-helical insertion that extends local structure and alters isoform-related localisation differences by repositioning pre-existing, flanking charged residues. Within the mutational series tested here, this effect is influenced more strongly by the exon insertion than by its precise amino acid composition, although the microexon's residues may fine-tune certain aspects of localisation.

We have also added a clarification to the results section explaining why PC2 was biologically emphasized:

Using PC1 to PC5 scores, which together captured 98.9% of the variance (**Figure S13D**), we then grouped the constructs into two clusters. These corresponded closely to the endogenous SNX2-EX (-EX cluster) and SNX2+EX (+EX cluster) and were predominantly separated along PC2 (**Figure 4H-I**). This pattern was absent in the non-transfected controls (**Figure S13E-F)**, indicating that the clustering reflected construct-specific effects rather than batch effects. Based on this clustering, we considered that PC2 predominantly captured the differences between the endogenous isoforms.

We have also updated the text regarding the SNX2 LUMIER interaction experiment:

Crucially, this enhanced binding persisted in two +EX mutants, including one of the most distinct +EX mutants (+EX E452K), as well as in the –EX R451E mutant (**Figure 5G**), all of which clustered with SNX2+EX in the imaging experiment and did not form aggregates. The 4-residue insertion, rather than the precise amino-acid identity of the microexon, was therefore sufficient to alter interaction strength.

And revised the conclusion to provide a more nuanced mention of the SNX2 mutagenesis:

Consistent with the microexon tuning an existing interface, our mutagenesis of SNX2 shows that isoform-specific differences in localisation and partner specificity arise largely

independently of the exon's amino-acid composition, implying a structural rather than compositional mechanism.

And the abstract:

Consistent with this, biochemical analysis of a four–amino acid microexon in sorting nexin 2 - identified through our screen - confirmed that residue insertion, rather than side chain chemistry, was driving key differences in protein localisation through repositioning of a flanking, charged residue.

Along the text changes, we have updated the Figures related to the SNX2 mutagenesis. Specifically, we have added the correlation heatmap (Rebuttal Figure 4, included as Figure S13C), updated scree plot (Rebuttal Figure 6, included as Figure S13D), and new PC plots and biplots for transfected (Rebuttal Figures 5 and 7, included as Figure 4I-J) and non-transfected cells (Figure S13E-F) to the Main Figures and Supplementary Figures, as indicated. We also expanded the SNX2 constructs for which we show cell images (Figure 4K, Figure S13G) and updated the granularity line plot to show all SNX2 constructs (Figure 4L).

Lastly, we updated the Methods to reflect the updated PCA analysis:

Principal component analysis of SNX2 mutants

We analysed the SNX2 mutant constructs by principal component analysis (PCA) in R. Starting with the image-derived measurements of the SNX2 isoform-distinguishing features (~20) for all SNX2 construct-positive cells, we first reduced the number of input features by removing highly correlated features to improve interpretability and diminish the impact of a specific localisation behaviour caught by multiple features would have on the PCA. To do this, we calculated the pairwise Pearson correlation (r) between the features using the measurements from all SNX2 construct-positive cells (without grouping cells according to construct). The cells were pooled from three experimental repeats with n cells ≥168 expressing each construct (cell numbers for each construct listed in **Figure S13B**).

With the Pearson correlation values, we created a distance matrix (1-r), which we visualised as a correlation heatmap and used for hierarchical clustering of the features. This analysis revealed many features were highly correlated and we considered there to be 7 main feature groups. We therefore reduced features with a Pearson correlation of ≥0.7 to the single feature that showed the greatest variance across the cells, resulting in 7 retained features. We centred and scaled the measurements of the retained features and then performed PCA using prcomp() for all SNX2 construct-positive cells. With the PCA output, we created centroid representations for each construct by extracting the PC scores for each cell, grouping the cells according to SNX2 construct, and then calculating the mean PC score across the cells for each PC. We used the centroid PC scores from the first 5 PCs (capturing 98.9% of the variance) to cluster the constructs into two clusters via hierarchical clustering using Euclidean distance and Ward's D2 linkage. We then plotted the centroid PC scores on our principal component plots, showing PC1/2, as well as the assigned cluster for each construct. From the PCA, we also generated biplots and scree plots, both of which were calculated using the full cell dataset (not centroids). The plots were created using the using the factoextra[80] and tidyverse[81] packages.

We performed the analysis above using transfected cells and then performed the PCA separately for non-transfected cells using the retained features identified using the transfected cells, as a way of assessing construct-specific effects versus batch effects.

Reviewer 1 Comment 5B: In the 3D modeling, the authors consider only the monomer and focus on intra-molecular interactions between two helices. What is the rationale for doing so while the study emphasises inter-protein interfaces?
What are the interfaces in this protein?
What is the role of the micro-exons in these interfaces?

We thank the reviewer for this point. Our original structural modelling focused on the SNX2 monomer because this was the level at which we could most confidently assess the direct structural consequence of microexon inclusion. Specifically, the modelling indicated that inclusion of the microexon extends an α-helix in the BAR domain near the BAR-tip without substantially altering the overall fold, suggesting that its effect is more likely to involve modulation of an exposed surface than wholesale rearrangement of the BAR domain.

We agree, however, that the functional relevance of this event is likely to involve intermolecular interfaces. SNX2 functions within SNX-BAR dimers, notably the ESCPE-1 complex with SNX5 or SNX6, which can further oligomerise into higher-order membrane-associated coats. To place the microexon-associated structural change in the context of higher-order assembly, we examined the published cryo-ET structure of the related SNX1-containing ESCPE-1 coat (Lopez-Robles *et al.,* 2023 [PMID:37322239]). Although this does not provide residue-level information for SNX2 itself, SNX2 is a close homolog of SNX1 and participates in the same ESCPE-1 complex, making this a relevant structural framework for interpreting the SNX2 BAR-tip region.

Within this structure, the microexon-containing α-helix in the BAR domain and the BAR-tip lies in close proximity to neighbouring ESCPE-1 subunits during oligomerisation, placing it in a position where local structural changes could plausibly influence intermolecular contacts

Prior work has also suggested that perturbation of a neighbouring BAR-domain region can affect cargo binding, supporting the view that this part of the protein is functionally important. However, because that perturbation is not directly comparable to the microexon studied here and likely influences cargo binding indirectly, and because our own data do not define the relevant interface at residue level, we have limited our interpretation to the conclusion that the microexon may modulate interaction behaviour rather than assigning a specific mechanism.

Accordingly, we have revised the Discussion to present this point more cautiously rather than as a central mechanistic conclusion, and now state:

Specifically, SNX2's microexon extends a BAR domain α-helix near the BARtip and shifts the relative position of the surrounding residues, including R451. This may influence intermolecular contacts formed between SNX-BAR dimers, notably ESCPE-1, when they assemble into higher-order protein coats[74], which may relate to the altered interaction behaviour we observed between the isoforms in the LUMIER assay.

Reviewer 1 Comment 6: Could the authors comment on the fact that the fraction of interfaces altered by AS is very small? Is it because some data are lacking or is the observed fraction a realistic estimate?

We thank the reviewer for raising this important point. We agree that the observed fraction of annotated interfaces altered by AS is likely to be a conservative estimate rather than a true genome-wide proportion.

One reason is technical and relates to annotation completeness. AS is strongly enriched within intrinsically disordered regions (IDRs), whereas currently available interaction-interface resources are biased towards structured regions of proteins, including folded interaction domains and other structurally-resolved interfaces. As a result, AS-sensitive interfaces occurring within disordered regions are likely to be systematically underrepresented in the available annotation space. This is particularly relevant because many interactions mediated through IDRs involve short linear motifs (SLiMs), which remain incompletely annotated at the proteome scale.

A second reason is that our analysis was intentionally restricted to a stringent subset of regulated splicing events. Specifically, we defined tissue-regulated events using a minimum ΔPSI threshold of 20, such that the analysed exons represent comparatively strong shifts in exon usage rather than the full spectrum of alternative splicing. The fraction we report therefore reflects the proportion of currently annotated interfaces affected within this stringent set of tissue-regulated, frame-preserving events, rather than the prevalence of interface rewiring across all AS events.

To reduce this bias, we deliberately included resources that capture non-canonical and disordered-region interfaces, including DOMINO and ELM, in addition to structure-focused datasets such as Interactome3D and 3DID. However, despite this effort, we expect that many SLiM-mediated and other disorder-associated interfaces remain unannotated. We have therefore revised the text to clarify that the reported fraction should be interpreted as the fraction of currently annotated interfaces affected by AS and is likely to underestimate the true extent of AS-mediated interaction rewiring.

At the same time, we note that not all alternative splicing events are expected to affect interaction interfaces directly, so the low observed fraction likely reflects both

incomplete annotation and biological specificity. We therefore consider the current estimate to be informative within the limits of available interface annotations, but conservative with respect to the full landscape of AS-regulated interactions.

To reflect this, we have made the differential splicing cutoffs more explicit in the Results section:

Integrating differential splicing analysis[29,30] across 75 tissues and cell types, (delta percent spliced in (dPSI) > 20) we identified frame-preserving tissue-regulated AS events whose encoded protein regions overlapped at least one mapped interface residue or interval.

And:

In this framework, AS-regulated edges were progressively removed from the interface-resolved interactome in order of decreasing exon tissue-specificity, beginning with the most strongly tissue-regulated exons and ending with more tissue-wide events, defined here as exons detected across many tissues but still showing substantial tissue-to-tissue differences in inclusion.

And added a limitations paragraph to the Discussion that includes following statement:

First, the fraction of interfaces classified as AS-regulated is likely to be conservative, as our analysis was restricted to experimentally supported, currently annotated interaction interfaces, which are incomplete and biased toward structured, well-characterised interaction regions[76,78]. Since tissue-regulated AS is enriched in intrinsically disordered regions and short linear motifs[16], many AS-sensitive interfaces are likely to remain unannotated. In addition, our framework was intentionally limited to frame-preserving AS events that directly overlap annotated interfaces, providing a physically interpretable view of direct interface rewiring but excluding indirect effects mediated through allosteric or other distal structural changes, as well as frame-altering isoforms that may remodel interaction capacity or localisation through truncation or larger-scale architectural changes. Our conclusions should therefore be interpreted in the specific context of direct, interface-overlapping, frame-preserving AS events, which represent a tractable subset of the broader landscape of AS-mediated rewiring.

Reviewer 2 Comment 1: However, I found this manuscript very difficult to follow and in its current format it is not likely to be accessible or easy to understand by the broad readership of NSMB. At a bare minimum, it would benefit from revision to improve the clarity of presentation and the inclusion of a visual abstract summarizing the main findings would be very helpful for the reader.

We thank the reviewer for this important comment, which partially overlaps with Reviewer 1 Comment 2, and agree that the original presentation of the manuscript, particularly in the opening Results section, was too dense and insufficiently accessible for a broad readership. In response, we substantially revised the manuscript to improve clarity at both the conceptual and presentation levels. Specifically, we rewrote the opening Results section to explain the percolation framework step-by-step, clarified the biological ordering of edge removal from the most tissue-specific to the least tissue-specific exons, and replaced overly technical or inaccurate descriptions of

the network metrics with reader-friendly definitions that more closely reflect the implemented analysis. We also rewrote the section on identifying protein properties associated with the network results to improve flow and more clearly link the main ideas in the manuscript. We also updated the related Figures (Figure 1 and Figure S1) and legends to improve clarity. In addition, we have updated Figures 2, 4, and 5 and many supplementary figures to also improve clarity and flow.

In particular, we have:

- **Added a visual abstract (Rebuttal Figure 9) to provide a summary of the main findings**

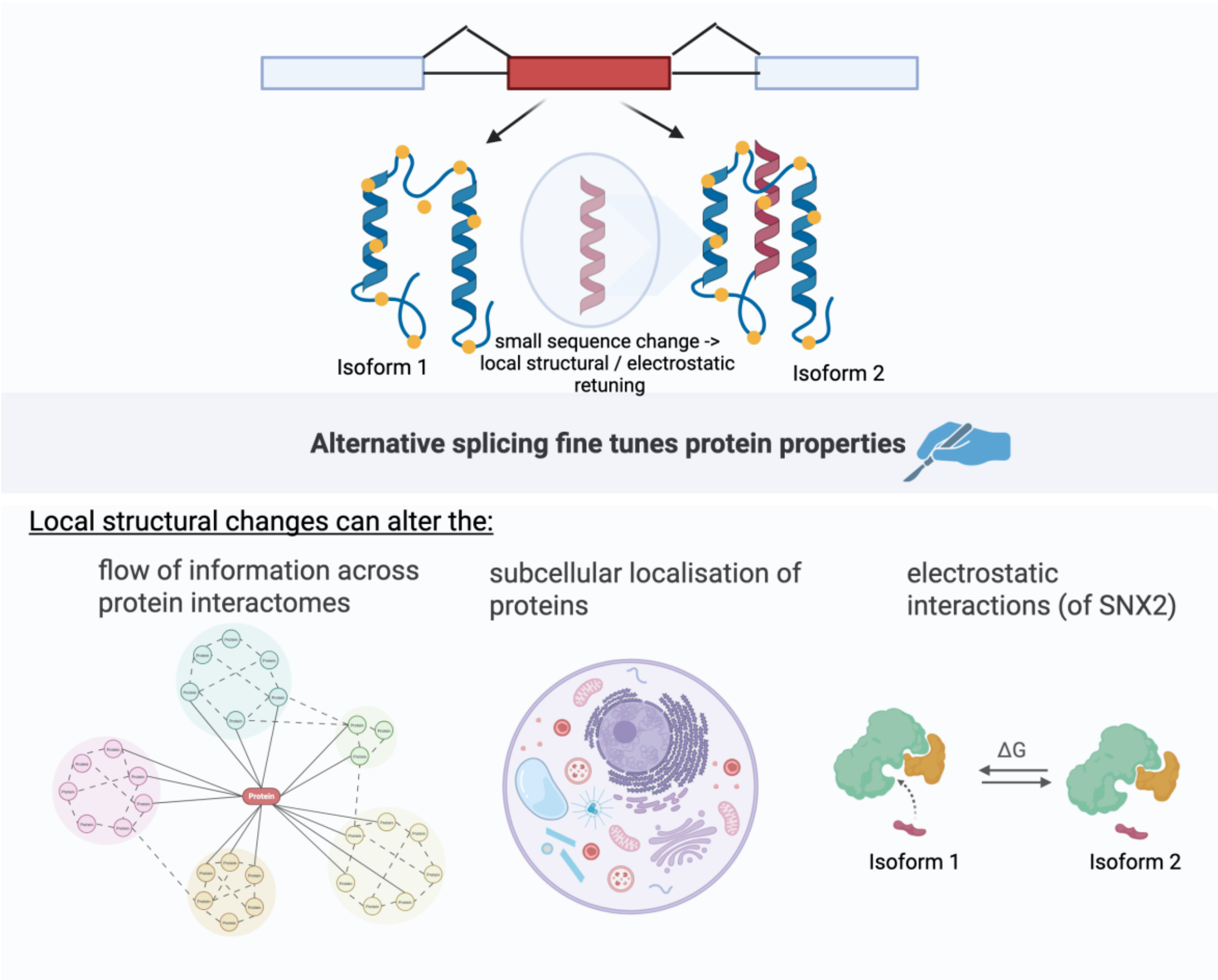

Rebuttal Figure 9 | Graphical Abstract.

- **Created an additional visual schema for Figure 1 to explain the network percolation analysis (Re-inserted below, Rebuttal Figure 3)**

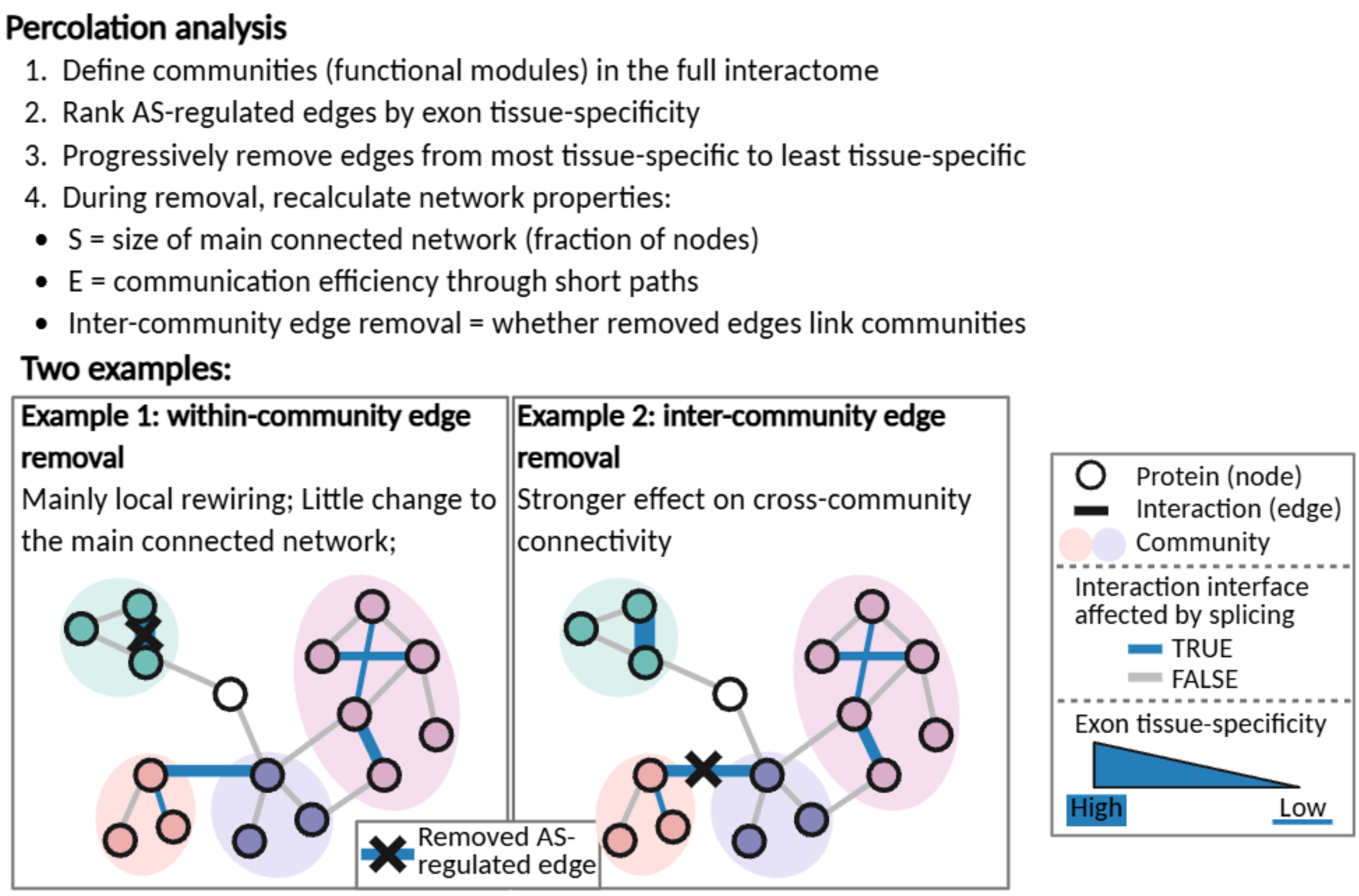

Rebuttal Figure 3 | Overview of the percolation analysis used to examine the interaction network and the impact of alternative splicing events overlapping interaction interfaces on network wiring. Communities refer to densely connected proteins and therefore often represent functional modules, such as proteins in the same pathway. AS-regulated edges were ranked by tissue-specificity and progressively removed, and network properties were recalculated at each step. Two examples illustrate removal of an intra-community edge versus an inter-community edge.

- **Rewritten opening Results section for clarity and flow as follows:**

Because our goal was to understand how transcript diversity translates into protein interaction rewiring across cellular contexts, we next investigated whether alternative splicing reshapes interaction topology at the systems level. To do this we examined how AS-regulated interfaces contribute to network organisation using percolation analysis and inter-community edge analysis (**Figure 1C**). In this framework, AS-regulated edges were progressively removed from the interface-resolved interactome in order of decreasing exon tissue-specificity, beginning with the most strongly tissue-regulated exons and ending with more tissue-wide events, defined here as exons detected across many tissues but still showing substantial tissue-to-tissue differences in inclusion. At each removal step, we quantified two complementary properties of the interactome network: we quantified S, the fraction of nodes remaining in the main connected part of the interactome, which captures whether the network stays largely intact rather than breaking into smaller disconnected pieces, and E, the approximate global efficiency, which captures how efficiently information can still travel across the remaining network. These observed trajectories were then compared with degree-matched random edge-removal null models to determine whether AS-regulated interfaces have non-random effects on network structure.

Removal of the most strongly tissue-specific AS edges caused a significant deviation from the degree-matched null expectation in the fraction of nodes retained in the main connected

part of the interactome network, but in a direction indicating less fragmentation than expected by chance (**Figure 1D**, ΔS: empirical two-sided $p < 0.001$). The sigmoidal shape of the ΔS trajectory suggests that the effect of progressive edge removal is not uniform across the ranked AS events: early removal of the most tissue-specific edges has relatively little effect on the main connected part of the interactome, whereas later removal produces a sharper transition once a more critical set of integrative edges is affected. Together with the absence of a significant effect on approximate global efficiency (**Figure S1E**, ΔE: $p > 0.05$), this pattern is most consistent with highly tissue-specific exons preferentially rewiring interactions locally rather than a broad disruption of network-wide communication. We next examined whether AS-regulated edges preferentially connect distinct functional communities, that is, groups of proteins that are more densely connected to one another than to the rest of the interactome. Consistent with network S, tissue-specific AS-regulated edges were depleted among edges linking distinct functional communities, whereas tissue-wide AS events significantly altered the fraction of edges linking distinct communities (**Figure S1F**, empirical two-sided $p < 0.002$). This pattern suggests tissue-wide events may contribute more strongly to maintaining connections between functional modules. Taken together, these findings are consistent with a model in which tissue-specific exons mainly fine-tune local network structure, whereas more tissue-wide exons have greater influence on inter-community integration.

Among tissue-specific events, microexons (< 30 nt) represent a distinct class of AS events in terms of effect on protein structure. Unlike longer exons, microexons frequently alter the structured domains of proteins[29], suggesting that they may exert different effects on interaction topology. We therefore tested whether microexons displayed a distinct pattern of network regulation. In contrast to the broader class of tissue-specific exons, which were associated primarily with local rewiring, microexons were significantly enriched among interfaces linking distinct functional communities (**Figure 1E; Figure S1G-H**, $p < 0.004$, empirical two-sided). This suggests that microexons preferentially regulate connections between pathways or complexes rather than acting only within local modules. Ordinary least-squares (OLS) modelling (see Methods) further showed that inter-community connectivity depended on both tissue regulation and local topology, with a significant regulation × microexon interaction term (**Figure S1I**, $p < 0.03$, OLS t-test). Together, these observations are consistent with microexons representing a specialised subset of tissue-specific exons with a disproportionate potential to occupy network positions that modulate crosstalk between different complexes or subcellular locations.

Tissue-specific splicing preferentially targets multi-domain proteins with broad interaction and localisation repertoires

We next asked whether the network effects associated with tissue-specific splicing were linked to specific protein properties. Regression and machine-learning analyses identified multi-domain architecture[31], interaction-substrate diversity, post-translational modification[32] density, and shared-neighbour connectivity as the strongest predictors of ΔS (**Figure 1F**, all $p < 10^{-5}$, Heteroscedasticity-robust OLS (HC3) Wald test), suggesting that tissue-specific AS may preferentially affect modular, multifunctional proteins. To determine whether these trends extended beyond the interface-overlapping subset, we then analysed all frame-preserving, tissue-specific AS events irrespective of direct interface overlap. Consistent with the interface-level results, tissue-specific events were enriched in multi-domain proteins (**Figure 1G**, $p < 3.47 \times 10^{-59}$, Kruskal–Wallis test) and in proteins with greater interaction-substrate diversity (**Figure 1H**, $p < 1.62 \times 10^{-16}$, Kruskal–Wallis test). Similarly, when proteins were grouped into broad categories based on cellular function, alternative splicing

propensity increased with the median number of unique domains per protein (**Figure 1I**, R = 0.85, $p = 0.0034$, Spearman's rank). Together, these analyses indicate that tissue-specific AS preferentially affects modular, multifunctional proteins that are likely to tolerate local changes in binding surfaces or domain arrangement without disrupting core activity.

A potential consequence of altered interaction behaviour is altered subcellular localisation[33]. We therefore next asked whether proteins regulated by tissue-specific AS also show greater spatial diversity within the cell. Integrating subcellular localisation annotations from the Human Protein Atlas[34], we found that proteins containing frame-preserving tissue-specific AS events were enriched among proteins localised to multiple cellular compartments (**Figure 1J**, $p < 0.0029$, Kruskal–Wallis test). Likewise, proteins with tissue-specific interface-regulating events showed higher localisation diversity (**Figure 1K**, all tissue-specific exons $p < 5.543 \times 10^{-17}$, Kruskal–Wallis across regulation bins). This association was strongest among highly modular proteins (**Figure 1L**, $\beta = 1.83$, $p = 9.5 \times 10^{-13}$, OLS t-test) and remained significant when proteins were analysed separately according to modularity (**Figure S1J**, high-modularity: $\beta = 0.58$, $p = 2.7 \times 10^{-15}$, OLS t-test). Microexon-containing proteins also showed elevated localisation diversity, although this association was attenuated at inter-community interfaces (**Figure 1M**, $\beta = 0.49$, $p < 0.001$, OLS t-test). Overall, these data support a link between tissue-specific AS and broader subcellular localisation diversity of proteins.

To move from this gene-level association to the level of individual splicing events, we next asked how often tissue-regulated exons might influence localisation by predicting localisation shifts between matched isoforms with and without the regulated exon using ProtGPS[35]. By this approach, 27.7% of tested exons (n = 7,979) were associated with a predicted redistribution across subcellular compartments (**Figure S1K**). Although this analysis is predictive rather than experimental, it suggested that exon-associated localisation changes may be sufficiently common to justify direct experimental testing, motivating the imaging-based screen described below.

- **Updated Figure 1 and Figure S1, including adding/updating plot axes and titles, and updating legends to help interpretability.**

- **Rewritten the Methods section to more specifically define metrics, background null distribution dataset and statistics (see Reviewer 1, Comment 2).**

- **More explicitly stated what we are comparing in the Results section on the protein isoform imaging and steps we take to improve comparability. For instance:**

Our aim was to determine whether isoforms from the same gene (referred to as isoform pairs) showed distinguishable subcellular localisation or behaviours when examined under comparable conditions (**Figure 2A**) based on quantitative features of the mCherry fluorescence signal, using identical and clearly distinct constructs as negative and positive controls.

And:

The isoform expression levels were generally comparable within isoform pairs (**Figure S2B**), and any residual imbalance was further reduced by excluding the brightest cells from the more highly expressed isoform (**Figure S2C)**.

- **Rewritten the section on using external data for assessing the isoform imaging results to clarify how these data were used and to improve the overall flow (See Reviewer 2, Comment 5). We updated Figure 2 and Figure S10 accordingly.**

Comparison of classifier-based localisation metric with independent interaction data suggests association between localisation and protein-protein interactions

To assess whether the localisation differences detected in our isoform library were supported by independent data, we examined whether differentially localising isoform pairs also showed interaction differences in an independent protein–protein interaction dataset. We reasoned that if isoforms differ in localisation, they may also differ in protein–protein interactions, because an altered subcellular context is expected to affect the pool of accessible interaction partners. While isoform-level data remain sparse, including isoform-related interaction data, we could examine the association for 10 isoform pairs by integrating data from LUMIER-based PPI screens[13,23,35] (**Table S5-6**). We observed that isoform pairs with greater localisation divergence also tended to exhibit greater interaction divergence (R^2 = 0.59, p = 0.027; **Figure S10**, Table S5). This cross-assay concordance is consistent with the idea that isoform-dependent localisation differences are accompanied by changes in interaction behaviour. Although limited in scope, this analysis therefore supports a broader association between localisation divergence and interaction divergence observed in the imaging screen and supports the measured localisation differences do occur for the select pairs present in both analyses.

- **Updated Figures 4 and 5, and supplementary Figures related to SNX2 to provide additional data to substantiate conclusions, and improve interpretability (see Reviewer 2, comments 7-12)**

Reviewer 2 Comment 2 + 3: I am not clear about what quality control for the overall workflow was used to validate reproducibility of the results. A major part of the experimental evidence presented makes use of a high content, fluorescence imaging approach using transient transfection of constructs to express and compare different fluorescent-tagged protein isoforms. How are these data controlled for variation in protein expression and/or modification levels between the respective protein isoforms? For example, it is well known that localisation of FP-tagged proteins will be distorted relative to endogenous counterparts if their expression level (copy number) is not matched in stoichiometry by copy numbers of endogenous interaction partner proteins.

We thank the reviewer for this important point. We apologise that our quality control procedures were not described clearly enough.

To assess reproducibility, we performed out-of-sample testing on the controls and a subset of the isoform pairs that were present in multiple experimental batches. The presence of the same pairs in multiple batches allowed us to train the classifier on one

batch and evaluate performance on an independent batch acquired weeks to months later. These analyses showed strong agreement between the results from the main screen and the out-of-sample testing (Figure 2H in manuscript) supporting the reproducibility of the imaging-based classification across independently acquired experiments.

We have revised the manuscript to describe the out-of-sample analysis more clearly:
To assess reproducibility, we performed out-of-sample testing on isoform pairs recurring in batches A and B. Out-of-sample accuracy was significantly correlated with classifier normalised ROC-AUC for both batches ($p = 0.01$ and 0.003; slopes ~0.36–0.41; **Figure 2H**), indicating reproducible discrimination of isoform pairs across independently acquired experiments. Moreover, this pattern is consistent with classifier performance reflecting construct-associated differences rather than batch- or well-specific effects.

We also agree that the transient expression approach doesn't inherently reproduce endogenous stoichiometry and that this is an important limitation for this type of fluorescence-based localisation assay. Our goal in these experiments was therefore not to reproduce endogenous stoichiometry exactly, but to compare matched isoform pairs under controlled conditions and test whether the alternative region confers reproducible differences in localisation behaviour.

To reduce bias arising from expression-level differences between isoforms, we took several quality control steps. First, for each isoform pair, we excluded very bright cells prior to analysis. Specifically, for each isoform we calculated an upper intensity cutoff as the 75th percentile of mean cellular intensity plus 1.5 × the interquartile range, and then applied the lower of the two cutoffs to both isoforms in the pair. This ensured that the compared populations were analysed over a matched upper intensity range.

To examine how this shaped the cell populations, for each isoform pair we have now calculated each cell's mCherry intensity relative to the population median mCherry intensity (cell mean intensity) of cells expressing either isoform. We did this before and after matching the upper intensity range and plotted the population distributions below (Rebuttal Figure 10-11, included as Figure S2B-S2C in manuscript). This showed that the isoform expression levels were generally comparable within pairs before filtering, but the that the filtering improved uniformity.

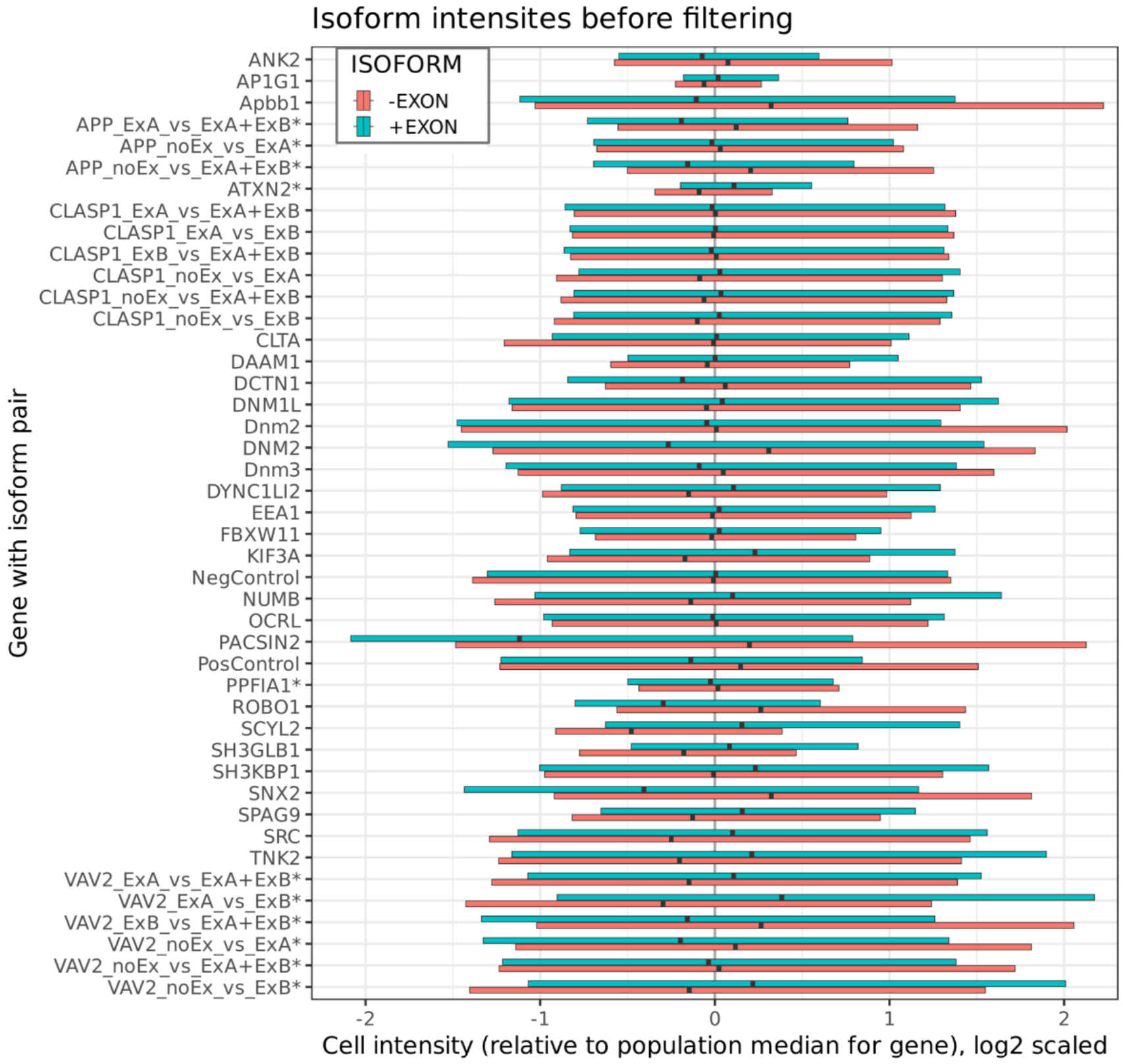

Rebuttal Figure 10 | Relative isoform expression levels within isoform pairs before filtering. Boxplots showing the cells' isoform expression (cell mean intensity) relative to the median population expression (mean cell intensity) of all cells expressing either isoform forming the given pair. The x-axis shows the relative expression level on a log2-scaled axis, and the y-axis shows the isoform pairs. Colour indicates isoform.

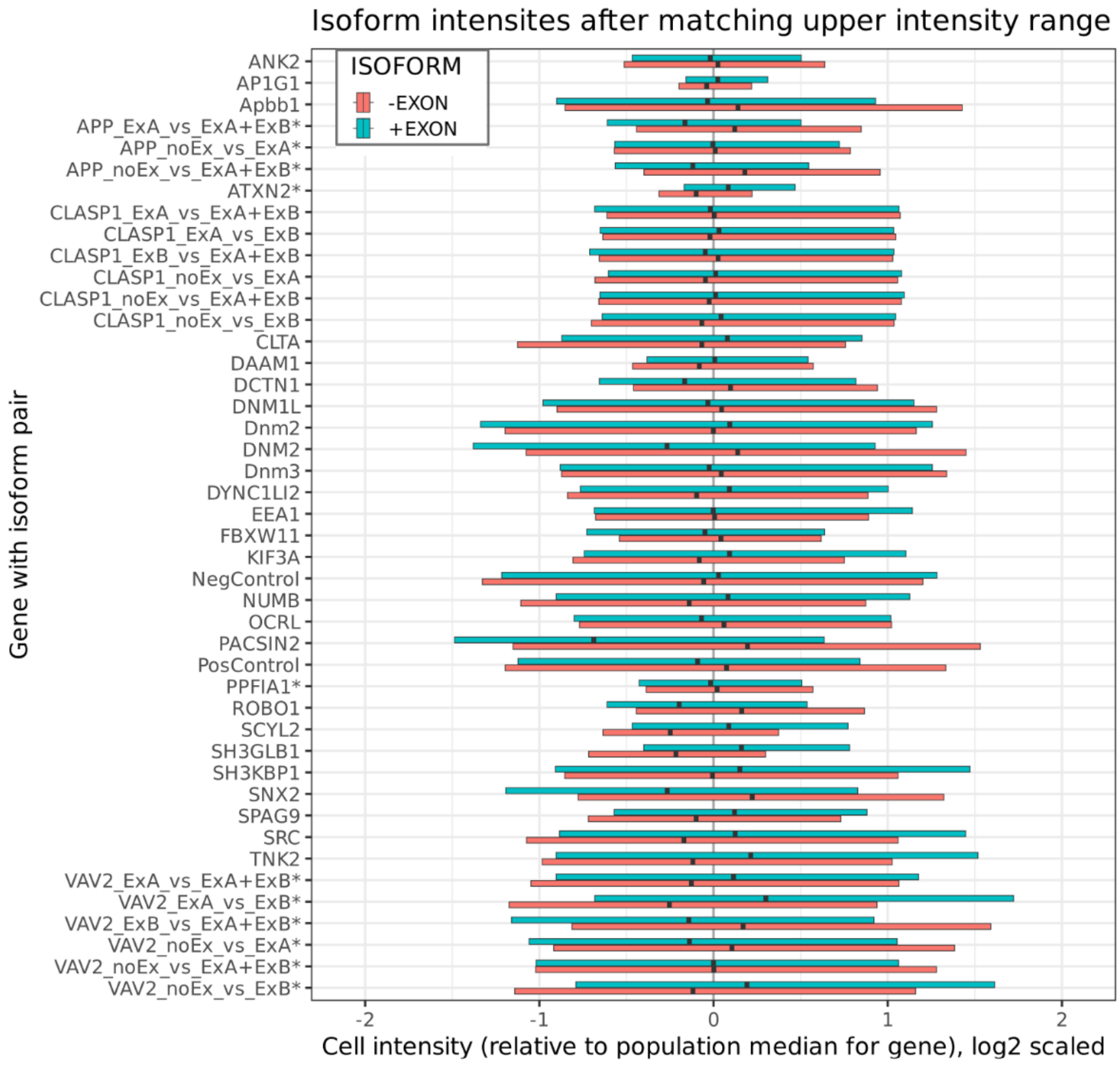

Rebuttal Figure 11 | Relative isoform expression levels within isoform pairs after matching upper intensity range. Boxplots showing the cells' isoform expression (cell mean intensity) relative to the median population expression (mean cell intensity) of all cells expressing either isoform forming the given pair, after the isoform pair had been filtered to have a matched upper intensity (Methods). The x-axis shows the relative expression level on a log2-scaled axis, and the y-axis shows the isoform pairs. Colour indicates isoform.

Second, after the classifier-based analysis of the library, we examined whether differential classifier performance correlated with expression-level differences between the compared isoforms and did not observe a general relationship (Figure S8A in manuscript). Third, we repeated the classifier analysis after stratifying cells into lower- and higher-expression bins and found that classifier performance remained highly consistent within isoform pairs across these ranges (Figure S9B in manuscript). Together, these analyses suggest that the detected localisation differences are not generally explained by simple differences in expression level between isoforms, nor are they restricted to a specific expression level within the measurable range of our experiment.

We have revised the Results and Methods to make these procedures more explicit. In particular, we now state clearly that expression levels were generally comparable within isoform pairs, that residual imbalance was further reduced by excluding the brightest cells using a matched upper-intensity threshold, and that isoforms were compared under comparable expression conditions. In addition, we have added the

two relative expression boxplots shown above to Figure S2 in the manuscript (as Figure S2B and S2C).

In the results section we have added following sentence:
The isoform expression levels were generally comparable within isoform pairs (**Figure S2B**), and any residual imbalance was further reduced by excluding the brightest cells from the more highly expressed isoform (**Figure S2C)**.

We have also added "when examined under comparable conditions" to the results section, to conclusions and discussion sections.

For example, in the Results, when introducing the imaging approach:

Our aim was to determine whether isoforms from the same gene (referred to as isoform pairs) showed distinguishable subcellular localisation or behaviours when examined under comparable conditions (**Figure 2A**) based on quantitative features of the mCherry fluorescence signal, using identical and clearly distinct constructs as negative and positive controls.

And in the concluding paragraph relating to the isoform library:

Together, our imaging-based examination of isoforms therefore support evidence from our *in silico* analysis that alternative splicing frequently alters subcellular localisation of multi-domain proteins and does so via both microexons and longer exons in the examined isoforms, when expressed at comparable levels.

And in the Discussion:

Whilst relatively small, our screen identified that nearly half of tested isoform pairs differ in subcellular distribution when examined at comparable expression levels

We have also added explicit mention of 'matched upper intensity range' to the Methods, when describing how the isoform pair datasets were generated:

We then applied the lower threshold for a given isoform to both samples in that pair, resulting in a matched upper intensity range.

For SNX2, we could directly test the question of over-expression and localisation by using HeLa SNX2 KO cells, made to stably express untagged SNX2-EX or SNX2+EX (referred to as rescue cells). The cells of the rescue populations expressed the SNX2 isoforms at varying levels with some expressing them at endogenous-like levels (see Reviewer 2 Comments 11 for specific aspects of the SNX2 isoform behaviour). In these cell lines, isoform-specific localisation differences were still detectable at endogenous-like expression levels and qualitatively consistent with those observed in the transient-expression assay, although the magnitude of the effect was smaller.

To see if these differences were also detectable using our classifier-based approach, we have applied our classifier-based workflow to these rescue cells. We found comparable normalised ROC-AUC values when analysis was restricted to endogenous-like expression levels versus the full expression range (0.5 versus 0.6) (Rebuttal Figure 12, included as Figure 5C in the manuscript). For SNX2, the rescue setting therefore

reproduced the same qualitative isoform-specific localisation difference observed in the over-expression system, while the classifier-based analysis also detected comparable separation when restricted to the endogenous-like expression range.

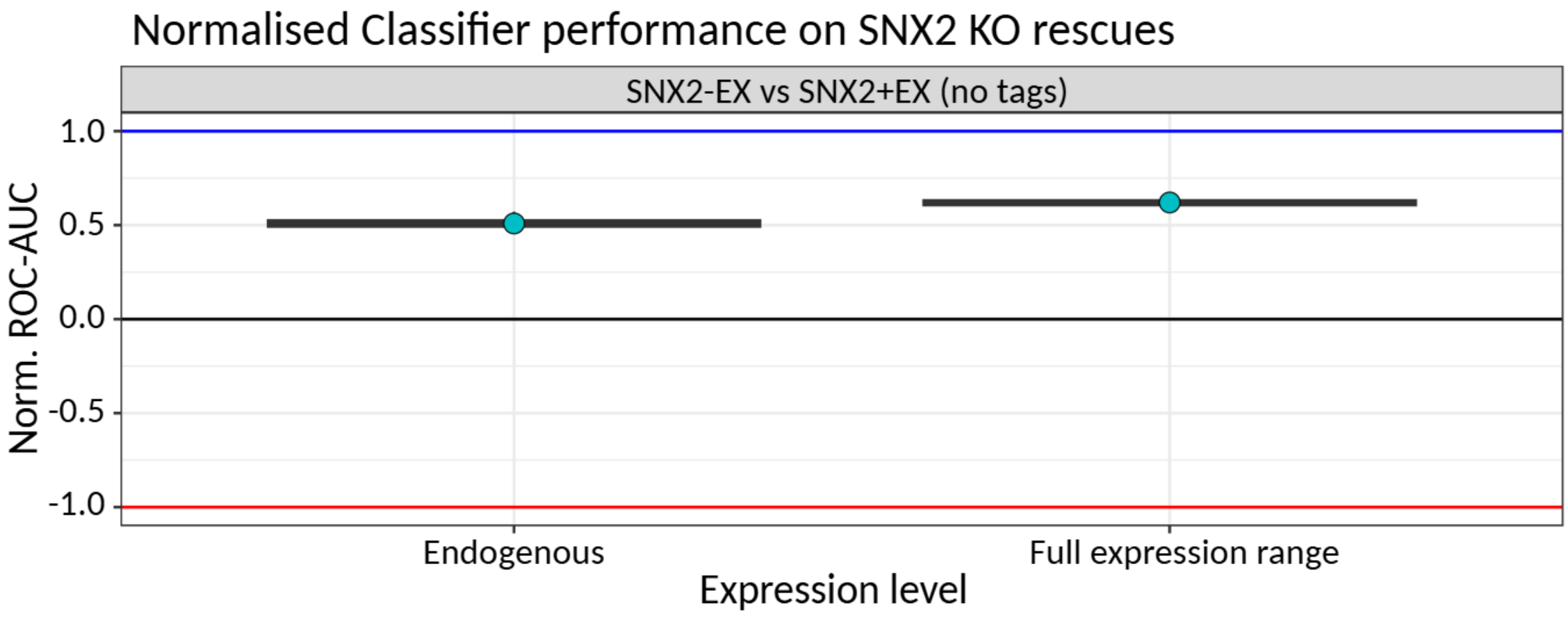

Rebuttal Figure 12 | Comparable classifier performance for SNX2 rescue cells at endogenous-like and full expression ranges. Boxplot showing the normalised ROC-AUC values from classifier-based discrimination of HeLa SNX2 rescue cells expressing untagged SNX2−EX and SNX2+EX based on the SNX2 signal. The analysis was performed using cells expressing the SNX2 isoforms at either endogenous-like levels (~0.7-1.5x the mean SNX2 intensity in HeLa wildtype, n cells = 418 per isoform) or the full measured expression range (~0.7-100x the mean SNX2 intensity in HeLa wildtype, n cells = 763 per isoform). The cell sampling and classifier evaluation was performed 50 times, and the boxplot shows the distribution of the normalised ROC-AUCs from these iterations. The dot indicates the median (n = 50).

We agree, however, that overexpression may modify some isoform-specific phenotypes, potentially including effects related to stoichiometry with endogenous interaction partners. We therefore interpret the imaging experiments as identifying isoform-specific differences that are detectable across a range of expression levels, rather than as a direct reconstruction of endogenous steady-state localisation.

We have now made this limitation explicit in the revised Discussion:

Second, the imaging-based localisation assays were performed primarily under transient over-expression of the mCherry-tagged isoforms rather than conditions that fully recapitulate endogenous stoichiometries. Although this enabled controlled comparison of matched isoform pairs, overexpression may modify phenotypes that depend on interaction-partner abundance, while tagging may also affect localisation. We addressed this by controlling for expression range in the analysis, comparing isoforms tagged in identical ways, and, for SNX2, observed a similar qualitative isoform-specific difference in an untagged rescue setting at endogenous-like expression, albeit with smaller magnitude. Thus, the assay likely captures intrinsic sequence-dependent localisation differences, while the strength of these effects may vary with expression context.

We have also included the classifier-based analysis of the untagged SNX2 isoforms in the SNX2 rescue cells (Rebuttal Figure 12) as part of Figure 5 and Figure S14 in the manuscript (see also Reviewer 2, Comment 12), and mentioned it in the Results as:

In agreement with this, our classifier-based approach confidently assigned correct isoform identities to the rescue cells at both endogenous-like levels (norm. ROC-AUC = ~0.5) and across the full expression-range of the rescue cell populations (norm. ROC-AUC = ~0.6) (**Figure 5C; Figure S14G**), supporting the differential localisation of untagged SNX2 isoforms.

And added a description of the classifier-based test of untagged SNX2 in the Methods, section:

Classifier-based analysis of untagged SNX2 isoforms in HeLa SNX2 KO rescues

We examined whether the untagged SNX2 isoforms were identified as differentially localising by our classifier-based approach by performing the classifier-based analysis as described in 'Classifier-based analysis of protein isoform pairs' on the SNX2 signal feature measurements from HeLa SNX2 rescue cells expressing either untagged SNX2-EX or SNX2+EX. We performed the analysis using cells expressing the SNX2 isoforms at two different levels: An endogenous-like level, consisting of cells positively expressing SNX2 in the images (See 'SNX2 cell lines') with a mean SNX2 intensity ≤1.5x that of the wildtype HeLa population mean SNX2 intensity, and a full-expression range, consisting of all cells positively expressing SNX2 in the images. This resulted in n cells per isoform of n= 418 for the endogenous-like level, and n=763 for the full-expression range, after down-sampling. As an internal normalisation control, we used the SNX2 negative cells present in the stable populations. These were defined as SNX negative cells with mean SNX2 intensities ≤0.27x that of the wildtype HeLa population mean SNX2 intensity. This threshold was chosen so that ≥99% of HeLa SNX2 KO cells stained with the αSNX2 antibody fell into the negative population.

As for post-translation modifications, we discuss that in Reviewer 2, Comment 4.

Reviewer 2 Comment 4: How do the authors know that the effects they are measuring reflect genuinely meaningful structural consequences resulting from AS, as opposed to differences affecting protein half-lives, copy number and/or post translational modification, all of which can in turn alter subcellular localisation?

We thank the Reviewer for this question. We agree that alternative splicing could influence localisation through multiple mechanisms, including direct effects on interaction interfaces, altered post-translational modification, altered protein stability or abundance, or more indirect regulatory effects. Our intention was not to claim that all localisation differences observed here arise from a single structural mechanism. Rather, our experimental framework asks whether AS events affecting regions linked to annotated interaction interfaces are associated with reproducible changes in localisation behaviour.

We apologise our overemphasis on 'structural' changes. In the network analysis part of the study, we use 'structural' in a broad sense to describe proteins as consisting of

modules that can mediate separate interactions, where 'structural changes' describes modular changes (module inclusion/exclusion/partial change), rather than using 'structural' to refer to stable tertiary structure. Accordingly, the affected regions may be structured or intrinsically disordered (See Reviewer 1, Comment 6) with both types being represented among the imaged isoforms (Table S2), and the relevant consequence of exon inclusion or exclusion may involve direct interface rewiring, altered accessibility of an interaction element, or altered regulation of that region.

We therefore agree with the reviewer that changes in PTM status could represent one route by which AS alters localisation. Indeed, if an AS event changes the presence of a PTM site, alters a nearby residue context, or modulates interaction with a modifying enzyme, and this in turn affects localisation, we would consider this a biologically meaningful consequence of the splice event. To examine this more systematically, we cross-referenced the imaged isoform library against PhosphoSitePlus and annotated whether the alternative region either contained a reported PTM site or lay within two residues of one. We observed a mixed pattern, with some alternative regions containing PTMs, others adjacent to PTMs, and others showing no nearby PTM annotations. Thus, PTM-dependent effects are plausible for a subset of cases, although many of the underlying PTM annotations are currently supported only by limited high-throughput evidence. We have now added PTM-status columns to the supplementary table describing the imaged isoform library, Table S2.

By contrast, our assay is less well suited to identifying localisation effects driven primarily by abundance or half-life differences. Because expression differences after transient transfection can arise both from biological effects and from technical variation in transfection efficiency, we deliberately filtered compared isoform populations to make their expression ranges more uniform (See Reviewer 2, Comments 2+3). This design reduces the risk that technical expression differences dominate the analysis but also means that modest localisation differences caused mainly by changes in abundance or half-life may be under-detected.

To assess whether large abundance differences were likely to be a widespread feature between the examined isoforms and might explain the observed phenotypes, we examined the distribution of isoform expression levels before filtering (Rebuttal Figure 10, same plot from Reviewer 2, Comment 2+3):

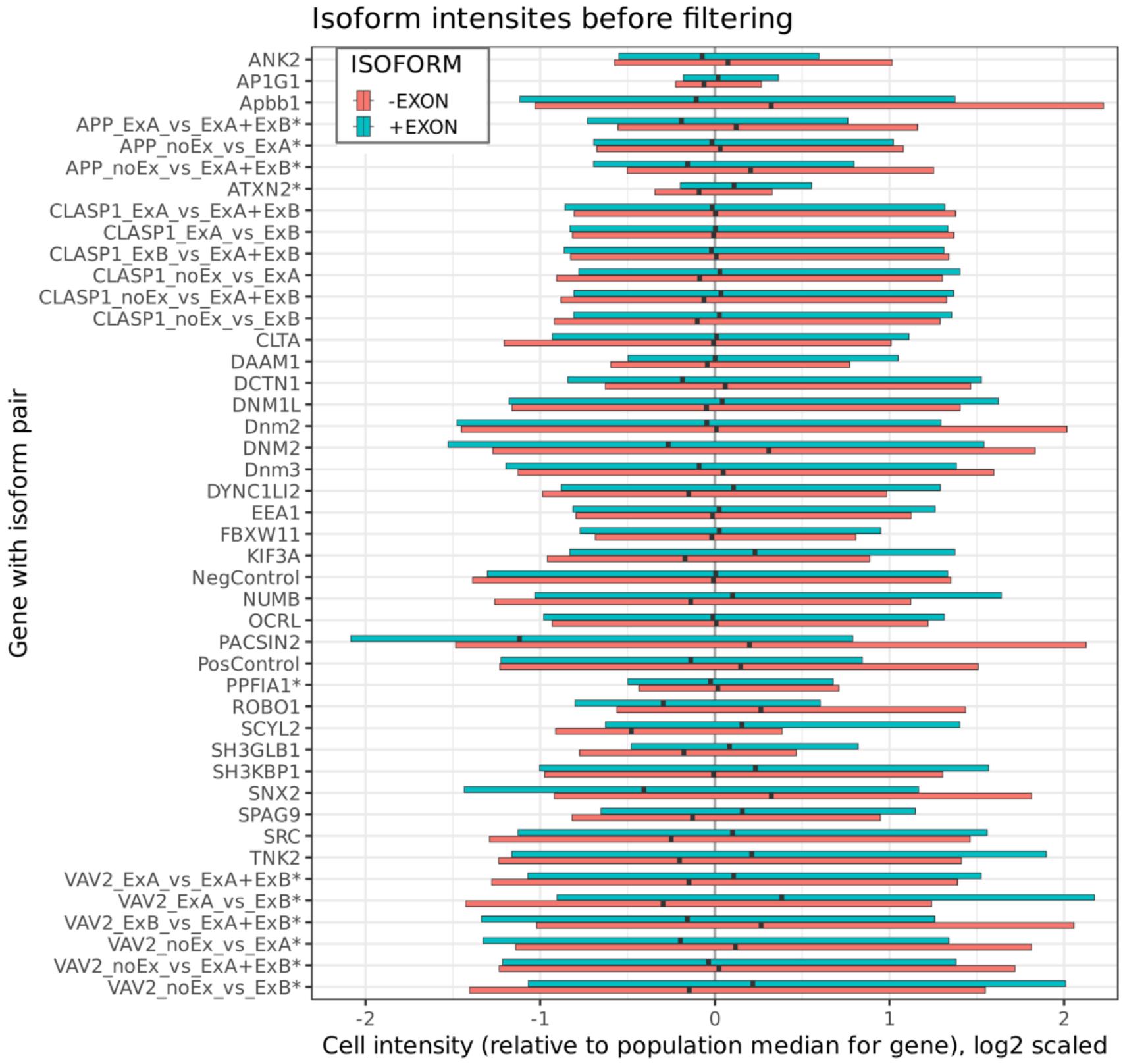

Rebuttal Fig. 10 | Relative isoform expression levels within isoform pairs before filtering. Boxplots showing the cells' isoform expression (cell mean intensity) relative to the median population expression (mean cell intensity) of all cells expressing either isoform forming the given pair. The x-axis shows the relative expression level on a log2-scaled axis, and the y-axis shows the isoform pairs. Colour indicates isoform.

Across the library, the two isoforms within a pair generally showed comparable intensity distributions, suggesting that large systematic abundance differences are not a dominant feature of the dataset. We acknowledge, however, that transient-expression intensity is only a crude proxy for protein abundance and cannot disentangle biological stability effects from technical variation in delivery and expression. We therefore view abundance-mediated effects as possible in individual cases, but not as the most likely general explanation for the observed localisation differences.

To make these points clearer, we have revised the manuscript to remove emphasis on "structural" consequences.

We have changed the last paragraph in the Introduction:

To address this, we combined interface-resolved ~~structural~~ network modelling with high content imaging of protein isoforms, machine-learning-based analysis,

And the start of the Results to the following:

To investigate how alternative splicing (AS) reshapes protein interactions at the level of mapped interaction interfaces, we constructed an interface-resolved human interactome by integrating curated interface annotations from UniProt[23], DOMINO[24], IntAct[25], 3DID[26,27] and ELM[28]. In this network, each node represents a protein, and each edge represents an

experimentally supported protein-protein interaction where the interface region was mapped onto UniProt canonical sequences (**Figure 1A**).

We have also included Rebuttal Figure 10 showing the relative isoform levels as part of Figure S2, and the PTM annotations in Table S2, which describes the imaged isoform library.

Moreover, we have expanded the Discussion to note that PTM-, abundance-, and half-life-related effects are all plausible mechanisms by which AS may alter localisation, even though our current assay is most sensitive to reproducible isoform differences within matched expression ranges:

More broadly, our assay does not by itself resolve the full mechanistic basis of the detected localisation differences. In addition to direct structural effects on targeting motifs or interaction interfaces, AS may also alter localisation indirectly through changes in post-translational modifications, protein abundance, stability or half-life[8,80]. The assay, however, is less suited for identifying localisation-affecting abundance effects as it is designed to compare isoforms expressed at similar levels.

We have also added a brief description on how PTMs were annotated to the Methods:

Annotation of the post-translation modification status of the alternative regions within imaged isoforms:

We annotated the post-translation modification status within and immediately around (within 2-residues on either side) the isoform-specific regions for the imaged isoforms by cross-referencing PhosphoSitePlus[108] (accessed March 2026). We considered a PTM detected in the region if it was identified in a single study. The annotations are included in **Table S2**.

Reviewer 2 Comment 5: While the authors state that an orthogonal validation of the localisation screen was performed, it is not clear that such validation was really performed in a comprehensive, unbiased way, as opposed to showing some cherry-picked examples showing positive results (NUMB and ANK2). How many examples are not consistent?

We thank the reviewer for this important point. We agree that our original wording may have overstated the extent of orthogonal validation and did not sufficiently distinguish between different forms of supporting evidence.

What we intended by "orthogonal validation" was the integration of independent LUMIER protein–protein interaction data, rather than a comprehensive second localisation-based assay across the imaged isoform library. These LUMIER data were incorporated for all isoform pairs in our dataset for which suitable interaction measurements were available. However, because isoform-resolved interaction data are sparse, this comparison could only be performed for a limited subset of the imaged isoforms (10 pairs). We therefore agree that this does not constitute a comprehensive,

unbiased validation of the localisation screen as a whole, and we have revised the manuscript to make this limitation clearer.

We have also removed the specific examples (ANK2, NUMB, and OCRL) from the section discussing orthogonal support for the screen, to avoid any implication that these illustrative cases constitute orthogonal validation. In the revised manuscript, these examples are instead mentioned in the Results section describing the imaged library, as representative isoform pairs containing either longer exons or microexons for which the classifier-based approach confidently identified localisation differences.

Regarding the general comparison with the previous literature, we now mention that we identify differences for 71% (5/7) of isoform pairs with previously described localisation differences, and that the localisation differences of those 5 pairs are comparable to those reported previously. This comparison is presented in the Results section describing the imaging library, rather than as evidence of comprehensive validation.

For the two isoform pairs where we don't recapitulate previous findings, SH3GLB1 [PMID: 24523556] and DNM2 [PMID: 18923138], we speculate the differences may relate to different cellular contexts of the experiments (neurons and embryonic stem cells, respectively, compared to HeLa cells) or homo-oligomerisation with endogenous isoforms which may attenuate isoform-specific effects. These discrepancies may therefore reflect differences in cell-type-specific interaction environments or other assay aspects, such as presence of endogenous isoforms.

We believe these changes: 1) a separate section on the LUMIER PPI data comparison, 2) reduced discussion of specific examples, and incorporation into the imaging results, clarifies what is intended as external validation and improves the overall flow and focus of the section.

New Results section on the use of external data for supporting screen observations:

Comparison of classifier-based localisation metric with independent interaction data suggests association between localisation and protein-protein interactions

To assess whether the localisation differences detected in our isoform library were supported by independent data, we examined whether differentially localising isoform pairs also showed interaction differences in an independent protein–protein interaction dataset. We reasoned that if isoforms differ in localisation, they may also differ in protein–protein interactions, because an altered subcellular context is expected to affect the pool of accessible interaction partners. While isoform-level data remain sparse, including isoform-related interaction data, we could examine the association for 10 isoform pairs by integrating data from LUMIER-based PPI screens[13,23,35] (**Table S5-6**). We observed that isoform pairs with greater localisation divergence also tended to exhibit greater interaction divergence ($R^2 = 0.59$, $p = 0.027$; **Figure S10**, Table S5). This cross-assay concordance is consistent with the

idea that isoform-dependent localisation differences are accompanied by changes in interaction behaviour. Although limited in scope, this analysis therefore supports a broader association between localisation divergence and interaction divergence observed in the imaging screen and supports the measured localisation differences do occur for the select pairs present in both analyses.

Changes to the Results on Library imaging:

Significant differences (bootstrap 99% CI > 0) were detected for the positive but not negative control, and in 38% (16/42) of pairs (**Figure 2F; Figure S7**), including 71% (5/7) of pairs with previously described localisation differences (**Table S3**) with these 5 pairs displaying localisation differences consistent with the previous reports[35–37,40–42]. We observed similar rates of localisation differences for microexons (~38%, 10/26) and longer exons (~38%, 6/16), with both classes represented among top performing classifiers - such as ANK2, NUMB, and OCRL (**Figure 2G**).

Reviewer 2 Comment 6: For the AP-MS used to identify the protein interactomes of the SNX2 isoforms, no details seem to be provided concerning either mass spectrometry analysis or data searching/analysis, so I cannot evaluate the validity of the AP-MS results shown. The authors should upload the raw and processed MS data to an open, online database archive, such as PRIDE, to allow other researchers to download and verify the data.

We thank the reviewer for highlighting this. This was an oversight on our part, which we apologise for. We have expanded the Methods section describing the AP-MS in terms of the sample processing, mass spectrometry acquisition, data processing, and interactome analysis to clarify the experiment. We have also deposited the mass-spec in PRIDE (PXD074758). This can be accessed via: https://www.ebi.ac.uk/pride/archive/projects/PXD074758. We have also included the protein search results from Mascot, and the interactome analysis from SAINT as Tables S7 and S8, to enable reuse and evaluation.

The sections added to the Methods are:

Downstream sample processing and mass spectrometry acquisition

The IP eluates were first reduced with 5mM DTT at +37°C for 30min, followed by alkylation with 10mM iodoacetamide at room temperature in the dark for 30min. Protein digestion was then performed using the SP3-based tryptic workflow[97]. Digested samples were desalted using two SDB-RPS disks (Empore, Sigma Cat#66886-U) packed into a 200µL pipette tip as previously described[99]. Extracted peptides from each clean-up were reconstituted in 10µL 0.1% (v/v) formic acid, 0.05% (v/v) heptafluorobutyric acid and 2% acetonitrile in water.

Digest peptides were separated by nanoLC using an Ultimate nanoRSLC UPLC and autosampler system (Dionex, Amsterdam, Netherlands). Samples (2.5µl) were concentrated and desalted onto a micro C18 precolumn (300 µm x 5 mm, Dionex) with H2O:CH3CN (98:2, 0.1 % TFA) at 15µl/min. After a 4 min wash the pre-column was switched (Valco 10 port UPLC valve, Valco, Houston, TX) into line with a fritless nano column (75µ x ~30 cm) containing C18AQ media (1.9µ, 120 Å Dr Maisch, Ammerbuch-Entringen Germany).

Peptides were eluted using a linear gradient of $H_2O:CH_3CN$ (98:2, 0.1 % formic acid) to $H_2O:CH_3CN$ (64:36, 0.1 % formic acid) at 200nl/min over 90 min. High voltage 2000V was applied to low volume Titanium union (Valco) and the tip positioned ~ 0.5 cm from the heated capillary (T=275°C) of a Orbitrap Fusion Lumos (Thermo Electron, Bremen, Germany) mass spectrometer. Positive ions were generated by electrospray and the Fusion Lumos operated in data-dependent acquisition mode (DDA).

A survey scan of m/z 350-1750 was acquired in the orbitrap (resolution = 120,000 at m/z 200, with an accumulation target value of 400,000 ions) and lockmass enabled (m/z 445.12003). Data-dependent tandem MS analysis was performed using a top-speed approach (cycle time of 2s). MS2 spectra were fragmented by HCD (NCE=30) activation mode and the ion-trap was selected as the mass analyzer. The intensity threshold for fragmentation was set to 25,000. A dynamic exclusion of 20 s was applied with a mass tolerance of 10ppm.

The "Downstream sample processing and mass spectrometry acquisition" and "Mass spectrometry data processing" steps were performed by the Bioanalytical Mass Spectrometry Facility at University of New South Wales.

Mass spectrometry data processing
The raw mass spectrometry data was processed using Mascot v2.8. Peak lists were generated using Mascot Daemon/Mascot Distiller (Matrix Science, London, England) using default parameters and submitted to the database search program Mascot (version 2.8, Matrix Science). Search parameters were: Precursor tolerance 4 ppm and product ion tolerances ±0.5 Da; Met (O) carboxyamidomethyl-Cys specified as variable modification, enzyme specificity was trypsin, 2 missed cleavage was possible and the Uniprot (February 2021) was searched. The mass-spectrometry data is deposited in PRIDE database (PXD074758).

Protein interactome analysis using SAINT
SAINTexpress[83] v3.6.3 was used to identify high-confidence protein interactors in the SNX2 positive lines (HeLa wildtype, HeLa Rescue SNX2+EX, HeLa Rescue SNX2-EX), while the HeLa SNX2 KO line was used to define the background. Raw spectral counts were extracted from the Mascot search results using the number of significant peptide matches per protein (prot_matches_sig, significance threshold $p \leq 0.05$), with a minimum requirement of two unique peptide sequences (prot_sequences) for a protein to be considered a hit in a given sample (**Table S7**). While three biological replicates were prepared for all cell lines, replicate 3 was excluded from the SAINT analysis for all SNX2 positive samples due to systematically lower total spectral counts compared to replicates 1 and 2. Note, all replicates were retained for HeLa SNX2 KO, which was used to model unspecific binding, and used for control subtraction prior to abundance scoring in the SNX2 positive lines. Candidate interactors were filtered using a Bayesian False Discovery Rate (BFDR) threshold of ≤0.01 as the primary cutoff, with a secondary threshold of ≤0.05. Protein abundance was reported as the average spectral count (AvgSpec) across replicates (**Table S8**), capped at 50 for visualisation purposes.

The included tables are referenced in the Results when the AP-MS is introduced:
We performed AP-MS of SNX2 in the HeLa SNX2 rescue lines stably expressing either isoform (untagged) (Table S7-8)

And the mass-spec data is also mentioned in the Data Availability section:

The mass spectrometry data has been deposited in PRIDE database with accession number: PXD074758

<u>Reviewer 2 Comment 7:</u> Figure S14 B clearly shows that the overall expression levels of the SNX2 -EX (granularity increased) is always higher than the expression of SNX2 +EX (lower levels of granularity) independently of the of the presence or absence of endogenous SNX2. Q: How did the authors achieve the same expression levels for both the SNX2 +EX and SMNX2 -EX in figure 4 A-B and S14 D and F?

We thank the reviewer for this comment. This was not sufficiently clear. We agree that the western blot in Figure S14B suggests the cells transduced to stably express SNX2-EX express relatively more protein than cells transduced to express SNX2+EX. However, this reflects the average behaviour of the bulk transduced populations rather than the single-cell expression ranges used for imaging-based comparisons.

The stable rescue cells were generated via lentiviral transduction followed by drug-based selection. The populations (Rescue SNX2-EX, Rescue SNX2+EX) are therefore heterogeneous cell populations with variable SNX2 expression levels as shown below (Rebuttal Figure 13). We have added this plot to Figure S14 (as Figure S14D in the manuscript) showing the distribution of the SNX2 levels at the single cell level across the wildtype, knockout, SNX2−EX rescue, and SNX2+EX rescue populations.

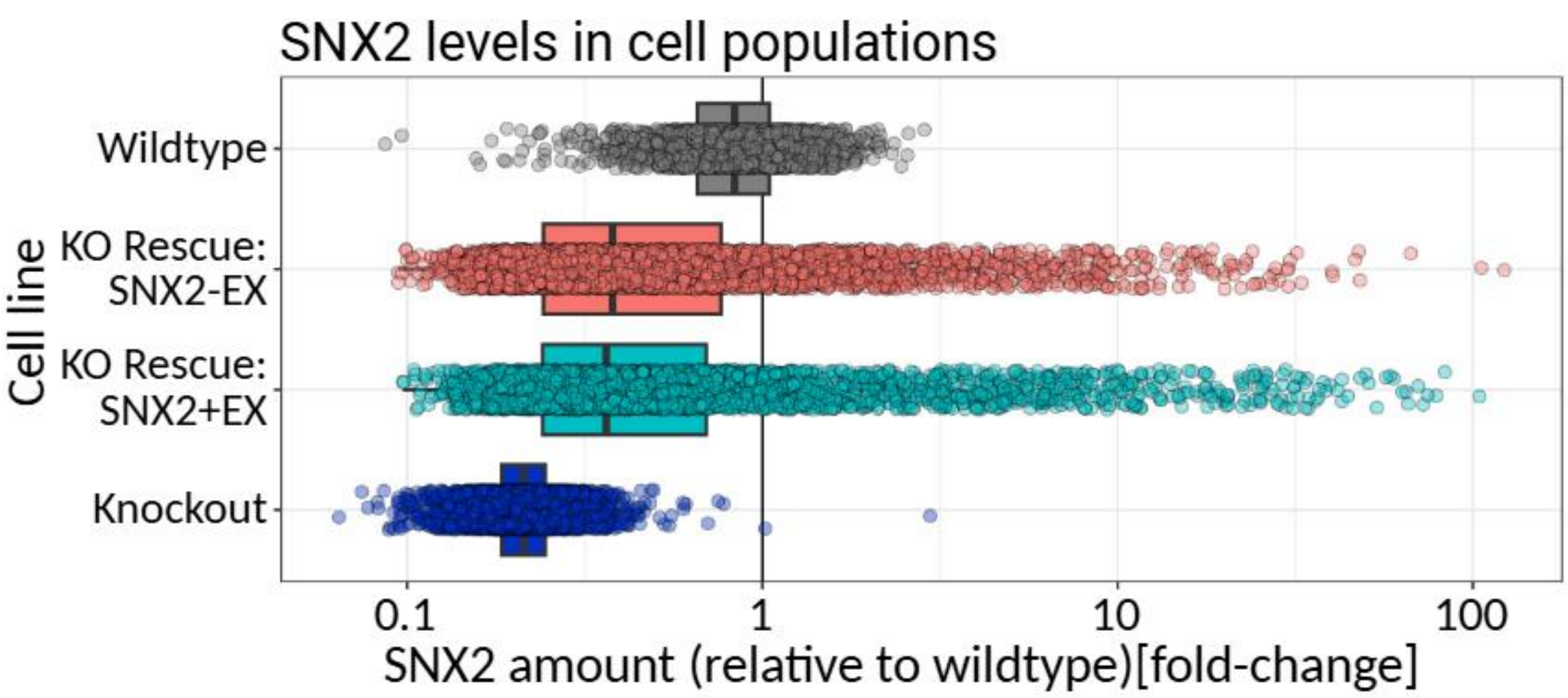

Rebuttal Figure 13 | SNX2 expression levels within different Hela populations (wildtype, KO, rescues). Boxplot of SNX2 expression levels (SNX2 intensity, relative to the mean SNX2 intensity in HeLa wildtype population [fold-change], measured via αSNX2 antibody) of all cells within the indicated HeLa lines. Each dot represents a single cell. Colour of dots and boxplots represents cell line. n cells wildtype = 2570, Rescue SNX2-EX = 4848, Rescue SNX2+EX = 4488, Knockout = 5579, pooled from N = 3 experimental repeats.

The plot shows that rescue cells stably expressing the two isoforms at comparable levels are present in both rescue populations. In the imaging analysis in the referenced

manuscript figures (Figure 5A-B and S14 D and F in the original manuscript, which are Figure 5A-B and Figure S14E and H in the updated manuscript), we utilise this overlap between SNX2 expression levels to selectively compare cells that express SNX2 within specific expression ranges. In Figure 5A, representative cells were deliberately chosen from the endogenous-like expression range (expression similar to HeLa wildtype). In Figure 5B, the analysis was restricted to cells with SNX2 levels comparable to endogenous HeLa cells (approximately 0.7× to 2.5× the wildtype mean). The lower threshold was defined based on unstained control wells to ensure that the measured signal reflected genuine SNX2 staining, and the upper threshold was set by the maximum intensity observed in the HeLa wildtype population. By contrast, Figure S14E and S14H show SNX2 isoform behaviour across a broader expression range, including overexpressing cells.

In summary, we therefore did not assume equal expression at the population level; instead, we utilise the overlap in single-cell expression distributions to compare the two isoforms at matched expression levels. We apologise this was not sufficiently clear in the original manuscript.

To amend this, we have re-written the Results introducing the stable cell lines to make this clear and included the Rebuttal Figure 13 as Figure S14D in the manuscript:
The rescue lines were heterogenous and expressed the SNX2 isoforms across varying expression levels (**Figure S14D**). By immunostaining the SNX2 KO rescue cells alongside wildtype HeLa cells with a pan-SNX2 antibody and imaging them using confocal microscopy, we could compare SNX2 isoform behaviour at varying expression levels by selectively examining cells expressing the isoforms within specific ranges.

We are using the same principle in all instances where we comparing cells expressing different isoforms/mutants. We have therefore added the following to all figure legends showing cell images to clarify this:
Representative cells were chosen to have comparable expression levels
And explicitly stated this in the 'Representative images' Methods section:
All cell images that are directly compared have the same contrast settings and were specifically chosen to show cells expressing the constructs at similar levels.

Reviewer 2 Comment 8 (+ 9): SNX2 +EX and SNX2 -EX are compared to endogenous SNX2 levels in HeLa cells (Figure S14D and F). Does endogenous incorporate all SNX2 isoforms expressed in HeLa cells? Is there anything known about the expression individual SNX2 splice isoforms in HeLa cells?

We thank the reviewer for this question. We apologise this was not sufficiently clear in the manuscript. In agreement with publicly available RNA-seq data, our HeLa cells endogenously express only the SNX2 isoform without the microexon (SNX2-EX). The

end-point RT-PCR panel (Rebuttal Figure 14, included as Figure S14C in the manuscript) shows the splicing status of our HeLa wildtype cells alongside the HeLa SNX2 KO clones and rescues, and we have now updated the Figure to state which isoform is expected in HeLa wildtype.

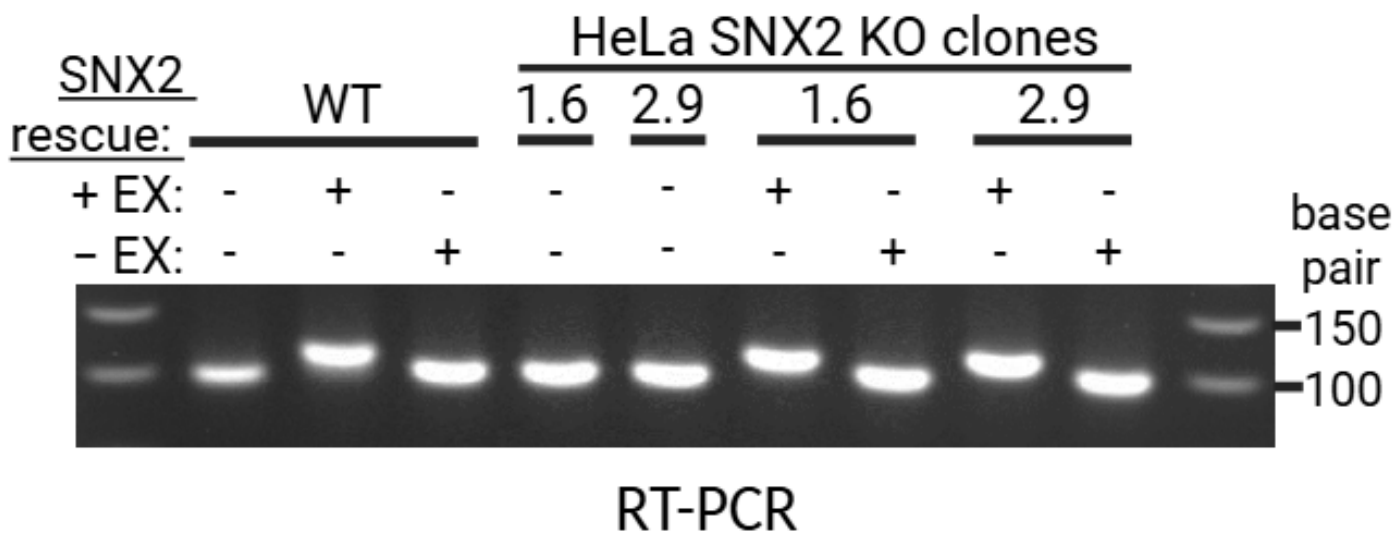

*WT only expresses SNX2-EX

Rebuttal Figure 14 | Updated end-point RT-PCR figure. Gel image of PCR products from end-point RT-PCR amplification using primers that bind the exons flanking the SNX2 microexon, using total RNA from HeLa wildtype or HeLa SNX2 lines as input. Expected PCR product sizes are 108nt (+EX) and 96nt (-EX) based on presence/absence of 12nt microexon. HeLa wildtype endogenously only express SNX2-EX. The presence of a PCR product in SNX2 KO cells may be due to trace amounts of SNX2 mRNAs, as KO was directed at a region of SNX2 upstream of the amplified region. Experiment was performed once.

The panel demonstrates that wildtype HeLa cells only express SNX2−EX under our experimental conditions, whereas the rescue lines express the expected introduced isoforms. Our figures showing SNX2 in HeLa wildtype (Figure 5A-B in the manuscript), endogenous SNX2 is therefore exclusively SNX2-EX. Also, our estimates of the relative abundance of SNX2 isoforms introduced in SNX2 KO refers to the level relative to the SNX2 amount in HeLa wildtype (and therefore SNX2-EX), although the used antibody recognises both isoforms.

We have amended the Results on the HeLa SNX2 cell lines to make this clear:

We first knocked out SNX2 in HeLa cells (**Figure S14A**), which endogenously only express SNX2-EX (**Figure S14C**), and then stably reintroduced untagged SNX2-EX or SNX2+EX by lentiviral transduction (rescue), as confirmed by western blotting (**Figure S14B**) and end-point RT-PCR (**Figure S14C**).

We have also updated Figures related to SNX2 in wildtype HeLa to state which isoform is used in HeLa (Figure 5A-B, Figure 5D, and Figure S14A-C in the manuscript).

Lastly, we have updated the Figure legend for the RT-PCR panel (Rebuttal Figure 14, included as Figure S14C in the manuscript) to indicate the expected PCR product sizes to improve interpretability.

<u>Reviewer 2 Comment 10:</u> Figure 5A: For a better understanding of where the SNX2 aggregates are located within the cell, it would be good to highlight the outline of the nucleus or show merged images of cell body/SNX2 staining cells.

We thank the reviewer for this suggestion and agree it may improve understanding. To make the subcellular position of SNX2 puncta clear, we have updated Figure 5A (Rebuttal Figure 15) to include DAPI to highlight the nucleus, as well as merged images to show the SNX2, and nuclear signals together. We have also included images of SNX2 KO cells transiently expressing mCherry-SNX2 +/- EX at endogenous-like levels. This allows direct visual comparison of untagged SNX2 isoforms and the mCherry-SNX2 isoforms at endogenous-like expression levels.

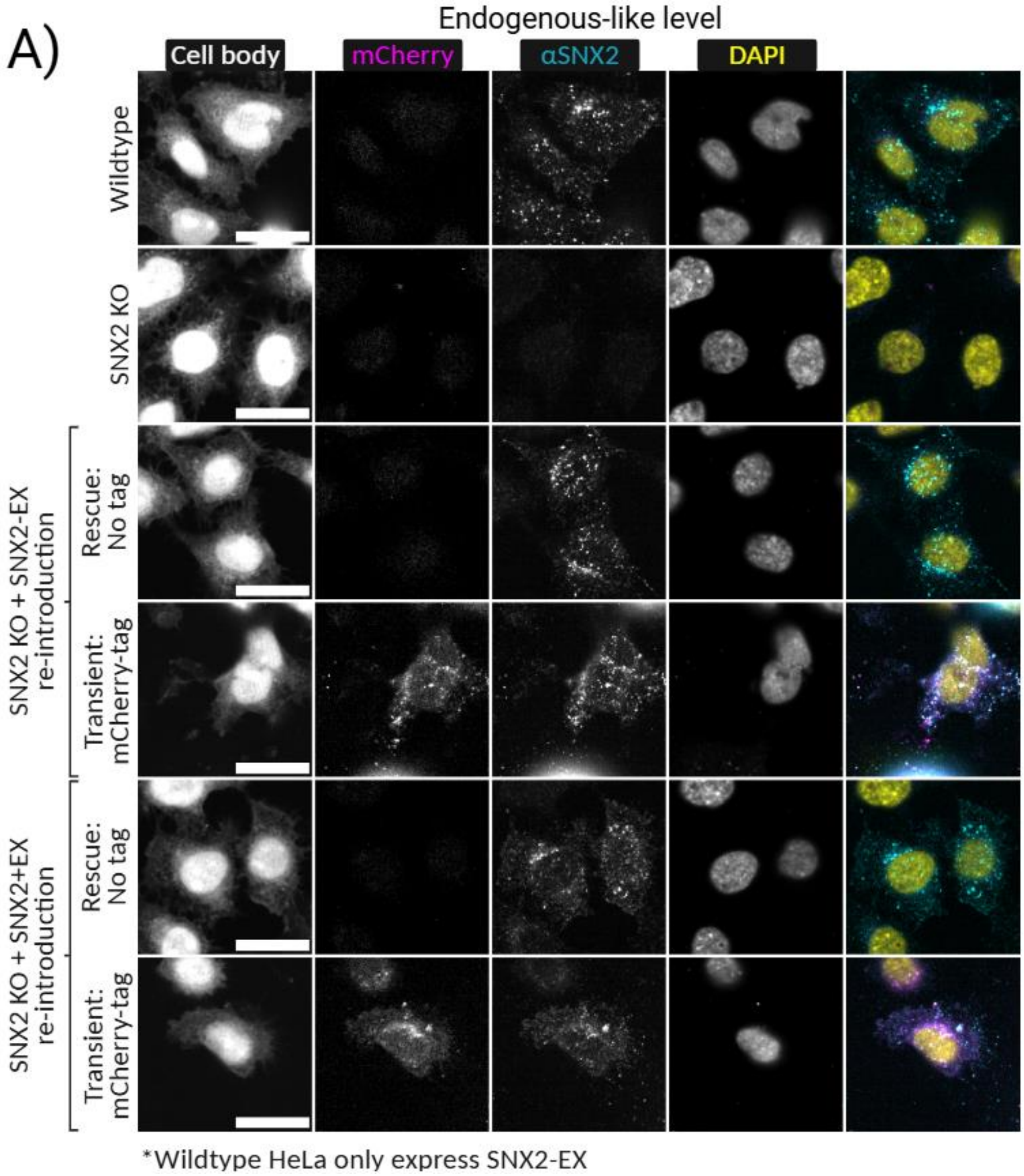

Rebuttal Figure 15 | Updated Figure 5A: Representative images of HeLa cell lines expressing SNX2 at endogenous-like levels. The cells are either HeLa wildtype, HeLa SNX2 KO, HeLa SNX2 KO stably expressing untagged SNX2+EX or SNX2-EX, or HeLa SNX2 KO transiently expressing mCherry-SNX2+EX or SNX2-EX. The cell body, mCherry, αSNX2, and DAPI channels are shown

independently and merged. Representative cells were chosen to have comparable expression levels based on the αSNX2 immunostaining signal. Scale bar: 25µm.

We have also updated the Results description to reflect the inclusion of the mCherry-tagged isoforms:

To evaluate the impact of the mCherry-tag on SNX2's behaviour, we next transfected the SNX2 KO cells with the mCherry-SNX2 isoforms. At endogenous-like and lower over-expression levels, we observed similar localisation behaviour between untagged and tagged isoforms (**Fig 5A; Figure S14H**), supporting the use of the tag in examining SNX2 localisation at lower expression levels.

And updated the legend of Figure 5A:

A) Representative images of HeLa cell lines expressing SNX2 at endogenous-like levels. The cells are either HeLa wildtype, HeLa SNX2 KO, HeLa SNX2 KO stably expressing untagged SNX2+EX or SNX2-EX, or HeLa SNX2 KO transiently expressing mCherry-SNX2+EX or SNX2-EX. The cell body, mCherry, αSNX2, and DAPI channels are shown independently and merged. Representative cells were chosen to have comparable expression levels based on the αSNX2 immunostaining signal. Scale bar: 25µm.

Reviewer 2 Comment 11: Figure 5A and Figure S14D: It looks like the mCherry tag is affecting the localization of SNX2 -Ex. Localization of SNX2+EX without mCherry tag in SNX2 KO cells (Figure 5A): SNX2 +EX shows a granular appearance mainly concentrated around the nucleus but also detected in the nucleus, albeit at lower levels when compared the SNX2 -EX. Localization of SNX2+Ex with mCherry tag in SNX2 KO cells Figure S14D and F: mCherry SNX2+Ex is very much evenly distributed throughout the cytoplasm excluding the nucleus.

We thank the reviewer for this comment. The potential of the mCherry tag affecting SNX2 localisation is an important consideration, and we have therefore explored this in more detail. To do this, we separated the Reviewer's concerns into two parts: first, whether the overlap of SNX2 signal with the DAPI signal reflects true nuclear localisation; and second, whether the mCherry tag alters the granular phenotype at different expression levels.

A. Nuclear localisation:

After examining the images, we found that the apparent differences in nuclear localisation are related to how the images are acquired and displayed rather than the constructs themselves. Indeed, we didn't find any of the SNX2 constructs (+/- mCherry-tag) localise to the nucleus.

Throughout the manuscript we acquired confocal stacks for each imaged field-of-view but only used a single slice from the stacks for analysis and display. This slice, which we deem the 'focus slice', was automatically selected during the image processing based on the % intensity variance of the cell body stain captured by the slice

(described in more detail in the Methods). This helped consistency between field-of-views and samples and was more robust than always selecting the same slice across stacks. The used parameters for selecting the 'focus slice' were established to capture the cell close to the adhesion site (glass surface). This increases the amount of cell area we could image and may contain the main regions of interest as we are examining cytoskeleton- and vesicle-associated genes. This also meant, however, we often captured a section of the cell that contained the cytosol located below the nucleus as well as a bit of the nucleus. The SNX2 signal that overlapped the DAPI/nuclear area could therefore be coming from the perinuclear region below the nucleus.

To examine this possibility, we inspected the slices around the focus slice (by 1µm). When moving away from the adhesion site (+1), we didn't observe SNX2 overlapping the nuclear signal when expressed at endogenous-like levels (Rebuttal Figure 16) or over-expressed (Rebuttal Figure 17) irrespective of whether SNX2 was tagged or not, suggesting SNX2 isn't nuclear but rather perinuclear with the signal coming from below the nucleus.

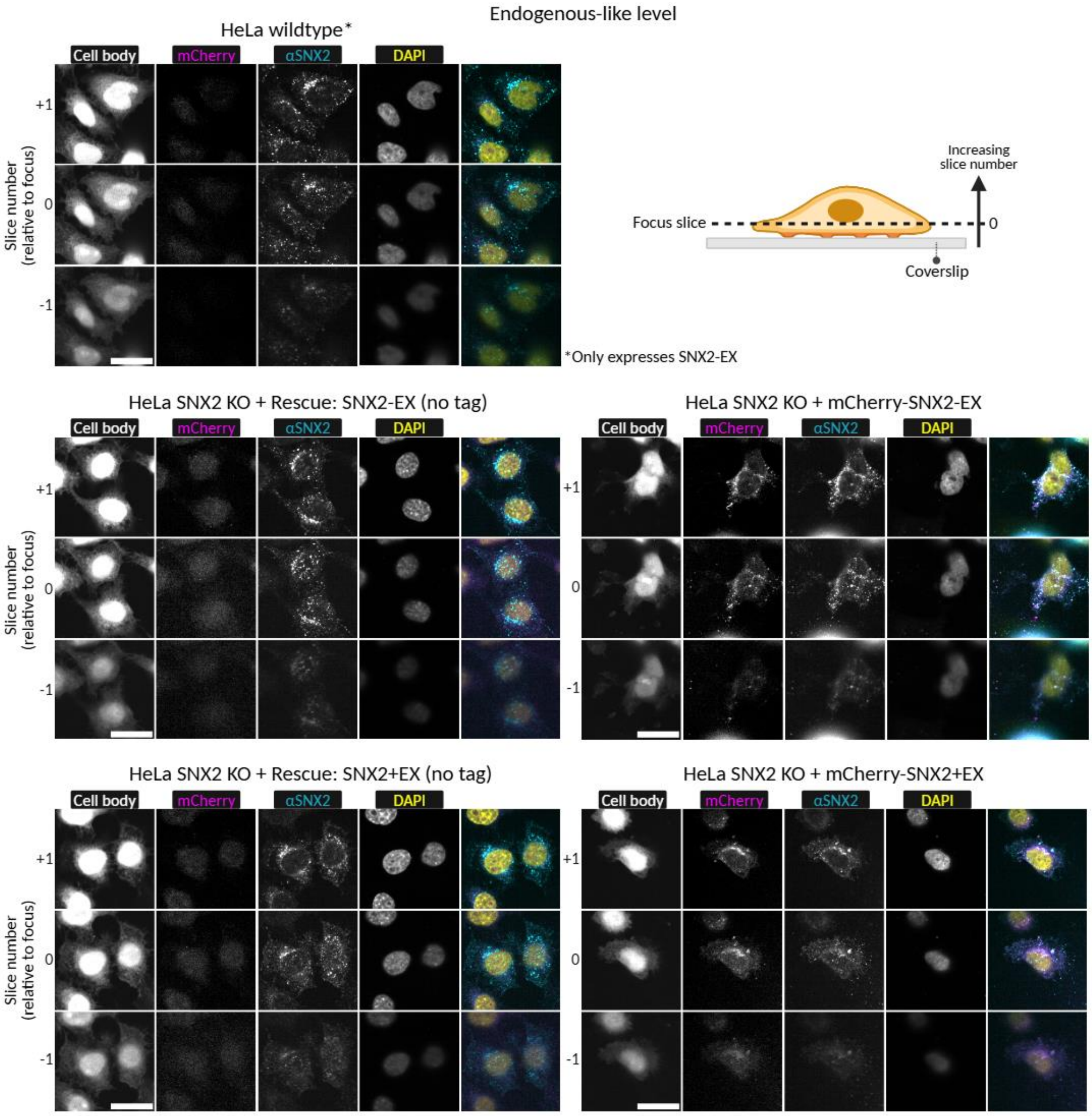

Rebuttal Figure 16 | Slice-by-slice of representative images of HeLa cell lines expressing untagged and mCherry-SNX2 isoforms at endogenous-like levels. The cells are either HeLa wildtype, HeLa SNX2 KO, HeLa SNX2 KO stably expressing untagged SNX2+EX or SNX2-EX, or HeLa SNX2 KO transiently expressing mCherry-SNX2+EX or SNX2-EX. The cell body, mCherry, αSNX2, and DAPI channels are shown independently and merged. Representative cells were chosen to have comparable expression levels based on the αSNX2 immunostaining signal. The same FOV is shown across three consecutive slices (-1, 0, +1) from the confocal stack, with 0 denoting the 'focus slice'. The association between slice and what region of the cell is imaged is indicated by the schematic top right. Scale bar: 25µm.

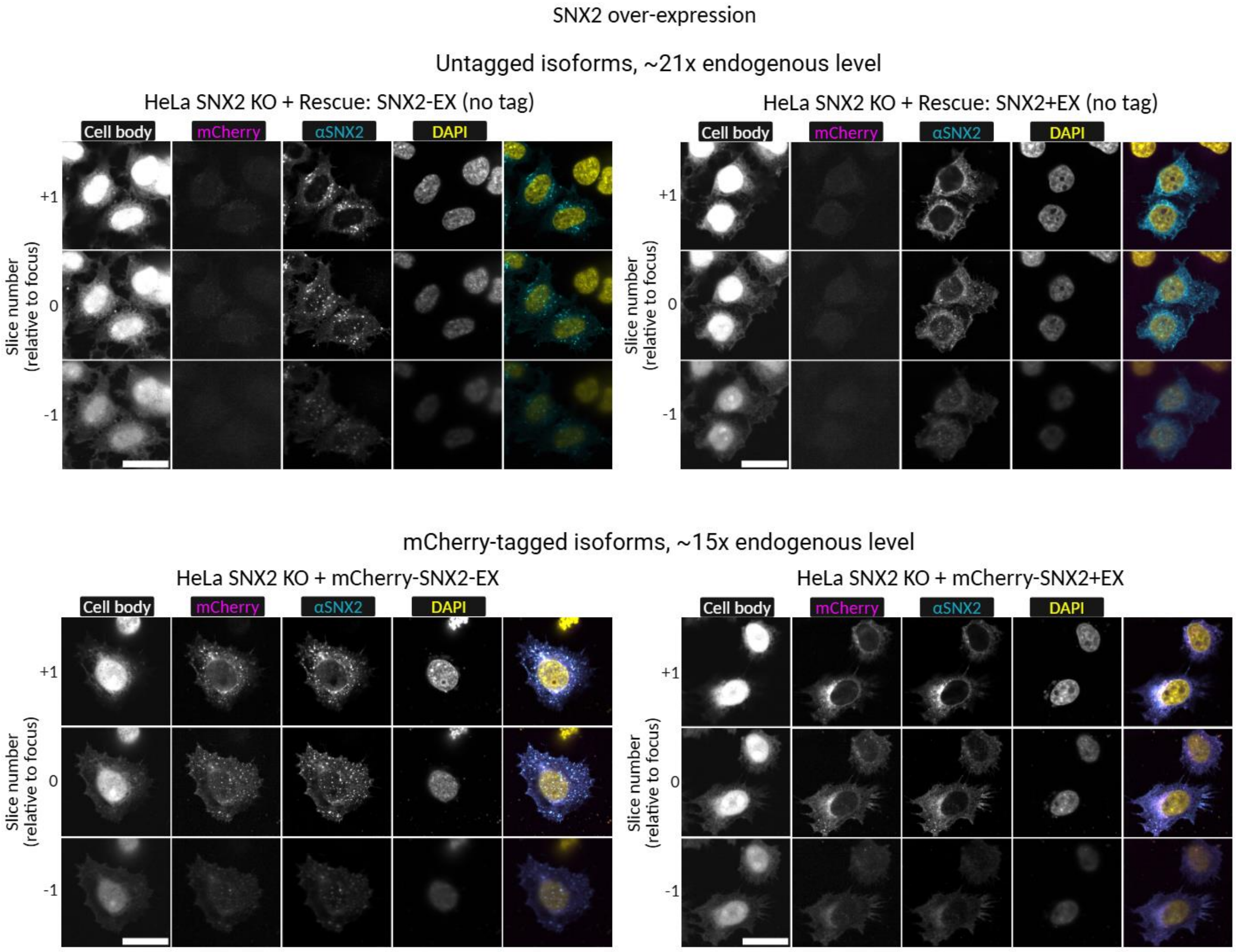

Rebuttal Figure 17 | Slice-by-slice of representative images of HeLa cell lines over-expressing untagged and mCherry-SNX2 isoforms. The cells are either HeLa SNX2 KO stably expressing untagged SNX2+EX or SNX2-EX, or HeLa SNX2 KO transiently expressing mCherry-SNX2+EX or SNX2-EX. The cell body, mCherry, αSNX2, and DAPI channels are shown independently and merged. Representative cells were chosen to have comparable expression levels based on the αSNX2 immunostaining signal. The same FOV is shown across three consecutive slices (-1, 0, +1) from the confocal stack, with 0 denoting the ‘focus slice’. Scale bar: 25μm.

This analysis indicates that the SNX2 isoforms are not detectably nuclear, irrespective of whether they are tagged or untagged within the measured expression range. The apparent overlap with the DAPI-positive region in the representative images instead reflects perinuclear SNX2 signal captured, rather than true nuclear localisation. Notably, the co-occurrence of SNX2 and DAPI also occured if we performed intensity max-Z projection rather than single slice analysis (Rebuttal Figure 18). We therefore found it difficult to avoid the apparent co-occurrence with the used imaging system and modality.

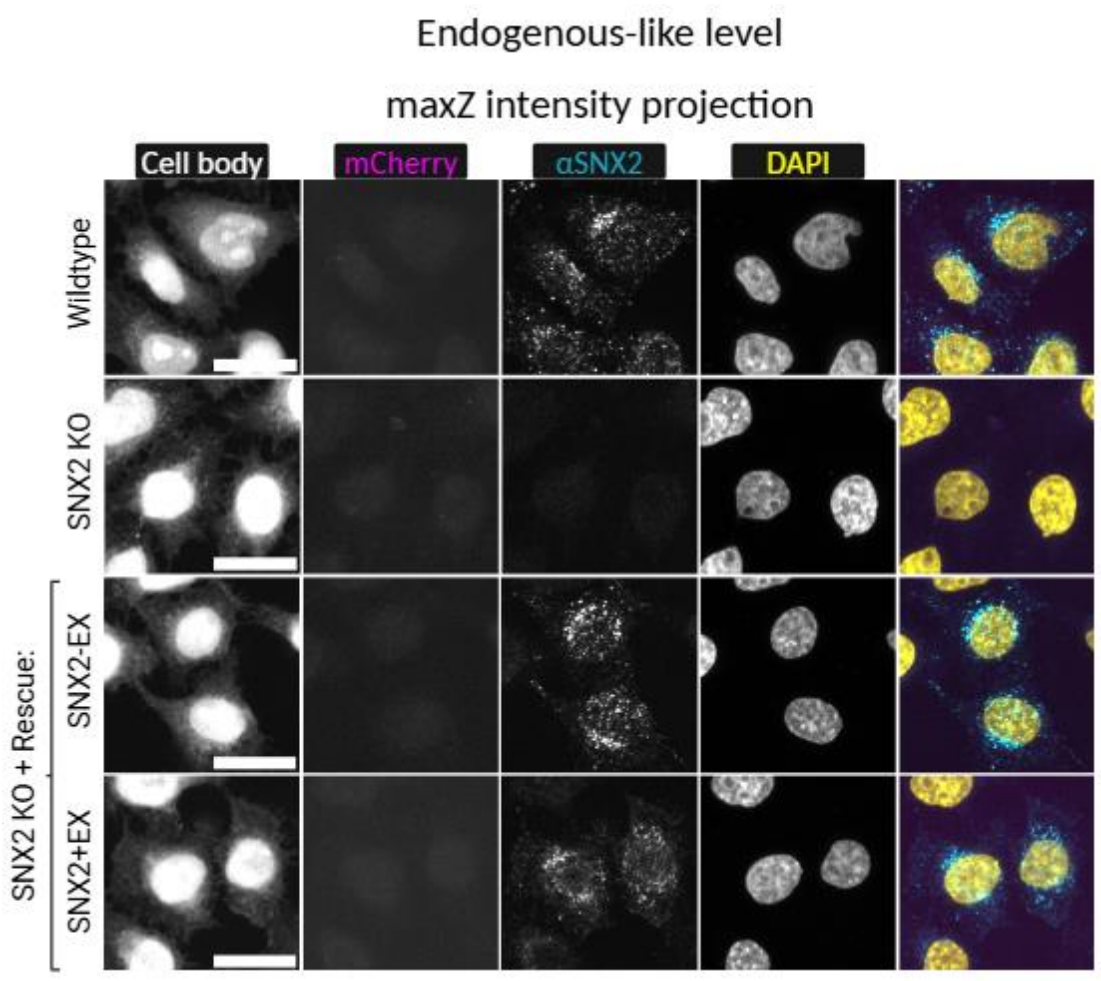

Rebuttal Figure 18 | Intensity Max-Z projections of representative images of HeLa cell lines expressing untagged SNX2 isoforms at endogenous-like levels. The cells are either HeLa wildtype, HeLa SNX2 KO, HeLa SNX2 KO stably expressing untagged SNX2+EX or SNX2-EX. The cell body, mCherry, αSNX2, and DAPI channels are shown independently and merged. Representative cells were chosen to have comparable expression levels based on the αSNX2 immunostaining signal. The FOV is intensity maxZ projected. Scale bar: 25µm.

To remove the ambiguity of whether SNX2 localises to the nucleus, we have included the consecutive z-slice images (Rebuttal Figures 16-17) as Figure S15 in the manuscript and clarified this point in the manuscript text. We apologise for this not being clear:

Although some SNX2 signal appeared to overlap with DAPI, inspection of consecutive confocal z-slices indicated that this reflected signal beneath the nucleus rather than nuclear localisation (**Figure S15**), consistent with the reported cytosolic localisation of SNX2[48].

B. Granular appearance:

We will address the granular behaviour at various SNX2 expression levels. Part of the discrepancy highlighted by the reviewer stems from the specific conditions (tag/untagged) not being labelled clearly enough in the original figure, which we apologize for.

Endogenous-like level and lower degrees of over-expression:

In the revised Figure 5A (Rebuttal Figure 15, same as Reviewer 2, Comment 10), we have now included both rescue cells expressing untagged SNX2 isoforms and SNX2 KO cells transiently expressing mCherry-SNX2 isoforms at endogenous-like levels. This allows qualitative comparison of the tagged and untagged isoforms. Under these conditions, SNX2−EX remains more punctate/perinuclear, whereas SNX2+EX is more diffuse, and this qualitative difference is similar for tagged and untagged constructs. The very few cells expressing the mCherry-SNX2 isoforms within this range, however, prevented us from quantitatively comparing the isoforms.

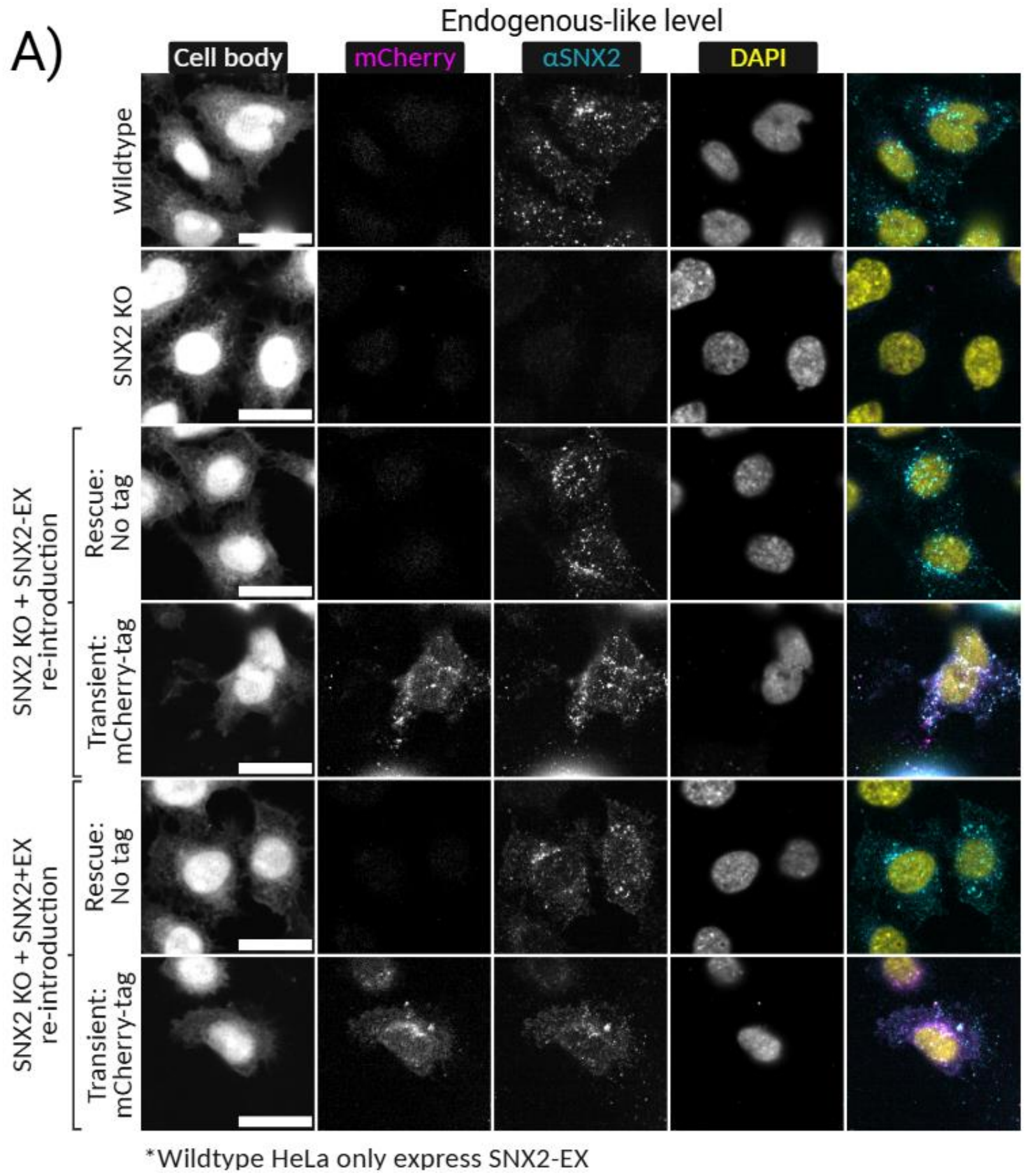

Rebuttal Figure 15 | Updated Figure 5A: Representative images of HeLa cell lines expressing SNX2 at endogenous-like levels. The cells are either HeLa wildtype, HeLa SNX2 KO, HeLa SNX2 KO stably expressing untagged SNX2+EX or SNX2-EX, or HeLa SNX2 KO transiently expressing mCherry-SNX2+EX or SNX2-EX. The cell body, mCherry, αSNX2, and DAPI channels are shown independently and merged. Representative cells were chosen to have comparable expression levels based on the αSNX2 immunostaining signal. Scale bar: 25μm.

We similarly observe comparable qualitative behaviour for untagged and mCherry-tagged SNX2 isoforms at lower degrees of over-expression (Rebuttal Figures 19-20, which correspond to Figure S14E and H in the updated manuscript or Figure S14D and F in the original manuscript). We have updated Figure S14 in the manuscript to explicitly state whether SNX2 is untagged or tagged in the images and included DAPI in the image panels to improve presentation.

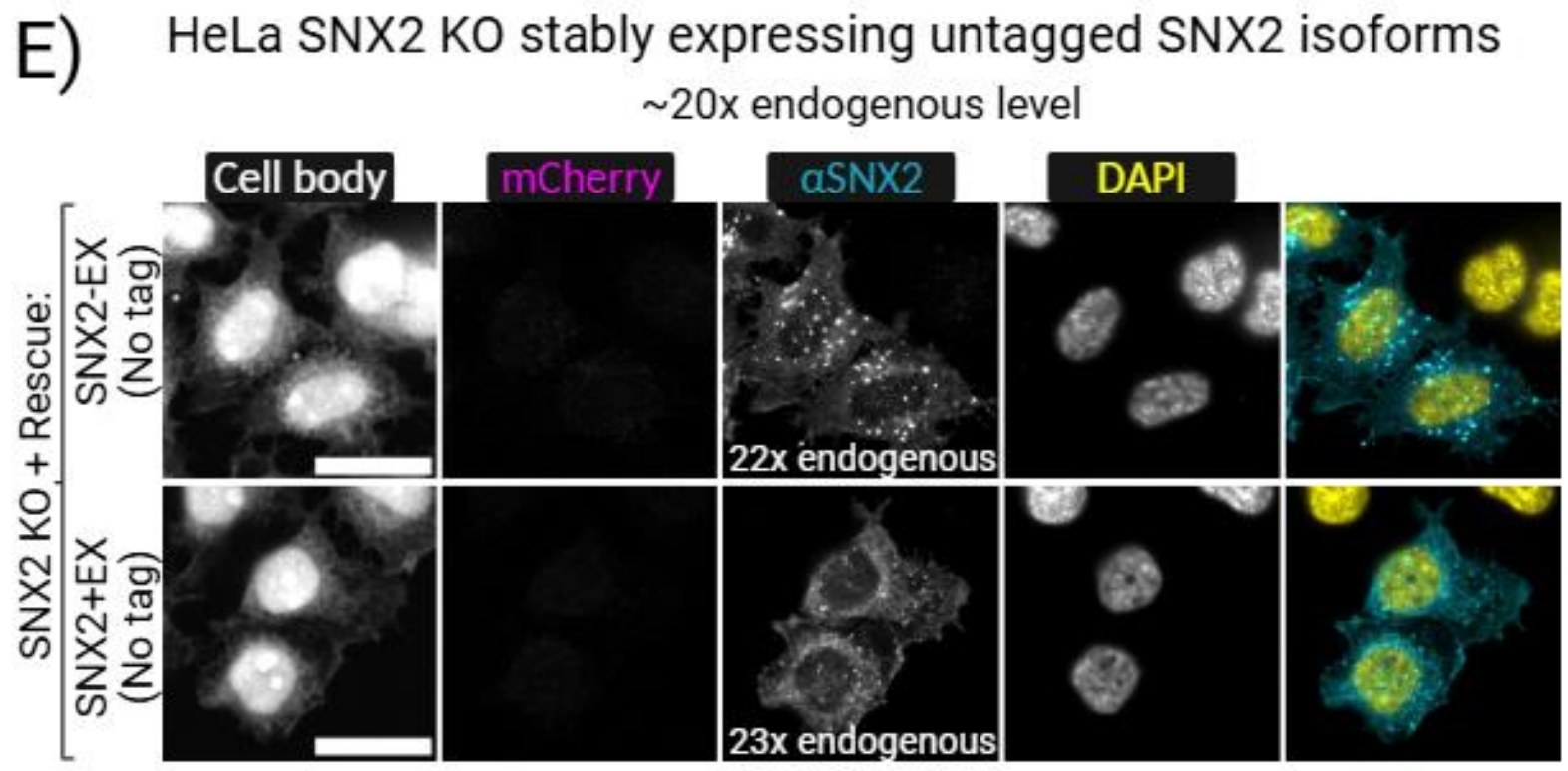

Rebuttal Figure 19 | Representative images of HeLa cell lines over-expressing untagged SNX2 isoforms at 20-25x endogenous levels. The cells are HeLa SNX2 KO stably expressing untagged SNX2+EX or SNX2-EX. The cell body, mCherry, αSNX2, and DAPI channels are shown independently and merged. Representative cells were chosen to have comparable expression levels based on the αSNX2 immunostaining signal. The expression level relative to the HeLa wildtype mean SNX2 level is indicated in the bottom of each image. Scale bar: 25µm.

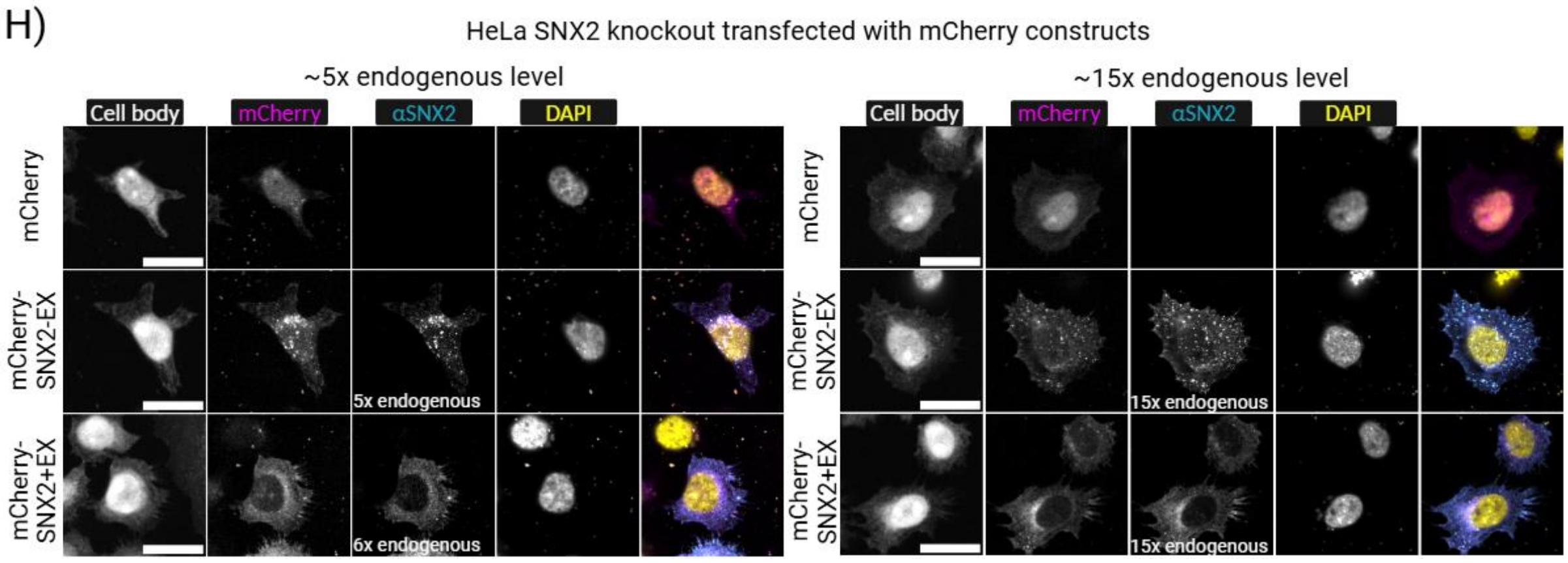

Rebuttal Figure 20 | Representative images of HeLa cell lines over-expressing mCherry-SNX2 isoforms at 5x and 15x endogenous levels. The cells are HeLa SNX2 KO transiently expressing mCherry-SNX2+EX or SNX2-EX. The cell body, mCherry, αSNX2, and DAPI channels are shown independently and merged. Representative cells were chosen to have comparable expression levels based on the αSNX2 immunostaining signal. The expression level of αSNX2 relative to the HeLa wildtype mean SNX2 level is indicated in the bottom of each image. Scale bar: 25µm.

Based on these observations, we do not think that the mCherry tag changes the basic isoform-specific localisation pattern when the proteins are expressed in the endogenous-like range or at lower degrees of over-expression.

<u>Higher level of overexpression:</u>

At higher levels of over-expression, we observe aggregate-like structures for mCherry-SNX2-EX, which were described as swollen endosomes by *Carlton et. al. 2005* [PMID: 16179610] upon over-expressing GFP-SNX2-EX. In contrast, these structures are absent or much less prominent for untagged SNX2−EX at comparable expression levels as exemplified in representative images of cells expressing ~50x endogenous level (Rebuttal Figure 21), and by plotting cells' SNX2 granularity against their SNX2 level (Rebuttal Figure 22). Conversely, we see similar granularity behaviours for mCherry-SNX2+EX and untagged SNX2+EX at these higher levels of overexpression.

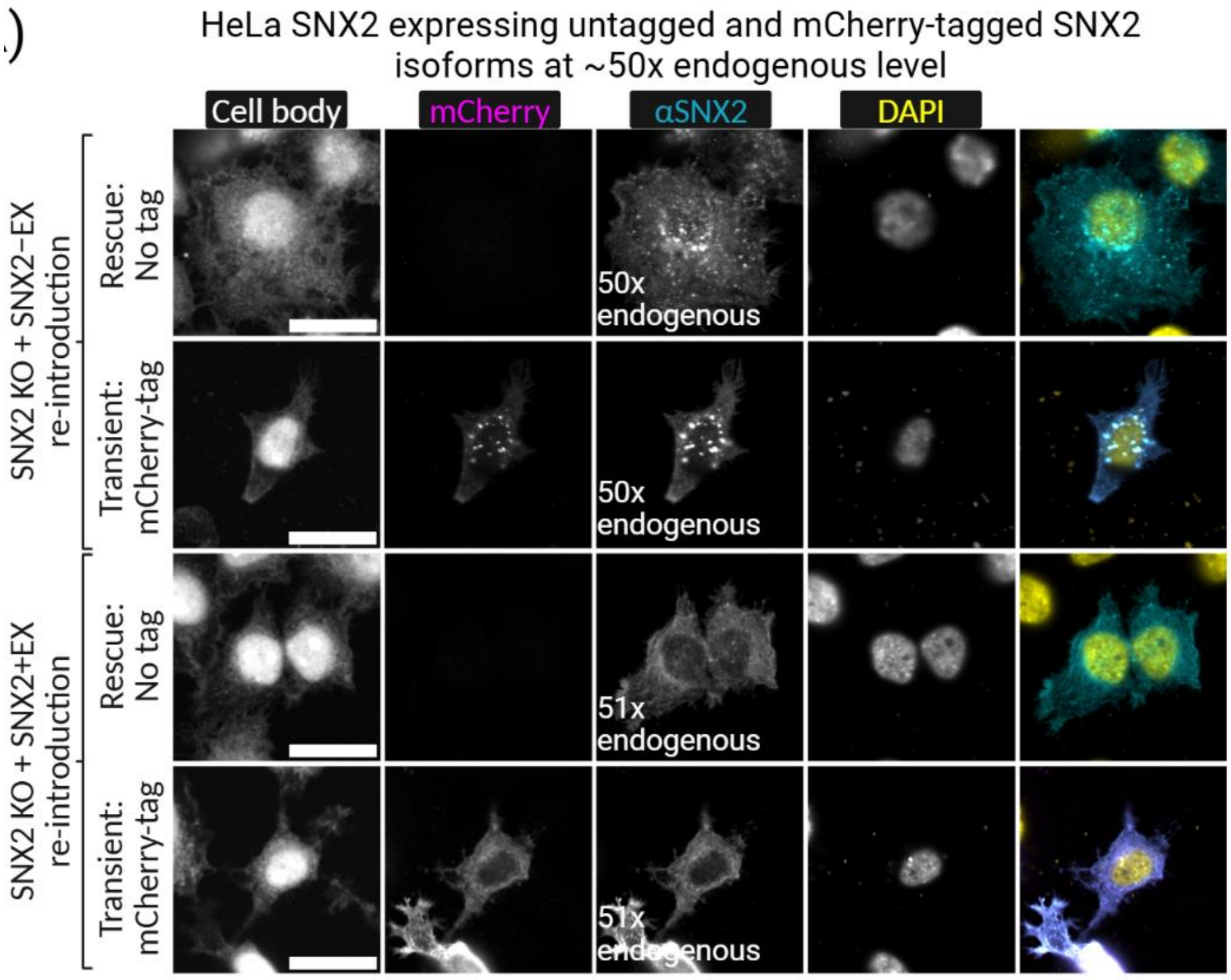

Rebuttal Figure 21 | Representative images of HeLa cell lines over-expressing untagged and mCherry-tagged SNX2 isoforms at 50x endogenous levels. The cells are either HeLa SNX2 KO stably expressing untagged SNX2+EX or SNX2-EX, or HeLa SNX2 KO transiently expressing mCherry-SNX2+EX or SNX2-EX. The cell body, mCherry, αSNX2, and DAPI channels are shown independently and merged. Representative cells were chosen to have comparable expression levels based on the αSNX2 immunostaining signal. The expression level of αSNX2 relative to the HeLa wildtype mean SNX2 level is indicated in the bottom of each image. Scale bar: 25µm.

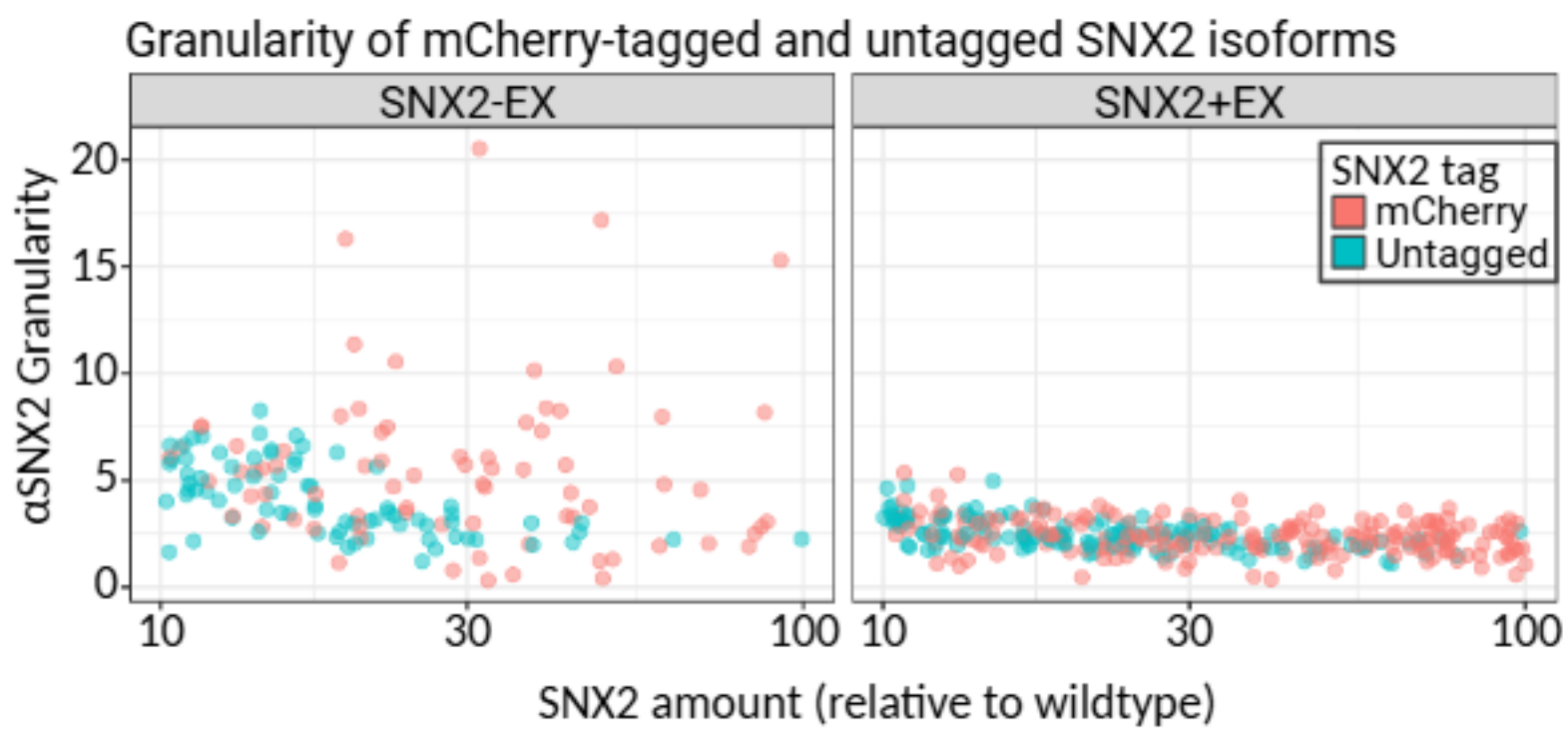

Rebuttal Figure 22 | XY plot of SNX2 granularity versus SNX2 amount for cells over-expressing untagged and mCherry-tagged SNX2 isoforms. Each dot represents a cell with a SNX2 amount of 10-100x that of the population mean of HeLa wildtype. The plot is split according to exon status (SNX2-EX, SNX2+EX), and dot colour indicates SNX2 tag status. n cells: untagged SNX2-EX n=76, mCherry-SNX2-EX n=76, untagged SNX2+EX n=133, mCherry-SNX2+EX, n=196. The cells were pooled from N = 3 experimental repeats.

Thus, we agree that the tag can influence the appearance of the high-expression phenotype, but this effect is selective, expression-dependent, and appears to represent

an exaggeration of the underlying SNX2−EX localisation behaviour rather than a reversal of the isoform difference. Importantly, we do not observe a comparable effect for SNX2+EX.

We have revised the manuscript to highlight these aspects of granularity and the impact of the tag in two ways: First, we have updated Figure 5A to include cells with endogenous-like expression of mCherry-tagged isoforms alongside the untagged rescue lines, which is mentioned in the Results here:

To evaluate the impact of the mCherry-tag on SNX2's behaviour, we next transfected the SNX2 KO cells with the mCherry-SNX2 isoforms. At endogenous-like and lower over-expression levels, we observed similar localisation behaviour between untagged and tagged isoforms (**Fig 5A; Figure S14H**), supporting the use of the tag in examining SNX2 localisation at lower expression levels.

Second, we have added the figures showing the representative images and XY plot illustrating the tag-dependent exaggeration of the SNX2-EX phenotype at high-expression (Rebuttal Figure 21-22) as Figure S16 in the manuscript and revised the Results to state explicitly state this behaviour becomes apparent only at stronger overexpression:

By contrast, at higher expression, aggregate formation was only observed for mCherry-fused SNX2-EX (**Figure S16A-B**), indicating the observed aggregates are tag-dependent, and likely arise from the tag accentuating isoform-specific behaviours, as we didn't observe it for mCherry-SNX2+EX.

Reviewer 2 Comment 12: This raises the question whether it would have been possible to pick up any differences in the localization of SNX2-EX and SNX2+EX in a high content screen without tags and how much the mCherry tag contributes to the observed changes in the localization of different isoforms?

We thank the reviewer for the question and agree it follows naturally from Reviewer 2, Comment 11. As discussed there, our additional analysis indicates that the mCherry-tag doesn't appear to notably affect SNX2 isoform localisation unless examined at higher over-expression levels (discussed in detail in Reviewer 2, Comment 11). We do agree, however, that it is important to ask whether the untagged SNX2 isoforms would be among the top hits if examined via the classifier-based analysis approach.

To examine this, we performed the same classifier-based analysis we had done for the mCherry-tagged isoform pairs, but using the immunostain-based αSNX2 signal measurements from the SNX2 KO rescue cells stably expressing untagged SNX2-EX or SNX2+EX as input. We utilised that the stable populations were not completely pure for SNX2 positive cells (Rebuttal Plot 14, see also Reviewer 2, Comment 7) to define an internal normalisation control consisting of the SNX2 negative cells (similar to non-transfected cells used as the internal normalisation controls for the mCherry isoforms).

We performed the analysis twice, using cells within two expression ranges: An endogenous-like range (intensities of ~0.7x - 1.5x mean of HeLa wildtype population), and a full expression range (~0.7x endogenous level to ~100x endogenous level). This stratification allowed us to consider the impact of over-expression besides the impact of the tag.

The single cell SNX2 intensities of the rescue cells within these ranges are shown below (Rebuttal Figure 23), alongside the normalised ROC-AUC values for the classifier-based analysis (Rebuttal Figure 12, same plot as Reviewer 2, Comment 2+3)

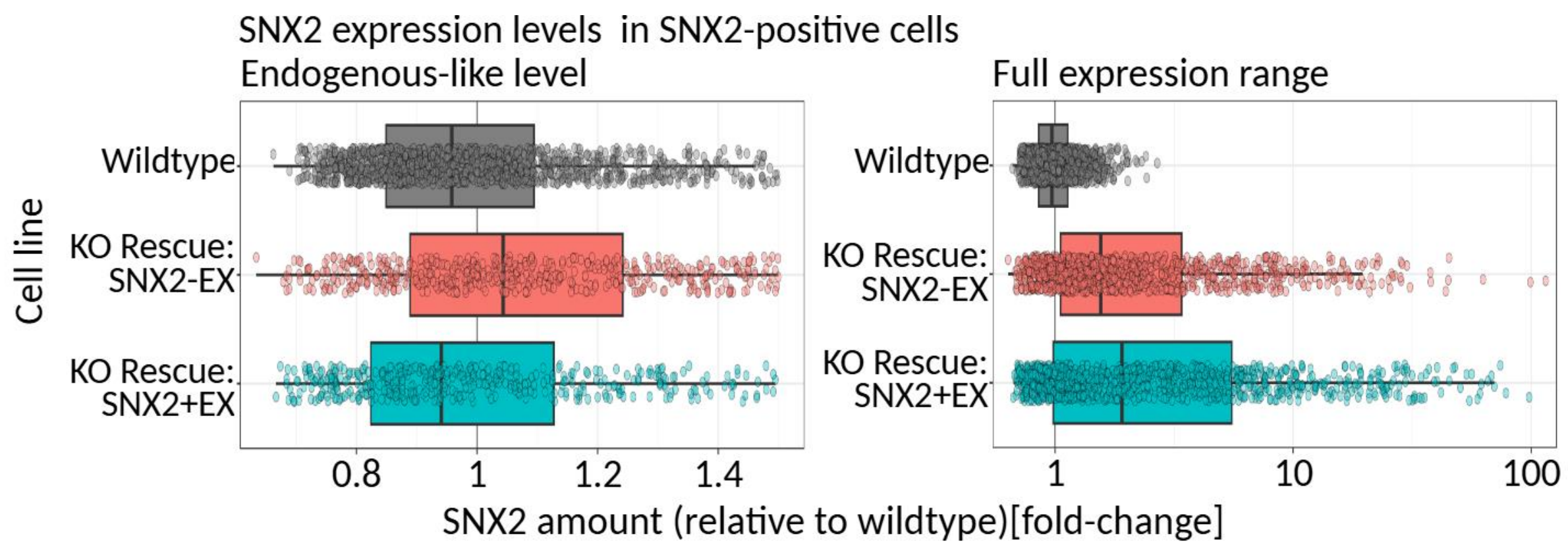

Rebuttal Figure 23 | Creating SNX2 rescue cells populations expressing untagged SNX2 isoforms at endogenous-like levels and full expression ranges. Boxplot and dotplot showing the SNX2 amount within HeLa wildtype cells or HeLa SNX2 KO cells stably expressing untagged SNX2-EX or SNX2+EX, relative to the population mean of HeLa wildtype. The endogenous-like level contains cells expressing SNX2 at ~0.7 to 1.5x that of HeLa wildtype. The full expression range contains cells expressing SNX2 at ~0.7-100x. Colours indicate cell line (grey = HeLa wildtype, red=Rescue SNX2-EX, blue=Rescue SNX2+EX).

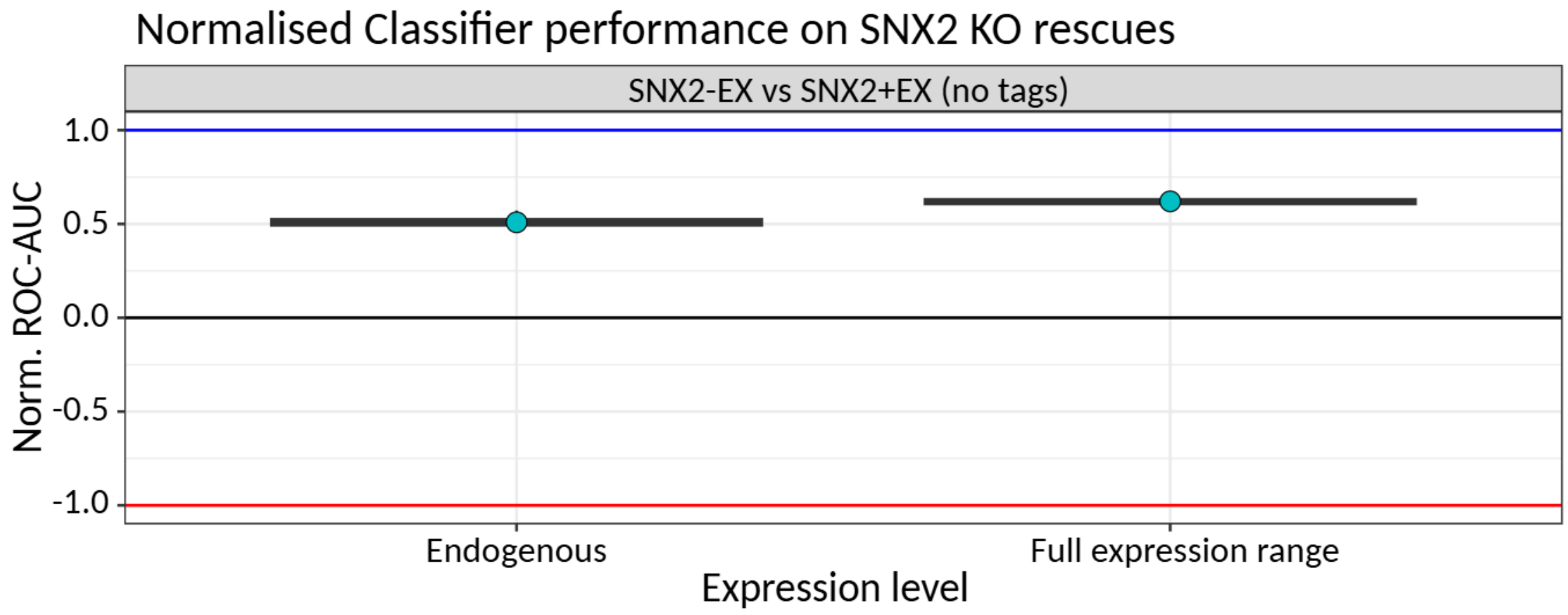

Rebuttal Figure 12 | Comparable classifier performance for SNX2 rescue cells at endogenous-like and full expression ranges. Boxplot showing the normalised ROC-AUC values from classifier-based discrimination of HeLa SNX2 rescue cells expressing untagged SNX2−EX and SNX2+EX based on the SNX2 signal. The analysis was performed using cells expressing the SNX2 isoforms at either endogenous-like levels (~0.7-1.5x the mean SNX2 intensity in HeLa wildtype, n cells = 418 per isoform) or the full measured expression range (~0.7-100x the mean SNX2 intensity in HeLa wildtype, n cells = 763 per isoform). The cell sampling and classifier evaluation was performed 50 times, and

the boxplot shows the distribution of the normalised ROC-AUCs from these iterations. The dot indicates the median (n = 50).

We found this approach confidently separated the untagged SNX2 isoforms, with normalised ROC-AUC values of approximately 0.5 in the endogenous-like range and 0.6 across the full expression range. Moreover, these scores suggest that the SNX2 isoform pair would have been a strong hit in the screen in the absence of the mCherry tag, even at endogenous-like levels.

We have added the cell intensity distributions (Rebuttal Figure 23) as Figure S14G and the classifier-based scoring (Rebuttal Figure 12) as Figure 5C in the manuscript, and included the following text in the Result section:

In agreement with this, our classifier-based approach confidently assigned correct isoform identities to the rescue cells at both endogenous-like levels (norm. ROC-AUC = ~0.5) and across the full expression-range of the rescue cell populations (norm. ROC-AUC = ~0.6) (**Figure 5C; Figure S14G**), supporting the differential localisation of untagged SNX2 isoforms.

With respect to the broader question of how much the mCherry tag may contribute to localisation differences for other isoform pairs, we agree that this is an important general limitation. In most cases, direct evaluation is challenging due to the sparsity of affinity reagents, such as antibodies that show isoform-specificity, which generally makes selective visualisation of isoforms difficult. This can be overcome by tagging specific isoforms (as we have generally done here) or by forcing cells to express only one isoform, that can then be visualised via an antibody that recognises all isoforms, as demonstrated for the SNX2 isoforms. Experimental data on isoform localisation is therefore generally limited.

Importantly, our experimental design compares isoforms that are tagged in the same way and examined over comparable expression level ranges (see also Reviewer 2 Comment 4). We therefore interpret any observed localisation difference as being isoform-specific as the only difference between the isoforms in a pair will be the amino acids encoded by the alternative exon, while recognizing that tags may influence the magnitude of some phenotypes.

We have now made this limitation explicit in the revised Discussion:

Second, the imaging-based localisation assays were performed primarily under transient over-expression of the mCherry-tagged isoforms rather than conditions that fully recapitulate endogenous stoichiometries. Although this enabled controlled comparison of matched isoform pairs, overexpression may modify phenotypes that depend on interaction-partner abundance, while tagging may also affect localisation. We addressed this by controlling for expression range in the analysis, comparing isoforms tagged in identical ways, and, for SNX2, observed a similar qualitative isoform-specific difference in an untagged rescue setting at endogenous-like expression, albeit with smaller magnitude. Thus, the assay likely captures intrinsic sequence-dependent localisation differences, while the strength of these effects may vary with expression context.

<u>Reviewer 3 Comment 1:</u> The analyses in Figures 1-2 are performed on highly-selective source data that will undoubtedly bias the resulting conclusions. For the most part these biases are acknowledged, but a more explicit discussion of these points would be in order. For example, by only studying exons that overlap mapped interaction sites, the results will be biased toward over-estimating interaction effects. On the other hand, they will miss potential roles for 'allosteric' splicing regulation where interaction effects occur due to distant amino acid changes. Also, by only including frame-preserving splicing changes, any frame-altering splicing events that might change interaction or localization properties will be missed.

We thank the reviewer for this comment and agree that the analyses in Figure 1-2 are necessarily shaped by the selectivity of the underlying annotation space and the scientific literature in general.

Our framework is intentionally focused on AS events that can be linked directly to mapped interaction interfaces, as this increases the interpretability at the level of physical interaction rewiring. However, this also means that the resulting estimates should not be interpreted as a complete census of all splicing-dependent interaction effects.

As the reviewer correctly highlights, we only consider exons that overlap annotated interaction interfaces in our analysis. This approach will therefore miss changes mediated indirectly through allosteric or other distal structural effects (some of which we have discussed previously [PMID: 38036695]), as well as events acting through incompletely annotated disordered region. In addition, because our primary analysis was restricted to frame-preserving events, it will also miss frame-altering events such as those that remodel the C-termini.

We have therefore now clarified in the Discussion of the revised manuscript that conclusions in Figure 1-2 apply specifically to the subset of frame-preserving AS events overlapping currently annotated interaction interfaces and should be interpreted as a physically grounded but incomplete view of AS-mediated rewiring. We have further note that frame-altering isoforms, disorder-mediated interactions, and distal structural effects may remodel interactions or localisation and make up a broader landscape of AS-mediated rewiring, but such effects will be missed in our analysis effects:

In addition, our framework was intentionally limited to frame-preserving AS events that directly overlap annotated interfaces, providing a physically interpretable view of direct interface rewiring but excluding indirect effects mediated through allosteric or other distal structural changes, as well as frame-altering isoforms that may remodel interaction capacity or localisation through truncation or larger-scale architectural changes. Our conclusions should therefore be interpreted in the specific context of direct, interface-overlapping, frame-preserving AS events, which represent a tractable subset of the broader landscape of AS-mediated rewiring.

<u>Reviewer 3 Comment 2:</u> Figure 1, in particular panels C and onward, would benefit from better presentation to allow the network-mathematical approaches to be more readily understood and interpreted by a structure and molecular biology reading audience

We thank the Reviewer for this comment, and agree the presentation was not clear enough.

To aid in the interpretation of Figure 1, we have created a schematic that provides visual illustration of the network-mathematical approaches used.

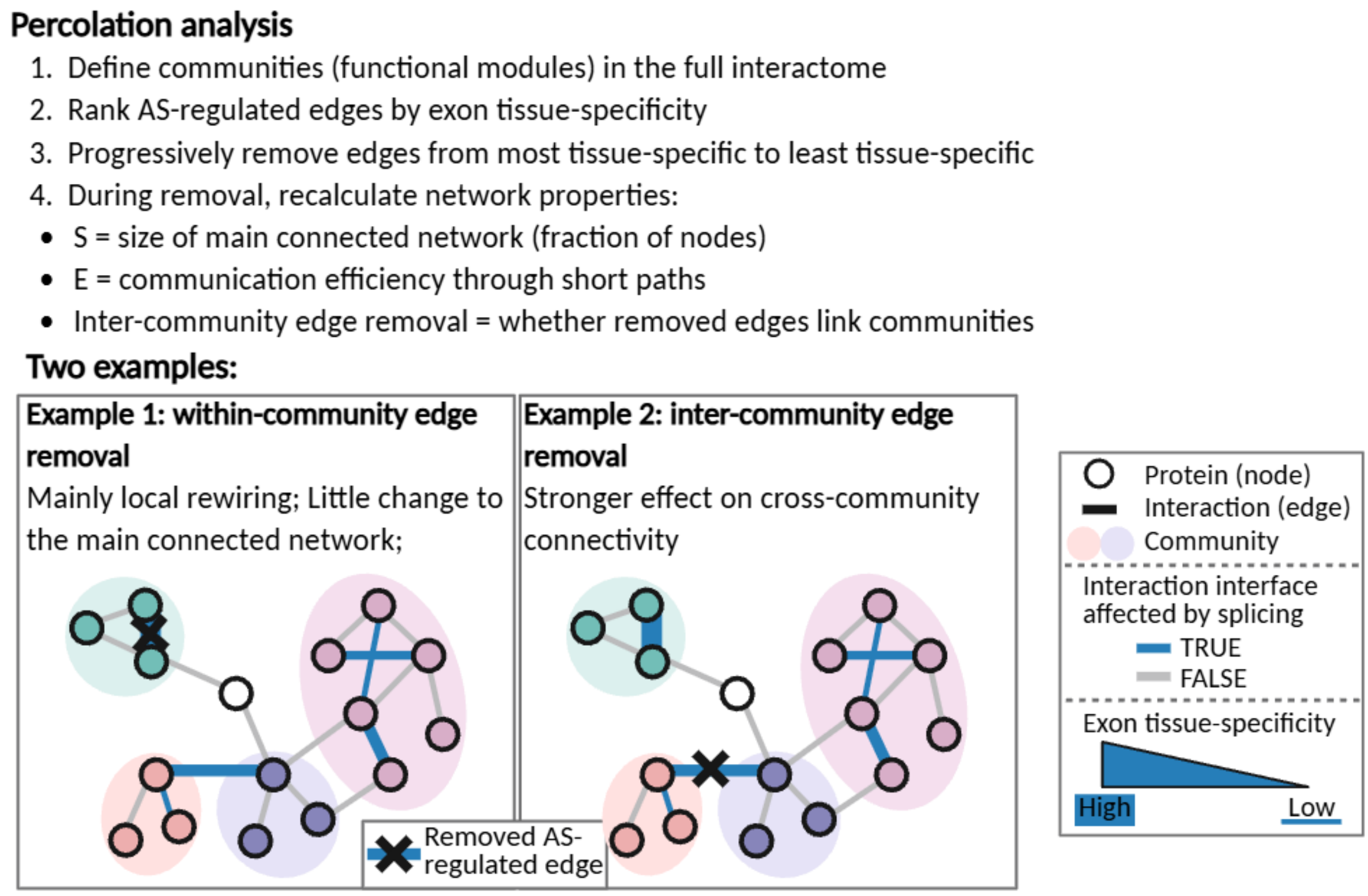

Rebuttal Figure. 3 | Overview of the percolation analysis used to examine the interaction network and the impact of alternative splicing events overlapping interaction interfaces on network wiring. Communities refer to densely connected proteins and therefore often represent functional modules, such as proteins in the same pathway. AS-regulated edges were ranked by tissue-specificity and progressively removed, and network properties were recalculated at each step. Two examples illustrate removal of an intra-community edge versus an inter-community edge.

We have also provided more information on each of the panels in Figure 1 to help guide the reader. For example, for Figure 1D (which was Figure 1C in the original manuscript):

The sigmoidal shape of the ΔS trajectory suggests that the effect of progressive edge removal is not uniform across the ranked AS events: early removal of the most tissue-specific edges has relatively little effect on the main connected part of the interactome, whereas later removal produces a sharper transition once a more critical set of integrative edges is affected.

In addition, we have extensively rewritten the results section for Figure 1 to make it more accessible for the structural and molecular biology reading audience.

Because our goal was to understand how transcript diversity translates into protein interaction rewiring across cellular contexts, we next investigated whether alternative splicing reshapes interaction topology at the systems level. To do this we examined how AS-regulated interfaces contribute to network organisation using percolation analysis and inter-community edge analysis (**Figure 1C**). In this framework, AS-regulated edges were progressively removed from the interface-resolved interactome in order of decreasing exon tissue-specificity, beginning with the most strongly tissue-regulated exons and ending with more tissue-wide events, defined here as exons detected across many tissues but still showing substantial tissue-to-tissue differences in inclusion. At each removal step, we quantified two complementary properties of the interactome network: we quantified S, the fraction of nodes remaining in the main connected part of the interactome, which captures whether the network stays largely intact rather than breaking into smaller disconnected pieces, and E, the approximate global efficiency, which captures how efficiently information can still travel across the remaining network. These observed trajectories were then compared with degree-matched random edge-removal null models to determine whether AS-regulated interfaces have non-random effects on network structure.

Removal of the most strongly tissue-specific AS edges caused a significant deviation from the degree-matched null expectation in the fraction of nodes retained in the main connected part of the interactome network, but in a direction indicating less fragmentation than expected by chance (**Figure 1D**, ΔS: empirical two-sided $p < 0.001$). The sigmoidal shape of the ΔS trajectory suggests that the effect of progressive edge removal is not uniform across the ranked AS events: early removal of the most tissue-specific edges has relatively little effect on the main connected part of the interactome, whereas later removal produces a sharper transition once a more critical set of integrative edges is affected. Together with the absence of a significant effect on approximate global efficiency (**Figure S1E**, ΔE: $p > 0.05$), this pattern is most consistent with highly tissue-specific exons preferentially rewiring interactions locally rather than a broad disruption of network-wide communication. We next examined whether AS-regulated edges preferentially connect distinct functional communities, that is, groups of proteins that are more densely connected to one another than to the rest of the interactome. Consistent with network S, tissue-specific AS-regulated edges were depleted among edges linking distinct functional communities, whereas tissue-wide AS events significantly altered the fraction of edges linking distinct communities (**Figure S1F**, empirical two-sided $p < 0.002$). This pattern suggests tissue-wide events may contribute more strongly to maintaining connections between functional modules. Taken together, these findings are consistent with a model in which tissue-specific exons mainly fine-tune local network structure, whereas more tissue-wide exons have greater influence on inter-community integration.

Among tissue-specific events, microexons (< 30 nt) represent a distinct class of AS events in terms of effect on protein structure. Unlike longer exons, microexons frequently alter the structured domains of proteins[29], suggesting that they may exert different effects on interaction topology. We therefore tested whether microexons displayed a distinct pattern of network regulation. In contrast to the broader class of tissue-specific exons, which were associated primarily with local rewiring, microexons were significantly enriched among interfaces linking distinct functional communities (**Figure 1E; Figure S1G-H**, $p < 0.004$, empirical two-sided). This suggests that microexons preferentially regulate connections

between pathways or complexes rather than acting only within local modules. Ordinary least-squares (OLS) modelling (see Methods) further showed that inter-community connectivity depended on both tissue regulation and local topology, with a significant regulation × microexon interaction term (**Figure S1I**, $p < 0.03$, OLS t-test). Together, these observations are consistent with microexons representing a specialised subset of tissue-specific exons with a disproportionate potential to occupy network positions that modulate crosstalk between different complexes or subcellular locations.

Tissue-specific splicing preferentially targets multi-domain proteins with broad interaction and localisation repertoires

We next asked whether the network effects associated with tissue-specific splicing were linked to specific protein properties. Regression and machine-learning analyses identified multi-domain architecture[31], interaction-substrate diversity, post-translational modification[32] density, and shared-neighbour connectivity as the strongest predictors of ΔS (**Figure 1F**, all $p < 10^{-5}$, Heteroscedasticity-robust OLS (HC3) Wald test), suggesting that tissue-specific AS may preferentially affect modular, multifunctional proteins. To determine whether these trends extended beyond the interface-overlapping subset, we then analysed all frame-preserving, tissue-specific AS events irrespective of direct interface overlap. Consistent with the interface-level results, tissue-specific events were enriched in multi-domain proteins (**Figure 1G**, $p < 3.47 \times 10^{-59}$, Kruskal–Wallis test) and in proteins with greater interaction-substrate diversity (**Figure 1H**, $p < 1.62 \times 10^{-16}$, Kruskal–Wallis test). Similarly, when proteins were grouped into broad categories based on cellular function, alternative splicing propensity increased with the median number of unique domains per protein (**Figure 1I**, $R = 0.85$, $p = 0.0034$, Spearman's rank). Together, these analyses indicate that tissue-specific AS preferentially affects modular, multifunctional proteins that are likely to tolerate local changes in binding surfaces or domain arrangement without disrupting core activity.

A potential consequence of altered interaction behaviour is altered subcellular localisation[33]. We therefore next asked whether proteins regulated by tissue-specific AS also show greater spatial diversity within the cell. Integrating subcellular localisation annotations from the Human Protein Atlas[34], we found that proteins containing frame-preserving tissue-specific AS events were enriched among proteins localised to multiple cellular compartments (**Figure 1J**, $p < 0.0029$, Kruskal–Wallis test). Likewise, proteins with tissue-specific interface-regulating events showed higher localisation diversity (**Figure 1K**, all tissue-specific exons $p < 5.543 \times 10^{-17}$, Kruskal–Wallis across regulation bins). This association was strongest among highly modular proteins (**Figure 1L**, $\beta = 1.83$, $p = 9.5 \times 10^{-13}$, OLS t-test) and remained significant when proteins were analysed separately according to modularity (**Figure S1J**, high-modularity: $\beta = 0.58$, $p = 2.7 \times 10^{-15}$, OLS t-test). Microexon-containing proteins also showed elevated localisation diversity, although this association was attenuated at inter-community interfaces (**Figure 1M**, $\beta = 0.49$, $p < 0.001$, OLS t-test). Overall, these data support a link between tissue-specific AS and broader subcellular localisation diversity of proteins.

To move from this gene-level association to the level of individual splicing events, we next asked how often tissue-regulated exons might influence localisation by predicting localisation shifts between matched isoforms with and without the regulated exon using ProtGPS[35]. By this approach, 27.7% of tested exons (n = 7,979) were associated with a predicted redistribution across subcellular compartments (**Figure S1K**). Although this analysis is predictive rather than experimental, it suggested that exon-associated localisation changes

may be sufficiently common to justify direct experimental testing, motivating the imaging-based screen described below.

Relatedly, we have added titles to most plots in Figure 1 and Figure S1, added/updated axes descriptions, updated the legends, and updated the overall graphics to improve presentation and clarity.

Reviewer 3 Comment 3: It might be interesting to know: do interaction-affecting or localization-affecting AS events tend to occur e.g. near the beginning of a transcript, or in the middle, or at the 3′ end?

We thank the reviewer for this suggestion. To address this question, we carried out two new analyses aimed specifically at testing whether interaction-affecting AS events or localisation-affecting AS events show positional bias within proteins, and whether this is related to occurrence in intrinsically disordered regions (IDRs).

For the interaction-related analysis, we asked whether interface-overlapping alternative exons showed a bias in their relative position within the transcript compared to a background set of annotated alternatively spliced exons from the VastDB database. This background set comprised of alternatively spliced exons, as defined by percent spliced in (PSI) value between 5 and 95, in at least one sample across 75 cell types and tissues. We divided each coding sequence into four equal bins, corresponding to 0–25%, 25–50%, 50–75%, and 75–100% of the open reading frame, and compared the distribution of interface-overlapping exons with the background set across these bins. Despite a slight general bias for all alternative exons to occur towards the N-terminus, we identified no positional bias for interface-affecting exons compared to our background (p = 0.116. Chi-square test) (Rebuttal Figure 24) suggesting AS events encoding interfaces do not display a simple positional bias. We therefore decided not to include this analysis in the manuscript, although we are happy to do so at the editor's / reviewer's discretion.

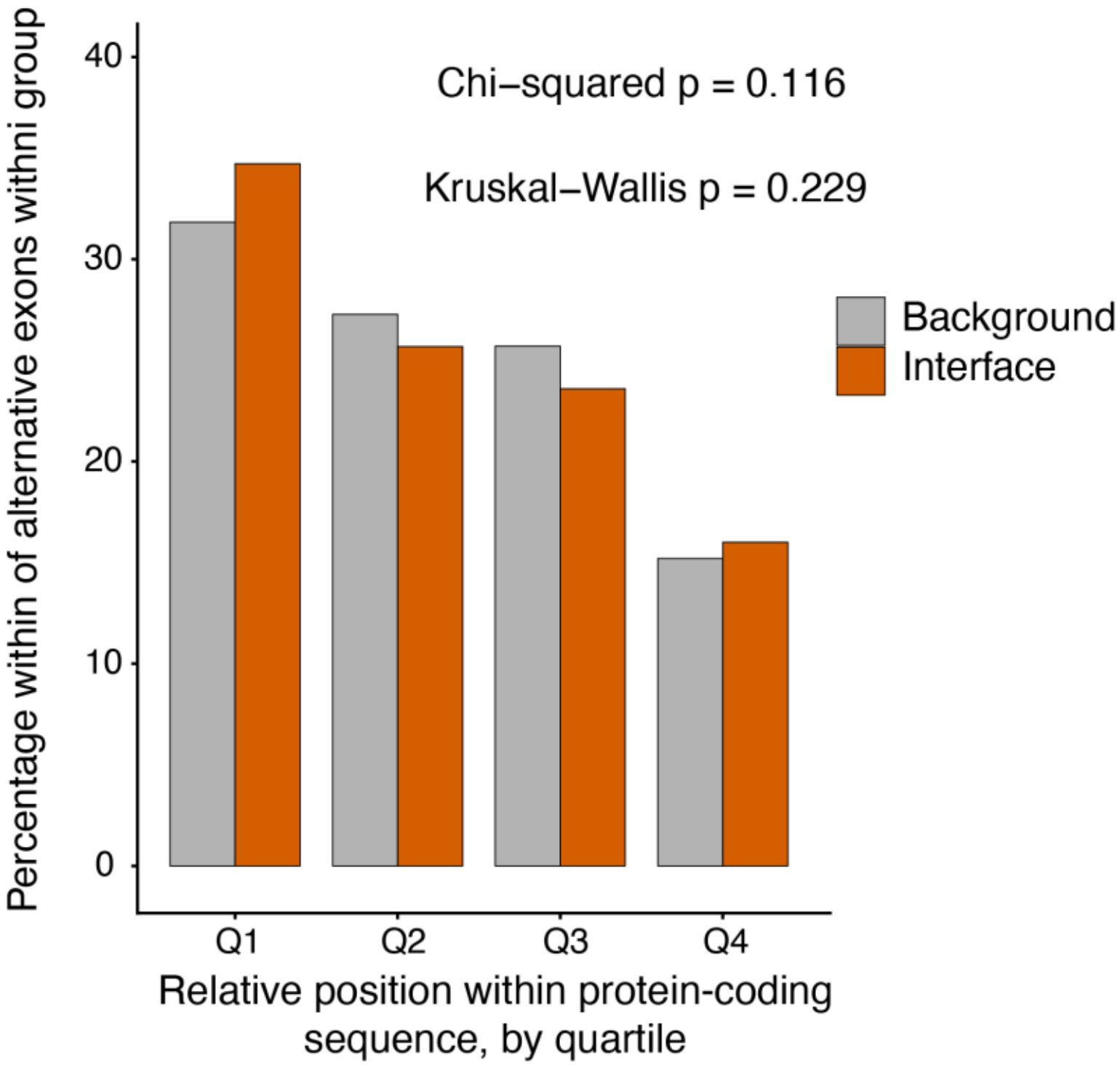

Rebuttal Figure 24 | Relative of interface-overlapping alternative exons within proteins. Protein-coding alternative exons annotated in vastDB were assigned to quartiles according to their relative position within the encoded protein sequence, from the N-terminal quarter to the C-terminal quarter. The distribution of all protein-coding alternative exons was compared with the subset of alternative exons overlapping annotated protein interaction interfaces. Bars show the percentage of exons from each group falling within each protein-position quartile. Interface-overlapping alternative exons showed a broadly similar positional distribution to the full set of coding alternative exons, with no significant difference detected by chi-squared test or Kruskal–Wallis test.

To examine positional bias for localisation-affecting AS events, we analysed our tissue-specific AS exon dataset using the recently published protein localisation predictor ProtGPS (Kilgore *et al.,* 2025 [PMID:39913643]). ProtGPS uses protein language model embeddings to infer localisation probabilities across 12 condensate-associated subcellular compartments from sequence. For each event, we compared matched protein isoforms with and without the tissue-regulated exon and quantified the resulting localisation shift using the Wasserstein distance (WD), which captures the magnitude of redistribution across predicted compartments. Using a threshold of WD > 0.1, consistent with the original ProtGPS study, we found that 27.7% of tested exons (n = 7,979) were associated with a predicted localisation change (Rebuttal Figure 25). We have added this analysis to the manuscript as together with the cumulative distribution plot shown below (Rebuttal Figure 25).

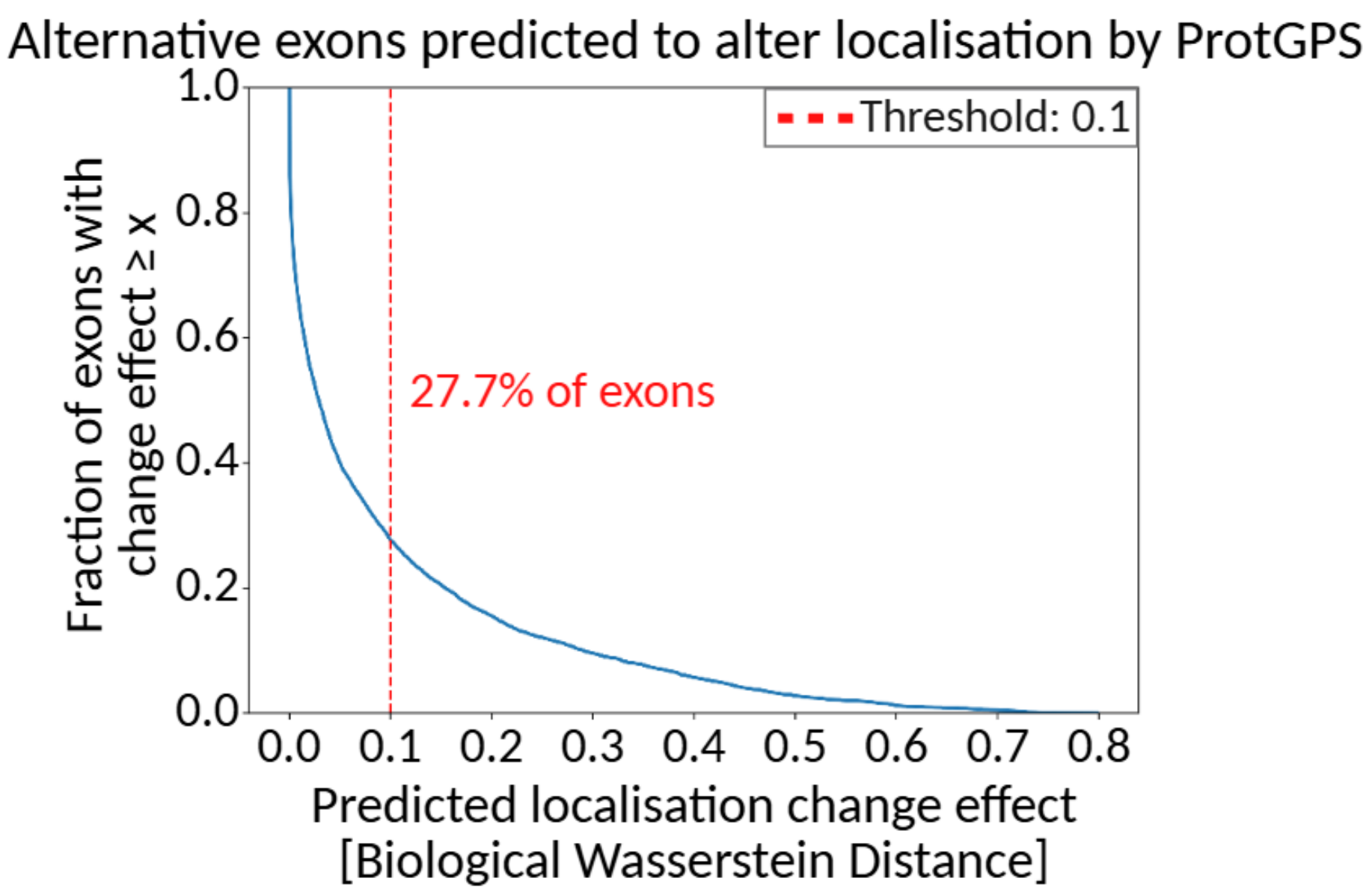

Rebuttal Figure 25 | Distribution of localisation shift magnitudes across exons. ProtGPS-predicted localisation differences to 12 subcellular compartments for pairs of protein isoforms that differ in the inclusion or exclusion of a tissue-regulated exon. The predicted values are shown as a cumulative distribution plot of the biological Wasserstein distance (WD) values for tested isoform pair (n=7,979) and shows the fraction of isoform pairs with localisation differences ≥ to a given magnitude. Isoform pairs with WD ≥ 0.1 (red, dashed line) are considered to show localisation differences, and so the splicing event is considered localisation-affecting. The used cutoff was the default ProtGPS cutoff.

We then asked whether these localisation-affecting exons were biased according to their position within the encoded protein (Rebuttal Figure 26) or their overlap with IDRs (Rebuttal Figure 27). To reduce this to a formal test, we used a logistic regression model with localisation change as the response variable and both normalised protein position and IDR status as predictors. This analysis identified a weak but statistically significant association with protein position ($p < 5.24 \times 10^{-13}$), with more C-terminal exons being somewhat less likely to associate with predicted localisation change, and a weaker contribution from IDR status ($p < 0.0042$). However, the overall explanatory power of the model was very limited (AUC = 0.56, R^2 = 0.011), indicating that neither feature explains much of the variance across events. We therefore interpret these effects as modest trends rather than strong determinants.

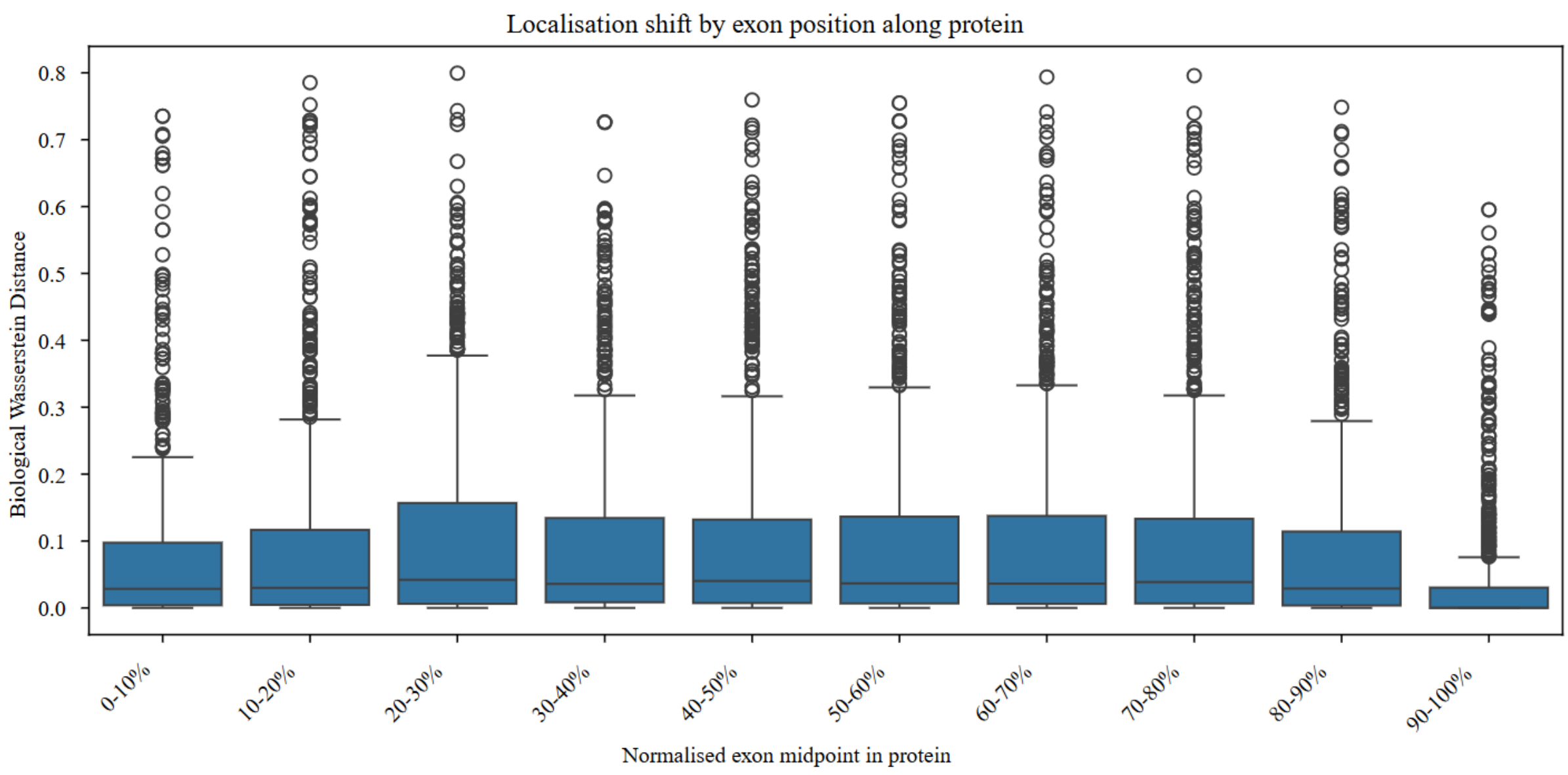

Rebuttal Figure 26 | Fraction of localisation-changing exons by protein position. Fraction of exons classified as localisation-changing (WD > 0.1) across bins of normalised exon midpoint position within the encoded protein. Protein length was divided into deciles from N- to C-terminus, and the proportion of exons exceeding the localisation-change threshold was calculated for each bin (n= 7,979).

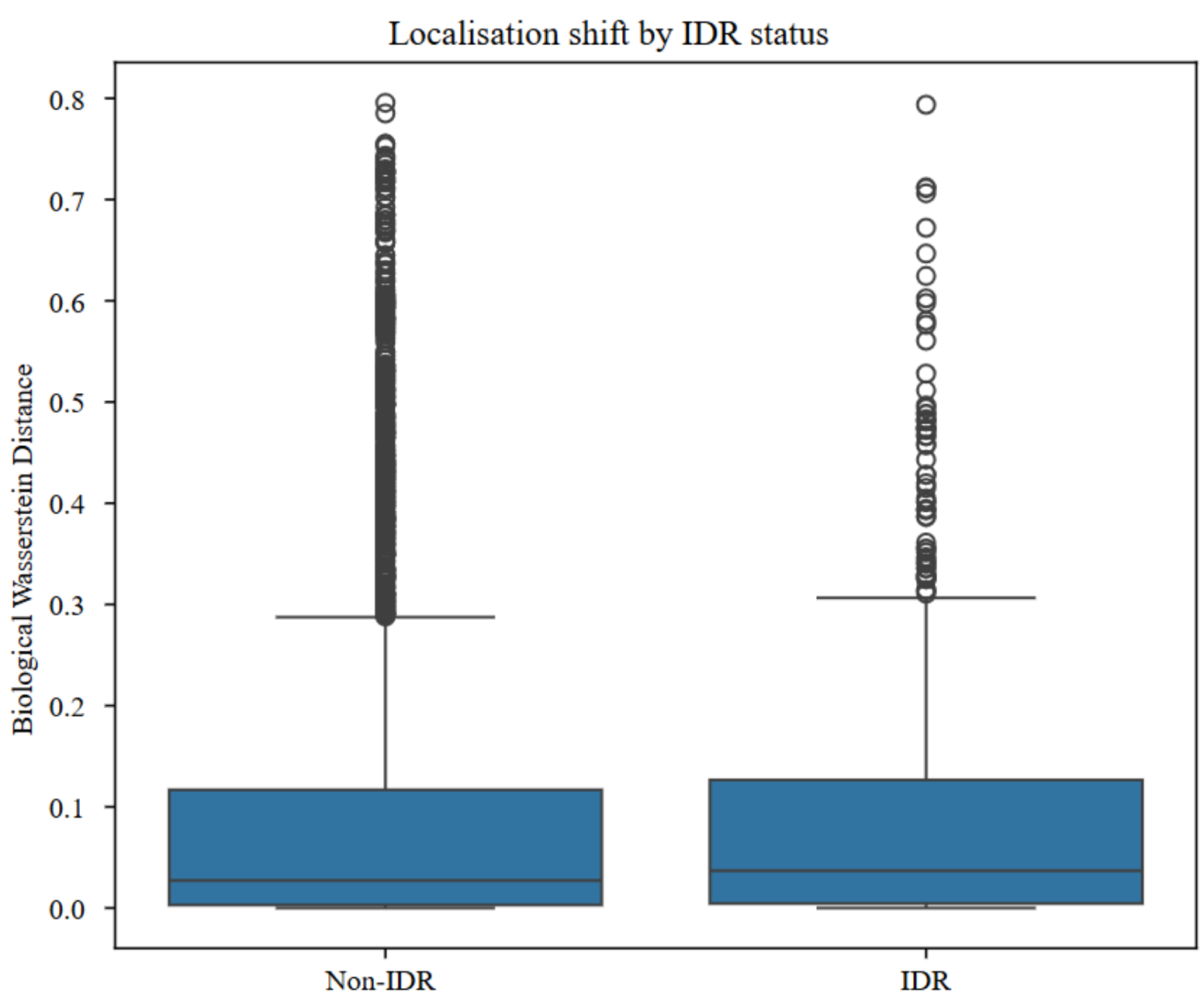

Rebuttal Figure 27 | Localisation shift by IDR status. Boxplot showing the distribution of biological Wasserstein distance (WD) values for exons located within intrinsically disordered regions (IDRs) or outside IDRs (non-IDRs). Each point represents an exon, centre lines indicate medians, boxes indicate the interquartile range, and whiskers extend to 1.5× the interquartile range. This plot compares the magnitude of exon-associated localisation shifts between IDR and non-IDR contexts (n = 7,979)

Overall, this follow-up analysis suggests that localisation-affecting exon events do not show a strong positional constraint within proteins, although there may be a modest tendency for such events to be less common toward the C-terminus. Given the limited predictive power of the formal model, we have included the overall ProtGPS localisation-shift analysis in the revised manuscript (Rebuttal Figure 25 included as Figure S1K in the manuscript) but have not added the position and IDR analyses to the main text to avoid over-interpreting weak effects. We would, however, be happy to include these additional analyses in the Supplementary Information if the reviewer or editor feels they would be valuable.

We have added the following section to the Results on the ProtGPS results:

To move from this gene-level association to the level of individual splicing events, we next asked how often tissue-regulated exons might influence localisation by predicting localisation shifts between matched isoforms with and without the regulated exon using ProtGPS[35]. By this approach, 27.7% of tested exons (n = 7,979) were associated with a predicted redistribution across subcellular compartments (**Figure S1K**). Although this analysis is predictive rather than experimental, it suggested that exon-associated localisation changes may be sufficiently common to justify direct experimental testing, motivating the imaging-based screen described below.

And the following information for how the analysis was conducted to the Methods section:

ProtGPS prediction analysis

For protein localisation analysis, alternative isoforms were compared using ProtGPS, a protein language model built on ESM2 that predicts the independent probability that a protein sequence localises to each of 12 condensate-associated subcellular compartments[34]. The model can report probabilities for compartments including, for example, the nucleolus, nuclear speckles, chromosome, and nuclear pore complex. For each exon, genomic coordinates were mapped to UniProt protein coordinatses, and the canonical UniProt sequence was compared with a matched sequence in which the protein segment encoded by the alternative exon was either included or excluded, yielding a +EXON and −EXON isoform pair. ProtGPS probabilities were calculated for both isoforms, and a compartment was considered positively predicted when its ProtGPS probability exceeded 0.83, a conservative threshold chosen to match the lower bound of the model's reported held-out performance. To quantify localisation change between isoforms, we calculated the Wasserstein distance (WD) between their 12-dimensional ProtGPS probability distributions. As used in the ProtGPS study[34], WD provides a measure of dissimilarity between two probability distributions, with larger values indicating a greater predicted redistribution across compartments. Exons with WD > 0.1 were classified as localisation changing.

In addition, the relevant scripts for this analysis have been uploaded to the study GitHub page (https://github.com/weatheritt2/Hansen_AS).
